# Synthesis of Thiaphenanthridinones from Sulfinate Esters and 2-Borylanilines

**DOI:** 10.1021/acs.orglett.4c03420

**Published:** 2024-11-06

**Authors:** Keisuke Nakamura, Minori Suzuki, Suguru Yoshida

**Affiliations:** Department of Biological Science and Technology, Faculty of Advanced Engineering, Tokyo University of Science, 6-3-1 Niijuku, Katsushika-ku, Tokyo 125-8585, Japan

## Abstract

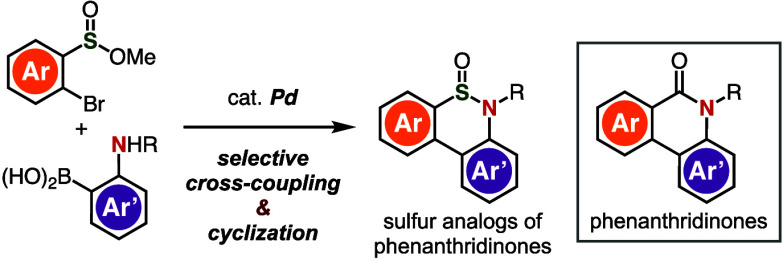

A facile palladium-catalyzed synthesis of thiaphenanthridinones from sulfinate esters and 2-borylanilines is disclosed. Various sulfur analogs of phenanthridinones were synthesized by bromide-selective cross-coupling and cyclization in one step. Further transformations of the obtained thiaphenanthridinones allowed preparing a broad range of thiaphenanthridinone derivatives involving analogs of bioactive compounds.

Heteroatom-analogs of bioactive compounds are gaining increasing attention in various fields of research including pharmaceutical sciences, agrochemistry, and chemical biology.^1^ Various bioactive molecules that contain a range of heteroatoms such as silicon,^1a^ phosphorus,^1b^ and boron,^1c^ have been developed so far. Here, we disclose an efficient synthesis of thiaphenanthridinones,^2^ i.e., sulfur analogs of phenanthridinones, wherein the sp^2^ carbon is replaced with an sp^3^ sulfur, from sulfinate esters and *o*-borylanilines (Figure 1A). As increasing F_sp3_ in bioactive compounds is highly desirable,^3^ we focused on the unusual compound class of thiaphenanthridinones. While a wide range of phenanthridinones^4,5^ and their sulfonyl analogs, i.e., dibenzosultams,^6,7^ have been identified as promising drug candidates, the chemistry of thiaphenanthridinones has remained unexplored so far (Figure 1B).

**Figure 1:**
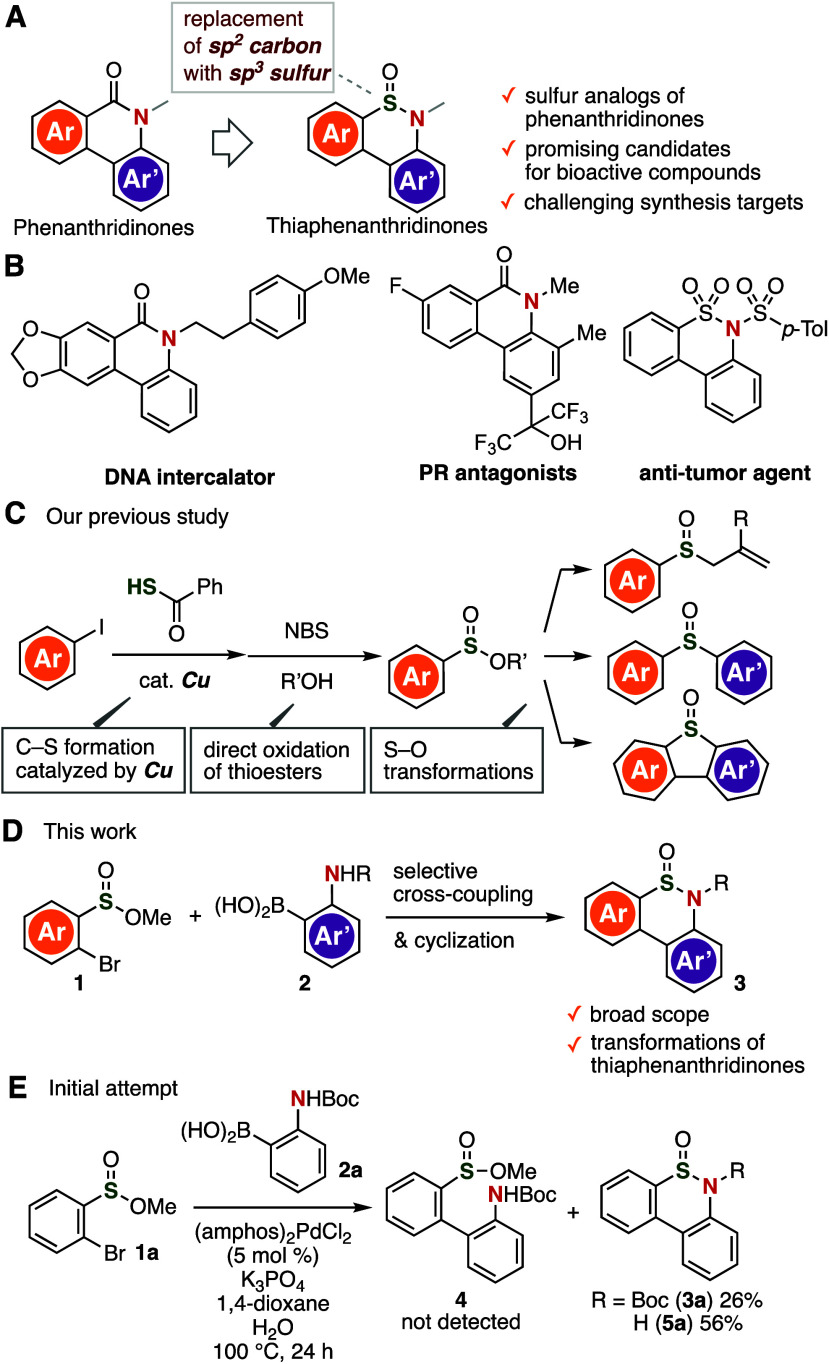
(A) Phenanthridinones and thiaphenanthridinones. (B) Bioactive phenanthridinones. (C) Our previous study. (D) This work. (E) Initial attempt. Amphos = *t*-Bu_2_PC_6_H_4_-4-NMe_2_.

The facile synthesis of diverse thiaphenanthridinones from easily accessible *o*-bromophenyl-substituted sulfinate esters was planned during our recent explorations into organosulfur chemistry (Figure 1C).^8,9^ For example, we have developed an efficient synthetic route to sulfinate esters from the corresponding aryl iodides.^8^ Moreover, novel transformations of sulfinate esters realized the preparation of various sulfoxides via electrophilic activation or palladium-catalyzed coupling.^9^ During these projects, we conceived the idea that it might be possible to construct a relatively unusual scaffold from *o*-bromophenyl-substituted sulfinate esters **1** by bromide-selective cross-coupling and S–N-bond formation based on our recent achievement in the context of benzothiophene *S*-oxide synthesis^9d^ (Figure 1D). Thus, we attempted the cross-coupling of **1a** with *o*-borylaniline **2a** in the presence of potassium phosphate and a catalytic amount of (amphos)_2_PdCl_2_ at 100 °C (Figure 1E). Although desired biaryl **4** was not generated during this reaction, to our surprise, thiaphenanthridinone **3a** was formed directly without the need for electrophilic activation or deprotection of the Boc group, together with thiaphenanthridinone **5a** via the removal of the Boc group. This result clearly shows that the formation of the C–C and S–N bonds occurred selectively without the previously reported synthesis of a sulfoxide.^9c^

Screening the reaction conditions allowed us to develop an efficient palladium-catalyzed synthesis of thiaphenanthridinone **3a** (Table 1). First, we decreased the temperature, which allowed preparing thiaphenanthridinone **3a** in moderate yield using a catalytic amount of (amphos)_2_PdCl_2_ in the presence of potassium carbonate without removing the Boc group (entry 1). After varying the palladium sources and ligands, we found that an *N*-heterocyclic-carbene (NHC) ligand or electron-rich bulky ligands such as tri(*tert*-butyl)phosphine and di(*tert*-butyl)(1-phenylindol-2-yl)phosphine facilitate the thiaphenanthridinone synthesis from sulfinate ester **1a** and arylboronic acid **2a** (entries 2–9). In particular, **3a** was successfully prepared in high yield when using Pd(dba)_2_ and di(*tert*-butyl)(1-phenylindol-2-yl)phosphine^11^ (**L**) (entry 8). When the reaction temperature of the thiaphenanthridinone synthesis was lowered to room temperature or increased to 100 °C while using Pd(dba)_2_ and **L**, the yield of **3a** decreased significantly (entries 10 and 11). Other bases or reducing the amount of arylboronic acid **2a** also decreased the yield of **3a** (entries 12–15). Ultimately, we succeeded in developing an efficient synthetic route to thiaphenanthridinone **3a** on the gram (5 mmol) scale (entry 16). Thiaphenanthridinone **3a** showed good stability under ambient conditions, i.e., *S*-oxidation of the unexplored scaffold was not observed, not even after 1 year.

**Table 1:**
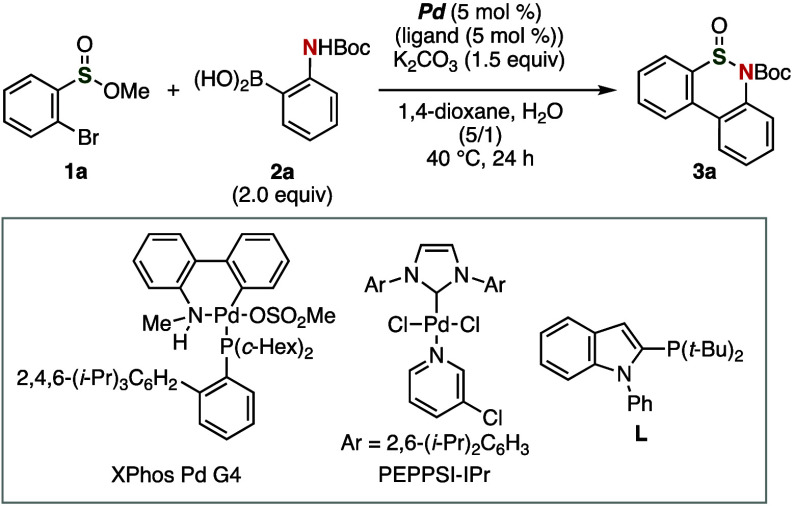
Optimization of the Reaction Conditions

entry	***Pd*** (+ ligand)	base	temp	**3a**/%^a^
1	(amphos)_2_PdCl_2_	K_2_CO_3_	40 °C	58
2	(Ph_3_P)_2_PdCl_2_	K_2_CO_3_	40 °C	34
3	Pd(PPh_3_)_4_	K_2_CO_3_	40 °C	27
4	XPhos Pd G4	K_2_CO_3_	40 °C	37
5	PEPPSI-IPr	K_2_CO_3_	40 °C	76
6	Pd(dba)_2_, dppf	K_2_CO_3_	40 °C	14
7	Pd(dba)_2_, *t*-Bu_3_P·HBF_4_	K_2_CO_3_	40 °C	79
8	Pd(dba)_2_, **L**	K_2_CO_3_	40 °C	89^b^
9	Pd(OAc)_2_, **L**	K_2_CO_3_	40 °C	77
10	Pd(dba)_2_, **L**	K_2_CO_3_	rt	43
11	Pd(dba)_2_, **L**	K_2_CO_3_	100 °C	0
12	Pd(dba)_2_, **L**	K_3_PO_4_	40 °C	52
13	Pd(dba)_2_, **L**	NaHCO_3_	40 °C	0
14	Pd(dba)_2_, **L**	Cs_2_CO_3_	40 °C	68
15^c^	Pd(dba)_2_, **L**	K_2_CO_3_	40 °C	58
16^d^	Pd(dba)_2_, **L**	K_2_CO_3_	40 °C	76^b^

a Yield based on ^1^H NMR analysis using 1,1,2,2-tetrachloroethane as an internal standard.

b Isolated yield.

c The reaction was performed using **2a** (1.1 equiv).

d The reaction was performed on the 5 mmol scale.

With the optimized conditions in hand, we synthesized a wide variety of thiaphenanthridinones **3** from various *o*-bromophenyl-substituted sulfinic acid esters **1** (Figure 2A).^12^ For example, methyl-substituted thiaphenanthridinones **3b** and **3c** were obtained in high yields from methyl 2-bromo-4-methylbenzenesulfinate (**1b**) and methyl 2-bromo-5-methylbenzenesulfinate (**1c**), respectively. When treating methyl 2-bromo-4,6-dimethylbenzenesulfinate (**1d**) with arylboronic acid **2a**, thiaphenanthridinone **3d** was prepared in low yield probably due to the steric hindrance in the cyclization. Electron-rich thiaphenanthridinone **3e** was synthesized in moderate yield along with the recovery of methyl 2-bromo-4-methoxybenzenesulfinate (**1d**) in 25%. Thiaphenanthridinones **3f** or **3g**, which contain a 4-fluorophenyl or 4-chlorophenyl group, were synthesized in good yield, whereby the cyclic sulfinimide moiety as well as the Boc, fluoro, and chloro groups remained intact. Moreover, chloro-, ethoxycarbonyl-, and trifluoromethyl-substituted thiaphenanthridinones **3h**–**3j** were obtained successfully. Treatment of benzyl-substituted sulfinic acid ester **6** with *o*-borylaniline **2a** in the presence of potassium carbonate and a catalytic amount of Pd(dba)_2_ and tri(*tert*-butyl)phosphonium tetrafluoroborate afforded cyclic sulfinimide **7**, which bears a seven-membered-ring, via selective cross-coupling and S–N-bond formation in moderate yield (Figure 2B). Cyclic sulfinimide **7** was synthesized in 26% when using Pd(dba)_2_ and **L**.

**Figure 2:**
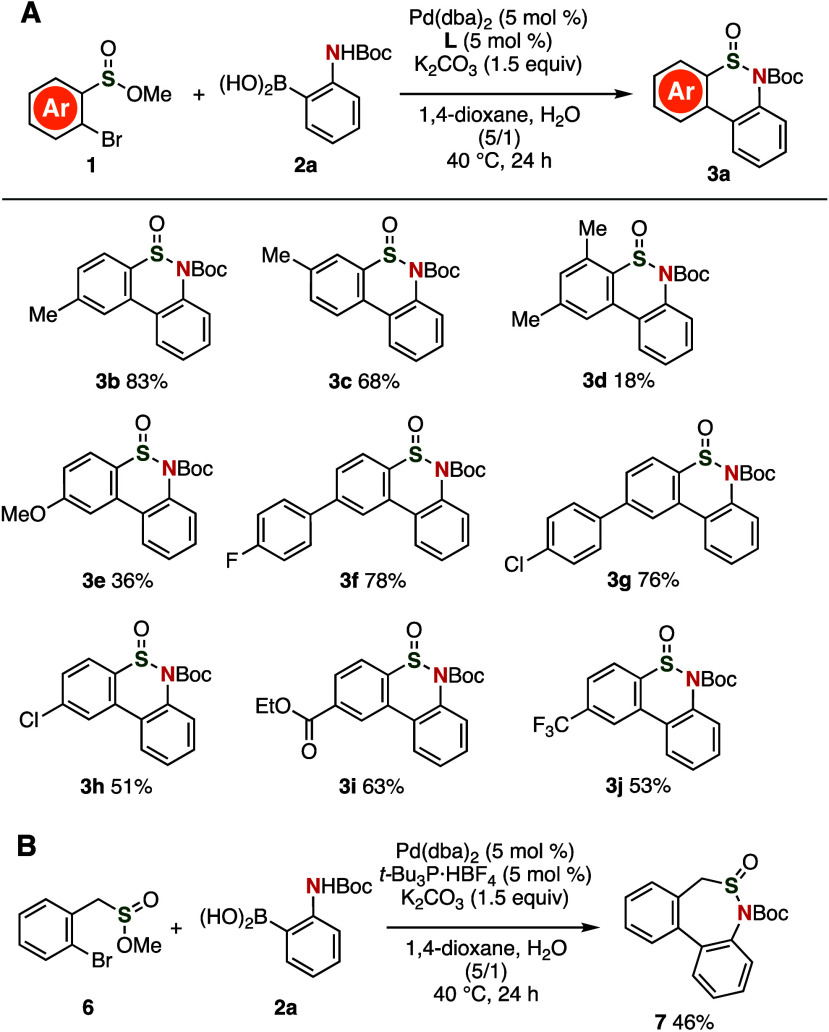
(A) Thiaphenanthridinone synthesis using a variety of sulfinate esters **1**; for the chemical structures of **1**, see the Supporting Information. The reaction was performed using **1**, **2a** (2.0 equiv), Pd(dba)_2_ (5 mol %), **L** (5 mol %), and K_2_CO_3_ (1.5 equiv) in 1,4-dioxane and H_2_O (v/v = 5/1) at 40 °C for 24 h. (B) Synthesis of thiaphenanthridinone analog **7**.

A broad range of thiaphenanthridinones were prepared from various *o*-borylanilines (Figure 3). For instance, methyl- or methoxy-substituted thiaphenanthridinones **3k** and **3l** were efficiently synthesized from electron-rich 4-methyl- and 4-methoxy-substituted 2-borylaniline, respectively. Treatment of 2-boryl-*N*-(*tert*-butoxycarbonyl)-4-(4-tolyl)-aniline with methyl 2-bromobenzenesulfinate (**1a**) under the optimized palladium-catalyzed conditions afforded arylated thiaphenanthridinone **3m** in good yield. It is worth noting here that diverse thiaphenanthridinones **3n**–**3q** that bear electron-withdrawing groups were successfully synthesized in moderate to good yields without affecting the integrity of the cyclic sulfinamide, Boc, chloro, fluoro, ethoxycarbonyl, and trifluoromethyl moieties. Thiophene-fused analog **3r** was synthesized using 2-(*tert*-butoxycarbonylamino)thiophene-3-yl-boronic acid pinacol ester, while the preparation of **3s** through the formation of a tetracyclic fused scaffold was achieved using the corresponding *o*-borylaniline via the regioselective deprotonation of 5-(Boc-amino)benzo[*d*][1,3]dioxol. Furthermore, we accomplished the synthesis of thiaphenanthridinone **3t** from *N*-benzyl-2-aminophenylboronic acid in good yield, while *N*-methyl-substituted analog **3u** was successfully synthesized by modifying the reaction conditions.

**Figure 3:**
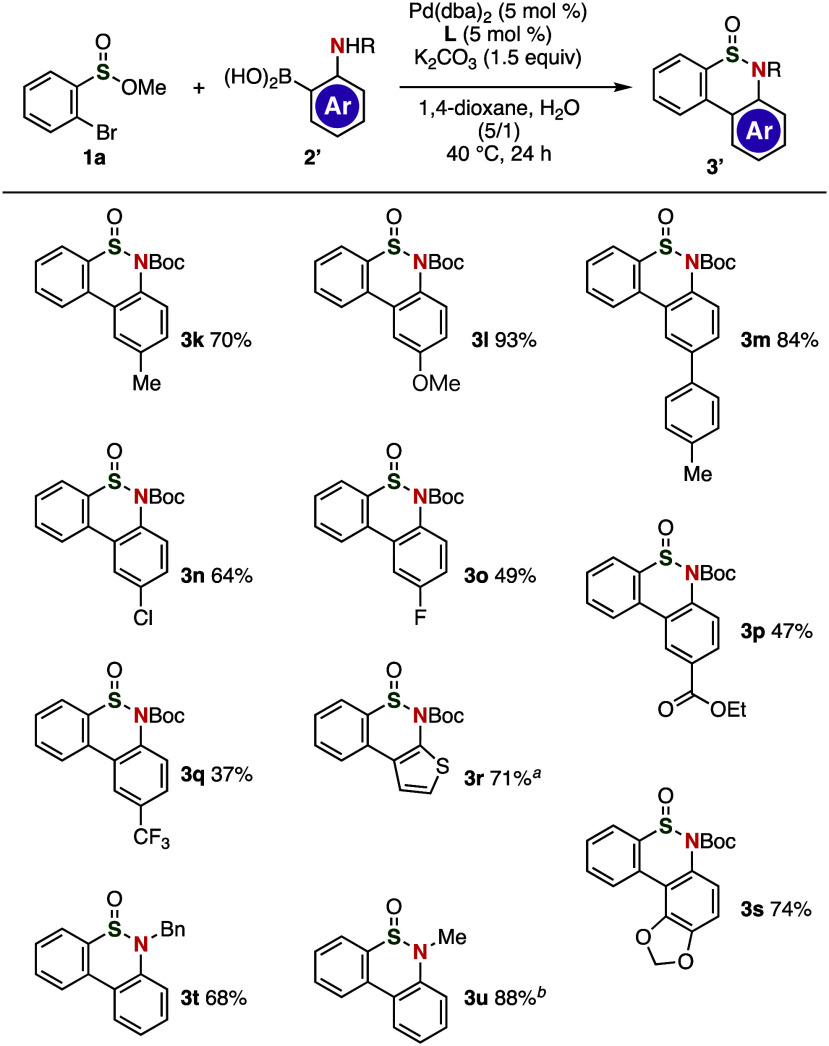
Synthesis of thiaphenanthridinones **3k**–**3u** from a variety of *o*-borylanilines **2**; for the chemical structures of **2**, see the Supporting Information. The reaction was performed using **1**, **2a** (2.0 equiv), Pd(dba)_2_ (5 mol %), **L** (5 mol %), and K_2_CO_3_ (1.5 equiv) in 1,4-dioxane and H_2_O (v/v = 5/1) at 40 °C for 24 h. ^*a*^The reaction was performed using (*t*-Bu)_3_P·HBF_4_ as a ligand. ^*b*^The reaction was performed using Pd(PPh_3_)_4_ (5 mol %) and K_2_CO_3_ (2.0 equiv) in 1,2-dimethoxyethane and water (v/v = 5/1) at 100 °C.

We conducted several control experiments in order to gain insight into the reaction mechanism (Figure 4). When the reaction time was reduced from 24 h to 3 h or 30 min, biaryl **4** was obtained (Figure 4A). The cyclization of biaryl **4** took place efficiently in the presence of potassium carbonate (Figure 4B). It is worth noting that the cyclization did not require strong bases in spite of the weak nucleophilicity of the Boc group.^13^ Based on these control experiments, we propose reaction mechanism shown in Figure 4C. First, the oxidative addition of aryl bromide **1a** to palladium(0) would lead to arylpalladium intermediate **I**. Then, biaryl **4** could be synthesized by a ligand exchange and reductive elimination prior to the formation of the S–N bond. Subsequently, a base-promoted sulfinylation of the amino group could proceed in an intramolecular fashion to provide thiaphenanthridinone **3a**.

**Figure 4:**
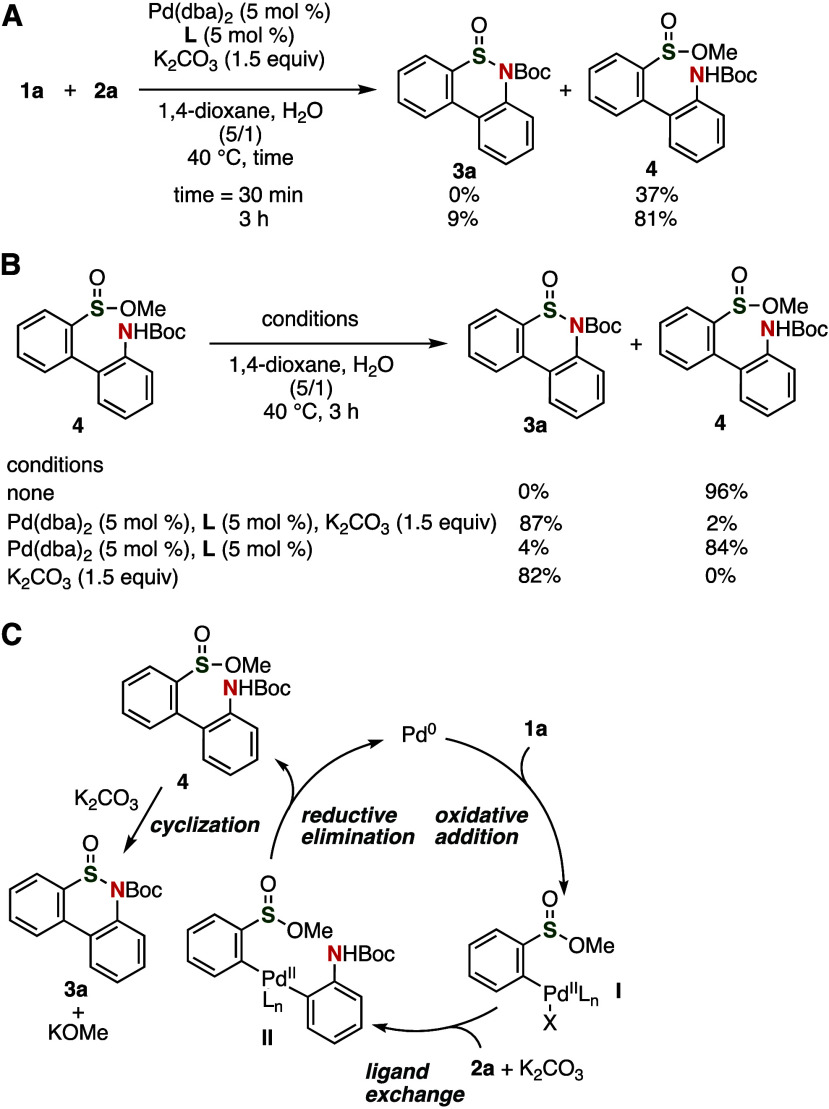
Control experiments - (A) reducing the reaction time from 24 to 3 h or 30 min; (B) modifying the conditions for the cyclization. (C) A plausible reaction mechanism.

Various transformations of the thus obtained thiaphenanthridinones **3** led to a variety of thiaphenanthridinone derivatives (Figure 5). For example, several NH-thiaphenanthridinones **5a**–**5c** were synthesized in excellent yields by removal of the Boc group using trifluoroacetic acid (TFA) (Figure 5A). We found that thiaphenanthridinone **5a** is stable under atmospheric conditions for more than 1 year, and decomposition, e.g., in the form of *S*-oxidation was not observed. Oxidation of **3a** with *m*CPBA efficiently afforded dibenzosultam **8**, wherein the structural integrity of the Boc moiety and of the aromatic rings remained intact (Figure 5B, top).^2a^ Sulfenamide analog **9** was prepared by the reduction of **3a** with potassium iodide in the presence of trifluoroacetic anhydride (Figure 5B, bottom).^14^

**Figure 5:**
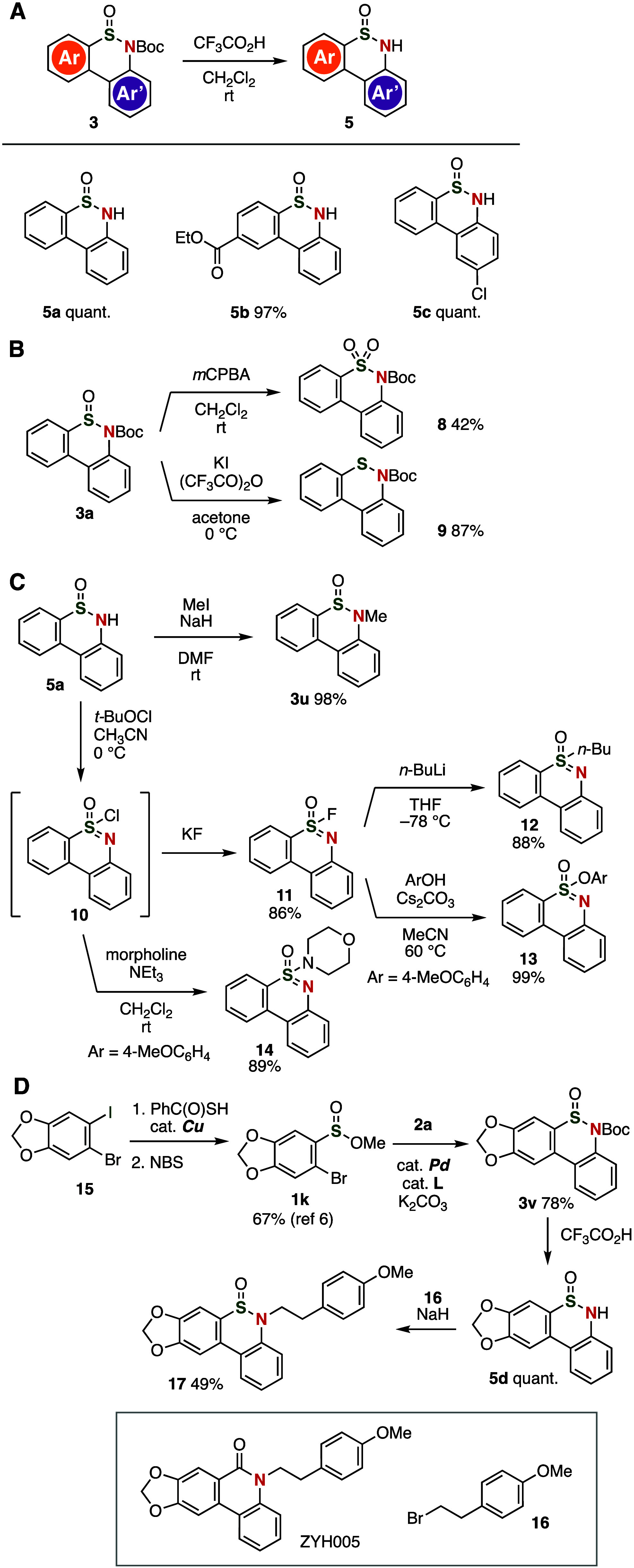
(A) Deprotection of thiaphenanthridinones **3**. (B) Oxidation and reduction of **3a**. (C) Transformations of **5a**. (D) Synthesis of **17**; for details, see the Supporting Information.

Further bond formations in NH-thiaphenanthridinone **5a**, such as *N*-alkylation or *S*-oxidation, followed by nucleophilic substitutions, allowed us to prepare a wide variety of thiaphenanthridinone derivatives (Figure 5C). Smooth *N*-methylation of **5a** proceeded to provide thiaphenanthridinone **3u** in high yield. Sulfonimidoyl chloride **10** was prepared by the oxidation of **5a** with *t*-BuOCl.^15^ Moreover, sulfonimidoyl fluoride **11** was obtained from the addition of potassium fluoride. This process has already been used successfully in sulfur-fluoride-exchange (SuFEx) reactions^16^ with *n*-butyllithium^15^ or 4-methoxyphenol.^17^ The preparation of sulfonimidoyl amide **14** was accomplished by the amination of sulfonimidoyl chloride **10**.^10e^ Given that a wide range of thiaphenanthridinone derivatives containing an sp^3^-hybridized sulfur center can be prepared by simple transformations of these thiaphenanthridinones, this novel chemical library based on the synthesis of thiaphenanthridinones can be expected to attract significant attention in the context of drug discovery.

Compound **17**, an analogue of a bioactive species, was easily synthesized from thiaphenanthridinone **3v** (Figure 5D). A palladium-catalyzed cross-coupling followed by cyclization occurred smoothly from sulfinate ester **1k**, which was prepared from the corresponding aryl iodide **15** by copper-catalyzed thiolation and subsequent *S*-oxidation in methanol. Then, the Boc group in the resulting thiaphenanthridinone **3v** was deprotected with TFA, followed by *N*-alkylation with bromoalkane **16** in the presence of sodium hydride to furnish ZYH005^4d^ analog **17**. Considering that a number of phenanthridinones have been found to exhibit desirable biological activity,^4^ their sulfur analogs promise significant potential as prospective bioactive compounds, which can be expected to find applications in a broad variety of research fields including pharmaceutical sciences, agrochemistry, and chemical biology.

In summary, we have developed a facile one-step synthesis of thiaphenanthridinones from sulfinate esters and 2-borylanilines. The palladium-catalyzed cross-coupling followed by cyclization proceeds under mild conditions, which allows the synthesis of a wide variety of sulfur analogs of phenanthridinones that contain a broad spectrum of functional groups from readily available starting materials. These results are significant since the catalytic transformation was accomplished although S(IV) compounds can poison transition metal catalysts.^18,19^ Further studies, including detailed mechanistic studies based on theoretical calculations, an in-depth examination of structure–activity relationships, and developing synthetic routes to further bioactive compounds, are currently in progress in our research group.

## Data Availability

The data underlying this study are available in the published article and its Supporting Information.

## Supporting Information Available

Experimental procedures, characterization for new compounds including NMR spectra (PDF)
