# Supplementary material for: Synthesis of Thiaphenanthridinones from Sulfinate Esters and 2-Borylanilines
Source: Org Lett. 2024 Nov 6;26(45):9676–81. doi: 10.1021/acs.orglett.4c03420 (PMC11574856; doi:10.1021/acs.orglett.4c03420)
Supplement: Supplementary file 1 — ol4c03420_si_001.pdf [file ol4c03420_si_001.pdf]

Supporting Information

Synthesis of Thiaphenanthridinones from Sulfinate Esters and 2-Borylanilines

Keisuke Nakamura,[†] Minori Suzuki,[†,‡] and Suguru Yoshida*[†]

[†]*Department of Biological Science and Technology, Faculty of Advanced Engineering, Tokyo University of Science, 6-3-1 Niijuku, Katsushika-ku, Tokyo, 125-8585, Japan*
[‡]*Laboratory of Chemical Bioscience, Institute of Biomaterials and Bioengineering, Tokyo Medical and Dental University (TMDU), 2-3-10 Kanda-Surugadai, Chiyoda-ku, Tokyo 101-0062, Japan*

Contents

General Information S2
Structures of Sulfinate Esters 1 and Organoborons 2 S3
Experimental Procedures S4
Characterization Data of New Compounds S12
References for Supporting Information S25
^{1}H and ^{13}C NMR Spectra of New Compounds S26

General Information

All reactions were performed with dry glassware under atmosphere of argon, unless otherwise noted. Analytical thin-layer chromatography (TLC) was performed on precoated (0.25 mm) silica-gel plates (Merck Chemicals, Silica Gel 60 F254, Cat. No. 1.05715). Column chromatography was conducted using silica-gel (Kanto Chemical Co., Inc., Silica Gel 60N, spherical neutral, particle size 40–50 μm, Cat. No. 37562-85 or particle size 63–210 μm, Cat. No. 37565-85). Preparative TLC (PTLC) was performed on silica gel (Wako Pure Chemical Industries Ltd., Wakogel B-5F, Cat. No. 230-00043). Melting points (Mp) were measured on an OptiMelt MPA100 (Stanford Research Systems), and are uncorrected. ^{1}H NMR spectra were obtained with a Bruker AVANCE 400 spectrometer at 400 MHz. ^{13}C NMR spectra were obtained with a Bruker AVANCE 400 spectrometer at 101 MHz. ^{19}F NMR spectra were obtained with a Bruker AVANCE 400 spectrometer at 376 MHz. ^{11}B NMR spectra were obtained with a Bruker AVANCE 400 spectrometer at 128 MHz. All NMR measurements were carried out at 25 ºC. $CDCl_3$, CD_3OD, or DMSO-d_6 was used as a solvent for obtaining NMR spectra. Chemical shifts (δ) are given in parts per million (ppm) downfield from the solvent peak (δ 7.26 for ^{1}H NMR in $CDCl_3$, δ 77.0 for ^{13}C NMR in $CDCl_3$; δ 3.31 for ^{1}H NMR in CD_3OD, δ 49.0 for ^{13}C NMR in CD_3OD) as an internal reference, or α,α,α-trifluorotoluene (δ −63.0 ppm for ^{19}F NMR in $CDCl_3$) or $BF_3 \cdot OEt_2$ (δ 0.0 ppm for ^{11}B NMR in $CDCl_3$) as external standards with coupling constants (*J*) in hertz (Hz). The abbreviations s, d, t, q, and m signify singlet, doublet, triplet, quartet, and multiplet, respectively. IR spectra were measured on a Shimadzu IRSpirit spectrometer with the absorption band given in cm^{-1}. High-resolution mass spectra (HRMS) were measured on a JEOL JMS-T100CS "AccuTOF CS" mass spectrometer under positive electrospray ionization (ESI^+) conditions or negative electrospray ionization (ESI^-) conditions, or JMS-700 (JEOL, Tokyo, Japan) mass spectrometer under electron impact ionization (EI) conditions.

Unless otherwise noted, materials obtained from commercial suppliers were used without further purification. Methyl 2-bromobenzenesulfinate (**1a**),[S1] methyl 2-bromo-4-methylbenzenesulfinate (**1b**),[S1] methyl 2-bromo-5-methylbenzenesulfinate (**1c**),[S1] methyl 2-bromo-4,6-dimethylbenzenesulfinate (**1d**),[S1] methyl 2-bromo-4-chlorobenzenesulfinate (**1h**),[S1] ethyl 3-bromo-4-(methoxysulfinyl)benzoate (**1i**),[S2] methyl 2-bromo-4-(trifluoromethyl)benzenesulfinate (**1j**),[S1] methyl 6-bromobenzo[*d*][1,3]dioxole-5-sulfinate (**1k**),[S1] (2-((*tert*-butoxycarbonyl)amino)phenyl)boronic acid (**2a**),[S3] (2-((*tert*-butoxycarbonyl)amino)-5-methylphenyl)boronic acid (**2b**),[S3] (2-((*tert*-butoxycarbonyl)amino)-5-methoxyphenyl)boronic acid (**2c**),[S3] (2-((*tert*-butoxycarbonyl)amino)-5-chlorophenyl)boronic acid (**2e**),[S3] (2-((*tert*-butoxycarbonyl)amino)-5-fluorophenyl)boronic acid (**2f**),[S3] (2-((*tert*-butoxycarbonyl)amino)-5-(trifluoromethyl)phenyl)boronic acid (**2h**),[S3] *tert*-butyl (3-(4,4,5,5-tetramethyl-1,3,2-dioxaborolan-2-yl)thiophen-2-yl)carbamate (**2i**),[S4] *N*-benzyl-2-(4,4,5,5-tetramethyl-1,3,2-dioxaborolan-2-yl)aniline (**2k**),[S5] *N*-methyl-2-(4,4,5,5-tetramethyl-1,3,2-dioxaborolan-2-yl)aniline (**2l**),[S6] and 1-(2-bromoethyl)-4-methoxybenzene (**16**)[S7] were prepared according to the reported methods.

Structures of Sulfinate Esters 1 and Organoborons 2

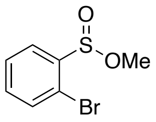
1a

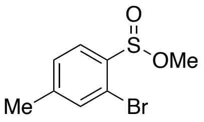
1b

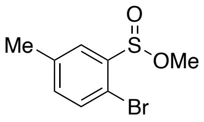
1c

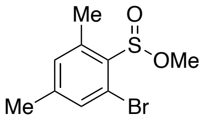
1d

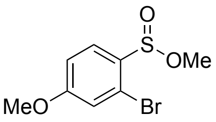
1e

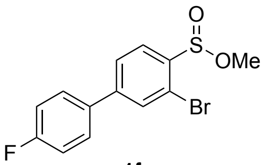
1f

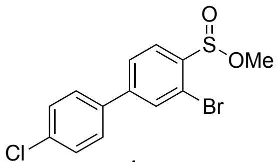
1g

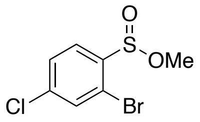
1h

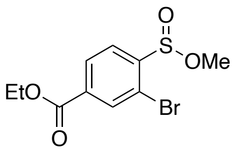
1i

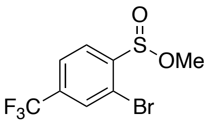
1j

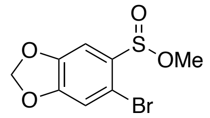
1k

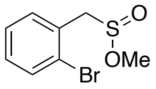
6

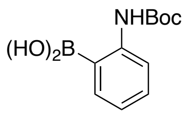
2a

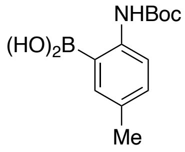
2b

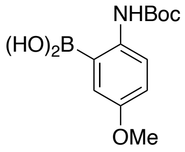
2c

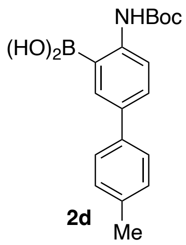
2d

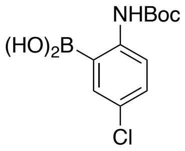
2e

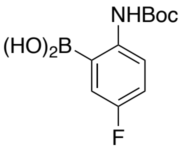
2f

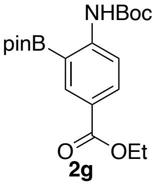
2g

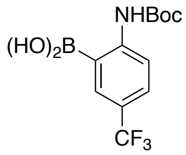
2h

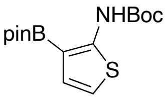
2i

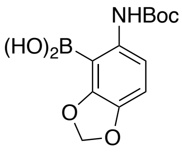
2j

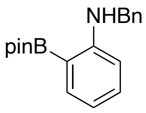
2k

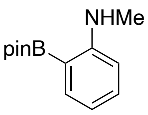
2l

Experimental Procedures

A typical procedure for the synthesis of 6-thiaphenanthridinones

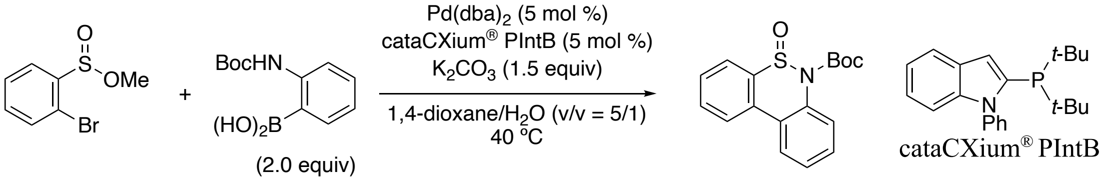

In a 5 mL screw-top V-vial® with a solid-top cap (Sigma-Aldrich, Cat. No. Z115118), to a mixture of 2-((*tert*-butoxycarbonyl)amino)phenyl)boronic acid (**2a**) (37.3 mg, 0.157 mmol, 2.0 equiv), Pd(dba)$_2$ (2.3 mg, 4.0 μmol, 5 mol %), cataCXium® PIntB (1.3 mg, 4.0 μmol, 5 mol %), and potassium carbonate (17.3 mg, 0.125 mmol, 1.5 equiv) were added a solution of methyl *o*-bromobenzenesulfinate (**1a**) (18.3 mg, 77.8 μmol, 1.0 equiv) in 1,4-dioxane (1.3 mL) and H_2O (260 μL) at room temperature. After stirring at 40 °C (aluminium heating block; block temp.: 40 °C) for 24 h, the mixture was cooled to room temperature. The mixture was filtered through Na_2SO_4 and washed with EtOAc (20 mL). The filtrate was concentrated under reduced pressure. The residue was purified by preparative TLC (*n*-hexane/EtOAc = 5/1) to give 5-(*tert*-butoxycarbonyl)-6-thiaphenanthridin-6(5*H*)-one (**3a**) (21.7 mg, 68.9 μmol, 89%) as a pale brown solid.

According to the procedure for preparing **3a**, 6-thiaphenanthridinones **3b–3q**, and **3s** were prepared from the corresponding sulfinate esters and arylboronic acids.

*Gram-scale synthesis of 6-thiaphenanthridinones (**3a**)*

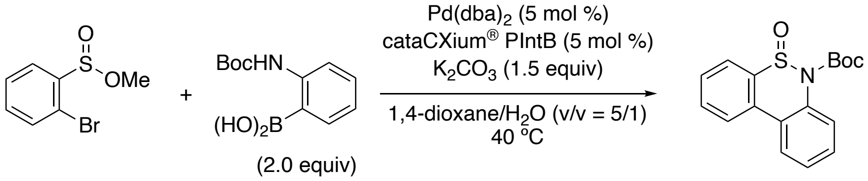

To a mixture of methyl *o*-bromobenzenesulfinate (**1a**) (1.18 g, 5.00 mmol, 1.0 equiv), 2-((*tert*-butoxycarbonyl)amino)phenyl)boronic acid (**2a**) (2.37 g, 9.99 mmol, 2.0 equiv), Pd(dba)$_2$ (144 mg, 0.250 mmol, 5 mol %), cataCXium® PIntB (84.4 mg, 0.250 mmol, 5 mol %), and potassium carbonate (1.04 g, 7.50 mmol, 1.5 equiv) were added 1,4-dioxane (83 mL) and H_2O (17 mL) at room temperature. After stirring at 40 °C (oil bath; bath temp.: 40 °C) for 24 h, the mixture was cooled to room temperature, the mixture was added water (20 mL). The mixture was extracted with EtOAc (20 mL × 3). The combined organic layer was washed with brine (20 mL) and dried with Na_2SO_4. After filtration, the filtrate was concentrated under reduced pressure. The residue was purified by column chromatography (*n*-hexane/EtOAc = 3/1) to give 5-(*tert*-butoxycarbonyl)-6-thiaphenanthridin-6(5*H*)-one (**3a**) (1.33 g, 3.80 mmol, 76%) as a brown solid.

Synthesis of tert-butyl dibenzo[c,e][1,2]thiazepine-5(7H)-carboxylate 6-oxide (7)

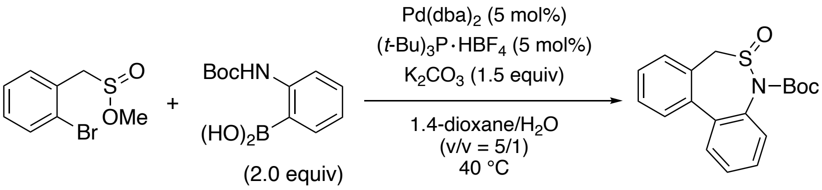

To a mixture of methyl (2-bromophenyl)methanesulfinate (**6**) (19.7 mg, 79.1 μmol, 1.0 equiv), 2-((*tert*-butoxycarbonyl)amino)phenyl)boronic acid (**2a**) (38.0 mg, 0.160 mmol, 2.0 equiv), Pd(dba)$_2$ (2.3 mg, 4,0 μmol, 5 mol %), tri-*tert*-butylphosphonium tetrafluoroborate (1.2 mg, 4.1 μmol, 5 mol %), and potassium carbonate (16.8 mg, 0.122 mmol, 1.5 equiv) were added 1,4-dioxane (1.3 mL) and water (267 μL) at room temperature. After stirring at 40 °C (aluminium heating block; block temp.: 40 °C) for 24 h, the mixture was cooled to room temperature. The mixture was filtered through Na_2SO_4 and washed with EtOAc (20 mL). The filtrate was concentrated under reduced pressure. The residue was purified by preparative TLC (*n*-hexane/EtOAc = 3/1) to give *tert*-butyl dibenzo[*c,e*][1,2]thiazepine-5(7*H*)-carboxylate 6-oxide (**7**) (12.1 mg, 36.7 μmol, 46%) as a pale yellow solid.

*Synthesis of tert-butyl 4H-benzo[e]thieno[2,3-c][1,2]thiazine-4-carboxylate 5-oxide (**3r**)*

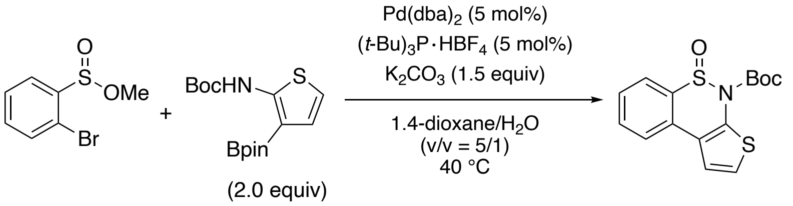

In a 5 mL screw-top V-vial® with a solid-top cap (Sigma–Aldrich, Cat. No. Z115118), to a mixture of methyl *o*-bromobenzenesulfinate (**1a**) (18.9 mg, 80.4 µmol, 1.0 equiv), *tert*-butyl (3-(4,4,5,5-tetramethyl-1,3,2-dioxaborolan-2-yl)thiophen-2-yl)carbamate (**2i**) (52.0 mg, 0.160 mmol, 2.0 equiv), $Pd(dba)_2$ (2.4 mg, 4.2 µmol, 5 mol %), tri-*tert*-butylphosphonium tetrafluoroborate (1.2 mg, 4.1 µmol, 5 mol %), and potassium carbonate (16.6 mg, 12.0 µmol, 1.5 equiv) were added 1,4-dioxane (1.3 mL) and H_2O (267 µL) at room temperature. After stirring at 40 °C (aluminium heating block; block temp.: 40 °C) for 24 h, the mixture was cooled to room temperature. The mixture was filtered through Na_2SO_4 and washed with EtOAc (20 mL). The filtrate was concentrated under reduced pressure. The residue was purified by preparative TLC (*n*-hexane/EtOAc = 3/1) to give *tert*-butyl 4*H*-benzo[*e*]thieno[2,3-*c*][1,2]thiazine-4-carboxylate 5-oxide (**3r**) (18.3 mg, 56.9 µmol, 71%) as a purple solid.

*Synthesis of 5-methyl-6-thiaphenanthridin-6(5H)-one (**3u**)*

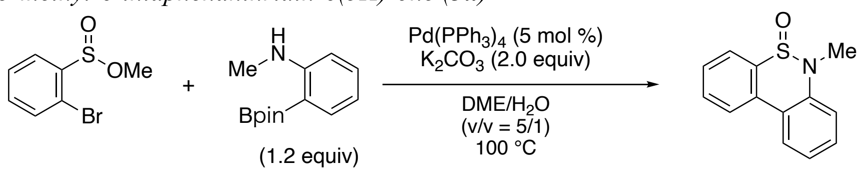

To a mixture of methyl *o*-bromobenzenesulfinate (**1a**) (18.7 mg, 79.5 µmol, 1.0 equiv), *N*-methyl-2-(4,4,5,5-tetramethyl-1,3,2-dioxaborolan-2-yl)aniline (**2l**) (22.2 mg, 95.2 µmol, 1.2 equiv), $Pd(PPh_3)_4$ (4.6 mg, 3.98 µmol, 5 mol %), and potassium carbonate (22.4 mg, 0.162 mmol, 2.0 equiv) were added 1,2-dimethoxyethane (1.3 mL) and H_2O (267 µL) at room temperature. After stirring at 100 °C (aluminium heating block; block temp.: 100 °C) for 24 h, the mixture was cooled to room temperature, the mixture was filtered through Na_2SO_4 and washed with EtOAc (20 mL). The filtrate was concentrated under reduced pressure. The residue was purified by preparative TLC (*n*-hexane/EtOAc = 1/1) to give 5-methyl-6-thiaphenanthridin-6(5*H*)-one (**3u**) (16.1 mg, 70.2 µmol, 88%) as a colorless oil.

A typical procedure of control experiments for the synthesis of 5-(tert-butoxycarbonyl)-6-thiaphenanthridin-6(5H)-one

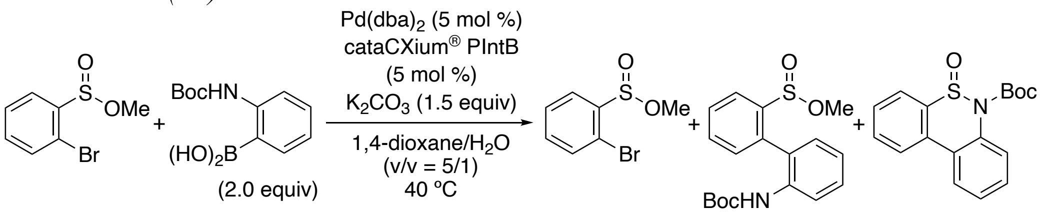

In a 5 mL screw-top V-vial® with a solid-top cap (Sigma-Aldrich, Cat. No. Z115118), to a mixture of 2-((*tert*-butoxycarbonyl)amino)phenyl)boronic acid (**2a**) (38.0 mg, 0.160 mmol, 2.0 equiv), $Pd(dba)_2$ (2.3 mg, 4.0 µmol, 5 mol %), cataCXium® PIntB (1.3 mg, 4.0 µmol, 5 mol %), and potassium carbonate (16.6 mg, 0.12 mmol, 1.5 equiv) was added a solution of methyl *o*-bromobenzenesulfinate (**1a**) (18.8 mg, 80.0 µmol, 1.0 equiv) in 1,4-dioxane (1.3 mL) and H_2O (260 µL) at room temperature. After stirring the mixture at 40 °C (aluminium heating block; block temp.: 40 °C) for 30 min, the mixture was cooled to room temperature. The mixture was filtered through Na_2SO_4 and washed with EtOAc (20 mL). The filtrate was concentrated under reduced pressure. The residue was purified by preparative TLC (*n*-hexane/EtOAc = 3/1) to give methyl 2′-((*tert*-butoxycarbonyl)amino)-[1,1′-biphenyl]-2-sulfinate (**4**) (10.0 mg, 28.8 µmol, 37%) as a brown oil.

*Synthesis of 5-(tert-butoxycarbonyl)-6-thiaphenanthridin-6(5H)-one (**3a**) from sulfinate ester **4***

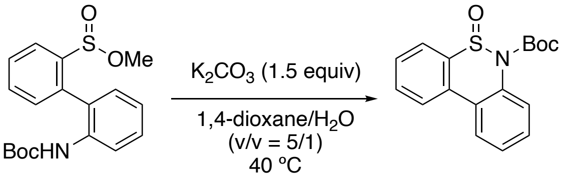

In a 5 mL screw-top V-vial® with a solid-top cap (Sigma-Aldrich, Cat. No. Z115118), to a mixture of potassium carbonate (8.3 mg, 60 µmol, 1.5 equiv) and 2′-((*tert*-butoxycarbonyl)amino)-[1,1′-biphenyl]-2-sulfinate (**4**) (13.9 mg, 40.0 µmol, 1.0 equiv) were added 1,4-dioxane (650 µL) and H_2O (130 µL) at room temperature. After stirring the mixture at 40 °C (aluminium heating block; block temp.: 40 °C) for 3 h, the mixture was cooled to room temperature. The mixture was filtered through Na_2SO_4 and washed with EtOAc (20 mL). The filtrate was concentrated under reduced pressure. The residue was purified by preparative TLC (*n*-hexane/EtOAc = 3/1) to give 5-(*tert*-butoxycarbonyl)-6-thiaphenanthridin-6(5*H*)-one (**3a**) (10.3 mg, 32.6 µmol, 82%) as a brown solid.

*A typical procedure for the synthesis of 6-thiaphenanthridinones **5***

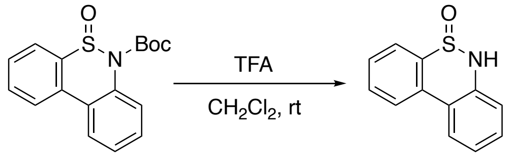

In a 5 mL screw-top V-vial® with a solid-top cap (Sigma-Aldrich, Cat. No. Z115118), to a solution of 5-(*tert*-butoxycarbonyl)-6-thiaphenanthridin-6(5*H*)-one (**3a**) (132 mg, 0.417 mmol, 1.0 equiv) in CH_2Cl_2 (6.4 mL) was added trifluoroacetic acid (TFA) (1.6 mL) at room temperature. After stirring the mixture for 1 h at the same temperature, to the mixture was added an aqueous saturated sodium hydrogen carbonate (1 mL + 9 mL). The mixture was extracted with CH_2Cl_2 (20 mL × 3). The combined organic layer was washed with brine (20 mL) and dried with Na_2SO_4. After filtration, the filtrate was concentrated under reduced pressure to give 6-thiaphenanthridin-6(5*H*)-one (**5a**) (90.8 mg, 0.421 mmol, quant) as a brown solid.

*Oxidation of 5-(tert-butoxycarbonyl)-6-thiaphenanthridin-6(5H)-one (**3a**)*

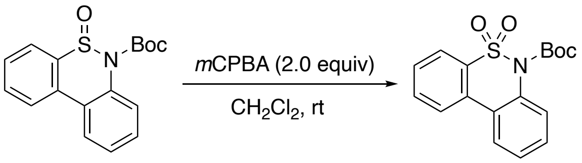

In a 5 mL screw-top V-vial® with a solid-top cap (Sigma-Aldrich, Cat. No. Z115118), to a solution of 5-(*tert*-butoxycarbonyl)-6-thiaphenanthridin-6(5*H*)-one (**3a**) (15.6 mg, 49.7 µmol, 1.0 equiv) in CH_2Cl_2 (500 µL) was added *m*CPBA (24.7 mg, 70%, 0.100 mmol, 2.0 equiv) at room temperature. The mixture was stirred at room temperature for 12 h at the same temperature. Then, to the mixture was added aqueous saturated sodium hydrogen carbonate (1 mL + 9 mL). The mixture was extracted with CH_2Cl_2 (10 mL × 3). The combined organic layer was washed with brine (10 mL) and dried with Na_2SO_4. After filtration, the filtrate was concentrated under reduced pressure. The residue was purified by preparative TLC (*n*-hexane/ EtOAc = 4/1) to give *tert*-butyl 6*H*-dibenzo[*c,e*][1,2]thiazine-6-carboxylate 5,5-dioxide (**8**) (6.9 mg, 21 µmol, 42%) as a colorless solid.

*Synthesis of tert-butyl 6H-dibenzo[c,e][1,2]thiazine-6-carboxylate (**9**)*

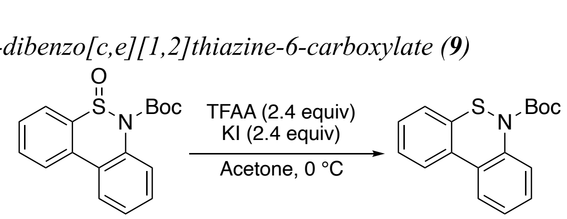

In a 5 mL screw-top V-vial® with a solid-top cap (Sigma–Aldrich, Cat. No. Z115118), to a mixture of 5-(*tert*-butoxycarbonyl)-6-thiaphenanthridin-6(5*H*)-one (**5a**) (126 mg, 0.400 mmol, 1.0 equiv) and sodium iodide (143.9 mg, 0.960 mmol, 2.4 equiv) in acetone (2.0 mL) was added trifluoroacetic anhydride (TFAA)

(136 μL, 0.980 mmol, 2.4 equiv) at 0 ºC. After stirring the mixture for 1 h at the same temperature, to the mixture were added an aqueous saturated sodium bicarbonate (10 mL) and an aqueous saturated sodium thiosulfate (10 mL). The mixture was extracted with dichloromethane (10 mL × 3). The combined organic extract was washed with brine (10 mL) and dried with Na_2SO_4. After filtration, the filtrate was concentrated under reduced pressure. The residue was purified by column chromatography (*n*-hexane/dichloromethane = 1/1) to give *tert*-butyl6*H*-dibenzo[*c,e*][1,2]thiazine-6-carboxylate (**9**) (104 mg, 0.347 mmol, 87%) as a colorless solid.

*A typical procedure for the alkylation of 6-thiaphenanthridin-6(5H)-one (**5a**)*

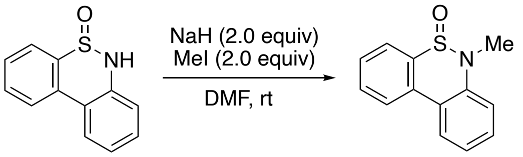

Sodium hydride (60%, dispersion in paraffin liquid) (2.4 mg, 60 μmol, 2.0 equiv) was added to 6-thiaphenanthridin-6(5*H*)-one (**5a**) (7.0 mg, 33 μmol, 1.0 equiv) in dry DMF (200 μL) at 0 ºC. After stirring the mixture for 15 min at the same temperature, iodomethane (3.7 μL, 60 μmol, 2.0 equiv) was added to the mixture in a single portion. The mixture was stirred at room temperature for 12 h. Then, the mixture was added to ice water (5 mL). The mixture was extracted with CH_2Cl_2 (10 mL × 3). The combined mixture was washed with brine (10 mL) and dried with Na_2SO_4. After filtration, the filtrate was concentrated under reduced pressure. The residue was purified by preparative TLC (*n*-hexane/EtOAc = 1/1) to give 5-methyl-6-thiaphenanthridin-6(5*H*)-one (**10**) (7.3 mg, 32 μmol, 98%) as a colorless oil.

*A procedure for the synthesis of 5-fluoro-5λ^4-dibenzo[c,e][1,2]thiazine 5-oxide (**11**)*

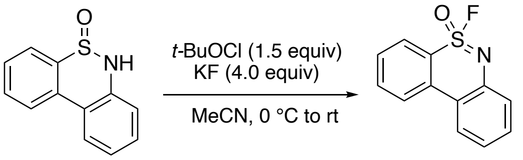

To a mixture of 6-thiaphenanthridin-6(5*H*)-one (**5a**) (215 mg, 1.00 mmol, 1.0 equiv) in acetonitrile (5.5 mL) was added *tert*-butyl hypochlorite (170 μL, 1.50 mmol, 1.5 equiv) at 0 ºC. After stirring the mixture for 10 min at the same temperature, potassium fluoride (232 mg, 4.00 mmol, 4.0 equiv) was added to the mixture in a single portion. After stirring the mixture at room temperature for 16 h, the solution was filtered through a short pad of celite and washed with EtOAc (20 mL). The filtrate was concentrated under reduced pressure. The residue was purified by column chromatography (*n*-hexane/ EtOAc = 4/1) to give 5-fluoro-5λ^4-dibenzo[*c*,*e*][1,2]thiazine 5-oxide (**11**) (201 mg, 0.863 mmol, 86%) as a pale yellow solid.

*Synthesis of 5-butyl-5λ^4-dibenzo[c,e][1,2]thiazine 5-oxide (**12**)*

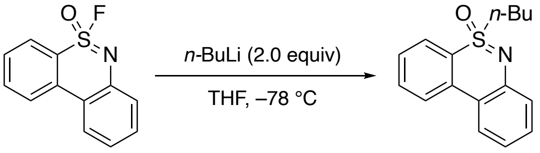

To a mixture of 5-fluoro-5λ^4-dibenzo[*c*,*e*][1,2]thiazine 5-oxide (**11**) (12.0 mg, 51.4 μmol, 1.0 equiv) in THF (250 μL) was slowly added *n*-BuLi (2.66 M in hexane, 37.6 μL, 0,100 mmol, 2.0 equiv) at –78 ºC. After stirring the mixture for 1 h at the same temperature, to the mixture was added an aqueous 1 M HCl (10 mL). The mixture was extracted with CH_2Cl_2 (5 mL × 3). The combined organic layer was washed with brine (10 mL) and dried with Na_2SO_4. After filtration, the filtrate was concentrated under reduced pressure. The residue was purified by preparative TLC (*n*-hexane/EtOAc = 3/1) to give 5-butyl-5λ^4-dibenzo[*c*,*e*][1,2]thiazine 5-oxide (**12**) (12.3 mg, 45.3 μmol, 88%) as a colorless solid.

*Synthesis of 5-(4-methoxyphenoxy)-5λ⁴-dibenzo[c,e][1,2]thiazine 5-oxide (**13**)*

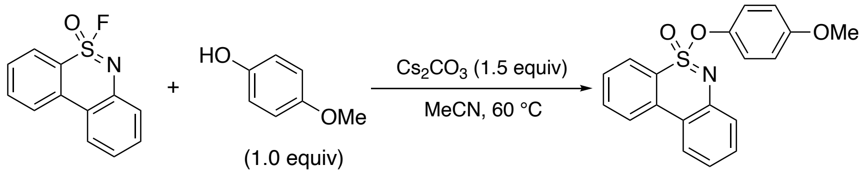

In a 5 mL screw-top V-vial® with a solid-top cap (Sigma–Aldrich, Cat. No. Z115118), to a mixture of 5-fluoro-5λ⁴-dibenzo[*c*,*e*][1,2]thiazine 5-oxide (**11**) (11.7 mg, 50.2 µmol, 1.0 equiv) in acetonitrile (500 µL) were added 4-methoxyphenol (6.4 mg, 52 µmol, 1.0 equiv) and Cs_2CO_3 (24.4 mg, 74.9 µmol, 1.5 equiv) at room temperature. After stirring the mixture at 60 ºC (oil bath; bath temp.: 60 ºC) for 16 h, the mixture was cooled to room temperature. To the mixture was added water (1 mL + 9 mL). The mixture was extracted with EtOAc (5 mL × 3). The combined organic layer was washed with brine (5 mL) and dried with Na_2SO_4. After filtration, the filtrate was concentrated under reduced pressure. The residue was purified by preparative TLC (*n*-hexane/EtOAc = 3/1) to give 5-(4-methoxyphenoxy)-5λ⁴-dibenzo[*c,e*][1,2]thiazine 5-oxide (**13**) (16.8 mg, 49.8 µmol, 99%) as a colorless solid.

*A procedure for the synthesis of 5-morpholinodibenzo[c,e][1,2]thiazine 5-oxide (**14**)*

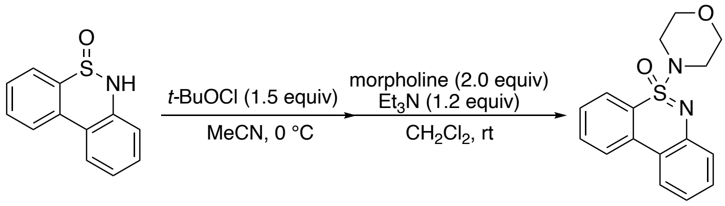

In a 5 mL screw-top V-vial® with a solid-top cap (Sigma–Aldrich, Cat. No. Z115118), to a mixture of 6-thiaphenanthridin-6(5*H*)-one (**5a**) (21.2 mg, 98.5 µmol, 1.0 equiv) in acetonitrile (550 µL) was added *tert*-butyl hypochlorite (17.0 µL, 0.150 mmol, 1.5 equiv) at 0 ºC. After stirring the mixture for 1 h at the same temperature, the mixture was concentrated under reduced pressure. To the residue dissolved in dichloromethane (550 µL) was added triethylamine (16.7 µL, 0.120 mmol, 1.2 equiv) and morpholine (17.3 µL, 0.200 mmol, 2.0 equiv) at room temperature. After stirring the mixture for 16 h at the same temperature, to the mixture was added an aqueous 1 M HCl (5 mL). The mixture was extracted with CH_2Cl_2 (5 mL × 3). The combined organic layer was washed with brine (10 mL) and dried with Na_2SO_4. After filtration, the filtrate was concentrated under reduced pressure. The residue was purified by preparative TLC (*n*-hexane/ EtOAc = 2/1) to give 5-morpholinodibenzo[*c,e*][1,2]thiazine 5-oxide (**14**) (26.3 mg, 87.6 µmol, 89%) as a colorless solid.

*Synthesis of 5-(4-methoxyphenethyl)-5H-[1,3]dioxolo[4',5':4,5]benzo[1,2-e]benzo[c][1,2]thiazine 6-oxide (**17**)*

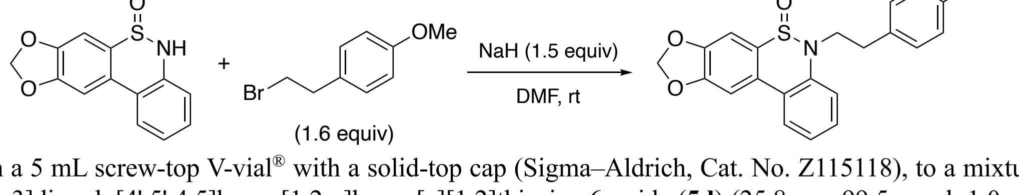

In a 5 mL screw-top V-vial® with a solid-top cap (Sigma–Aldrich, Cat. No. Z115118), to a mixture of 5*H*-[1,3]dioxolo[4',5':4,5]benzo[1,2-*e*]benzo[*c*][1,2]thiazine 6-oxide (**5d**) (25.8 mg, 99.5 µmol, 1.0 equiv) in dry DMF (600 µL) was added sodium hydride (60%, dispersion in paraffin liquid) (6.0 mg, 0.15 mmol, 1.5 equiv) at 0 ºC. After stirring the mixture for 15 min at the same temperature, 1-(2-bromoethyl)-4-methoxybenzene (**16**) (32.3 mg, 0.164 mmol, 1.6 equiv) was added to the mixture. After stirring the mixture at room temperature for 12 h, to the mixture was added ice-cold water (1 mL + 4 mL). The mixture was extracted with CH_2Cl_2 (10 mL × 3). The combined organic layer was washed with brine (10 mL) and dried with Na_2SO_4. After filtration, the filtrate was concentrated under reduced pressure. The residue was purified by preparative TLC (*n*-hexane/EtOAc = 1/1) to give 5-(4-methoxyphenethyl)-5*H*-[1,3]dioxolo[4',5':4,5]benzo[1,2-*e*]benzo[*c*][1,2]thiazine 6-oxide (**17**) (19.3 mg, 49.1 µmol, 49%) as a colorless solid.

A typical procedure for the synthesis of sulfinate esters from aryl iodides via S-Aryl benzothioate

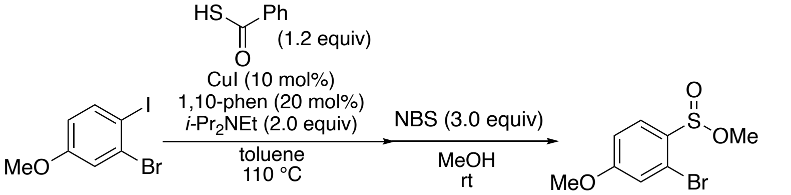

To a mixture of 2-bromo-1-iodo-4-methoxybenzene (197 mg, 0.740 mmol), 1,10-phenanthroline (26.7 mg, 0.148 mmol, 20 mol %), and CuI (9.6 mg, 74 μmol, 10 mol %) in toluene (1.5 mL) were added thiobenzoic acid (105 μL, 0.888 mmol, 1.2 equiv) and *N,N*-diisopropylethylamine (201 μL, 1.48 mmol, 2.0 equiv) at room temperature. After stirring at 110 °C (oil bath; bath temp.: 110 °C) for 19 h, the mixture was cooled to room temperature and quenched with water (20 mL). The mixture was extracted with EtOAc (10 mL × 3). The combined organic extract was washed with brine (20 mL) and dried with Na_2SO_4. After filtration, the filtrate was concentrated under reduced pressure. To the resulting mixture in MeOH (3.8 mL) was added *N*-bromosuccinimide (396 mg, 2.22 mmol, 3.0 equiv) at room temperature. After stirring for 1 h at the same temperature, to the mixture were added an aqueous saturated sodium bicarbonate (10 mL) and an aqueous saturated sodium thiosulfate (10 mL). The mixture was extracted with dichloromethane (10 mL × 3). The combined organic extract was washed with brine (10 mL) and dried with Na_2SO_4. After filtration, the filtrate was concentrated under reduced pressure. The residue was purified by column chromatography (*n*-hexane/EtOAc = 4/1) to give methyl 2-bromo-4-methoxybenzenesulfinate (**1e**) (76.9 mg, 0.290 mmol. 39%) as a pale yellow oil.

Similarly, *o*-bromo sulfinate esters **1a**–**1k** were prepared from the corresponding *o*-bromo phenyl iodides.

Synthesis of 3-bromo-4'-fluoro-4-iodo-1,1'-biphenyl

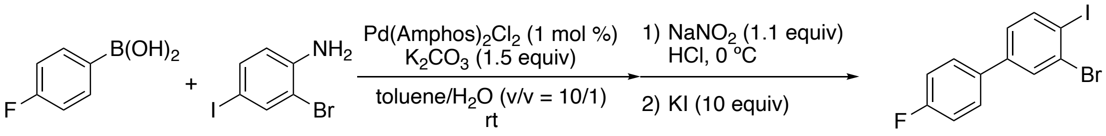

To a mixture of (4-fluorophenyl)boronic acid (350 mg, 2.50 mmol, 1.0 equiv), bis[di-*tert*-butyl(4-dimethylaminophenyl)phosphine]dichloropalladium(II) (17.7 mg, 25.0 μmol, 1 mol %), and potassium carbonate (588 mg, 3.75 mmol, 1.5 equiv) was added a solution of 2-bromo-4-iodoaniline (744 mg, 2.49 mmol, 1.0 equiv) dissolved in toluene (11 mL) and H_2O (1.1 mL) at room temperature. The mixture was stirred for 24 h at the same temperature. Then, to the mixture was added an aqueous saturated ammonium chloride (10 mL). The mixture was extracted with EtOAc (10 mL × 3). The combined mixture was washed with brine (10 mL) and dried with Na_2SO_4. After filtration, the filtrate was concentrated under reduced pressure. To the residue was added 12 M aqueous HCl (2.5 mL) at 0 °C. Then, to the mixture was added sodium nitrite (190 mg, 2.76 mmol, 1.1 equiv) in water (2.0 mL) dropwise at 0 °C and stirred for 15 min at the same temperature. Potassium iodide (4.16 g, 25.0 mmol, 10 equiv) dissolved in water (4.8 mL) was added dropwise to the mixture at 0 °C. After stirring at room temperature for 18 h, to the mixture was added an aqueous saturated sodium thiosulfate (20 mL). The mixture was extracted with EtOAc (15 mL × 3). The combined organic extract was washed with brine (10 mL). The mixture was dried with Na_2SO_4. After filtration, the filtrate was concentrated under reduced pressure. The residue was purified by column chromatography (*n*-hexane only) to give 3-bromo-4'-fluoro-4-iodo-1,1'-biphenyl (397 mg, 1.05 mmol, 42%) as a colorless solid.

Similarly, 3-bromo-4'-chloro-4-iodo-1,1'-biphenyl was prepared from the corresponding boronic acid.

Synthesis of tert-butyl (4'-methyl-[1,1'-biphenyl]-4-yl)carbamate

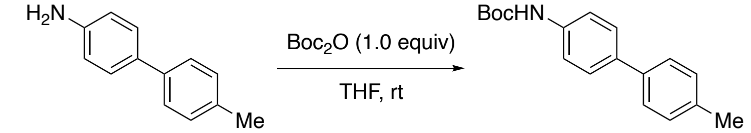

To a solution of 4'-methyl-[1,1'-biphenyl]-4-amine (1.90 g, 10.4 mmol, 1.0 equiv) in THF (10 mL) was added di-*tert*-butyl dicarbonate (2.38 mL, 10.4 mmol, 1.0 equiv) at room temperature. After stirring

the mixture for 12 h at the same temperature, the mixture was concentrated under reduced pressure. The residue was recrystallized from 2-propanol (20 mL) to give *tert*-butyl (4'-methyl-[1,1'-biphenyl]-4-yl)carbamate (2.95 g, 10.4 mmol, quant) as a colorless solid.

A typical procedure for the synthesis of the organoborons ***2***

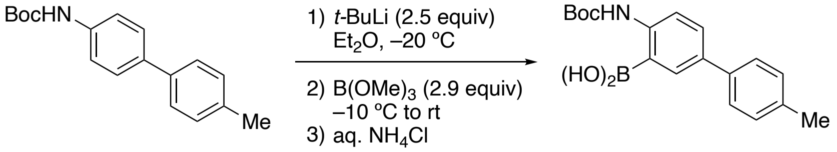

A solution of *tert*-butyllithium in *n*-pentane (7.80 mL, 1.6 M, 13 mmol, 2.4 equiv) was slowly added to *tert*-butyl (4'-methyl-[1,1'-biphenyl]-4-yl)carbamate (1.47 g, 5.19 mmol, 1.0 equiv) in dry Et_2O (18 mL) at –20 °C. After stirring the mixture at –10 °C for 3 h, trimethyl borate (1.7 mL, 15 mmol, 2.9 equiv) was added to the mixture in a single portion. The mixture was allowed to warm up to room temperature and stirred for 12 h at the same temperature. Then, to the mixture was added an aqueous saturated ammonium chloride (20 mL). The mixture was extracted with CH_2Cl_2 (20 mL × 3). The combined organic layer was basified with 1 M KOH aq. to pH 14. The mixture was extracted with water (40 mL × 3). The combined aqueous layer was acidified with 1 M HCl aq. to pH 2. The combined mixture was extracted with CH_2Cl_2 (40 mL × 3). The combined organic extract was washed with brine (20 mL) and dried with Na_2SO_4. After filtration, the filtrate was concentrated under reduced pressure to give (4-((*tert*-butoxycarbonyl)amino)-4′-methyl-[1,1′-biphenyl]-3-yl)boronic acid (**2d**) (247 mg, 0.756 mmol, 15%) as a colorless solid.

Similarly, organoborons **2a**–**f**, **2h**, and **2j** were prepared from the corresponding *N*-Boc anilines.

*Synthesis of ethyl 4-((tert-butoxycarbonyl)amino)-3-(4,4,5,5-tetramethyl-1,3,2-dioxaborolan-2-yl)benzoate (**2g**)*

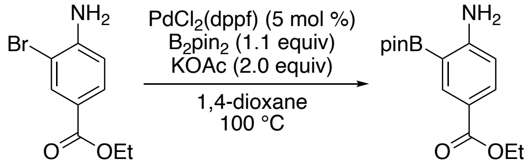

To a mixture of ethyl 4-amino-3-bromobenzoate (489 mg, 2.00 mmol, 1.0 equiv), $PdCl_2$(dppf) (81.7 mg, 0.100 mmol, 5 mol %), potassium acetate (393 mg, 4.00 mmol, 2.0 equiv), and B_2pin_2 (557 mg, 2.19 mmol, 1.1 equiv) was added 1,4-dioxane (4.0 mL) at room temperature. After stirring at 100 °C (oil bath; bath temp. : 100 °C) for 16 h, the mixture was cooled to room temperature. To the mixture was added water (20 mL). The mixture was extracted with EtOAc (20 mL × 3). The combined organic layer was washed with brine (10 mL) and dried with Na_2SO_4. After filtration, the filtrate was concentrated under reduced pressure. The residue was purified by column chromatography (*n*-hexane/EtOAc = 4/1) to give ethyl 4-amino-3-(4,4,5,5-tetramethyl-1,3,2-dioxaborolan-2-yl)benzoate (415 mg, 1.35 mmol, 67%) as a colorless solid.

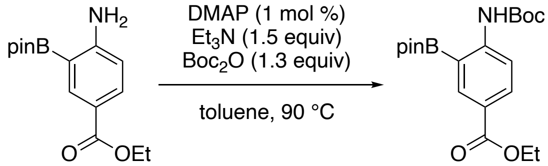

To a mixture of 4-amino-3-(4,4,5,5-tetramethyl-1,3,2-dioxaborolan-2-yl)benzoate (291 mg, 1.00 mmol, 1.0 equiv), 4-dimethylaminopyridine (2.2 mg, 10 µmol, 1 mol %) in toluene (4.0 mL) were added Boc_2O (160 µL, 1.25 mmol, 1.3 equiv) and triethylamine (209 µL, 1.50 mmol, 1.5 equiv) at room temperature. After stirring at 90 °C (oil bath; bath temp.: 90 °C) for 18 h, the mixture was cooled to room temperature. To the mixture was added an aqueous 1 M HCl (10 mL). The mixture was extracted with EtOAc (10 mL × 3). The combined organic layer was washed with brine (10 mL) and dried with Na_2SO_4. After filtration, the filtrate was concentrated under reduced pressure. The residue was purified by column chromatography (*n*-hexane/EtOAc = 10/1) to give ethyl 4-((*tert*-butoxycarbonyl)amino)-3-(4,4,5,5-tetramethyl-1,3,2-dioxaborolan-2-yl)benzoate (**2g**) (47.3 mg, 0.121 mmol, 12%) as a colorless solid.

*Synthesis of methyl (2-bromophenyl)methanesulfinate (**6**)*

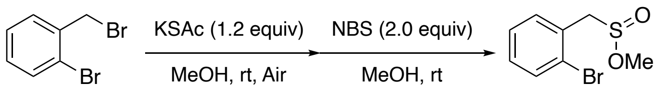

To a mixture of 1-bromo-2-(bromomethyl)benzene (681 mg, 2.73 mmol, 1.0 equiv) in MeOH (5.45 mL) was added potassium thioacetate (373 mg, 3.27 mmol, 1.2 equiv) at room temperature under air. After stirring the mixture for 1 h at the same temperature, to the mixture was added water (10 mL). The mixture was extracted with CH_2Cl_2 (10 mL × 3). The combined mixture was washed with brine (10 mL) and dried with Na_2SO_4. After filtration, the filtrate was concentrated under reduced pressure. To the resulting mixture in MeOH (13.6 mL) was added *N*-bromosuccinimide (970 mg, 5.45 mmol, 2.0 equiv) at room temperature. After stirring for 1 h at the same temperature, to the mixture were added an aqueous saturated sodium bicarbonate (10 mL) and an aqueous saturated sodium thiosulfate (10 mL). The mixture was extracted with dichloromethane (10 mL × 3). The combined organic extract was washed with brine (10 mL) and dried with Na_2SO_4. After filtration, the filtrate was concentrated under reduced pressure. The residue was purified by column chromatography (*n*-hexane/EtOAc = 4/1) to give methyl (2-bromophenyl)methanesulfinate (**6**) (661 mg, 2.65 mmol, 97%) as a pale yellow oil.

Characterization Data of New Compounds

Methyl 2-bromo-4-methoxybenzenesulfinate (**1e**)

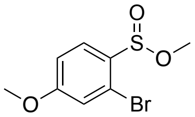

Yield: 39% (76.9 mg, 0.290 mmol); Pale yellow oil; TLC R_f 0.29 (*n*-hexane/EtOAc = 3/1); ^{1}H NMR ($CDCl_3$, 400 MHz): δ 7.84 (d, 1H, J = 8.7 Hz), 7.14 (d, 1H, J = 2.4 Hz), 7.02 (dd, 1H, J = 2.4, 8.7 Hz), 3.86 (s, 3H), 3.56 (s, 3H); $^{13}C\{^1H\}$ NMR ($CDCl_3$, 101 MHz): δ 163.2, 134.2, 128.4, 121.9, 119.0, 113.3, 55.9, 50.8; IR (NaCl, cm^{-1}) 853, 966, 1029, 1125, 1223, 1272, 1302, 1435, 1471, 1564, 1585; HRMS (ESI) *m*/*z*: $[M+Na]^+$ Calcd for $C_8H_9{}^{79}BrNaO_3S^+$ 286.9354; Found 286.9355.

Methyl 3-bromo-4'-fluoro-[1,1'-biphenyl]-4-sulfinate (**1f**)

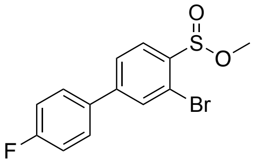

Yield: 79% (130 mg, 0.394 mmol); Pale wellow solid; Mp 77–79 °C; TLC R_f 0.62 (*n*-hexane/EtOAc = 4/1); ^{1}H NMR ($CDCl_3$, 400 MHz): δ 7.97 (d, 1H, J = 8.4 Hz), 7.79 (d, 1H, J = 1.7 Hz), 7.68 (dd, 1H, J = 1.7, 8.4 Hz), 7.60–7.52 (m, 2H), 7.21–7.13 (m, 2H), 3.63 (s, 3H); $^{13}C\{^1H\}$ NMR ($CDCl_3$, 101 MHz): δ 163.2 (d, J = 249 Hz), 145.9, 141.2, 134.4 (d, J = 3.0 Hz), 131.9, 129.0 (d, J = 8.6 Hz), 127.4, 126.2, 121.4, 116.1 (d, J = 21.5 Hz), 51.5; $^{19}F\{^1H\}$: NMR ($CDCl_3$, 377 MHz): δ –112.9 (s); IR (NaCl, cm^{-1}) 827, 970, 1023, 1100, 1130, 1162, 1235, 1372, 1458, 1515, 1587, 1601; HRMS (ESI) *m*/*z*: $[M+Na]^+$ Calcd for $C_{13}H_{10}{}^{79}BrFNaO_2S^+$ 350.9467; Found 350.9468.

Methyl 3-bromo-4'-chloro-[1,1'-biphenyl]-4-sulfinate (**1g**)

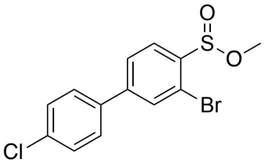

Yield: 57% (98.1 mg, 0.284 mmol); Pale yellow solid; Mp 77–79 °C; TLC R_f 0.68 (*n*-hexane/EtOAc = 3/1); ^{1}H NMR ($CDCl_3$, 400 MHz): δ 7.98 (d, 1H, J = 8.1 Hz), 7.80 (d, 1H, J = 1.7 Hz), 7.69 (dd, 1H, J = 1.7, 8.1 Hz), 7.55–7.50 (AA’BB’, 2H), 7.48–7.43 (AA’BB’, 2H), 3.64 (s, 3H); $^{13}C\{^1H\}$ NMR ($CDCl_3$, 101 MHz): δ 145.6, 141.5, 136.7, 135.0, 131.9, 129.3, 128.5, 127.5, 126.1, 121.5, 51.5; IR (NaCl, cm^{-1}) 820, 831, 960, 1012, 1020, 1097, 1116, 1504, 1538, 1544, 1583; HRMS (ESI) *m*/*z*: $[M+Na]^+$ Calcd for $C_{13}H_{10}{}^{79}Br^{35}ClNaO_2S^+$ 366.9171; Found 366.9171.

(4-((*t*ert-Butoxycarbonyl)amino)-4′-methyl-[1,1′-biphenyl]-3-yl)boronic acid (**2d**)

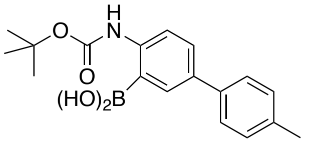

Yield: 15% (247 mg, 0.756 mmol); Colorless solid; Mp 119–121 °C; TLC R_f 0.50 (CH_2Cl_2/MeOH = 10/1); ^{1}H NMR ($CDCl_3$, 400 MHz): δ 7.61 (d, 1H, J = 2.0 Hz), 7.52–7.44 (m, 3H), 7.26–7.20 (AA’BB’, 2H), 6.95 (d, 1H, J = 8.4 Hz), 2.36 (s, 3H), 1.68 (s, 9H); $^{13}C\{^1H\}$ NMR (CD_3OD, 101 MHz): δ 159.4, 139.5, 139.1, 138.7, 137.8, 131.1, 130.4, 127.7, 127.5, 116.5, 87.8, 28.7, 21.1 (the signal for the carbon which is attached to the boron atom was not observed); IR (NaCl, cm^{-1}) 1103, 1155, 1248, 1607, 1694, 1723, 2855; HRMS (ESI) *m*/*z*: $[M+Na]^+$ Calcd for $C_{18}H_{22}BNNaO_4^+$ 350.1534; Found 350.1543.

In the thiaphenanthridinone synthesis, we used arylboronic acid **2d** immediately after the preparation due to its instability. Unfortunately, arylboronic acid **2d** was decomposed even stored at –30 °C. Thus, we could not obtain ^{11}B NMR spectrum for **2d**.

Ethyl 4-((*tert*-butoxycarbonyl)amino)-3-(4,4,5,5-tetramethyl-1,3,2-dioxaborolan-2-yl)benzoate (**2g**)

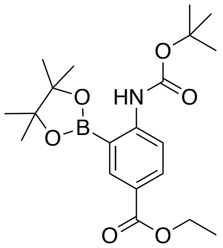

Yield: 12% (47.3 mg, 0.121 mmol); Colorless solid; Mp 126–128 °C; TLC R_f 0.35 (*n*-hexane/EtOAc = 10/1); ^{1}H NMR ($CDCl_3$, 400 MHz): δ 8.92 (br s, 1H), 8.39 (d, 1H, *J* = 2.2 Hz), 8.28 (d, 1H, *J* = 8.8 Hz), 8.07 (dd, 1H, *J* = 2.2, 8.8 Hz), 4.35 (q, 2H, *J* = 7.1 Hz), 1.53 (s, 9H), 1.42–1.34 (m, 15H); ^{13}C{^{1}H}NMR ($CDCl_3$, 101 MHz): δ 166.3, 152.7, 149.1, 138.0, 134.2, 123.3, 116.7, 84.5, 80.4, 60.6, 28.3, 24.8, 14.4 (the signal for the carbon which is attached to the boron atom was not observed); ^{11}B NMR ($CDCl_3$, 128 MHz): δ 30.3; IR (NaCl, cm^{-1}) 1126, 1145, 1160, 1233, 1299, 1391, 1417, 1531, 1590, 1710, 1728, 3342; HRMS (ESI) *m*/*z*: [M+Na]$^+$ Calcd for $C_{20}H_{30}BNNaO_6^+$ 414.2064; Found 414.2060.

(5-((*tert*-Butoxycarbonyl)amino)benzo[*d*][1,3]dioxol-4-yl)boronic acid (**2j**)

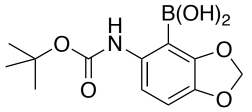

Yield: 48% (674 mg, 2.40 mmol); Brown solid; Mp 129–131 °C; TLC R_f 0.44 (CH_2Cl_2/MeOH = 10/1); ^{1}H NMR (CD_3OD, 400 MHz): δ 6.69 (d, 1H, *J* = 8.0 Hz), 6.39 (d, 1H, *J* = 8.0 Hz), 5.90 (s, 2H), 1.64 (s, 9H); ^{13}C{^{1}H} NMR (CD_3OD, 101 MHz): δ 158.9, 150.7, 145.1, 134.7, 109.0, 108.7, 102.1, 87.6, 28.7 (the signal for the carbon which is attached to the boron atom was not observed); IR (NaCl, cm^{-1}) 1046, 1162, 1235, 1402, 1515, 1520, 1605, 1716, 2855; HRMS (ESI) *m*/*z*: [M+Na]$^+$ Calcd for $C_{12}H_{16}BNNaO_6^+$ 304.0963; Found 304.0971.
In the thiaphenanthridinone synthesis, we used arylboronic acid **2j** immediately after the preparation due to its instability. Unfortunately, arylboronic acid **2j** was decomposed even stored at –30 °C. Thus, we could not obtain ^{11}B NMR spectrum for **2j**.

5-(*tert*-Butoxycarbonyl)-6-thiaphenanthridin-6(5*H*)-one (**3a**)

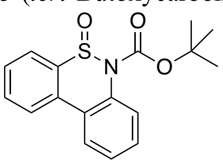

Yield: 89% (21.7 mg, 68.9 μmol); Brown solid; Mp 138–140 °C; TLC R_f 0.22 (*n*-hexane/EtOAc = 5/1); ^{1}H NMR ($CDCl_3$, 400 MHz): δ 8.06 (d, 1H, *J* = 8.0 Hz), 8.00 (dd, 1H, *J* = 1.2, 8.0 Hz), 7.85–7.76 (m, 2H), 7.72 (ddd, 1H, *J* = 1.2, 7.6, 7.6 Hz), 7.57–7.46 (m, 2H), 7.42 (ddd, 1H, *J* = 1.2, 7.6, 7.6 Hz), 1.58 (s, 9H); ^{13}C{^{1}H} NMR ($CDCl_3$, 101 MHz): δ 150.7, 138.8, 132.9, 129.3, 129.2, 129.0, 128.1, 127.04, 126.95, 126.5, 126.0, 125.6, 125.4, 84.9, 28.1; IR (NaCl, cm^{-1}) 947, 959, 1053, 1095, 1120, 1148, 1222, 1296, 1637, 1733; HRMS (ESI) *m*/*z*: [M+Na]$^+$ Calcd for $C_{17}H_{17}NNaO_3S^+$ 338.0827; Found 338.0828.

5-(*tert*-Butoxycarbonyl)-9-methyl-6-thiaphenanthridin-6(5*H*)-one (**3b**)

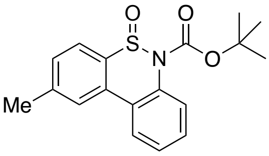

Yield: 83% (22.4 mg, 68.0 μmol); Colorless solid; Mp 141–143 °C; TLC R_f 0.55 (*n*-hexane/EtOAc = 2/1); ^{1}H NMR ($CDCl_3$, 400 MHz): δ 7.97 (dd, 1H, *J* = 1.2, 8.0 Hz), 7.85 (s, 1H), 7.77 (dd, 1H, *J* = 1.2, 8.0 Hz), 7.71 (d, 1H, *J* = 7.6 Hz), 7.48 (ddd, 1H, *J* = 1.2, 8.0, 8.0 Hz), 7.40 (ddd, 1H, *J* = 1.2, 8.0, 8.0 Hz), 7.34 (dd, 1H, *J* = 0.8, 7.6 Hz), 2.52 (s, 3H), 1.58 (s, 9H); ^{13}C{^{1}H} NMR ($CDCl_3$, 101 MHz): δ 150.8, 143.5, 136.2,

129.3, 129.1, 128.9, 128.8, 127.01, 126.97, 126.4, 126.0 (two signals overlapped), 125.3, 84.7, 28.0, 21.9; IR (NaCl, cm^{-1}) 834, 953, 1105, 1148, 1155, 1256, 1279, 1299, 1721, 1731; HRMS (ESI) *m/z*: $[M+Na]^+$ Calcd for $C_{18}H_{19}NNaO_3S^+$352.0983; Found 352.0983.

tert-Butyl 3-methyl-6*H*-dibenzo[*c*,*e*][1,2]thiazine-6-carboxylate 5-oxide (**3c**)

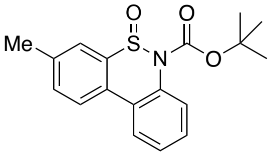

Yield: 68% (18.1 mg, 54.9 μmol); Gray solid; Mp 76–78 °C; TLC R_f 0.47 (*n*-hexane/EtOAc = 3/1); ^{1}H NMR ($CDCl_3$, 400 MHz): δ 7.97–7.91 (m, 2H), 7.77 (dd, 1H, *J* = 1.2, 8.2 Hz), 7.64–7.60 (br, 1H), 7.51 (dd, 1H, *J* = 0.6, 8.8 Hz), 7.46 (ddd, 1H, *J* = 1.6, 8.2, 8.2 Hz), 7.39 (ddd, 1H, *J* = 1.2, 8.2, 8.2 Hz), 2.49 (s, 3H), 1.58 (s, 9H); $^{13}C\{^1H\}$ NMR ($CDCl_3$, 101 MHz): δ 150.7, 138.6, 138.5, 133.7, 128.9, 128.8, 127.4, 126.9, 126.4, 126.2, 126.0, 125.4, 125.1, 84.7, 28.0, 21.1; IR (NaCl, cm^{-1}) 838, 959, 1096, 1156, 1249, 1295, 1457, 1727; HRMS (ESI) *m/z*: $[M+Na]^+$ Calcd for $C_{18}H_{19}NNaO_3S^+$ 352.0983; Found 352.0984.

5-(*tert*-Butoxycarbonyl)-7,9-dimethyl-6-thiaphenanthridin-6(5*H*)-one (**3d**)

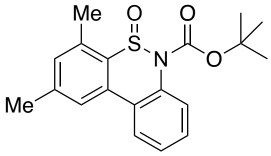

Yield: 18% (5.0 mg, 15 μmol); Colorless solid; Mp 138–140 °C; TLC R_f 0.44 (*n*-hexane/EtOAc = 3/1); ^{1}H NMR ($CDCl_3$, 400 MHz): δ 7.93 (dd, 1H, *J* = 1.2, 7.6 Hz), 7.76 (dd, 1H, *J* = 1.2, 7.6 Hz), 7.66 (s, 1H), 7.47 (ddd, 1H, *J* = 1.2, 7.6, 7.6 Hz), 7.38 (ddd, 1H, *J* = 1.2, 7.6, 7.6 Hz), 7.13 (s, 1H), 2.74 (s, 3H), 2.46 (s, 3H), 1.57 (s, 9H); $^{13}C\{^1H\}$ NMR ($CDCl_3$, 101 MHz): δ 142.5, 140.0, 135.9, 131.3, 129.5, 129.0, 128.8, 126.9, 126.8, 126.4, 125.7 (two signals overlapped), 124.0, 84.5, 28.1, 21.7, 18.3; IR (NaCl, cm^{-1}) 806, 836, 1032, 1050, 1096, 1155, 1261, 1301, 1601, 1734; HRMS (ESI) *m/z*: $[M+Na]^+$ Calcd for $C_{19}H_{21}NNaO_3S^+$ 366.1140; Found 366.1139.

tert-Butyl 2-methoxy-6*H*-dibenzo[*c*,*e*][1,2]thiazine-6-carboxylate 5-oxide (**3e**)

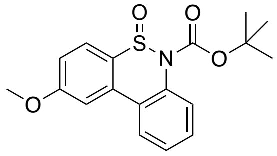

Yield: 36% (10.1 mg, 29.2 μmol); Brown solid; Mp 141–142 °C; TLC R_f 0.27 (*n*-hexane/EtOAc = 3/1); ^{1}H NMR ($CDCl_3$, 400 MHz): δ 7.93 (dd, 1H, *J* = 1.5, 7.9 Hz), 7.80–7.73 (m, 2H), 7.52–7.46 (m, 2H), 7.40 (ddd, 1H, *J* = 1.2, 7.6, 7.6 Hz), 7.03 (dd, 1H, *J* = 2.5, 8.5 Hz), 3.95 (s, 3H), 1.58 (s, 9H); $^{13}C\{^1H\}$ NMR ($CDCl_3$, 101 MHz): δ 163.0, 150.7, 131.6, 130.9, 129.4 (two signals overlapped), 128.9, 127.0, 126.4, 126.0, 125.4, 113.4, 110.9, 84.7, 55.7, 28.0; IR (NaCl, cm^{-1}) 807, 956, 1020, 1089, 1158, 1218, 1301, 1564, 1605, 1651, 1713, 3417; HRMS (ESI) *m/z*: $[M+Na]^+$ Calcd for $C_{18}H_{19}NNaO_4S^+$ 368.0933; Found 368.0933.

tert-Butyl 2-(4-fluorophenyl)-6*H*-dibenzo[*c*,*e*][1,2]thiazine-6-carboxylate 5-oxide (**3f**)

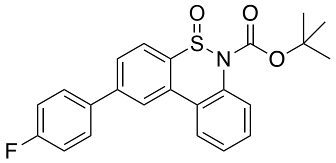

Yield: 78% (25.4 mg, 62.0 μmol); Pale yellow solid; Mp 150–152 °C; TLC R_f 0.40 (*n*-hexane/EtOAc = 3/1); ^{1}H NMR ($CDCl_3$, 400 MHz): δ 8.17 (d, 1H, *J* = 1.2 Hz), 8.05 (dd, 1H, *J* = 1.2, 8.0 Hz), 7.88 (d, 1H, *J* = 8.0 Hz), 7.81 (dd, 1H, *J* = 1.2, 7.8 Hz), 7.68 (dd, 1H, *J* = 1.7, 7.8 Hz), 7.65–7.59 (m, 2H), 7.53 (ddd, 1H, *J* = 1.7, 7.8, 7.8 Hz), 7.43 (ddd, 1H, *J* = 1.2, 7.8, 7.8 Hz), 7.24–7.17 (m, 2H), 1.60 (s, 9H); $^{13}C\{^1H\}$ NMR ($CDCl_3$, 101 MHz): δ 163.1 (d, *J* = 248 Hz), 150.7, 145.0, 137.5, 135.9 (d, *J* = 3.3 Hz), 129.5, 129.4 (d, *J*

= 8.3 Hz), 129.2, 129.1, 127.6, 127.0, 126.6, 126.5, 126.0, 125.4, 124.2, 116.1 (d, *J* = 21.4 Hz), 84.9, 28.0; ^{19}F{^{1}H}: NMR ($CDCl_3$, 377 MHz): δ –113.5 (s); IR (NaCl, cm^{-1}) 823, 956, 1109, 1152, 1236, 1252, 1302, 1514, 1603, 1727; HRMS (ESI) *m/z*: $[M+Na]^+$ Calcd for $C_{23}H_{20}FNNaO_3S^+$ 432.1046; Found 432.1047.

tert-Butyl 2-(4-chlorophenyl)-6*H*-dibenzo[*c*,*e*][1,2]thiazine-6-carboxylate 5-oxide (**3g**)

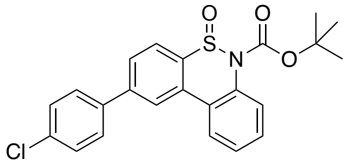

Yield: 76% (26.2 mg, 61.5 μmol); Pale yellow solid; Mp 153–155 °C; TLC R_f 0.37 (*n*-hexane/EtOAc = 3/1); ^{1}H NMR ($CDCl_3$, 400 MHz): δ 8.18 (d, 1H, *J* = 1.6 Hz), 8.05 (dd, 1H, *J* = 1.6, 8.0 Hz), 7.89 (d, 1H, *J* = 8.0 Hz), 7.81 (dd, 1H, *J* = 1.2, 8.2 Hz), 7.69 (dd, 1H, *J* = 1.7, 8.0 Hz), 7.61–7.57 (AA'BB', 2H), 7.55–7.46 (m, 3H), 7.43 (ddd, 1H, *J* = 1.7, 8.0, 8.0 Hz), 1.59 (s, 9H); ^{13}C{^{1}H} NMR ($CDCl_3$, 101 MHz): δ 150.7, 144.8, 138.2, 137.8, 134.8, 129.5, 129.4, 129.3, 128.7 (two signals overlapped), 127.6, 127.1, 126.6, 126.5, 125.9, 125.4, 124.2, 84.9, 28.1; IR (NaCl, cm^{-1}) 954, 1099, 1153, 1251, 1273, 1285, 1305, 1597, 1721; HRMS (ESI) *m/z*: $[M+Na]^+$ Calcd for $C_{23}H_{20}{}^{35}ClNNaO_3S^+$ 448.0750; Found 448.0751.

tert-Butyl 2-chloro-6*H*-dibenzo[*c*,*e*][1,2]thiazine-6-carboxylate 5-oxide (**3h**)

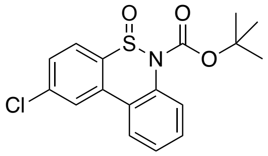

Yield: 51% (14.3 mg, 40.9 μmol); Off-white solid; Mp 139–141 °C; TLC R_f 0.49 (*n*-hexane/EtOAc = 3/1); ^{1}H NMR ($CDCl_3$, 400 MHz): δ 8.02 (d, 1H, *J* = 2.0 Hz), 7.94 (dd, 1H, *J* = 1.4, 7.9 Hz), 7.81–7.74 (m, 2H), 7.56–7.48 (m, 2H), 7.42 (ddd, 1H, *J* = 1.4, 7.6, 7.6 Hz), 1.59 (s, 9H); ^{13}C{^{1}H} NMR ($CDCl_3$, 101 MHz): δ 150.5, 139.1, 137.1, 130.7, 129.9, 129.2, 128.3, 128.0, 127.0, 126.6, 125.8, 125.4, 125.0, 85.2, 28.0; IR (NaCl, cm^{-1}) 827, 962, 1096, 1149, 1223, 1292, 1369, 1554, 1584, 1734; HRMS (ESI) *m/z*: $[M+Na]^+$ Calcd for $C_{17}H_{16}{}^{35}ClNNaO_3S^+$ 372.0437; Found 372.0437.

6-(*tert*-Butyl) 2-ethyl 6*H*-dibenzo[*c*,*e*][1,2]thiazine-2,6-dicarboxylate 5-oxide (**3i**)

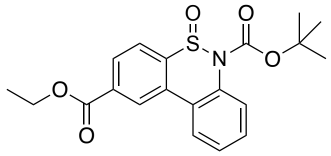

Yield: 63% (244 mg, 0.629 mmol); Off-white solid; Mp 146–148 °C; TLC R_f 0.35 (*n*-hexane/EtOAc = 3/1); ^{1}H NMR ($CDCl_3$, 400 MHz): δ 8.73 (d, 1H, *J* = 1.5 Hz), 8.18 (dd, 1H, *J* = 1.5, 8.0 Hz), 8.08 (dd, 1H, *J* = 1.5, 8.0 Hz), 7.90 (d, 1H, *J* = 8.0 Hz), 7.79, (dd, 1H, *J* = 1.3, 8.0 Hz), 7.53 (ddd, 1H, *J* = 1.5, 8.0, 8.0 Hz), 7.45 (ddd, 1H, *J* = 1.3, 8.0, 8.0 Hz), 4.51–4.42 (m, 2H), 1.59 (s, 9H), 1.45 (t, 3H, *J* = 7.1 Hz); ^{13}C{^{1}H} NMR ($CDCl_3$, 101 MHz): δ 165.2, 150.5, 141.9, 134.4, 129.8, 129.21, 129.17, 128.7, 127.1, 126.92, 126.91, 126.7, 125.6, 125.5, 85.2, 61.8, 28.0, 14.3; IR (NaCl, cm^{-1}) 840, 1103, 1156, 1251, 1276, 1294, 1324, 1371, 1480, 1716, 1721; HRMS (ESI) *m/z*: $[M+Na]^+$ Calcd for $C_{20}H_{21}NNaO_5S^+$ 410.1038; Found 410.1040.

tert-Butyl 2-(trifluoromethyl)-6*H*-dibenzo[*c*,*e*][1,2]thiazine-6-carboxylate 5-oxide (**3j**)

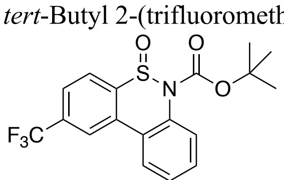

Yield: 53% (16.3 mg, 42.5 μmol); Colorless solid; Mp 141–143 °C; TLC R_f 0.45 (*n*-hexane/EtOAc = 3/1); ^{1}H NMR ($CDCl_3$, 400 MHz): δ 8.30 (s, 1H), 8.03 (dd, 1H, *J* = 1.4, 8.0 Hz), 7.96 (d, 1H, *J* = 8.0 Hz), 7.84–7.77 (m, 2H), 7.56 (ddd, 1H, *J* = 1.5, 7.8, 7.8 Hz), 7.46 (ddd, 1H, *J* = 1.5, 7.8, 7.8 Hz), 1.59 (s, 9H); ^{13}C{^{1}H}

NMR ($CDCl_3$, 101 MHz): δ 150.4, 141.6, 134.7 (q, *J* = 32.9 Hz), 130.2, 129.9, 129.3, 127.7, 127.1, 126.8, 125.5, 125.0, 124.7 (q, *J* = 3.7 Hz), 123.3 (q, *J* = 273 Hz), 122.8 (q, *J* = 3.7 Hz), 85.4, 28.0; $^{19}F\{^1H\}$ NMR ($CDCl_3$, 377 MHz): δ –62.9 (s); IR (NaCl, cm^{-1}) 1107, 1155, 1172, 1258, 1282, 1299, 1336, 1487, 1720; HRMS (ESI) *m*/*z*: $[M+Na]^+$ Calcd for $C_{18}H_{16}F_3NNaO_3S^+$ 406.0701; Found 406.0701.

5-(*tert*-Butoxycarbonyl)-3-methyl-6-thiaphenanthridin-6(5*H*)-one (**3k**)

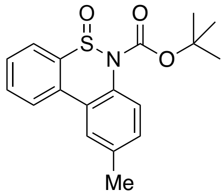

Yield: 70% (17.6 mg, 53.5 μmol); Colorless solid; Mp 145–147 °C; TLC R_f 0.53 (*n*-hexane/EtOAc = 2/1); ^1H NMR ($CDCl_3$, 400 MHz): δ 8.04 (d, 1H, *J* = 7.6 Hz), 7.83–7.75 (m, 2H), 7.73–7,64 (m, 2H), 7.52 (ddd, 1H, *J* = 1.2, 7.6, 7.6 Hz), 7.30 (dd, 1H, *J* = 1.6, 8.4 Hz), 2.46 (s, 3H), 1.57 (s, 9H); $^{13}C\{^1H\}$ NMR ($CDCl_3$, 101 MHz): δ 150.7, 138.9, 136.2, 132.8, 130.2, 129.1, 127.9, 127.0, 126.8, 126.7, 125.72, 125.71, 125.6, 84.6, 28.1, 21.2; IR (NaCl, cm^{-1}) 830, 1096, 1149, 1251, 1269, 1298, 1714, 1721; HRMS (ESI) *m*/*z*: $[M+Na]^+$ Calcd for $C_{18}H_{19}NNaO_3S^+$ 352.0983; Found 352.0976.

5-(*tert*-Butoxycarbonyl)-3-methoxy-6-thiaphenanthridin-6(5*H*)-one (**3l**)

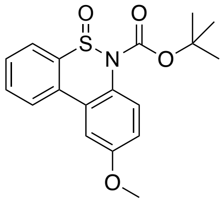

Yield: 93% (25.1 mg, 72.7 μmol); Colorless solid; Mp 152–154 °C; TLC R_f 0.58 (*n*-hexane/EtOAc = 1/1); ^1H NMR ($CDCl_3$, 400 MHz): δ 8.00 (br d, 1H, *J* = 7.6 Hz), 7.82 (dd, 1H, *J* = 1.2, 7.6 Hz), 7.74–7.67 (m, 2H), 7.54 (ddd, 1H, *J* = 1.2, 7.6, 7.6 Hz), 7.43 (d, 1H, *J* = 3.2 Hz), 7.06 (dd, 1H, *J* = 3.2, 9.2 Hz), 3.91 (s, 3H), 1.57 (s, 9H); $^{13}C\{^1H\}$ NMR ($CDCl_3$, 101 MHz): δ 157.8, 150.8, 139.1, 132.8, 128.9, 128.4, 128.2, 127.1, 127.0, 125.7, 122.2, 115.7, 109.7, 84.6, 55.6, 28.1; IR (NaCl, cm^{-1}) 1093, 1149, 1216, 1252, 1278, 1299, 1562, 1613, 1710; HRMS (ESI) *m*/*z*: $[M+Na]^+$ Calcd for $C_{18}H_{19}NNaO_4S^+$ 368.0933; Found 368.0933.

5-(*tert*-Butoxycarbonyl)-3-(4-tolyl)-6-thiaphenanthridin-6(5*H*)-one (**3m**)

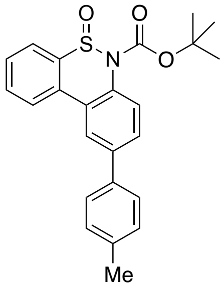

Yield: 84% (27.0 mg, 66.7 μmol); Colorless solid; Mp 166–168 °C; TLC R_f 0.61 (*n*-hexane/EtOAc = 2/1); ^1H NMR ($CDCl_3$, 400 MHz): δ 8.17–8.09 (m, 2H), 7.87–7.82 (AA'BB', 2H), 7.76–7.66 (m, 2H), 7.59–7.52 (m, 3H), 7.32–7.27 (AA'BB', 2H), 2.43 (s, 3H), 1.60 (s, 9H); $^{13}C\{^1H\}$ NMR ($CDCl_3$, 101 MHz): δ 150.7, 139.5, 138.9, 137.6, 137.4, 133.0, 129.6, 129.1 , 128.2, 128.08, 128.07, 127.2, 127.08, 127.06, 126.2, 125.7, 123.9, 84.9, 28.1, 21.2; IR (NaCl, cm^{-1}) 952, 1099, 1150, 1156, 1249, 1272, 1301, 1720; HRMS (ESI) *m*/*z*: $[M+Na]^+$ Calcd for $C_{24}H_{23}NNaO_3S^+$ 428.1296; Found 428.1300.

5-(*tert*-Butoxycarbonyl)-3-chloro-6-thiaphenanthridin-6(5*H*)-one (**3n**)

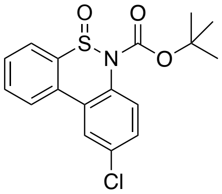

Yield: 64% (18.4 mg, 52.6 μmol); Colorless solid; Mp 150–152 °C; TLC R_f 0.46 (*n*-hexane/EtOAc = 3/1); ^{1}H NMR ($CDCl_3$, 400 MHz): δ 8.01 (d, 1H, *J* = 8.0 Hz), 7.95 (d, 1H, *J* = 2.4 Hz), 7.84 (dd, 1H, *J* = 0.8, 8.0 Hz), 7.77–7.71 (m, 2H), 7.58 (ddd, 1H, *J* = 0.8, 8.0, 8.0 Hz), 7.45 (dd, 1H, *J* = 2.4, 8.8 Hz), 1.58 (s, 9H); ^{13}C{^{1}H} NMR ($CDCl_3$, 101 MHz): δ 150.4, 139.0, 133.1, 132.1, 129.2, 128.7, 128.4, 127.9, 127.7, 127.5, 127.1, 125.7, 125.2, 85.2, 28.0; IR (NaCl, cm^{-1}) 949, 953, 1057, 1097, 1149, 1223, 1238, 1265, 1298, 1731; HRMS (ESI) *m*/*z*: [M+Na]$^+$ Calcd for $C_{17}H_{16}{}^{35}ClNNaO_3S^+$ 372.0437; Found 372.0437.

tert-Butyl 9-fluoro-6*H*-dibenzo[*c*,*e*][1,2]thiazine-6-carboxylate 5-oxide (**3o**)

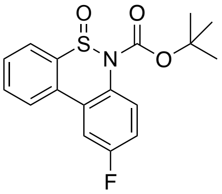

Yield: 49% (13.1 mg, 39.3 μmol); Pale brown solid; Mp 144–146 °C; TLC R_f 0.39 (*n*-hexane/EtOAc = 3/1); ^{1}H NMR ($CDCl_3$, 400 MHz): δ 8.00 (d, 1H, *J* = 7.7 Hz), 7.84 (dd, 1H, *J* = 1.1, 7.7 Hz), 7.80–7.76 (m, 1H), 7.73 (ddd, 1H, *J* = 1.4, 7.6, 7.6 Hz), 7.65 (dd, 1H, *J* = 2.9, 9.3 Hz), 7.58 (ddd, 1H, *J* = 1.1, 7.6, 7.6 Hz), 7.23–7.18 (m, 1H), 1.58 (s, 9H); ^{13}C{^{1}H} NMR ($CDCl_3$, 101 MHz): δ 160.6 (d, *J* = 246 Hz), 150.6, 139.0, 133.0, 129.0 (d, *J* = 8.5 Hz), 128.7, 128.4, 128.1 (d, *J* = 2.5 Hz), 127.9 (d, *J* = 8.6 Hz), 127.1, 125.1 (d, *J* = 2.0 Hz), 116.6 (d, *J* = 23.0 Hz), 111.6 (d, *J* = 24.1 Hz), 85.1, 28.0; ^{19}F{^{1}H}: NMR ($CDCl_3$, 377 MHz): δ –114.7 (s); IR (NaCl, cm^{-1}) 1093, 1150, 1189, 1252, 1301, 1485, 1614, 1714, 1721; HRMS (ESI) *m*/*z*: [M+Na]$^+$ Calcd for $C_{17}H_{16}{}^{19}FNNaO_3S^+$ 356.0733; Found 356.0734.

5-(*tert*-Butoxycarbonyl)-3-(ethoxycarbonyl)-6-thiaphenanthridin-6(5*H*)-one (**3p**)

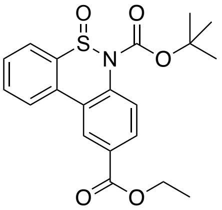

Yield: 47% (9.0 mg, 23 μmol); Brown solid; Mp 208–210 °C; TLC R_f 0.32 (*n*-hexane/EtOAc = 3/1); ^{1}H NMR ($CDCl_3$, 400 MHz): δ 8.70 (d, 1H, *J* = 1.9 Hz), 8.19–8.11 (m, 2H), 7.89–7.82 (m, 2H), 7.77 (ddd, 1H, *J* = 1.3, 7.6, 7.6 Hz), 7.59 (ddd, 1H, *J* = 1.1, 7.6, 7.6 Hz), 4.48–4.40 (m, 2H), 1.60 (s, 9H), 1.44 (t, 3H, *J* = 7.1 Hz); ^{13}C{^{1}H} NMR ($CDCl_3$, 101 MHz): δ 165.7, 150.4, 138.8, 133.14, 133.09, 129.9, 128.6, 128.4, 128.3, 127.1, 127.0, 126.8, 125.93, 125.92, 85.4, 61.3, 28.0, 14.4; IR (NaCl, cm^{-1}) 834, 1027, 1102, 1115, 1153, 1243, 1259, 1301, 1611, 1708, 1723; HRMS (ESI) *m*/*z*: [M+Na]$^+$ Calcd for $C_{20}H_{21}NNaO_5S^+$ 410.1038; Found 410.1037.

5-(*tert*-Butoxycarbonyl)-3-(trifluoromethyl)-6-thiaphenanthridin-6(5*H*)-one (**3q**)

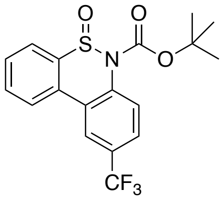

Yield: 37% (11.3 mg, 29.4 µmol); Colorless solid; Mp 203–205 °C; TLC R_f 0.56 (*n*-hexane/EtOAc = 2/1); ^{1}H NMR ($CDCl_3$, 400 MHz): δ 8.25 (s, 1H), 8.10 (d, 1H, J = 8.0 Hz), 7.94 (d, 1H, J = 8.0 Hz), 7.87 (dd, 1H, J = 1.2, 7.6 Hz), 7.81–7.65 (m, 2H), 7.62 (ddd, 1H, J = 1.2, 7.6, 7.6 Hz), 1.60 (s, 9H); ^{13}C{^{1}H} NMR ($CDCl_3$, 101 MHz): δ 150.3, 139.0, 133.3, 132.1, 129.0, 128.4 (q, J = 33.3 Hz), 127.9, 127.5, 127.1, 126.4, 125.81, 125.80 (q, J = 3.8 Hz), 123.7 (q, J = 274 Hz), 122.7 (q, J = 5.0 Hz), 85.6, 28.0; ^{19}F{^{1}H} NMR ($CDCl_3$, 376 Hz): δ –62.4 (s); IR (NaCl, cm^{-1}) 1085, 1097, 1130, 1155, 1173, 1239, 1271, 1285, 1301, 1311, 1339, 1588, 1621, 1731; HRMS (ESI) *m*/*z*: [M+Na]$^+$ Calcd for $C_{18}H_{16}F_3NNaO_3S^+$ 406.0701; Found 406.0712.

tert-Butyl 4*H*-benzo[*e*]thieno[2,3-*c*][1,2]thiazine-4-carboxylate 5-oxide (**3r**)

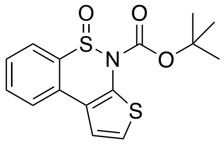

Yield: 71% (4.1 mg, 18 µmol); Purple solid; Mp 144–146 °C; TLC R_f 0.39 (*n*-hexane/EtOAc = 3/1); ^{1}H NMR ($CDCl_3$, 400 MHz): δ 7.88–7.83 (m, 2H), 7.69 (ddd, 1H, J = 1.3, 7.6, 7.6 Hz), 7.47 (ddd, 1H, J = 1.1, 7.6, 7.6 Hz), 7.43 (d, 1H, J = 5.8 Hz), 7.20 (d, 1H, J = 5.8 Hz), 1.66 (s, 9H); ^{13}C{^{1}H} NMR ($CDCl_3$, 101 MHz): δ 150.8, 135.2, 133.1, 132.6, 127.52, 127.48, 127.0, 124.5, 123.5, 121.4, 120.7, 86.4, 28.0; IR (NaCl, cm^{-1}) 824, 871, 910, 1106, 1150, 1253, 1281, 1316, 1730; HRMS (ESI) *m*/*z*: [M+Na]$^+$ Calcd for $C_{15}H_{15}NNaO_3S_2^+$ 344.0391; Found 344.0392.

5-(*tert*-Butoxycarbonyl)-[1,3]dioxolo[4,5-*b*]-6-thiaphenanthridin-6(5*H*)-one (**3s**)

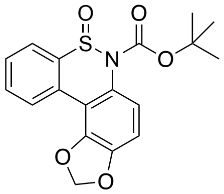

Yield: 74% (20.9 mg, 58.2 µmol); Pale red solid; Mp 150–152 °C; TLC R_f 0.58 (*n*-hexane/EtOAc = 1/1); ^{1}H NMR ($CDCl_3$, 400 MHz): δ 8.39 (dd, 1H, J = 1.6, 7.6 Hz), 7.80 (dd, 1H, J = 1.2, 7.6 Hz), 7.68 (ddd, 1H, J = 1.6, 7.6, 7.6 Hz), 7.51 (ddd, 1H, J = 1.2, 7.6, 7.6 Hz), 7.26 (d, 1H, J = 8.4 Hz), 6.95 (d, 1H, J = 8.4 Hz), 6.123 (d, 1H, J = 1.2 Hz), 6.117 (d, 1H, J = 1.2 Hz), 1.58 (s, 9H); ^{13}C{^{1}H} NMR ($CDCl_3$, 101 MHz): δ 150.8, 145.9, 144.7, 138.8, 132.5, 128.5, 128.1, 126.8, 125.8, 123.0, 120.7, 111.1, 108.8, 101.9, 84.6, 28.0; IR (NaCl, cm^{-1}) 830, 940, 1037, 1070, 1089, 1102, 1155, 1236, 1253, 1292, 1308, 1437, 1713; HRMS (ESI) *m*/*z*: [M+Na]$^+$ Calcd for $C_{18}H_{17}NNaO_5S^+$ 382.0725; Found 382.0725.

6-Benzyl-6*H*-dibenzo[*c*,*e*][1,2]thiazine 5-oxide (**3t**)

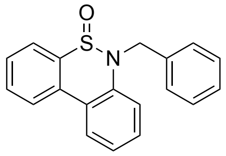

Yield: 68% (16.6 mg, 54.4 µmol); Colorless solid; Mp 157–159 °C; TLC R_f 0.19 (*n*-hexane/EtOAc = 3/1); ^{1}H NMR ($CDCl_3$, 400 MHz): δ 8.16–8.08 (m, 2H), 7.80 (dd, 1H, J = 1.1, 7.6 Hz), 7.71 (ddd, 1H, J = 1.4, 7.6, 7.6 Hz), 7.56 (ddd, 1H, J = 1.1, 7.6, 7.6 Hz), 7.38 (ddd, 1H, J = 1.5, 7.8, 7.8 Hz), 7.30–7.21 (m, 7H), 5.40 (d, 1H, J = 15.9 Hz), 5.28 (d, 1H, J = 15.9 Hz); ^{13}C{^{1}H} NMR ($CDCl_3$, 101 MHz): δ 137.4, 136.0,

134.4, 131.8, 129.9, 128.8, 128.4, 127.8, 127.7, 127.1, 126.4, 125.4, 125.1, 123.3, 123.0, 118.6, 55.6; IR (NaCl, cm^{-1}) 857, 953, 1070, 1080, 1123, 1229, 1269, 1581, 1601; HRMS (ESI) *m*/*z*: $[M+Na]^+$ Calcd for $C_{19}H_{15}NNaOS^+$ 328.0772; Found 328.0773.

5-Methyl-6-thiaphenanthridin-6(5*H*)-one (**3u**)

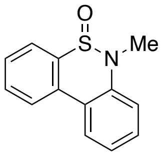

Yield: 88% (16.1 mg, 70.2 μmol) from **1a**; 98% (7.3 mg, 32 μmol) from **5a**; Colorless solid; Mp 121–123 °C; TLC R_f 0.31 (*n*-hexane/EtOAc = 1/1); ^{1}H NMR ($CDCl_3$, 400 MHz): δ 8.15–8.09 (m, 2H), 7.78 (dd, 1H, *J* = 1.2, 7.6 Hz), 7.68 (ddd, 1H, *J* = 1.2, 7.6, 7.6 Hz), 7.58–7.50 (m, 2H), 7.33–7.27 (m, 2H), 3.70 (s, 3H); ^{13}C{^{1}H} NMR ($CDCl_3$, 101 MHz): δ 136.9, 135.8, 131.8, 130.1, 128.2, 127.7, 126.4, 125.1, 124.9, 122.9, 122.6, 117.2, 38.6; IR (NaCl, cm^{-1}) 851, 1046, 1065, 1075, 1086, 1125, 1176, 1268, 1289, 1431, 1148, 1470, 1480, 1601; HRMS (ESI) *m*/*z*: $[M+Na]^+$ Calcd for $C_{13}H_{11}NNaOS^+$ 252.0459; Found 252.0459.

Methyl (2-bromophenyl)methanesulfinate (**6**)

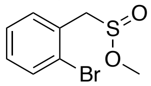

Yield: 97% (661 mg, 2.65 mmol); Pale yellow oil; TLC R_f 0.50 (*n*-hexane/EtOAc = 2/1); ^{1}H NMR ($CDCl_3$, 400 MHz): δ 7.60 (dd, 1H, *J* = 1.1, 8.0 Hz), 7.38–7.28 (m, 2H), 7.20 (ddd, 1H, *J* = 2.0, 7.6 Hz), 4.26 (d, 1H, *J* = 12.9 Hz), 4.15 (d, 1H, *J* = 12.9 Hz), 3.76 (s, 3H); ^{13}C{^{1}H} NMR ($CDCl_3$, 101 MHz): δ 132.9 (two signals overlapped), 130.0, 129.4, 127.8, 125.2, 64.4, 54.9; IR (NaCl, cm^{-1}) 966, 992, 1027, 1095, 1128, 1149, 1407, 1441, 1471, 1568, 2938, 2988, 3061; HRMS (ESI) *m*/*z*: $[M+Na]^+$ Calcd for $C_8H_9{}^{79}BrNaO_2S^+$ 270.9404; Found 270.9402.

tert-Butyl dibenzo[*c*,*e*][1,2]thiazepine-5(7*H*)-carboxylate 6-oxide (**7**)

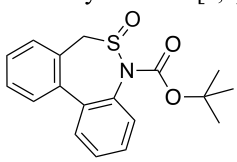

Yield: 46% (12.1 mg, 36.7 μmol); Pale yellow solid; Mp 158–160 °C; TLC R_f 0.33 (*n*-hexane/EtOAc = 3/1); ^{1}H NMR ($CDCl_3$, 400 MHz): δ 7.56–7.45 (m, 5H), 7.42–7.34 (m, 3H), 4.10 (d, 1H, *J* = 13.2 Hz), 3.56 (d, 1H, *J* = 13.2 Hz) 1.32 (s, 9H); ^{13}C{^{1}H} NMR ($CDCl_3$, 101 MHz): δ 153.1, 139.0, 138.8 132.4, 131.4, 129.6, 129.5, 129.4, 129.2, 128.9, 128.7, 128.5, 128.1, 83.7, 59.1, 27.8; IR (NaCl, cm^{-1}) 838, 963, 1086, 1113, 1158, 1190, 1223, 1258, 1306, 1704; HRMS (ESI) *m*/*z*: $[M+Na]^+$ Calcd for $C_{18}H_{19}NNaO_3S^+$ 352.0983; Found 352.0983.

Methyl 2′-((*tert*-butoxycarbonyl)amino)-[1,1′-biphenyl]-2-sulfinate (**4**)

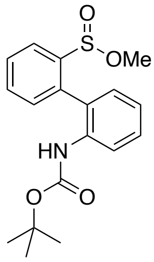

The product was obtained as a mixture of rotational isomers (1:1.5), analyzed by ^{1}H NMR($CDCl_3$) spectrum. Yield: 37% (10.0 mg, 28.8 μmol); Brown oil; TLC R_f 0.43 (*n*-hexane/EtOAc = 3/1); ^{1}H NMR ($CDCl_3$, 400 MHz): δ 8.17–8.04 (m, 2H of major + 2H of minor), 7.70–7.62 (m, 2H of major + 2H of minor), 7.46–7.29 (m, 2H of major + 2H of minor), 7.18–7.08 (m, 2H of major + 2H of minor), 6.01 (br, 1H of minor), 6.00 (br, 1H of major), 3.41 (s, 3H of minor), 3.39 (s, 3H of major), 1.434 (s, 9H of major), 1.428 (s, 9H of

minor); $^{13}C\{^1H\}$ NMR ($CDCl_3$, 101 MHz): δ 152.7, 152.5, 144.3, 143.6, 136.9, 136.5, 136.3, 135,5, 132.8, 132.4, 131.2, 130.93, 130.85, 129.9, 129.51, 129.47, 128.94, 128.90, 128.86, 126.9, 124.5, 124.3, 122.9, 122.7, 120.4, 120.3, 80.8, 80.6, 54.0, 52.3, 28.20, 28.15; IR (NaCl, cm^{-1}) 972, 1125, 1158, 1235, 1252, 1302, 1515, 1584, 1731; HRMS (ESI) *m*/*z*: $[M+Na]^+$ Calcd for $C_{18}H_{21}NNaO_4S^+$ 370.1089; Found 370.1085.

6-Thiaphenanthridin-6(5*H*)-one (**5a**)

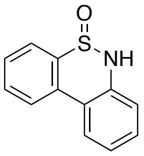

Yield: quant (90.8 mg, 0.422 mmol); Brown solid; Mp 168–170 °C; TLC R_f 0.46 (CH_2Cl_2/MeOH = 20/1); ^1H NMR ($CDCl_3$, 400 MHz): δ 8.17–8.05 (m, 2H), 7.81 (dd, 1H, *J* = 0.8, 7.6 Hz), 7.70 (ddd, 1H, *J* = 1.6, 7.6, 7.6 Hz), 7.54 (ddd, 1H, *J* = 1.2, 7.6, 7.6 Hz), 7.40 (ddd, 1H, *J* = 1.6, 7.6, 7.6 Hz), 7.34–7.21 (m, 2H), 7.10 (dd, 1H, *J* = 0.8, 7.6 Hz); $^{13}C\{^1H\}$ NMR (CD_3OD, 101 MHz): δ 136.8, 134.4, 133.3, 131.1, 129.4, 129.1, 128.1, 126.0, 125.7, 124.4, 122.0, 120.7; IR (NaCl, cm^{-1}) 1034, 1045, 1057, 1075, 1119, 1288, 1564, 1590, 3072, 3136; HRMS (ESI) *m*/*z*: $[M+Na]^+$ Calcd for $C_{12}H_9NNaOS^+$ 238.0303; Found 238.0301.

Ethyl 6*H*-dibenzo[*c*,*e*][1,2]thiazine-2-carboxylate 5-oxide (**5b**)

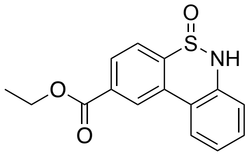

Yield: quant (143 mg, 0.482 mmol); Pale brown solid; Mp 179–181 °C; TLC R_f 0.36 (*n*-hexane/EtOAc = 2/3); ^1H NMR ($CDCl_3$, 400 MHz): δ 8.80 (d, 1H, *J* = 1.4 Hz), 8.21–8.13 (m, 2H), 7.90–7.83 (m, 2H), 7.37 (ddd, 1H, *J* = 1.2, 7.9, 7.9 Hz), 7.28 (ddd, 1H, *J* = 1.2, 7.9, 7.9 Hz), 7.03 (dd, 1H, *J* = 1.2, 7.9 Hz), 4.47 (q, 2H, *J* = 7.1 Hz), 1.46 (t, 3H, *J* = 7.1 Hz); $^{13}C\{^1H\}$ NMR ($CDCl_3$, 101 MHz): δ 165.5, 138.9, 133.4, 132.4, 130.5, 128.4, 128.2, 127.3, 126.3, 124.9, 123.7, 120.4, 119.9, 61.7, 14.3; IR (NaCl, cm^{-1}) 1035, 1112, 1263, 1299, 1322, 1564, 1607, 1721, 1779; HRMS (ESI) *m*/*z*: $[M+Na]^+$ Calcd for $C_{15}H_{13}NNaO_3S^+$ 310.0514; Found 310.0514.

9-Chloro-6*H*-dibenzo[*c*,*e*][1,2]thiazine 5-oxide (**5c**)

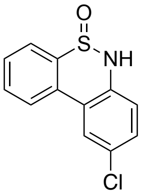

Yield: 97% (37.0 mg, 0.148 mmol); Pale yellow solid; Mp 159–161 °C; TLC R_f 0.39 (*n*-hexane/EtOAc = 1/1); ^1H NMR ($CDCl_3$, 400 MHz): δ 8.08 (br d, 1H, *J* = 8.5 Hz), 8.05 (d, 1H, *J* = 2.2 Hz), 7.82 (dd, 1H, *J* = 1.4, 7.7 Hz), 7.72 (ddd, 1H, *J* = 1.4, 7.7, 7.7 Hz), 7.58 (ddd, 1H, *J* = 1.1, 7.7, 7.7 Hz), 7.39–7.32 (m, 2H), 7.06 (d, 1H, *J* = 8.5 Hz); $^{13}C\{^1H\}$ NMR (CD_3OD, 101 MHz): δ 137.1, 133.5, 133.2, 131.0, 129.9, 129.6, 128.3, 128.2, 126.2, 125.4, 123.6, 122.3; IR (NaCl, cm^{-1}) 826, 876, 897, 1033, 1095, 1149, 1231, 1278, 1648; HRMS (ESI) *m*/*z*: $[M+Na]^+$ Calcd for $C_{12}H_8{}^{35}ClNNaOS^+$ 271.9913; Found 271.9912.

tert-Butyl 6*H*-dibenzo[*c*,*e*][1,2]thiazine-6-carboxylate 5,5-dioxide (**8**)

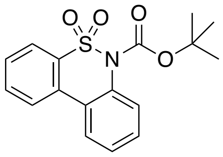

Yield: 42% (6.9 mg, 21 μmol); Colorless solid; Mp 122–124 °C; TLC R_f 0.39 (*n*-hexane/EtOAc = 3/1); ^1H NMR ($CDCl_3$, 400 MHz): δ 8.03 (dd, 1H, *J* = 0.8, 7.6 Hz), 7.96–7.89 (m, 2H), 7.75 (ddd, 1H, *J* = 1.2, 7.6

Hz), 7.58 (ddd, 1H, J = 1.2, 7.6, 7.6 Hz), 7.50–7.43 (m, 3H), 1.51 (s, 9H); $^{13}C\{^1H\}$ NMR ($CDCl_3$, 101 MHz): δ 149.2, 136.3, 135.3, 133.3, 132.7, 129.7, 128.5, 127.8, 127.3, 126.6, 126.0, 125.6, 123.7, 85.5, 27.8; IR (NaCl, cm^{-1}) 1132, 1148, 1507, 1558, 1653, 1696, 1750; HRMS (ESI) *m*/*z*: $[M+Na]^+$ Calcd for $C_{17}H_{17}NNaO_4S^+$ 354.0771; Found 354.0776.

tert-Butyl 6*H*-dibenzo[*c*,*e*][1,2]thiazine-6-carboxylate (**9**)

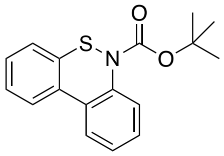

Yield: 87% (104 mg, 0.347 mmol); Pale brown solid; Mp 85–87 °C; TLC R_f 0.61 (*n*-hexane/EtOAc = 3/1); ^1H NMR ($CDCl_3$, 400 MHz): δ 7.86–7.79 (m, 1H), 7.78–7.70 (m, 1H), 7.45–7.40 (m, 1H), 7.39–7.27 (m, 5H) 1.43 (s, 9H); $^{13}C\{^1H\}$ NMR ($CDCl_3$, 101 MHz): δ 154.5, 140.7, 136.5, 132.3, 130.9, 127.9, 127.8, 127.7, 126.6, 126.5, 125.7, 125.4, 124.5, 82.7, 28.0; IR (NaCl, cm^{-1}) 960, 1067, 1112, 1155, 1223, 1241, 1258, 1299, 1366, 1429, 1706; HRMS (ESI) *m*/*z*: $[M+Na]^+$ Calcd for $C_{17}H_{17}NNaO_2S^+$ 322.0878.; Found 322.0876.

5-Fluoro-5λ^4-dibenzo[*c*,*e*][1,2]thiazine 5-oxide (**11**)

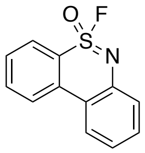

Yield: 86% (201 mg, 0.863 mmol) ; Pale brown solid; Mp 79–81 °C; TLC R_f 0.45 (*n*-hexane/EtOAc = 3/1); ^1H NMR ($CDCl_3$, 400 MHz): δ 8.32 (br d, 1H, J = 8.5 Hz), 8.20–8.11 (m, 2H), 7.92 (ddd, 1H, J = 1.1, 7.4, 8.5 Hz), 7.69 (ddd, 1H, J = 0.9, 7.4, 7.4 Hz), 7.51 (ddd, 1H, J = 1.4, 6.9, 8.3 Hz), 7.41 (br d, 1H, J = 8.3 Hz), 7.35–7.28 (m, 1H); $^{13}C\{^1H\}$ NMR ($CDCl_3$, 101 MHz): δ 141.0 (d, J = 7.3 Hz), 136.8, 135.0, 131.2 (d, J = 2.1 Hz), 128.4, 125.8 (d, J = 2.0 Hz), 124.4 (d, J = 1.2 Hz), 124.3 (d, J = 1.5 Hz), 123.8 (d, J = 2.6 Hz), 123.7 (d, J = 2.8 Hz), 121.7 (d, J = 30.4 Hz), 118.9 (d, J = 2.8 Hz); $^{19}F\{^1H\}$ NMR ($CDCl_3$, 377 MHz): δ 105.1 (s); IR (NaCl, cm^{-1}) 821, 1042, 1062, 1095, 1242, 1269, 1306, 1354, 1552, 1574, 1603; HRMS (ESI) *m*/*z*: $[M+Na]^+$ Calcd for $C_{12}H_8FNNaOS^+$ 256.0208; Found 256.0207.

5-Butyl-5λ^4-dibenzo[*c*,*e*][1,2]thiazine 5-oxide (**12**)

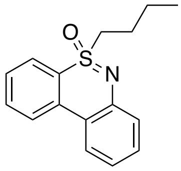

Yield: 88% (12.3 mg, 45.3 μmol); Colorless solid; Mp 84–86 °C; TLC R_f 0.45 (*n*-hexane/EtOAc = 3/1); ^1H NMR ($CDCl_3$, 400 MHz): δ 8.22 (br d, 1H, J = 8.3 Hz), 8.00 (dd, 1H, J = 1.4, 8.1 Hz), 7.86 (dd, 1H, J = 1.3, 8.3 Hz), 7.74 (ddd, 1H, J = 1.4, 7.0, 8.3 Hz), 7.57 (ddd, 1H, J = 1.0, 7.0, 8.1 Hz), 7.38 (ddd, 1H, J=1.3, 7.1, 8.3 Hz), 7.26 (dd, 1H, J = 1.2, 8.3 Hz), 7.07 (ddd, 1H, J = 1.2, 7.1, 8.3 Hz), 3.70–3.60 (m, 1H), 3.59–3.49 (m, 1H), 1.79–1.66 (m, 1H), 1.57–1.31 (m, 3H), 0.87 (t, 3H, J = 7.3 Hz); $^{13}C\{^1H\}$ NMR ($CDCl_3$, 101 MHz): δ 143.4, 135.0, 133.0, 130.7, 127.8, 124.9, 124.4, 123.7, 123.4, 122.4, 120.5, 116.8, 56.1, 25.7, 21.4, 13.5; IR (NaCl, cm^{-1}) 1013, 1116, 1152, 1185, 1206, 1231, 1295, 1315, 1548, 1573, 1598; HRMS (ESI) *m*/*z*: $[M+Na]^+$ Calcd for $C_{16}H_{17}NNaOS^+$ 294.0923; Found 294.0922.

5-(4-Methoxyphenoxy)-5λ[4]-dibenzo[*c*,*e*][1,2]thiazine 5-oxide (**13**)

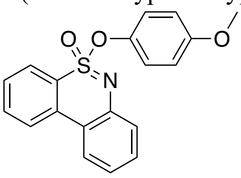

Yield: 99% (16.8 mg, 49.8 μmol); Colorless solid; Mp 76–78 °C; TLC R_f 0.28 (*n*-hexane/EtOAc = 3/1); ^{1}H NMR ($CDCl_3$, 400 MHz): δ 8.17 (br d, 1H, *J* = 8.5 Hz), 7.97 (dd, 1H, *J* = 1.4, 8.1 Hz), 7.93 (d, 1H, *J* = 1.0, 8.2 Hz), 7.80 (ddd, 1H, *J* = 1.4, 7.2, 8.5 Hz), 7.56 (ddd, 1H, *J* = 1.0, 7.2, 8.1 Hz), 7.40 (ddd, 1H, *J* = 1.0 7.2, 8.2 Hz), 7.30 (dd, 1H, *J* = 1.2, 8.2 Hz), 7.13 (ddd, 1H, *J* = 1.2, 7.2, 8.2 Hz), 6.77–6.72 (AA'BB', 2H), 6.65–6.60 (AA'BB', 2H), 3.69 (s, 3H); $^{13}C\{^1H\}$ NMR ($CDCl_3$, 101 MHz): δ 158.0, 143.3, 142.0, 136.7, 133.9, 130.8, 127,7, 125.0 (two signals overlapped), 123.9, 123.7, 123.6, 122.5, 121.9, 118.7, 114.2, 55.5; IR (NaCl, cm^{-1}) 837, 1029, 1055, 1083, 1103, 1119, 1182, 1246, 1301, 1338, 1504, 1551, 1574, 1603; HRMS (ESI) *m*/*z*: $[M+Na]^+$ Calcd for $C_{19}H_{15}NNaO_3S^+$ 360.0670; Found 360.0675.

5-Morpholinodibenzo[*c*,*e*][1,2]thiazine 5-oxide (**14**)

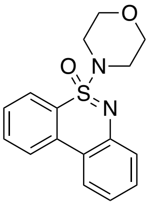

Yield: 89% (26.3 mg, 87.6 μmol); Colorless solid; Mp 81–83 °C; TLC R_f 0.25 (*n*-hexane/EtOAc = 3/1); ^{1}H NMR ($CDCl_3$, 400 MHz): δ 8.26 (br d, 1H, *J* = 8.5 Hz), 8.03 (dd, 1H, *J* = 1.4, 8.1 Hz), 7.85 (dd, 1H, *J* = 1.3, 8.3 Hz), 7.76 (ddd, 1H, *J* = 1.4, 7.2, 8.5 Hz), 7.59 (ddd, 1H, *J* = 1.0, 7.2, 8.1 Hz), 7.38 (ddd, 1H, *J* = 1.3, 7.1, 8.3 Hz), 7.29 (dd, 1H, *J* = 1.3, 8.3 Hz), 7.09 (ddd, 1H, *J* = 1.3, 7.1, 8.3 Hz), 3.80–3.63 (m, 4H), 3.02–2.98 (AA'BB', 4H); $^{13}C\{^1H\}$ NMR ($CDCl_3$, 101 MHz): δ 144.6, 136.1, 132.9, 130.5, 127.8, 124.8, 124.6, 123.3, 123.2, 121.3, 120.7, 116.4, 66.3, 46.0; IR (NaCl, cm^{-1}) 933, 1025, 1073, 1112, 1159, 1238, 1242, 1252, 1304, 1338, 1574, 1601; HRMS (ESI) *m*/*z*: $[M+Na]^+$ Calcd for $C_{16}H_{16}N_2NaO_2S^+$ 323.0830; Found 323.0822.

tert-Butyl 5*H*-[1,3]dioxolo[4',5':4,5]benzo[1,2-*e*]benzo[*c*][1,2]thiazine-5-carboxylate 6-oxide (**3v**)

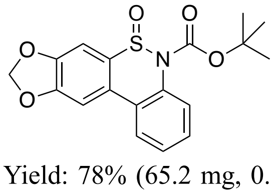

Yield: 78% (65.2 mg, 0.181 mmol); Colorless solid; Mp 154–146 °C; TLC R_f 0.37 (*n*-hexane/EtOAc = 2/1); ^{1}H NMR ($CDCl_3$, 400 MHz): δ 7.83 (dd, 1H, *J* = 1.5, 7.9 Hz), 7.76 (dd, 1H, *J* = 1.3, 8.2 Hz), 7.48–7.42 (m, 2H), 7.38 (ddd, 1H, *J* = 1.3, 7.9, 7.9 Hz), 7.24 (s, 1H), 6.12 (d, 1H, *J* = 1.3 Hz), 6.11 (d, 1H, *J* = 1.3 Hz), 1.58 (s, 9H); $^{13}C\{^1H\}$ NMR ($CDCl_3$, 101 MHz): δ 151.6, 150.6, 147.4, 132.7, 128.7, 128.5, 126.8, 126.4, 125.9, 125.1, 124.9, 107.0, 105.6, 102.3, 84.8, 28.0; IR (NaCl, cm^{-1}) 929, 1035, 1095, 1152, 1228, 1269, 1291, 1455, 1716, 1723; HRMS (ESI) *m*/*z*: $[M+Na]^+$ Calcd for $C_{18}H_{17}NNaO_5S^+$ 382.0725; Found 382.0725.

5*H*-[1,3]Dioxolo[4',5':4,5]benzo[1,2-*e*]benzo[*c*][1,2]thiazine 6-oxide (**5d**)

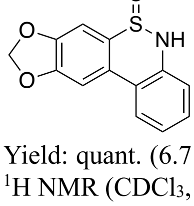

Yield: quant. (6.7 mg, 26 μmol); Colorless solid; Mp 126–128 °C; TLC R_f 0.19 (*n*-hexane/EtOAc = 1/1); ^{1}H NMR ($CDCl_3$, 400 MHz): δ 7.92 (dd, 1H, *J* = 1.4, 7.9 Hz), 7.53 (s, 1H), 7.38 (ddd, 1H, *J* = 1.4, 7.9, 7.9 Hz), 7.28–7.21 (m, 2H), 7.13 (br s, 1H), 7.10 (dd, 1H, *J* = 1.0, 7.9, 7.9 Hz), 6.12 (s, 2H); $^{13}C\{^1H\}$ NMR

(CDCl$_3$, 101 MHz): δ 151.1, 147.3, 131.7, 130.3, 129.3, 124.5, 123.9, 123.6, 121.0, 119.6, 106.9, 104.6, 102.1; IR (NaCl, cm^{-1}) 871, 1033, 1042, 1055, 1206, 1231, 1259, 1458, 1501; HRMS (ESI) *m*/*z*: [M+Na]$^+$ Calcd for $C_{13}H_9NNaO_3S^+$ 282.0201; Found 282.0200.

5-(4-Methoxyphenethyl)-5*H*-[1,3]dioxolo[4',5':4,5]benzo[1,2-*e*]benzo[*c*][1,2]thiazine 6-oxide (**17**)

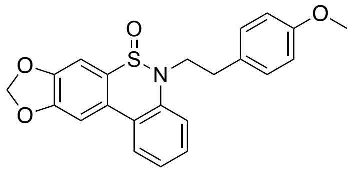

Yield: 49% (19.3 mg, 49.1 μmol); Colorless solid; Mp 121–123 °C; TLC R_f 0.43 (*n*-hexane/EtOAc = 1/1); ^{1}H NMR (CDCl$_3$, 400 MHz): δ 8.01 (dd, 1H, *J* = 1.2, 8.1 Hz), 7.53–7.47 (m, 2H), 7.40 (br d, 1H, *J* = 7.6 Hz), 7.27 (ddd, 1H, *J* = 1.1, 7.0, 8.1 Hz), 7.01 (s, 1H), 6.92–6.87 (AA'BB', 2H), 6.72–6.66 (AA'BB', 2H), 6.10 (d, 1H, *J* = 1.3 Hz), 6.09 (d, 1H, *J* = 1.3 Hz), 4.55–4.44 (m, 1H), 4.17–4.06 (m, 1H), 3.75 (s, 3H), 3.03–2.87 (m, 2H); ^{13}C{^{1}H} NMR (CDCl$_3$, 101 MHz): δ 158.2, 150.6, 147.1, 133.2, 131.5, 129.9, 129.6, 129.3, 125.3, 124.0, 123.5, 122.9, 118.0, 113.7, 105.7, 104.8, 101.0, 55.1, 53.2, 33.8; IR (NaCl, cm^{-1}) 866, 1026,1083, 1243, 1263, 1511, 1608; HRMS (ESI) *m*/*z*: [M+Na]$^+$ Calcd for $C_{22}H_{19}NNaO_4S^+$ 416.0933; Found 416.0934.

3-Bromo-4'-fluoro-4-iodo-1,1'-biphenyl

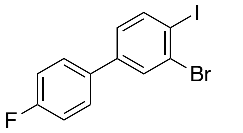

Yield: 42% (397 mg, 1.05 mmol); Colorless solid; Mp 92–94 °C; TLC R_f 0.67 (*n*-hexane); ^{1}H NMR (CDCl$_3$, 400 MHz): δ 7.89 (d, 1H, *J* = 8.2 Hz), 7.79 (d, 1H, *J* = 2.2 Hz), 7.52–7.45 (m, 2H), 7.18–7.09 (m, 3H); ^{13}C{^{1}H} NMR (CDCl$_3$, 101 MHz): δ 162.9 (d, *J* = 248 Hz), 141.9, 140.5, 134.8 (d, *J* = 3.2 Hz), 131.0, 130.2, 128.6 (d, *J* = 8.3 Hz), 127.0, 116.0 (d, *J* = 21.7 Hz), 99.5; ^{19}F{^{1}H} NMR (CDCl$_3$, 377 MHz): δ –114.9 (s); IR (NaCl, cm^{-1}) 814, 838, 1000, 1105, 1163, 1228, 1285, 1309, 1514, 1597; HRMS (EI) *m*/*z*: [M]$^{•+}$ Calcd for $C_{12}H_7{}^{79}BrFI^{•+}$ 375.8760; Found 375.8762.

3-Bromo-4'-chloro-4-iodo-1,1'-biphenyl

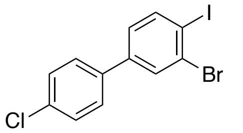

Yield: 34% (336 mg, 0.854 mmol); Colorless solid; Mp 51–53 °C; TLC R_f 0.68 (*n*-hexane); ^{1}H NMR (CDCl$_3$, 400 MHz): δ 7.90 (d, 1H, *J* = 8.2 Hz), 7.80 (d, 1H, *J* = 2.2 Hz), 7.49–7.44 (AA'BB', 2H), 7.44–7.38 (AA'BB', 2H), 7.17 (dd, 1H, *J* = 2.2, 8.2 Hz); ^{13}C{^{1}H} NMR (CDCl$_3$, 101 MHz): δ 141.6, 140.6, 137.1, 134.4, 131.0, 130.3, 129.2, 128.1, 126.9, 99.9; IR (NaCl, cm^{-1}) 811, 837, 884, 1002, 1013, 1095, 1495, 1537, 1594; HRMS (EI) *m*/*z*: [M]$^{•+}$ Calcd for $C_{12}H_7{}^{79}Br^{35}ClI^{•+}$ 391.8464; Found 391.8465.

tert-Butyl (4'-methyl-[1,1'-biphenyl]-4-yl)carbamate

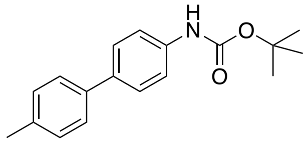

Yield: quant. (2.95 g, 10.4 mmol); Colorless solid; Mp 145–147 °C; TLC R_f 0.28 (*n*-hexane/EtOAc = 10/1); ^{1}H NMR (CDCl$_3$, 400 MHz): δ 7.54–7.38 (m, 6H), 7.25–7.20 (AA'BB', 2H), 6.50 (br s, 1H), 2.38 (s, 3H), 1.53 (s, 9H); ^{13}C{^{1}H} NMR (CDCl$_3$, 101 MHz): δ 152.8, 137.8, 137.4, 136.7, 135.9, 129.5, 127.4, 126.6, 118.8, 80.7, 28.4, 21.1; IR (NaCl, cm^{-1}) 807, 1059, 1158, 1236, 1263, 1324, 1394, 1418, 1505, 1511, 1535, 1590, 1694, 1698, 3363; HRMS (ESI) *m*/*z*: [M+Na]$^+$ Calcd for $C_{18}H_{21}NNaO_2^+$ 306.1470; Found 306.1470.

3-Bromo-4'-methyl-[1,1'-biphenyl]-4-amine

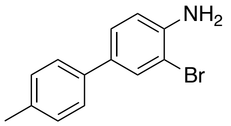

Yield: 20% (465 mg, 1.77 mmol); brown solid; Mp 91–93 °C; TLC R_f 0.27 (*n*-hexane/EtOAc = 10/1); ^{1}H NMR ($CDCl_3$, 400 MHz): δ 7.65 (d, 1H, *J* = 2.1 Hz), 7.42–7.37 (AA'BB', 2H), 7.34 (dd, 1H, *J* = 2.1, 8.3 Hz), 7.23–7.18 (AA'BB', 2H), 6.81 (d, 1H, *J* = 8.3 Hz), 4.11 (br s, 2H), 2.37 (s, 3H); $^{13}C\{^{1}H\}$ NMR ($CDCl_3$, 101 MHz): δ 143.0, 136.9, 136.5, 132.7, 130.8, 129.4, 126.8, 126.2, 115.9, 109.7, 21.0; IR (NaCl, cm^{-1}) 808, 881, 1027, 1160, 1285, 1294, 1307, 1493; HRMS (ESI) *m*/*z*: $[M+H]^+$ Calcd for $C_{13}H_{13}{}^{79}BrN^+$ 262.0231; Found 262.0227.

Ethyl 4-amino-3-(4,4,5,5-tetramethyl-1,3,2-dioxaborolan-2-yl)benzoate

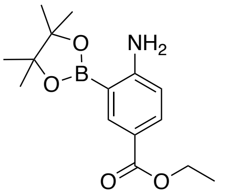

Yield: 67% (415 mg, 1.35 mmol); Colorless solid; Mp 79–81 °C; TLC R_f 0.71 (*n*-hexane/EtOAc = 1/1); ^{1}H NMR ($CDCl_3$, 400 MHz): δ 8.30 (d, 1H, *J* = 2.2 Hz), 7.88 (dd, 1H, *J* = 2.2, 8.6 Hz), 6.55 (d, 1H, *J* = 8.6 Hz), 5.14 (br s, 2H), 4.31 (q, 2H, *J* = 7.1 Hz), 1.38–1.32 (m, 15H); $^{13}C\{^{1}H\}$ NMR ($CDCl_3$, 101 MHz): δ 166.8, 157.3, 139.4, 134.3, 118.5, 113.8, 83.8, 60.2, 24.9, 14.5 (the signal for the carbon which is attached to the boron atom was not observed); ^{11}B NMR ($CDCl_3$, 128 MHz): δ 30.7; IR (NaCl, cm^{-1}) 1126, 1148, 1248, 1289, 1302, 1314, 1322, 1391, 1501, 1603, 1635, 1696, 3368, 3474; HRMS (ESI) *m*/*z*: $[M+Na]^+$ Calcd for $C_{15}H_{22}BNNaO_4{}^+$ 314.1540; Found 314.1539.

References for Supporting Information

S1 K. Nakamura, Y. Kumagai, A. Kobayashi, M. Suzuki, S. Yoshida, *Org. Biomol. Chem*. **2023**, *21*, 6886.
S2 Y. Kumagai, A. Kobayashi, K. Nakamura, S. Yoshida, *Chem. Commun*. **2024**, *60*, 1611.
S3 H. Lavrard, F. Popowycz, *Eur. J. Org. Chem*. **2017**, 600.
S4 T. Deng, E. Shi, E. Thomas, T. Driver, *Org. Lett*. **2020**, *22*, 9102.
S5 C. Ni, J. Gao, X. Fang, *Chem. Commun*. **2020**, *56*, 2654.
S6 H. Lam, J. Tsoung, M. Lautens, *J. Org. Chem*. **2017**, *82*, 6089.
S7 J. Sun, Y. Zhou, R. Gu, X. Li, X. Zhang, *Nat. Commun*. **2022**, *13*, 7093.

^{1}H, ^{13}C, ^{19}F, and ^{11}B NMR Spectra of New Compounds

^{1}H NMR (400 MHz) and ^{13}C NMR (101 MHz) spectra of methyl 2-bromo-4-methoxybenzenesulfinate (**1e**) ($CDCl_3$)

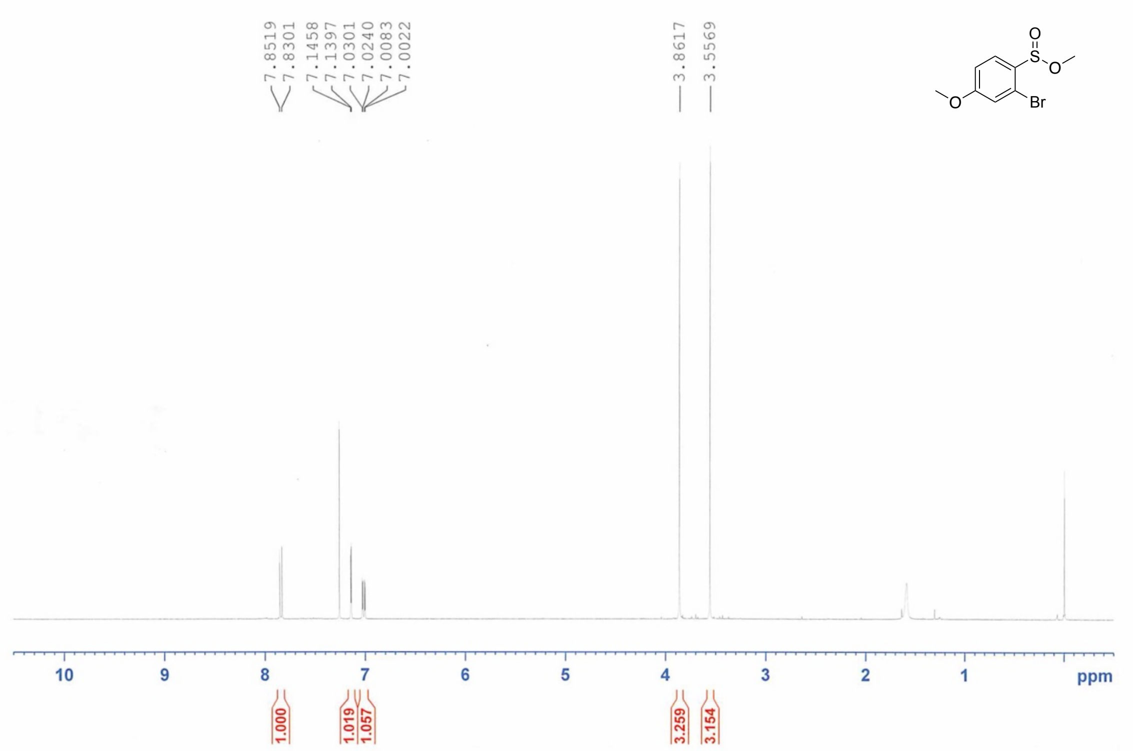

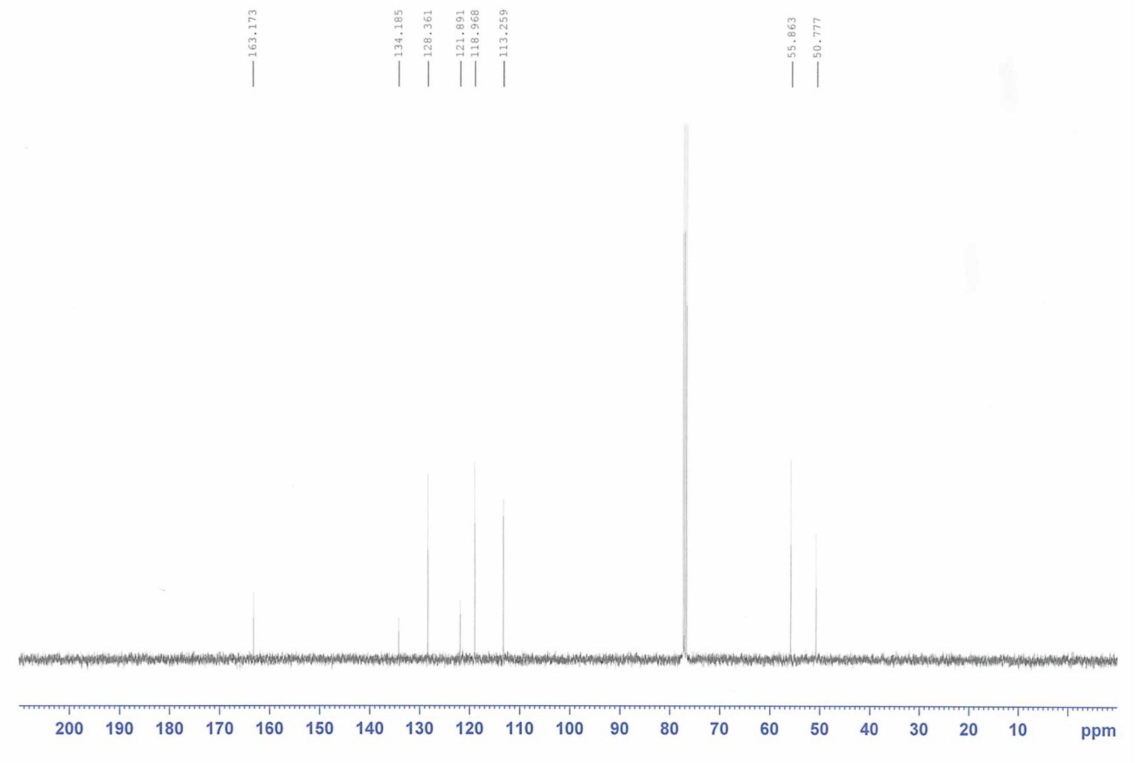

^{1}H NMR (400 MHz), ^{13}C (101 MHz), and ^{19}F NMR (376 MHz) spectra of methyl 3-bromo-4'-fluoro-[1,1'-biphenyl]-4-sulfinate (**1f**) ($CDCl_3$)

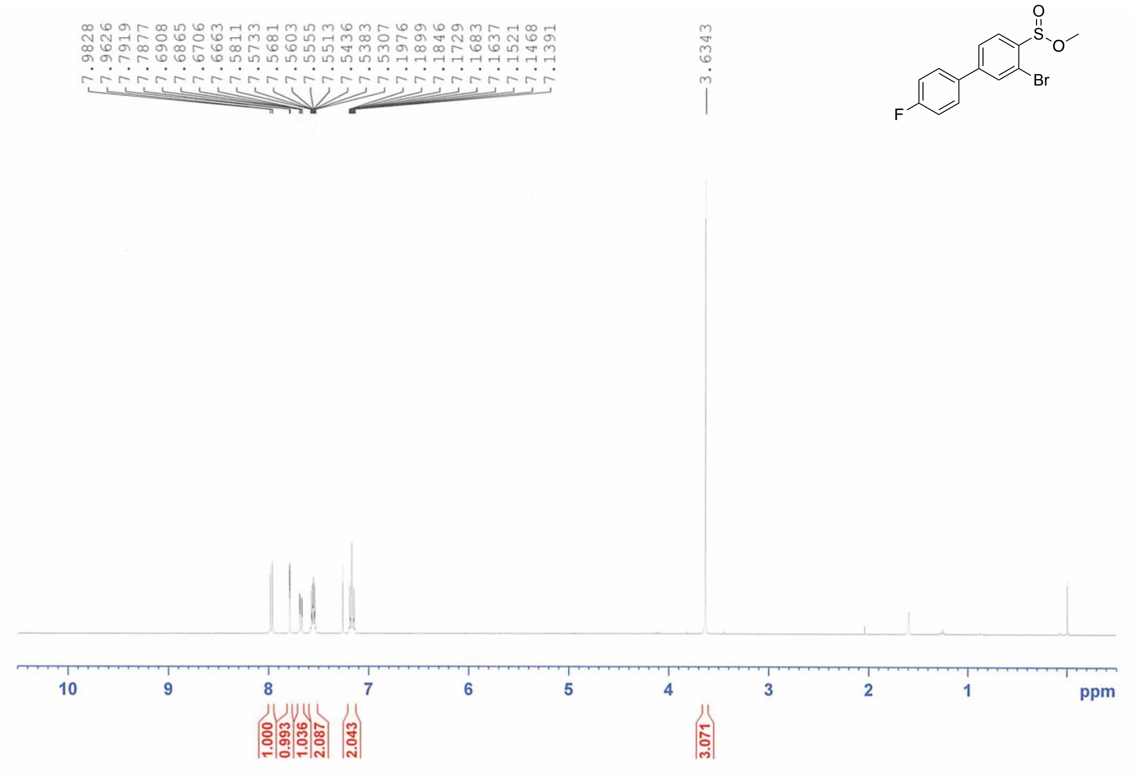

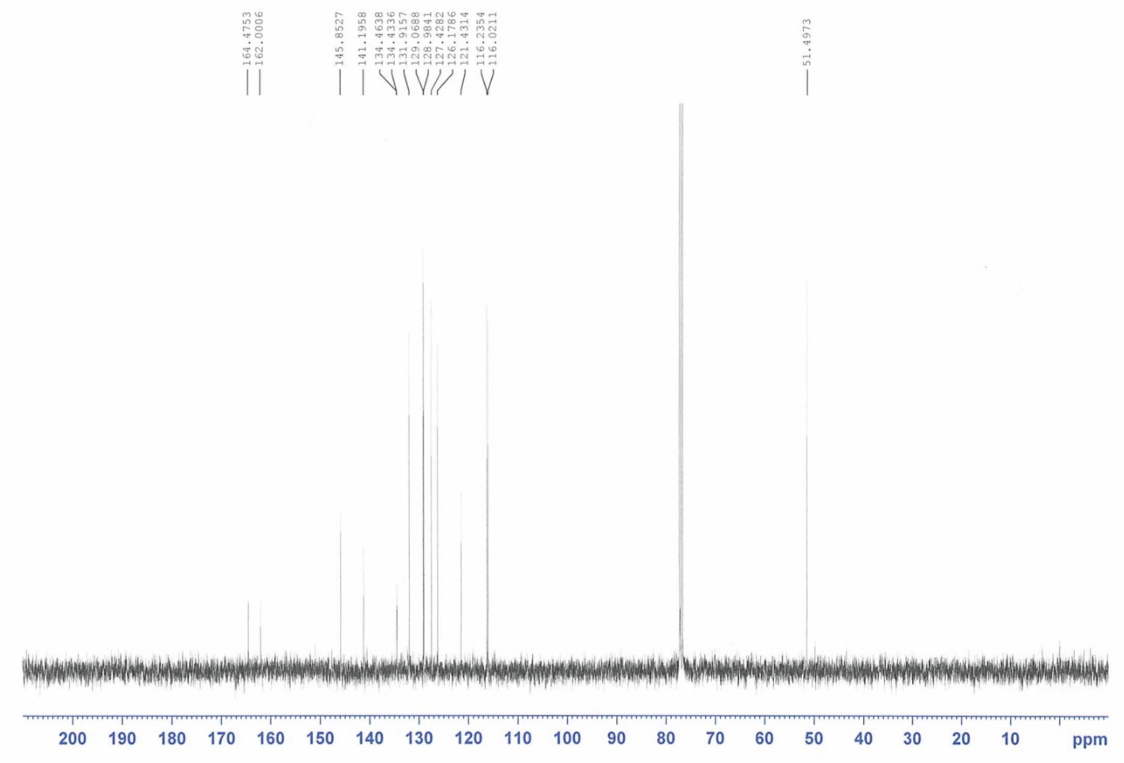

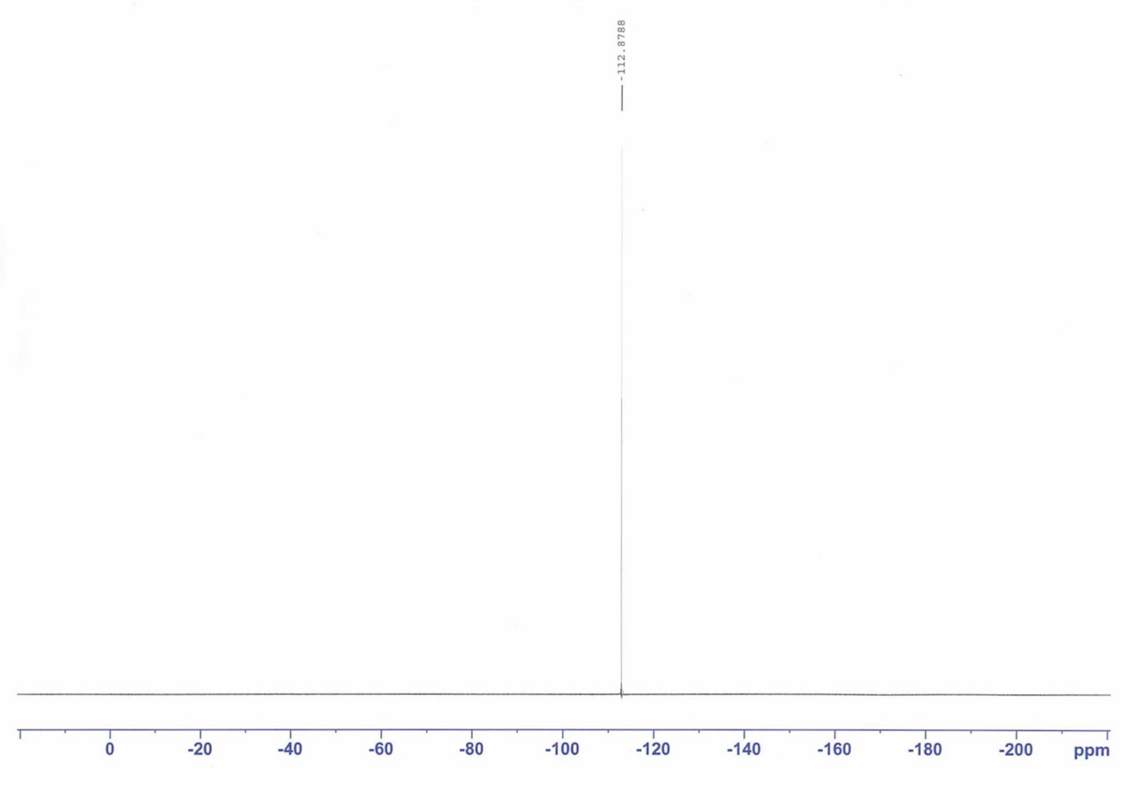
-112.8788
0
-20
-40
-60
-80
-100
-120
-140
-160
-180
-200
ppm

^{1}H NMR (400 MHz) and ^{13}C NMR (101 MHz) spectra of methyl 3-bromo-4'-chloro-[1,1'-biphenyl]-4-sulfinate (**1g**) ($CDCl_3$)

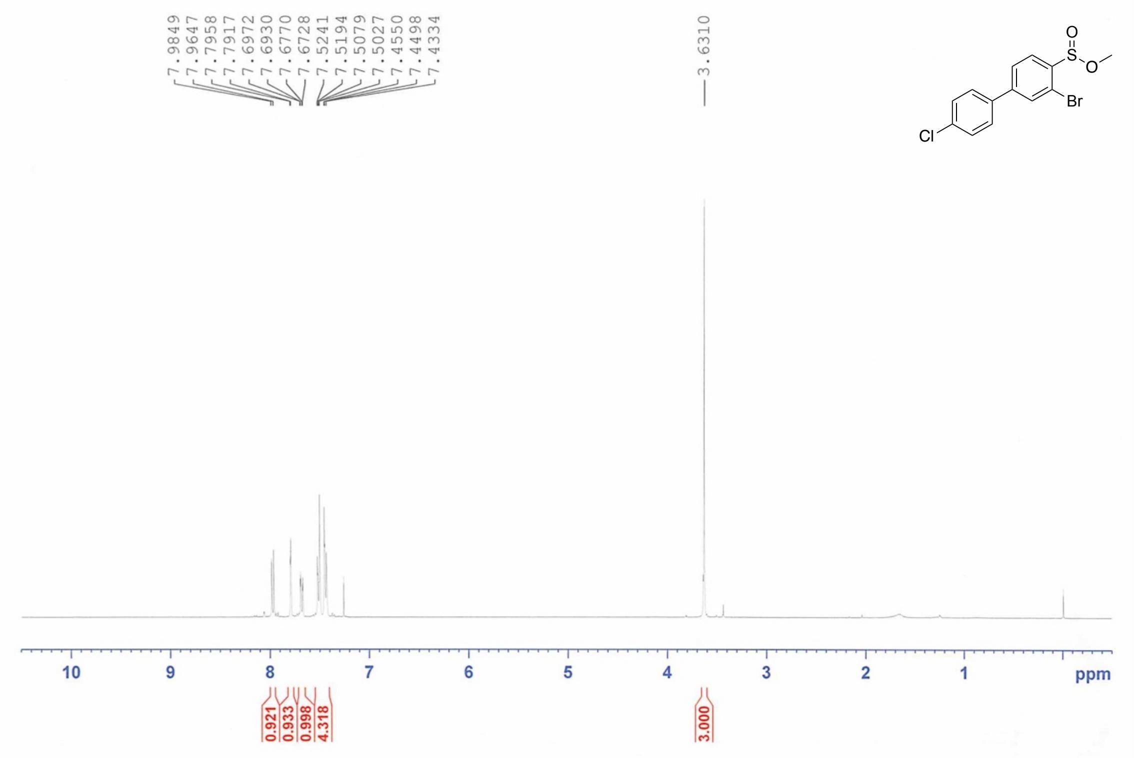

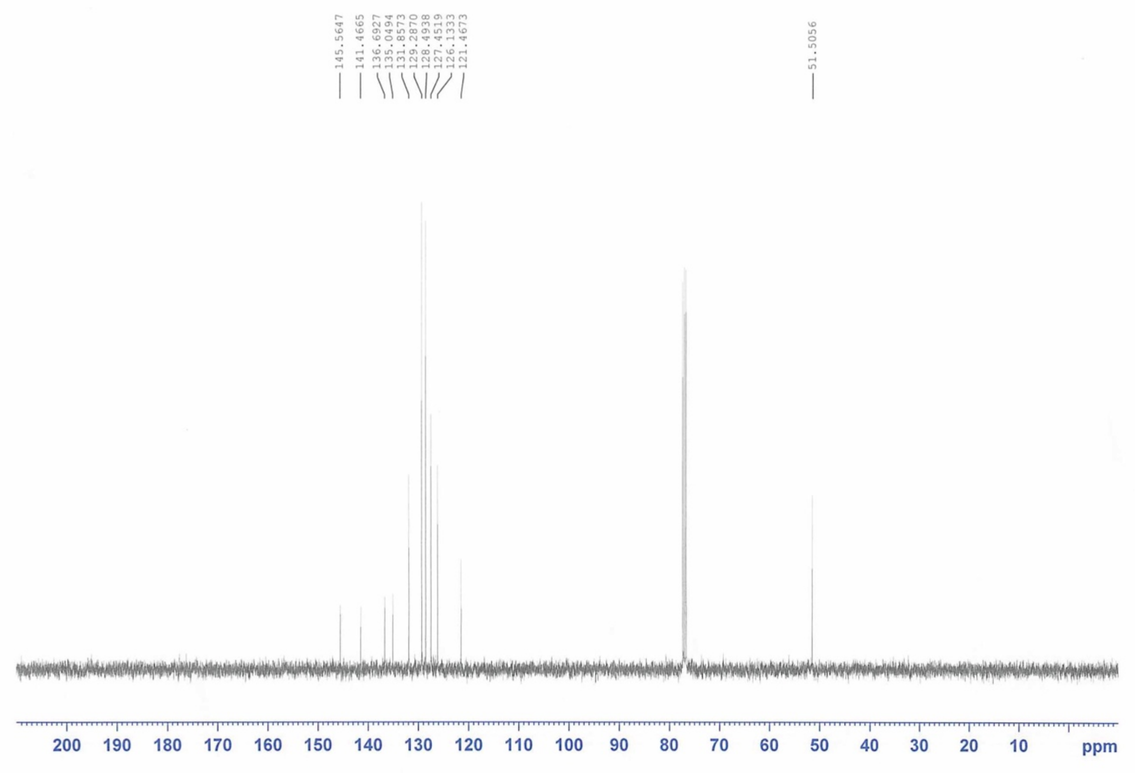

^{1}H NMR (400 MHz) and ^{13}C NMR (101 MHz) spectra of (4-((*tert*-butoxycarbonyl)amino)-4′-methyl-[1,1′-biphenyl]-3-yl)boronic acid (**2d**) (CD_3OD)

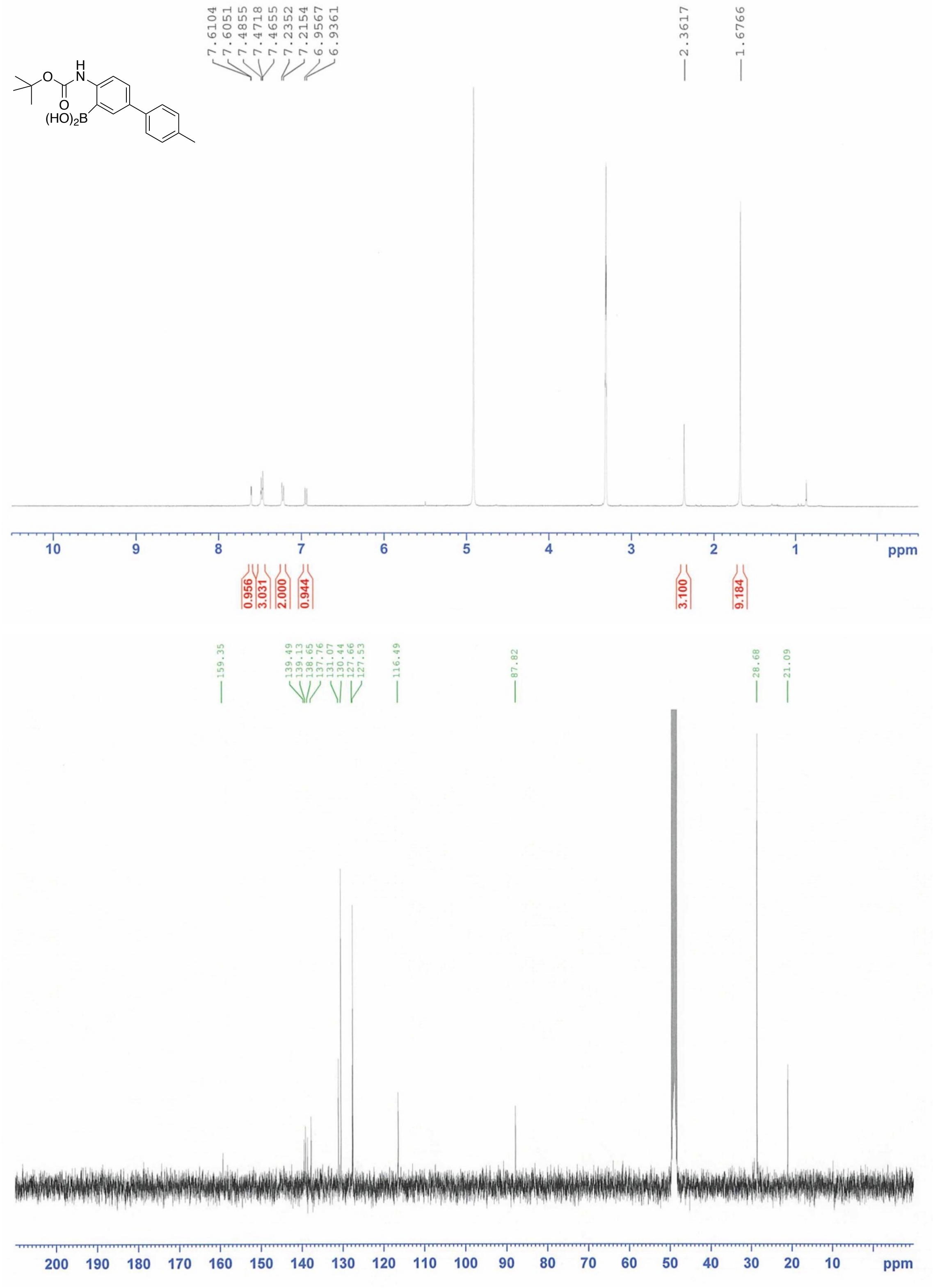

^{1}H NMR (400 MHz), ^{13}C NMR (101 MHz), and ^{11}B NMR (128 MHz) spectra of ethyl 4-((*tert*-butoxycarbonyl)amino)-3-(4,4,5,5-tetramethyl-1,3,2-dioxaborolan-2-yl)benzoate (**2g**) ($CDCl_3$)

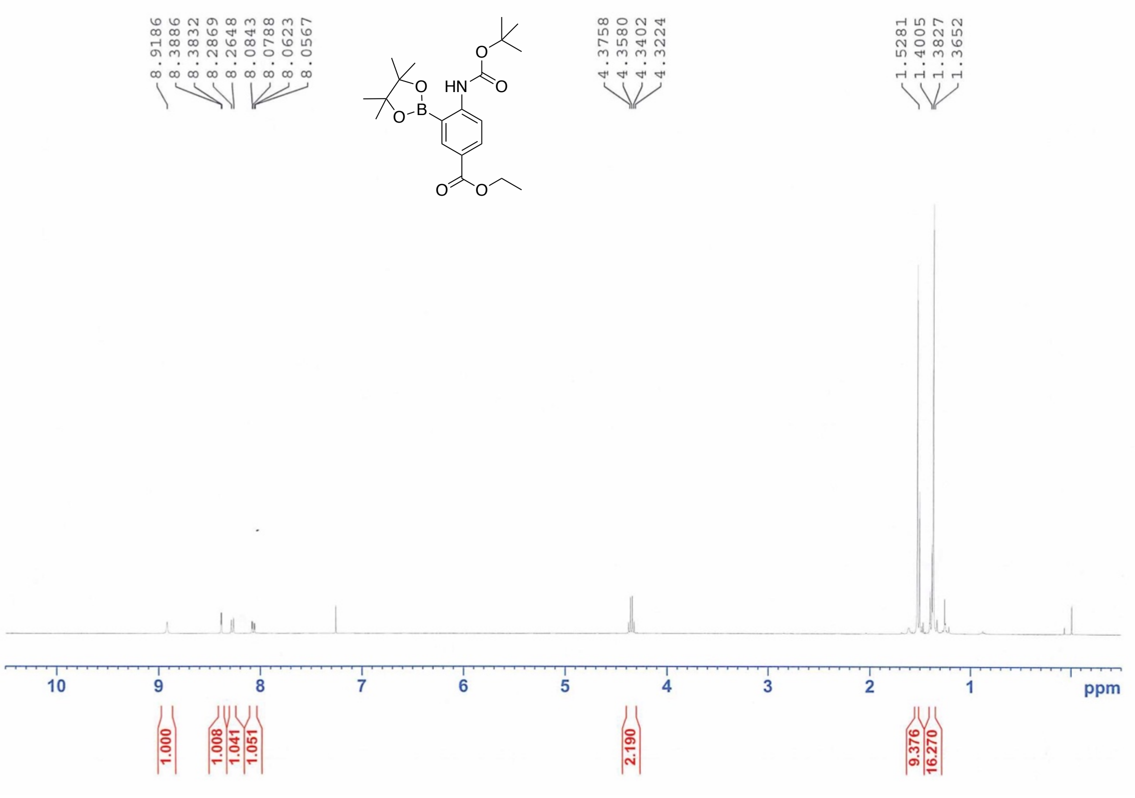

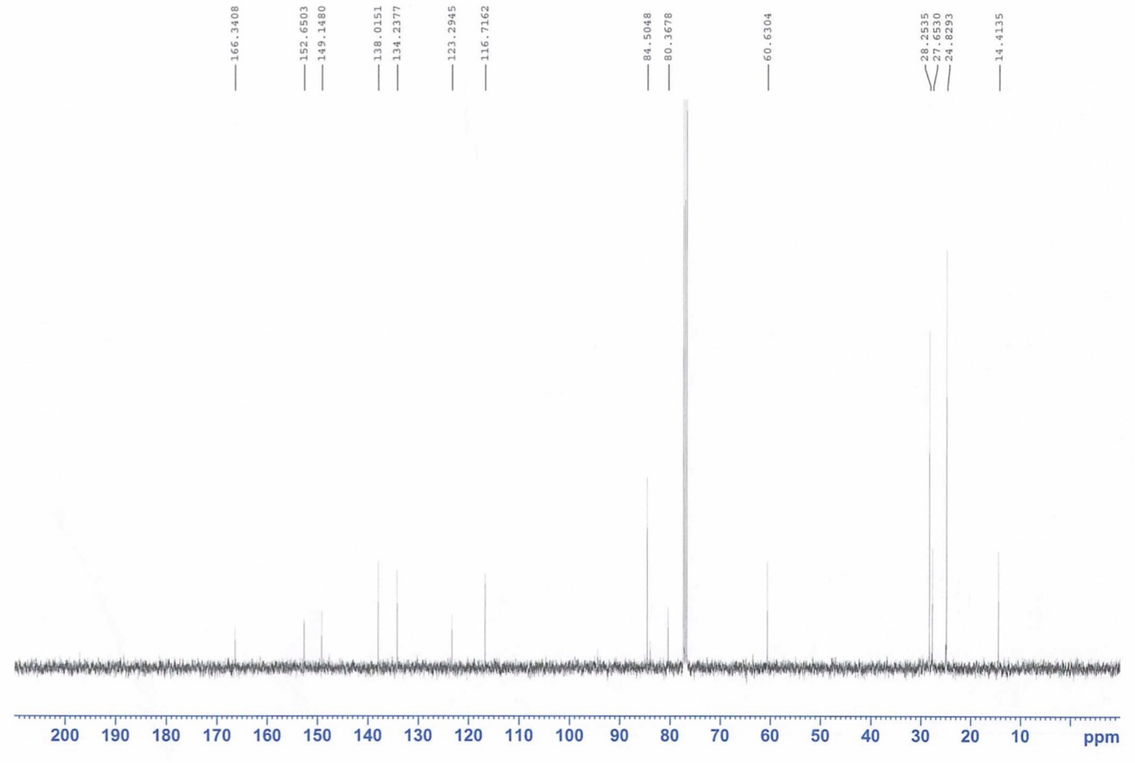

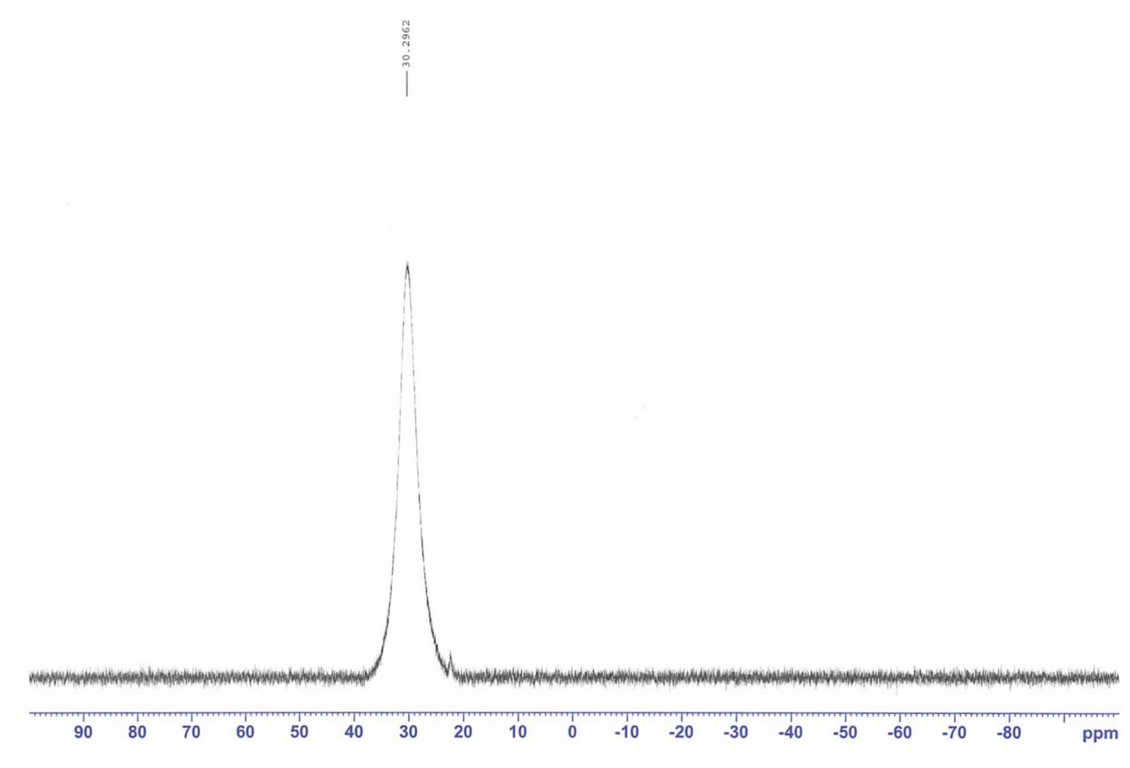
30.2962
90 80 70 60 50 40 30 20 10 0 -10 -20 -30 -40 -50 -60 -70 -80 ppm

^{1}H NMR (400 MHz) and ^{13}C NMR (101 MHz) spectra of (5-((*tert*-butoxycarbonyl)amino)benzo[*d*][1,3]dioxol-4-yl)boronic acid (**2j**) (CD_3OD)

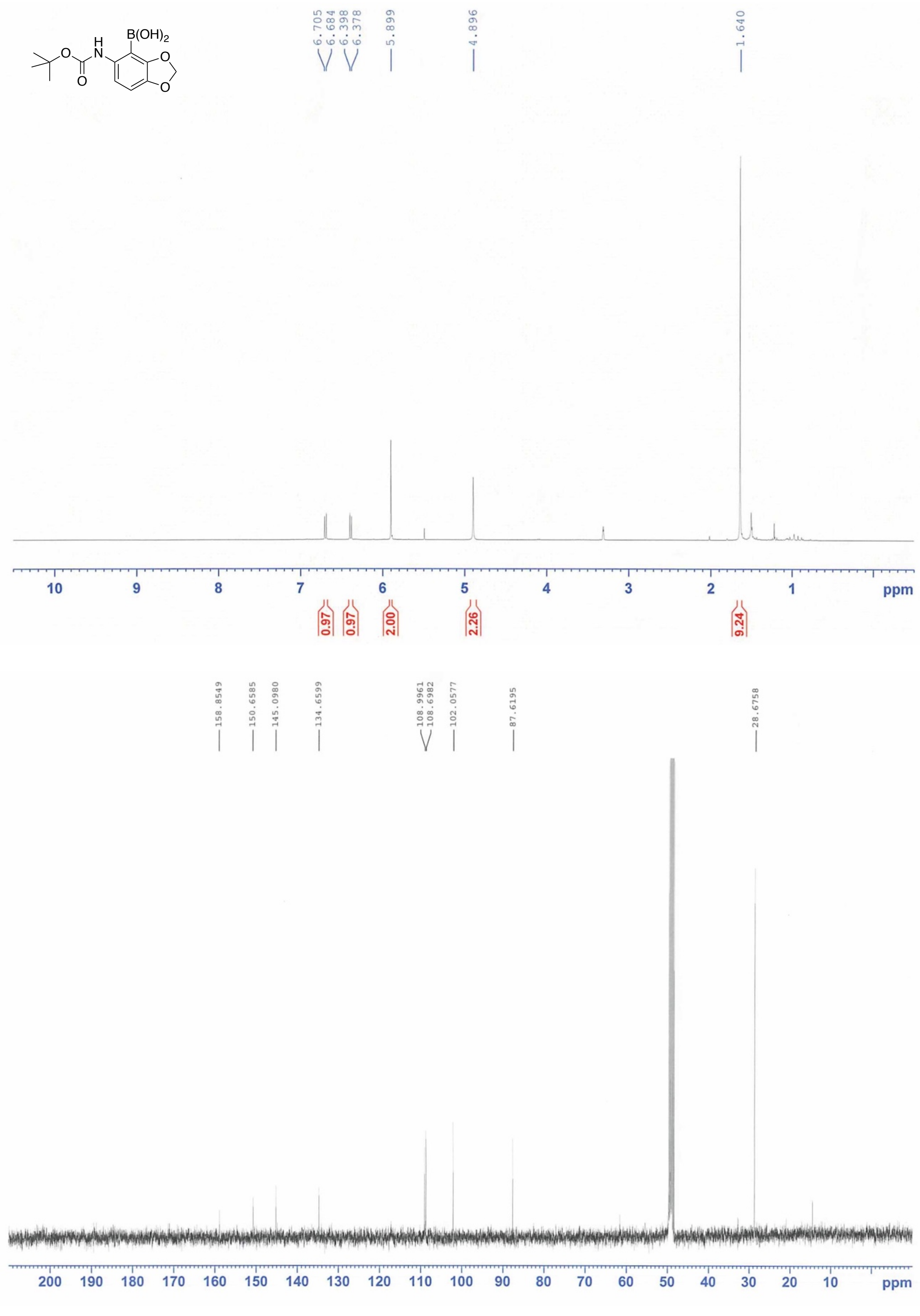

^{1}H NMR (400 MHz) and ^{13}C NMR (101 MHz) spectra of 5-(*tert*-butoxycarbonyl)-6-thiaphenanthridin-6(5*H*)-one (**3a**) ($CDCl_3$)

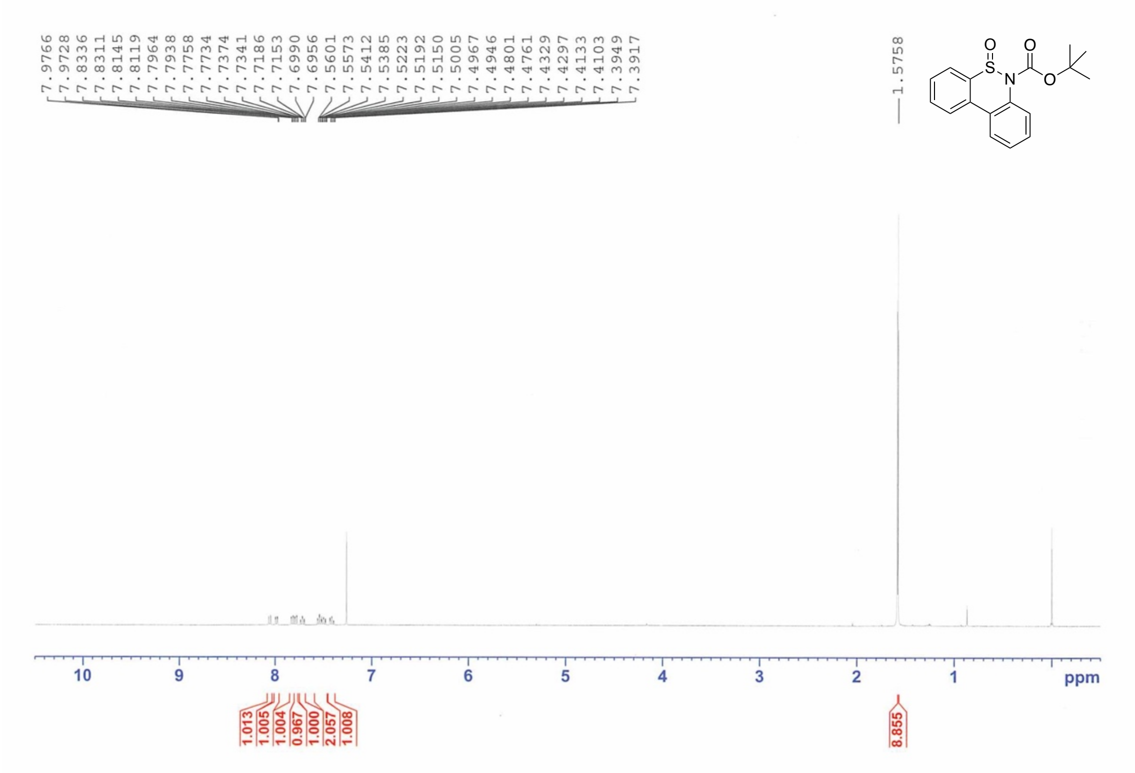

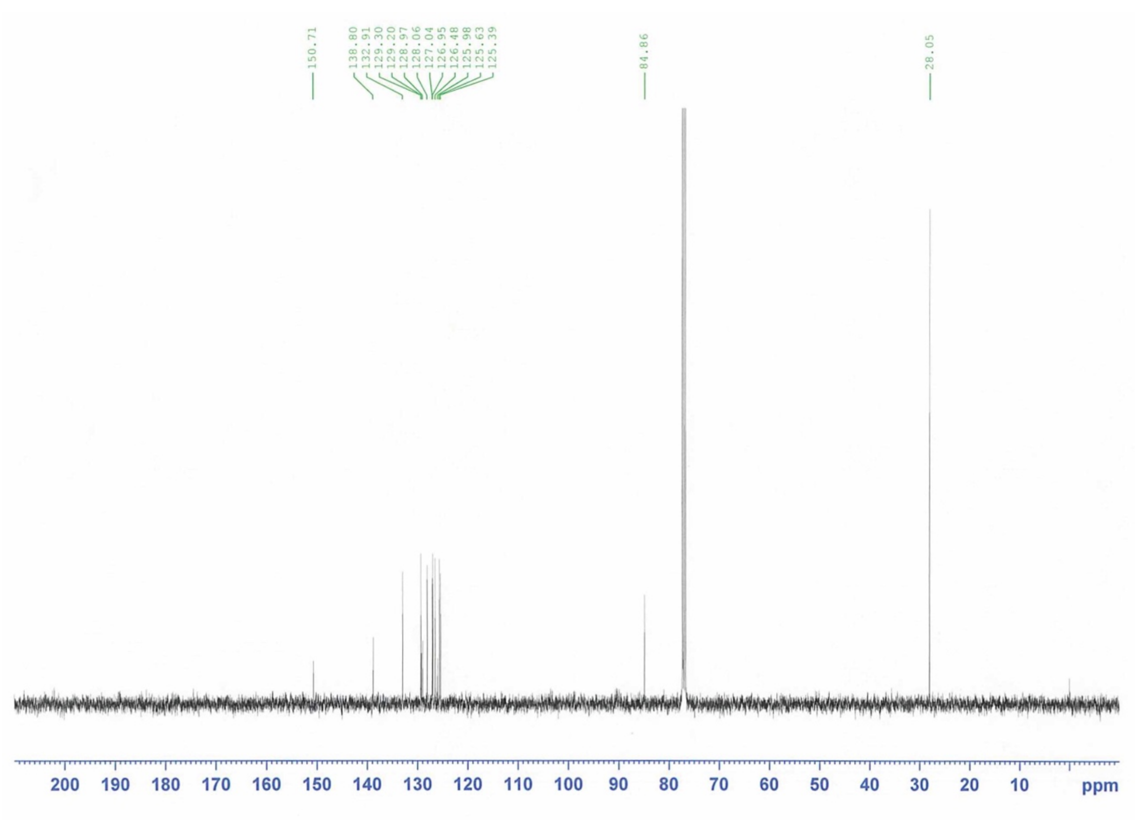

^1H NMR (400 MHz) and ^{13}C NMR (101 MHz) spectra of 5-(*tert*-butoxycarbonyl)-9-methyl-6-thiaphenanthridin-6(5*H*)-one (**3b**) ($CDCl_3$)

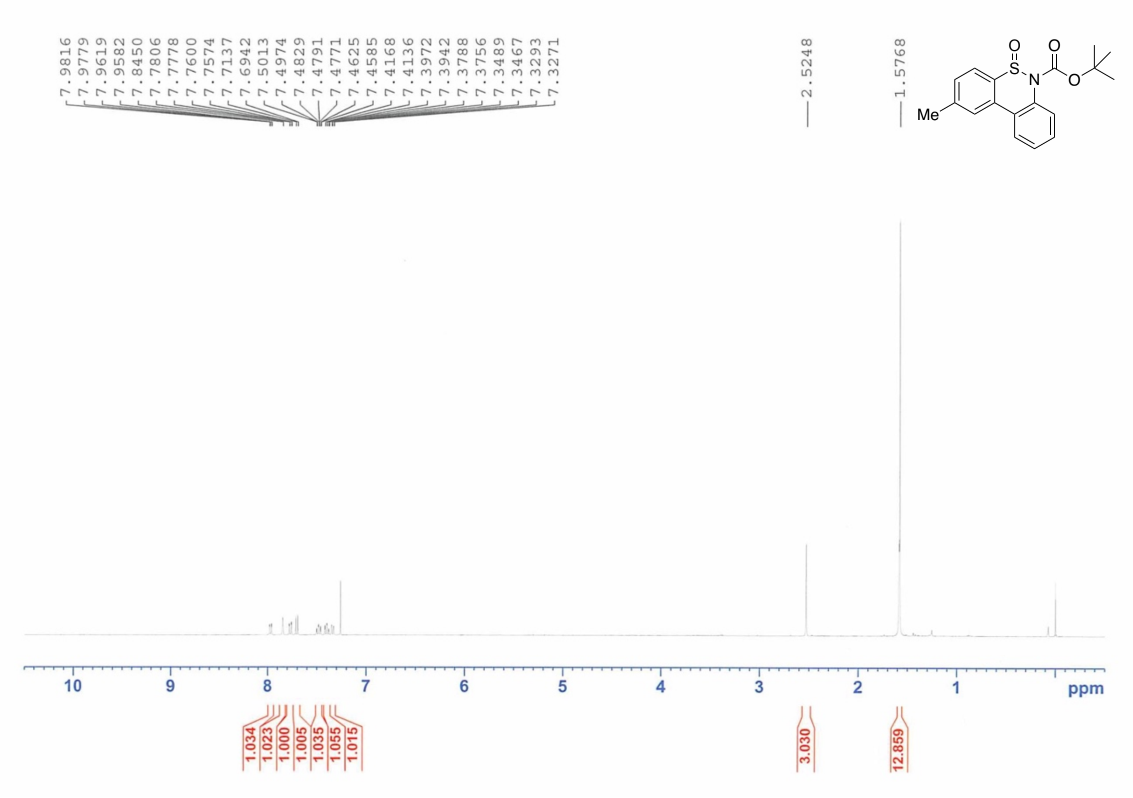

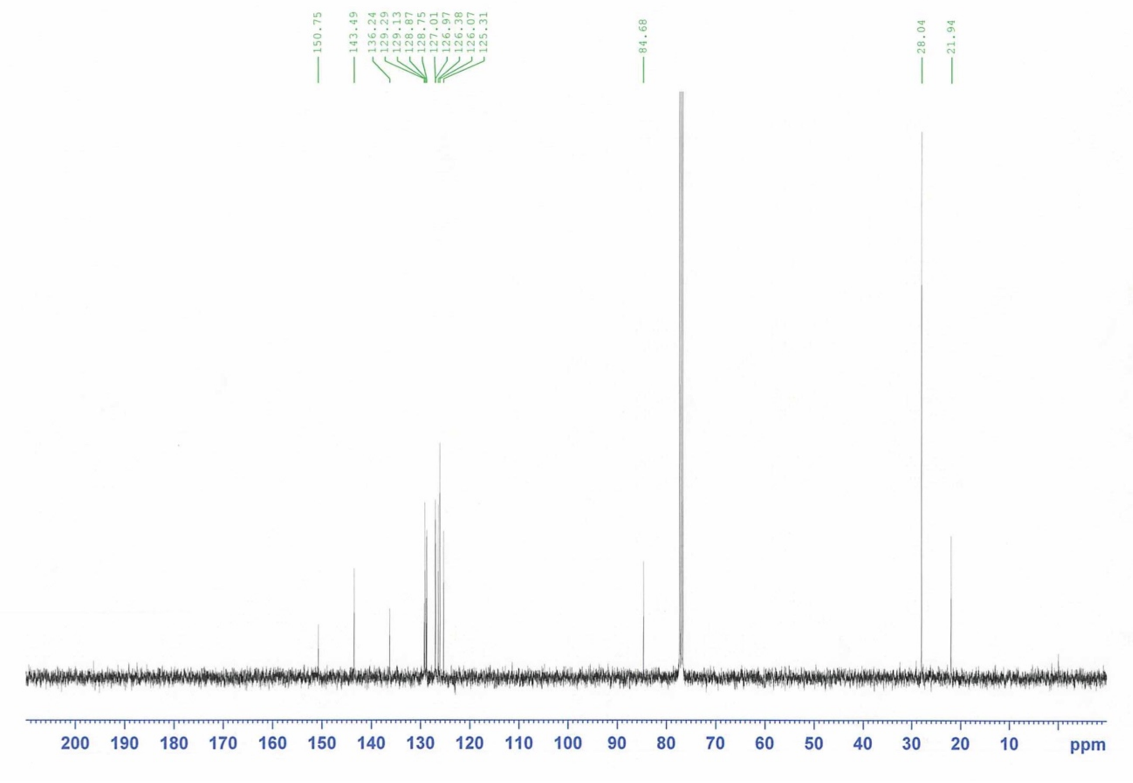

^{1}H NMR (400 MHz) and ^{13}C NMR (101 MHz) spectra of *tert*-butyl 3-methyl-6*H*-dibenzo[*c*,*e*][1,2]thiazine-6-carboxylate 5-oxide (**3c**) ($CDCl_3$)

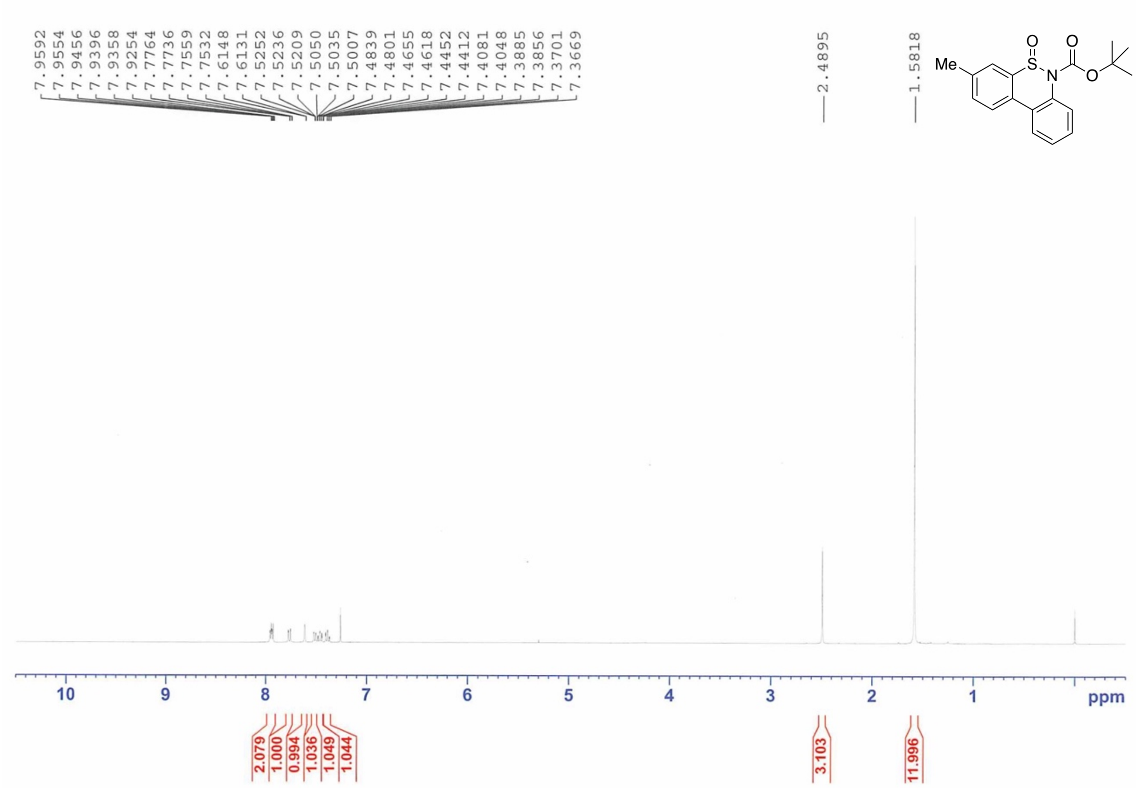

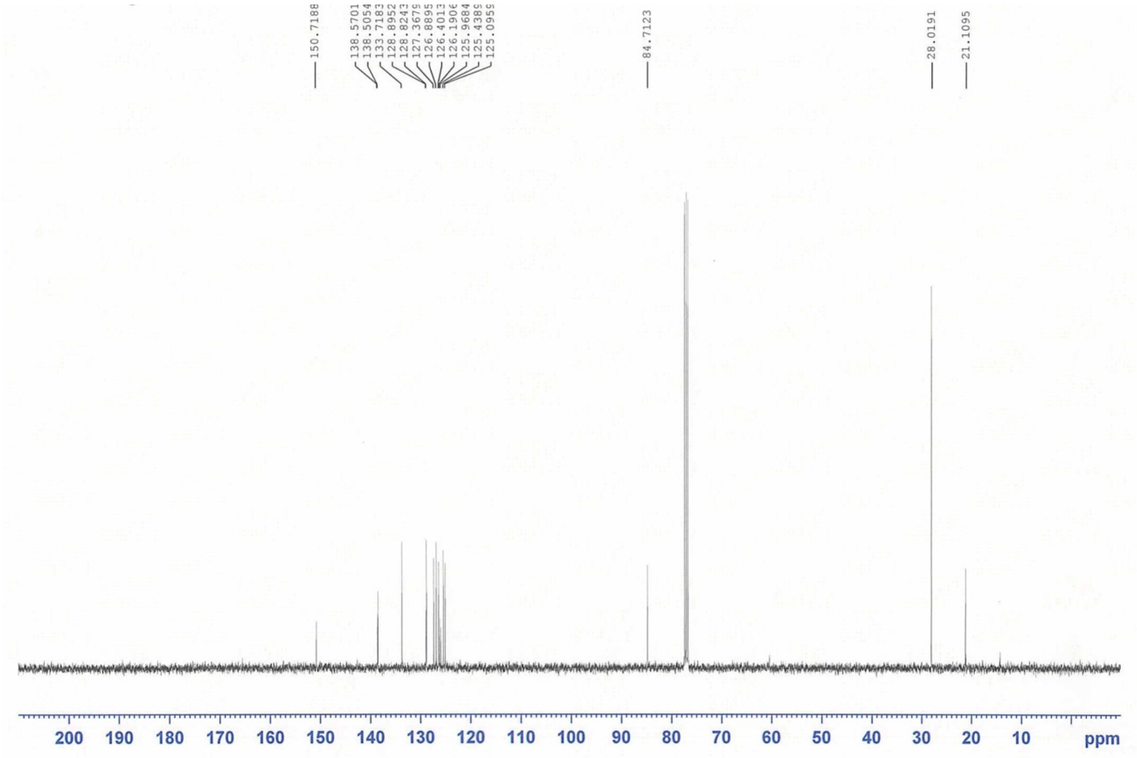

^{1}H NMR (400 MHz) and ^{13}C NMR (101 MHz) spectra of 5-(*tert*-butoxycarbonyl)-7,9-dimethyl-6-thiaphenanthridin-6(5*H*)-one (**3d**) ($CDCl_3$)

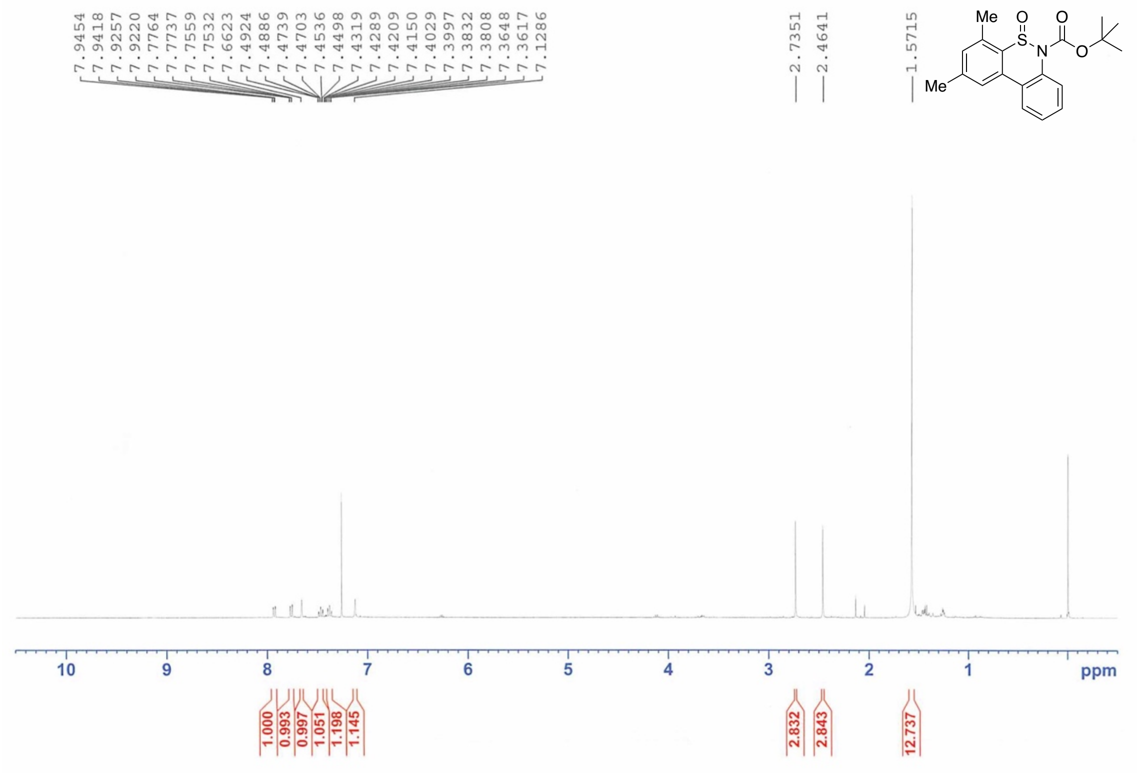

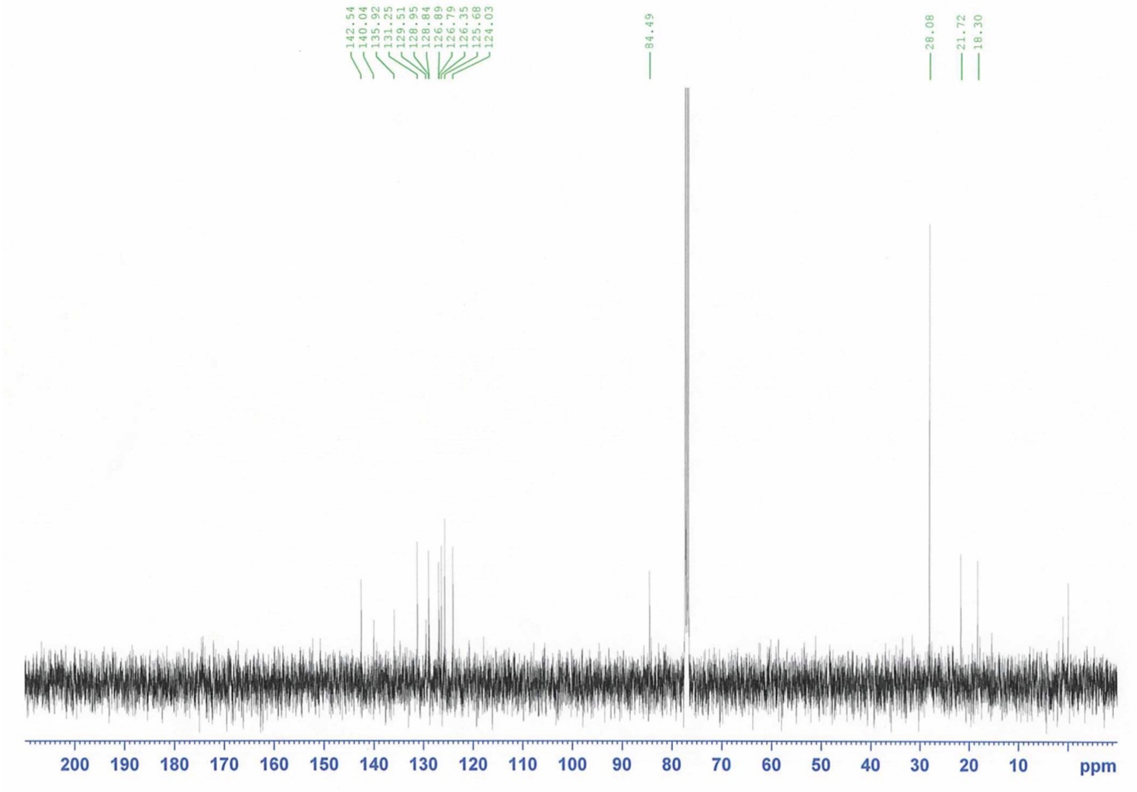

^{1}H NMR (400 MHz) and ^{13}C NMR (101 MHz) spectra of *tert*-butyl 2-methoxy-6*H*-dibenzo[*c*,*e*][1,2]thiazine-6-carboxylate 5-oxide (**3e**) ($CDCl_3$)

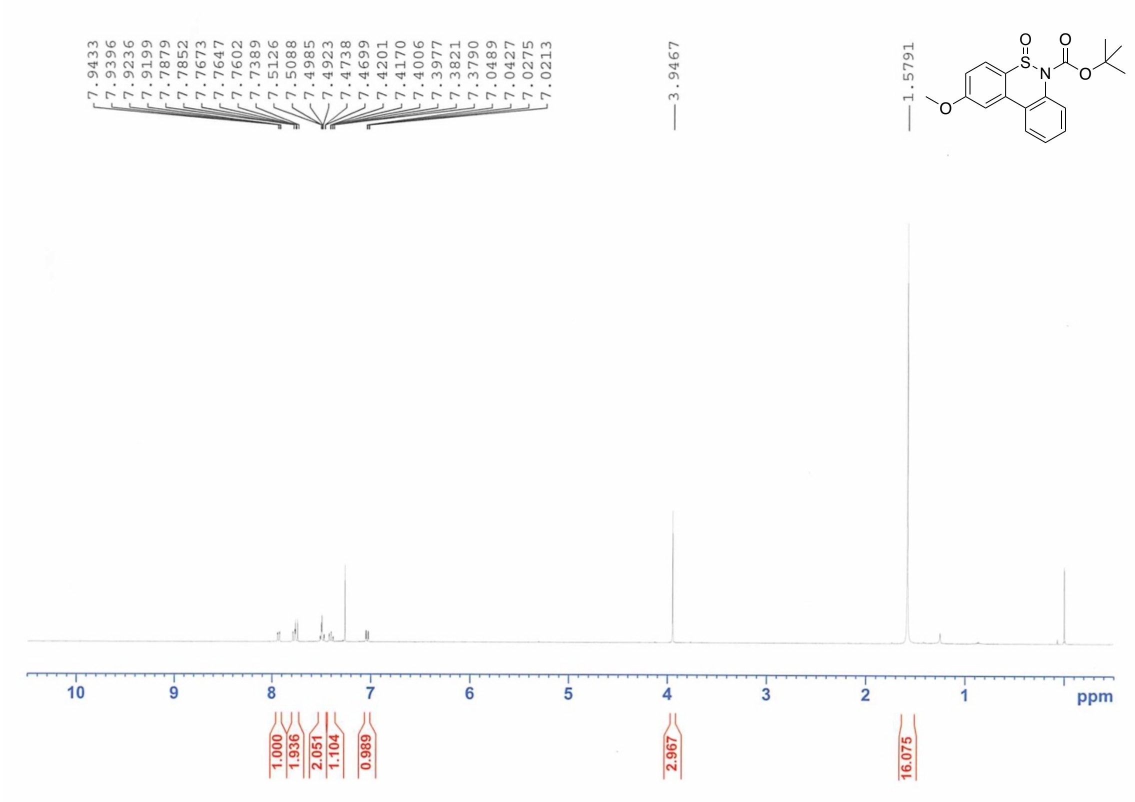

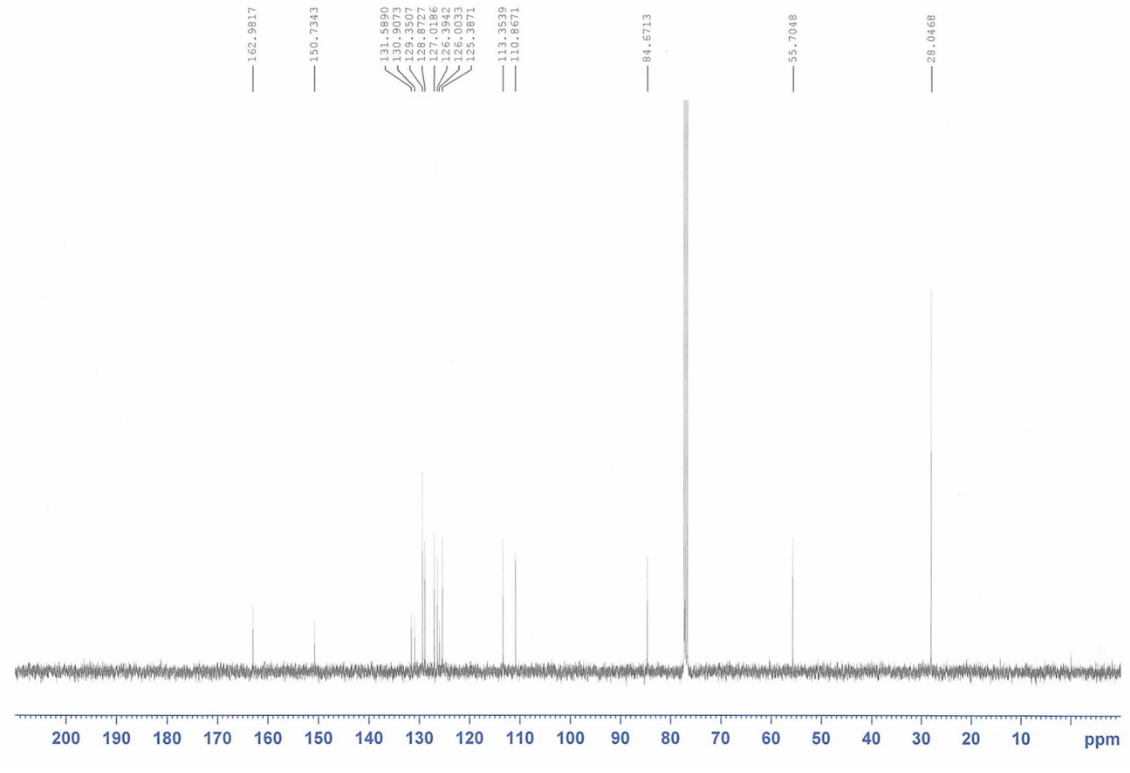

^{1}H NMR (400 MHz), ^{13}C NMR (101 MHz), and ^{19}F NMR (376 MHz) spectra of *tert*-butyl 2-(4-fluorophenyl)-6*H*-dibenzo[*c*,*e*][1,2]thiazine-6-carboxylate 5-oxide (**3f**) ($CDCl_3$)

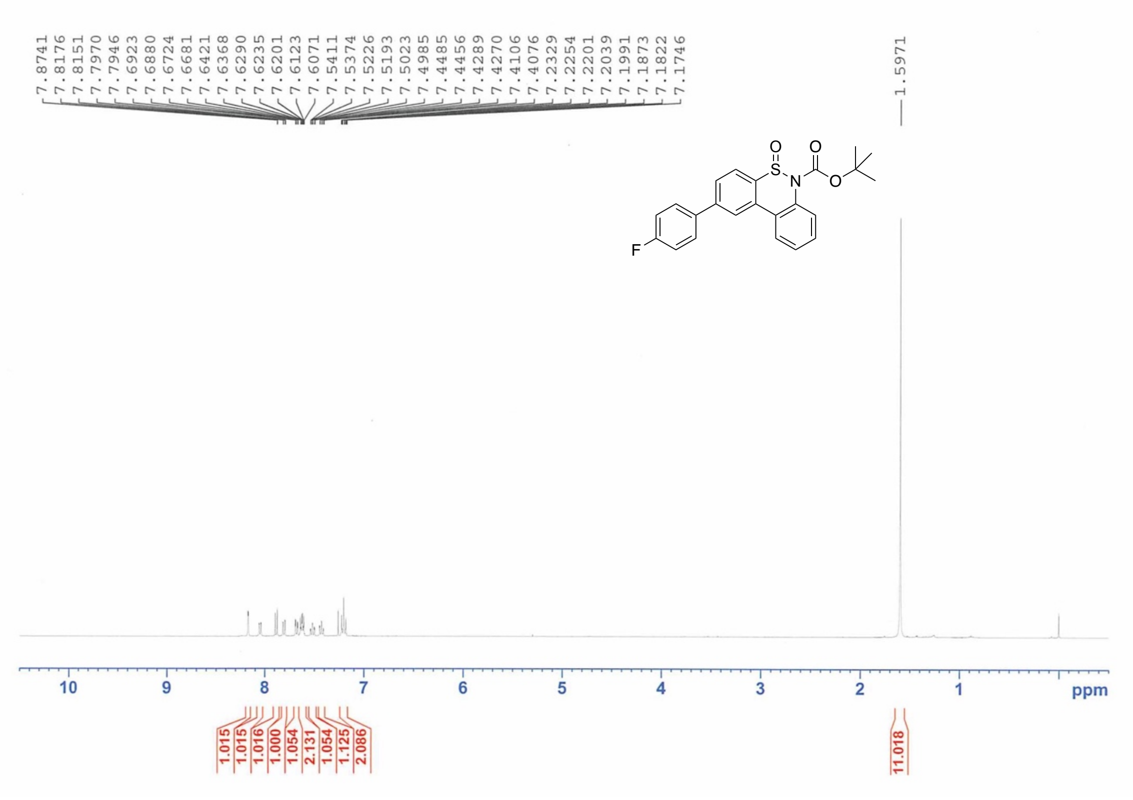

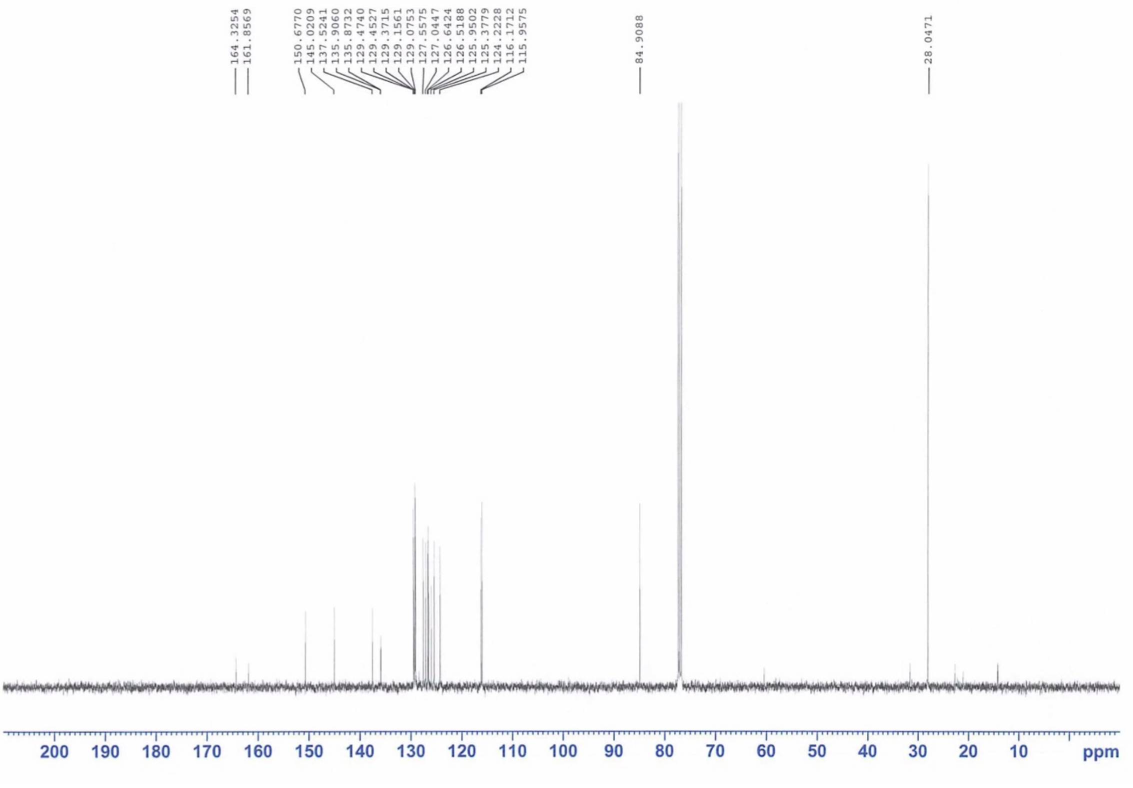

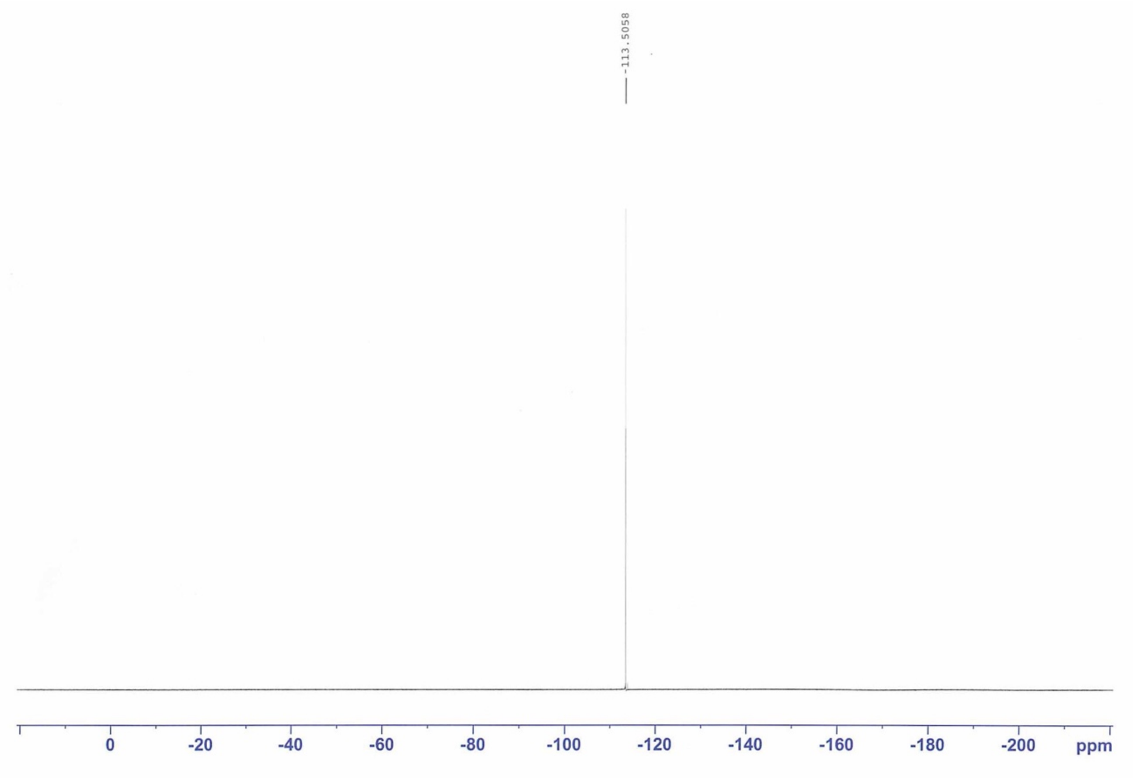
-113.5058
0
-20
-40
-60
-80
-100
-120
-140
-160
-180
-200
ppm

^{1}H NMR (400 MHz) and ^{13}C NMR (101 MHz) spectra of *tert*-butyl 2-(4-chlorophenyl)-6*H*-dibenzo[*c*,*e*][1,2]thiazine-6-carboxylate 5-oxide (**3g**) ($CDCl_3$)

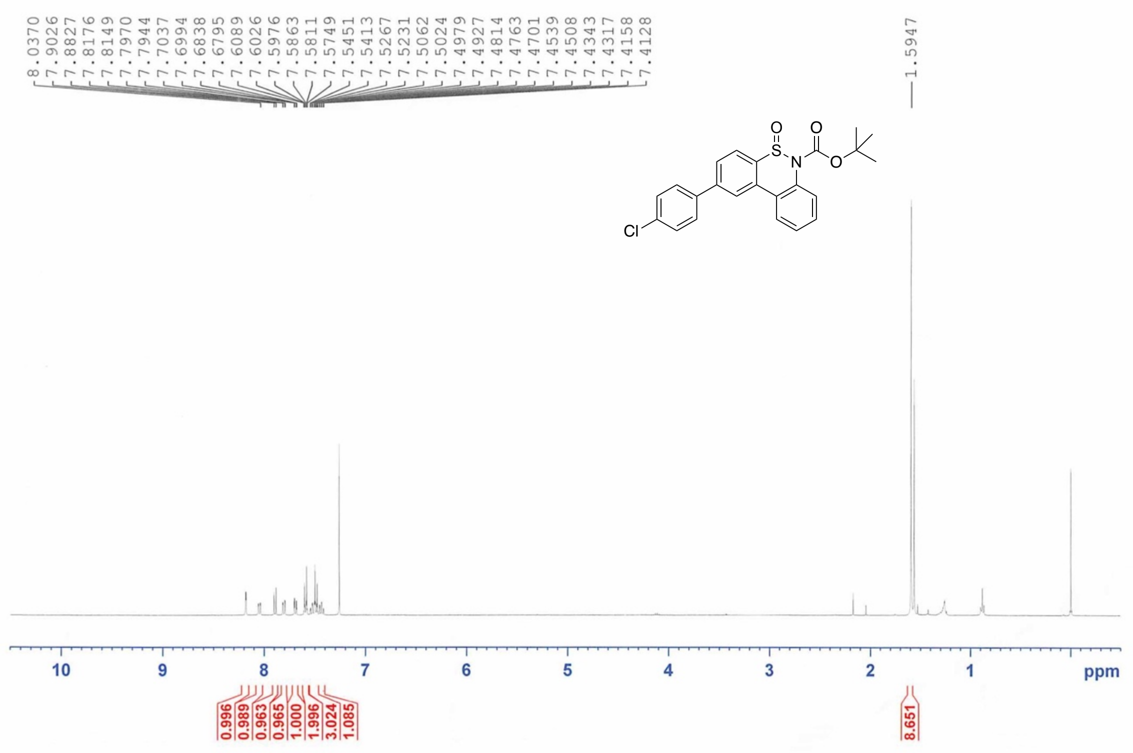

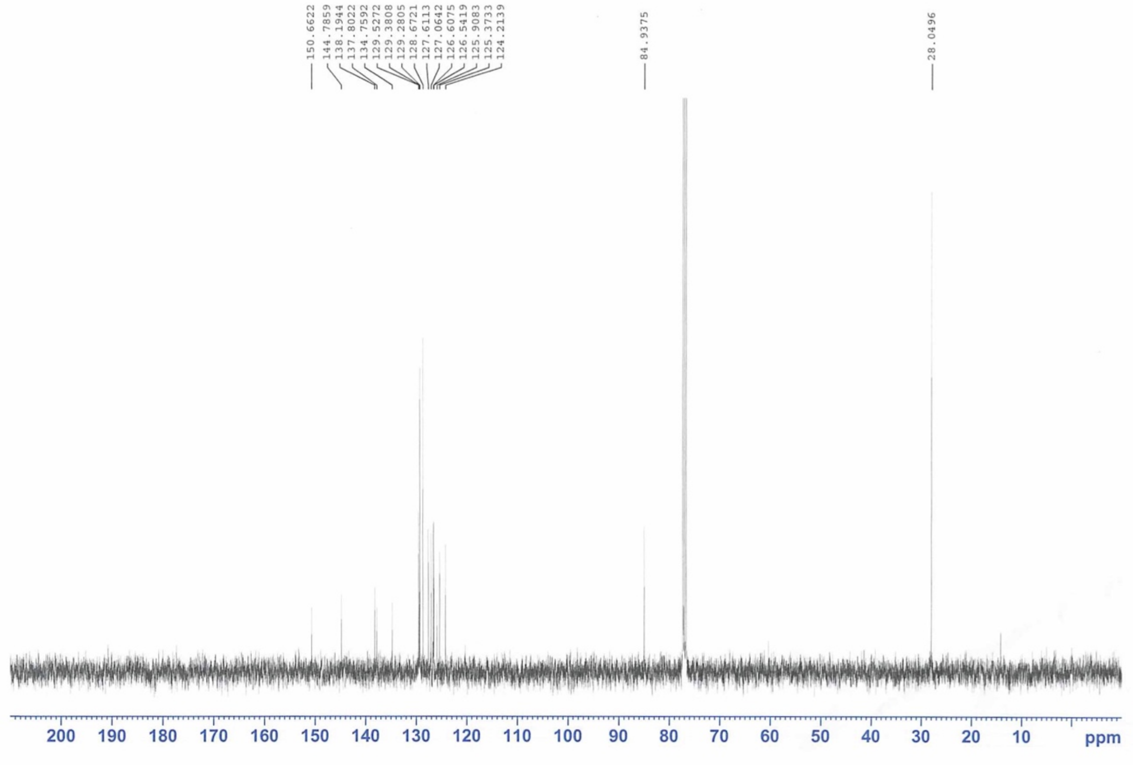

^{1}H NMR (400 MHz) and ^{13}C NMR (101 MHz) spectra of *tert*-butyl 2-chloro-6*H*-dibenzo[*c*,*e*][1,2]thiazine-6-carboxylate 5-oxide (**3h**) ($CDCl_3$)

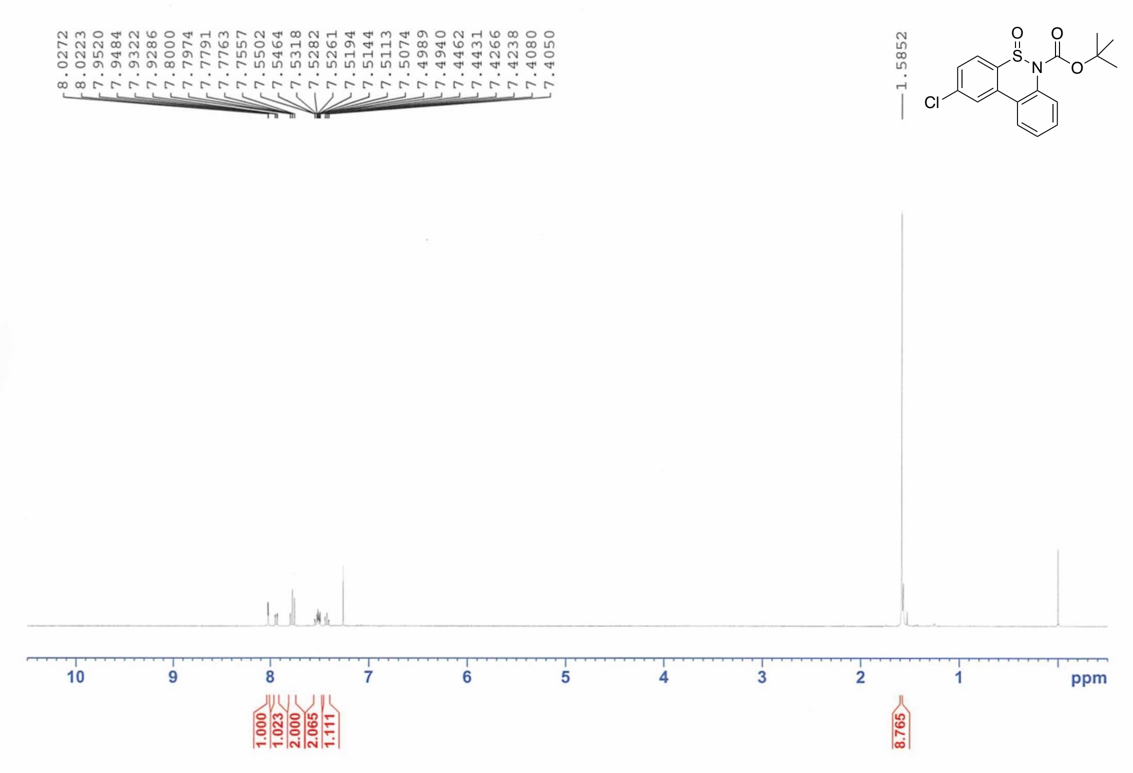

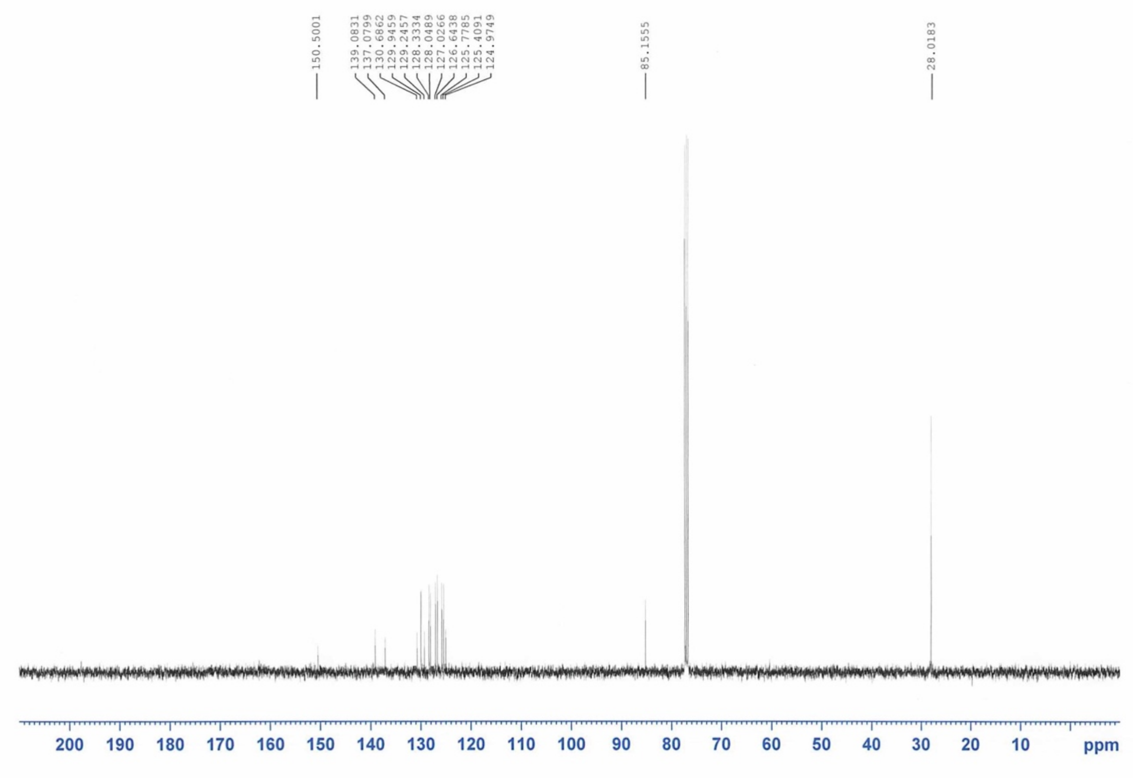

^{1}H NMR (400 MHz) and ^{13}C NMR (101 MHz) spectra of 6-(*tert*-butyl) 2-ethyl 6*H*-dibenzo[*c*,*e*][1,2]thiazine-2,6-dicarboxylate 5-oxide (**3i**) ($CDCl_3$)

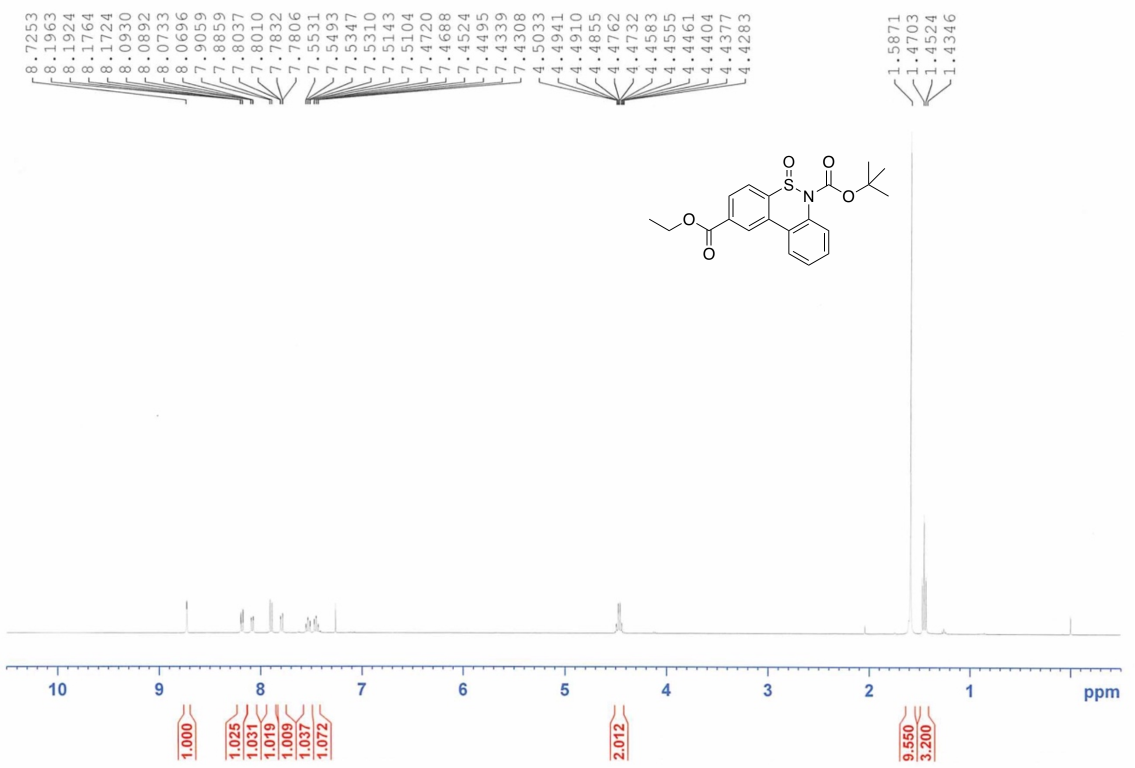

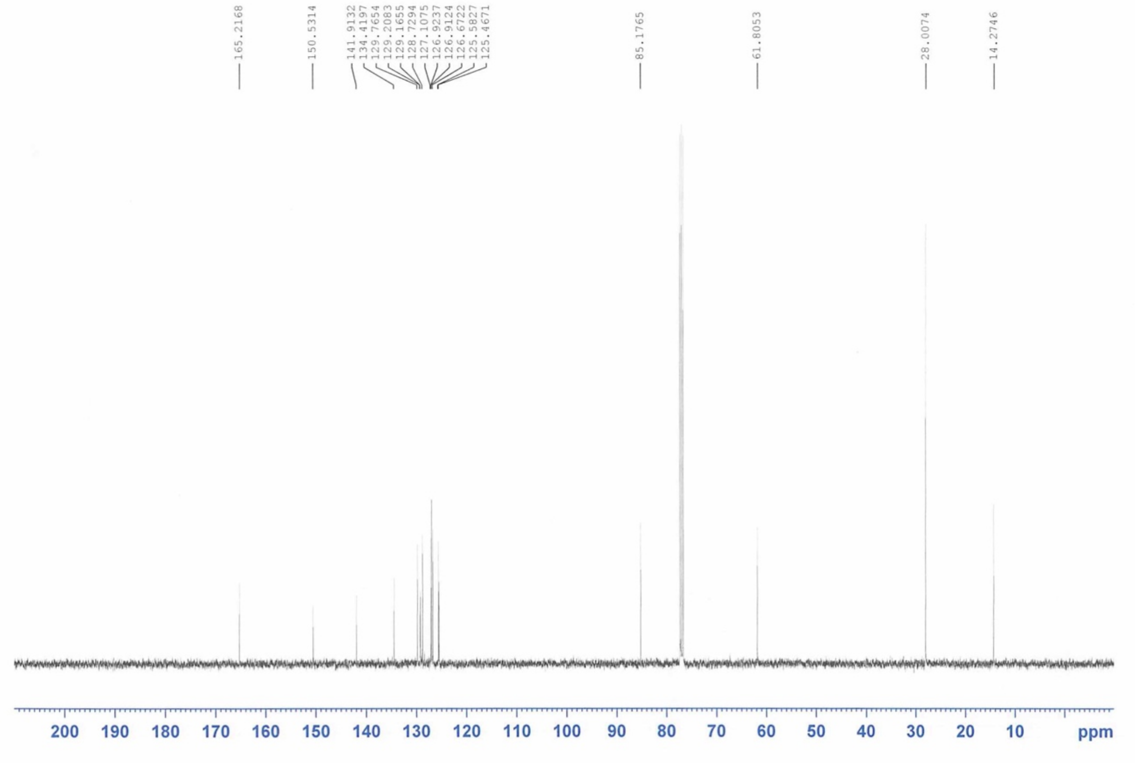

^{1}H NMR (400 MHz), ^{13}C NMR (101 MHz), and ^{19}F NMR (376 MHz) spectra of *tert*-butyl 2-(trifluoromethyl)-6*H*-dibenzo[*c*,*e*][1,2]thiazine-6-carboxylate 5-oxide (**3j**) ($CDCl_3$)

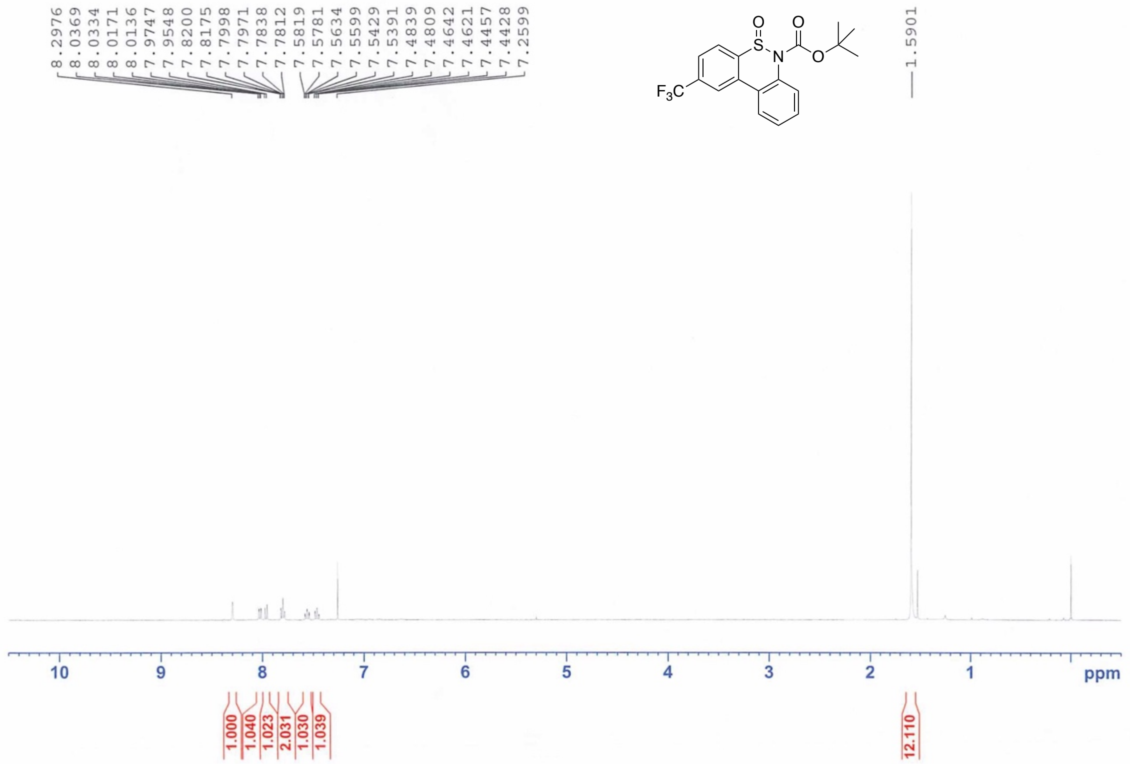

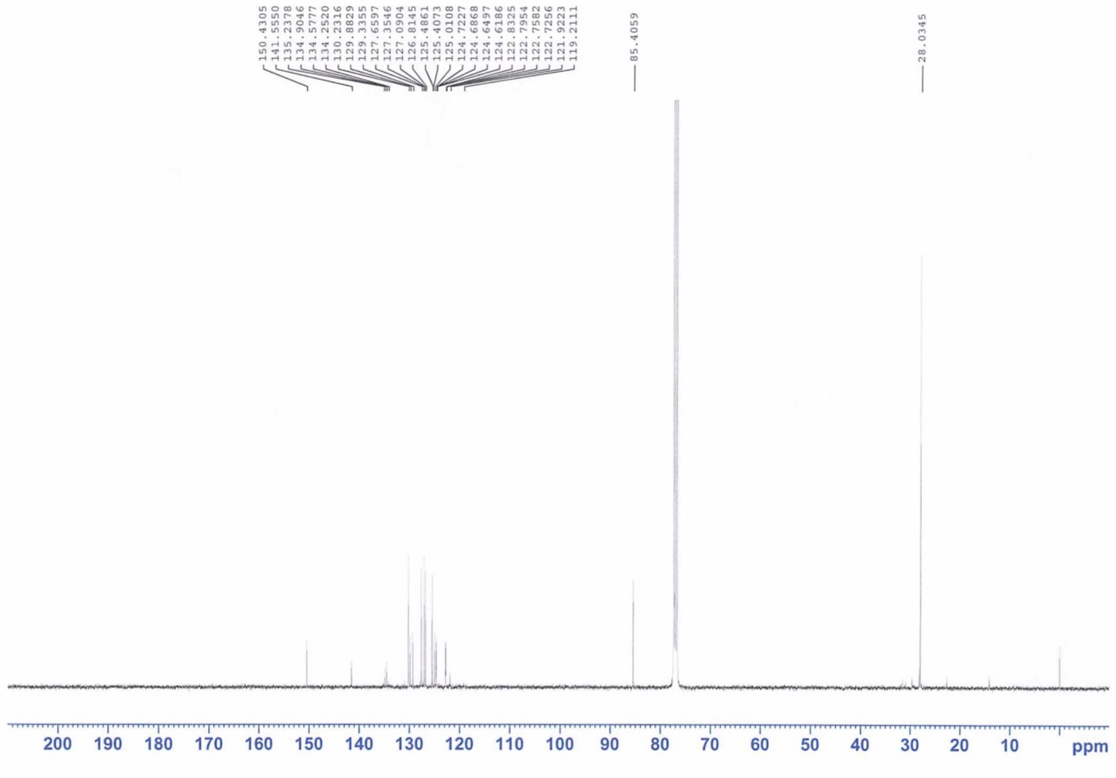

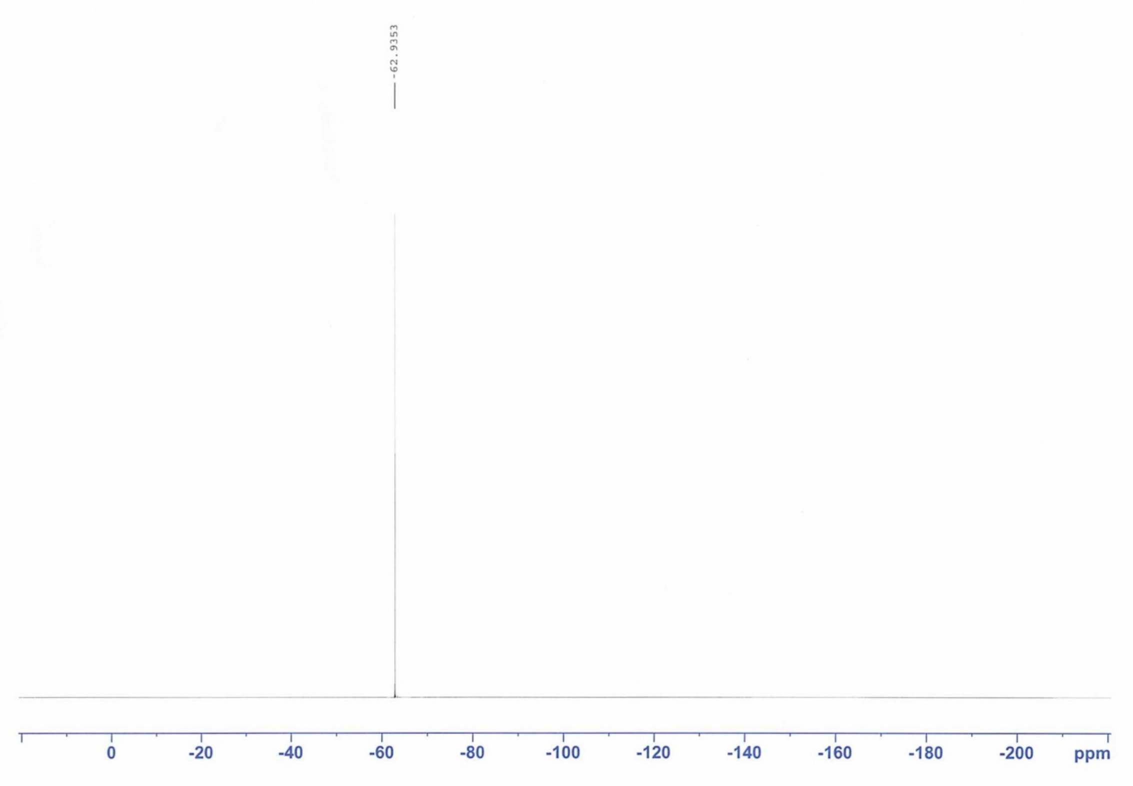
-62.9353
0
-20
-40
-60
-80
-100
-120
-140
-160
-180
-200
ppm

^{1}H NMR (400 MHz) and ^{13}C NMR (101 MHz) spectra of 5-(*tert*-butoxycarbonyl)-3-methyl-6-thiaphenanthridin-6(5*H*)-one (**3k**) ($CDCl_3$)

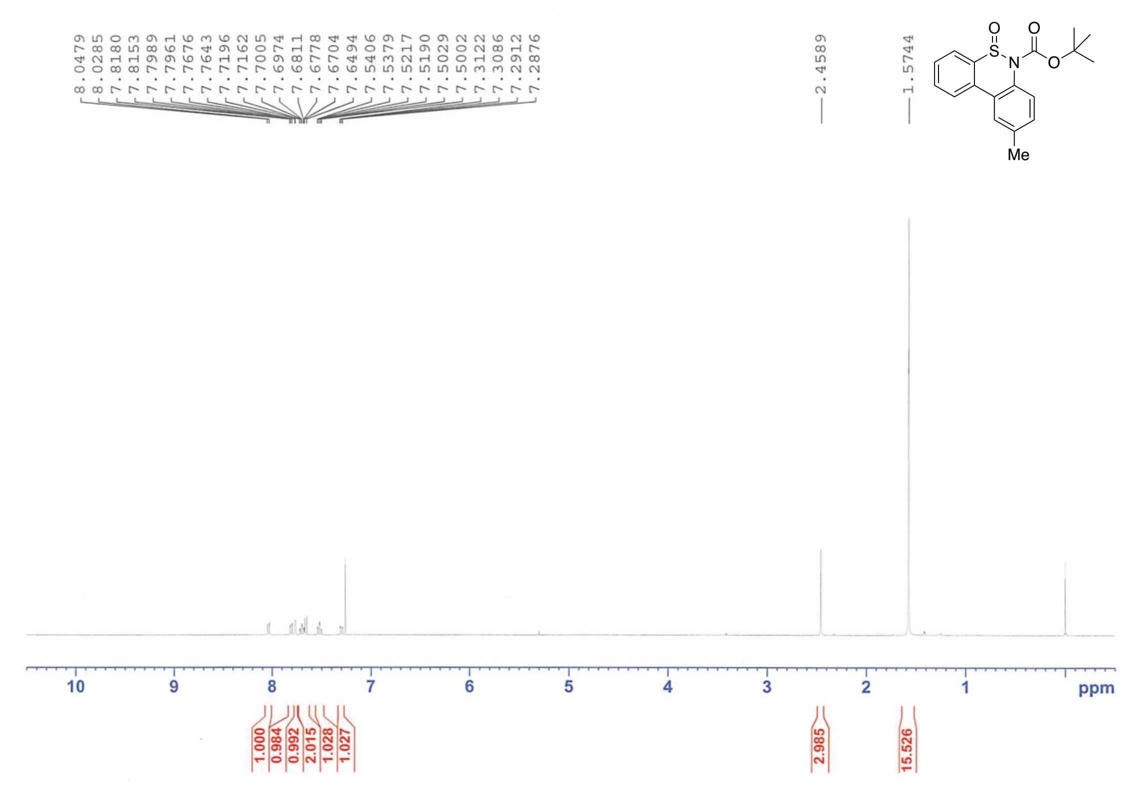

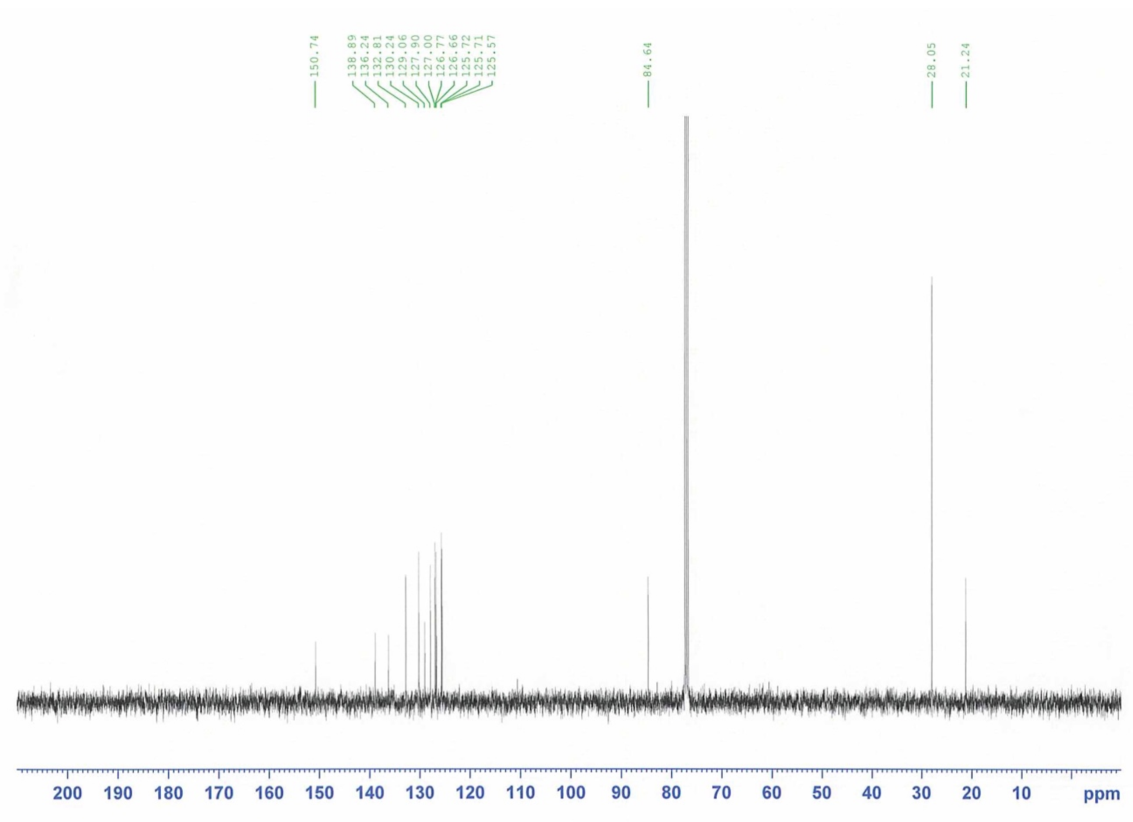

^{1}H NMR (400 MHz) and ^{13}C NMR (101 MHz) spectra of 5-(*tert*-butoxycarbonyl)-3-methoxy-6-thiaphenanthridin-6(5*H*)-one (**3l**) ($CDCl_3$)

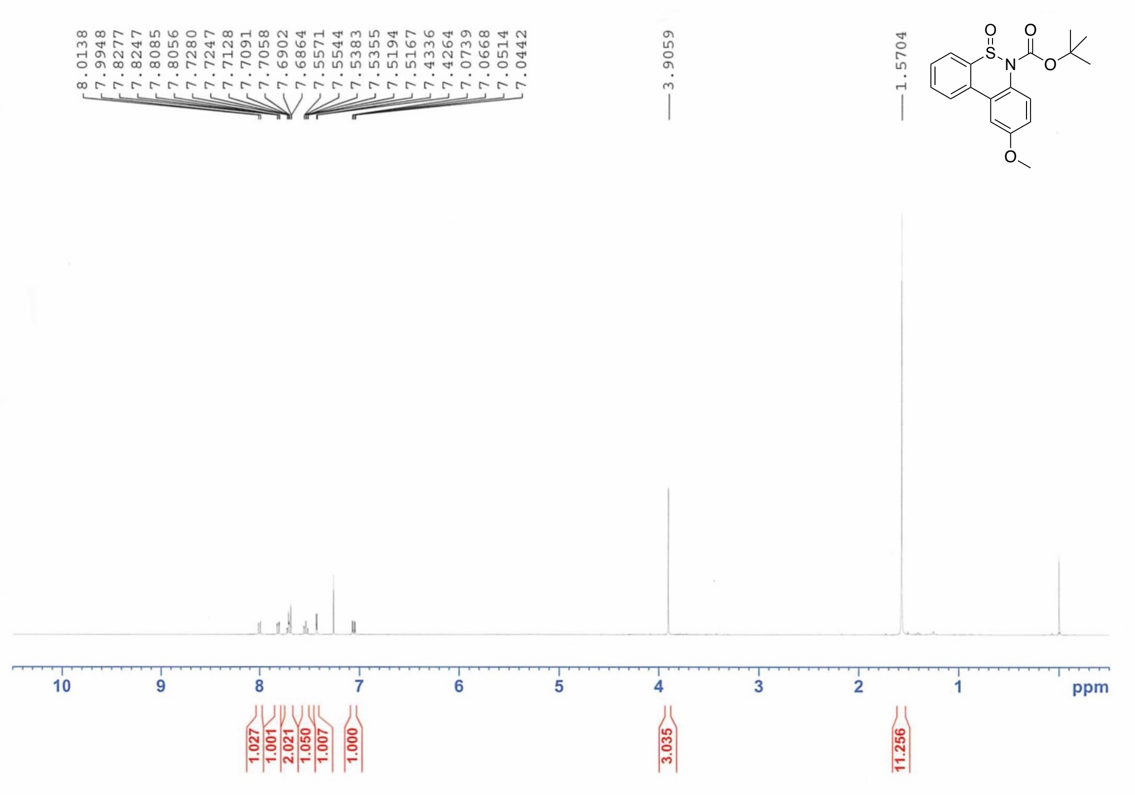

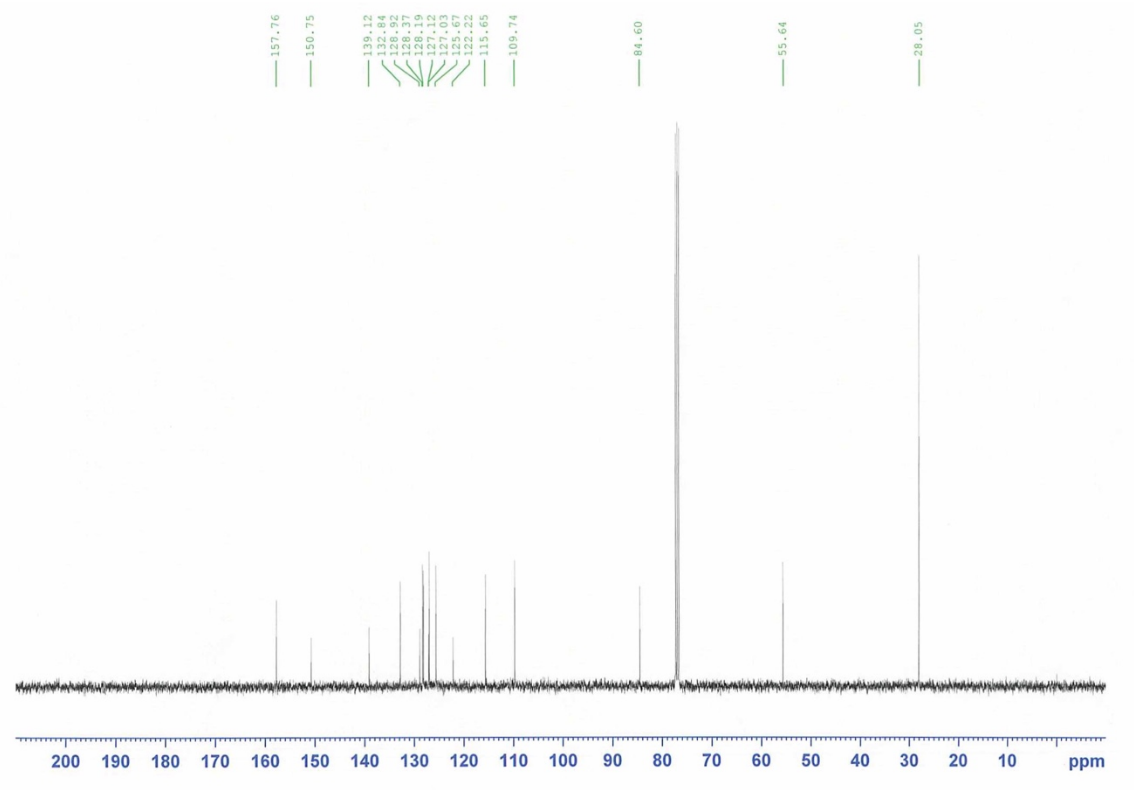

^{1}H NMR (400 MHz) and ^{13}C NMR (101 MHz) spectra of 5-(*tert*-butoxycarbonyl)-3-(4-tolyl)-6-thiaphenanthridin-6(5*H*)-one (**3m**) ($CDCl_3$)

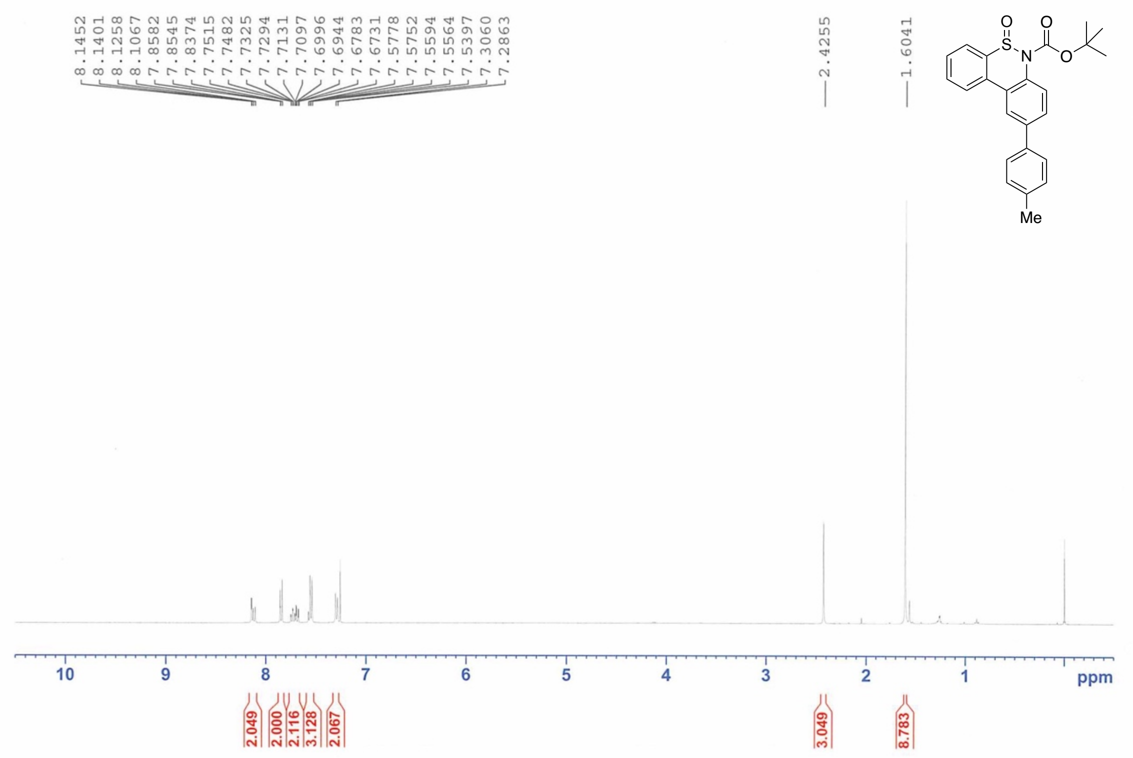

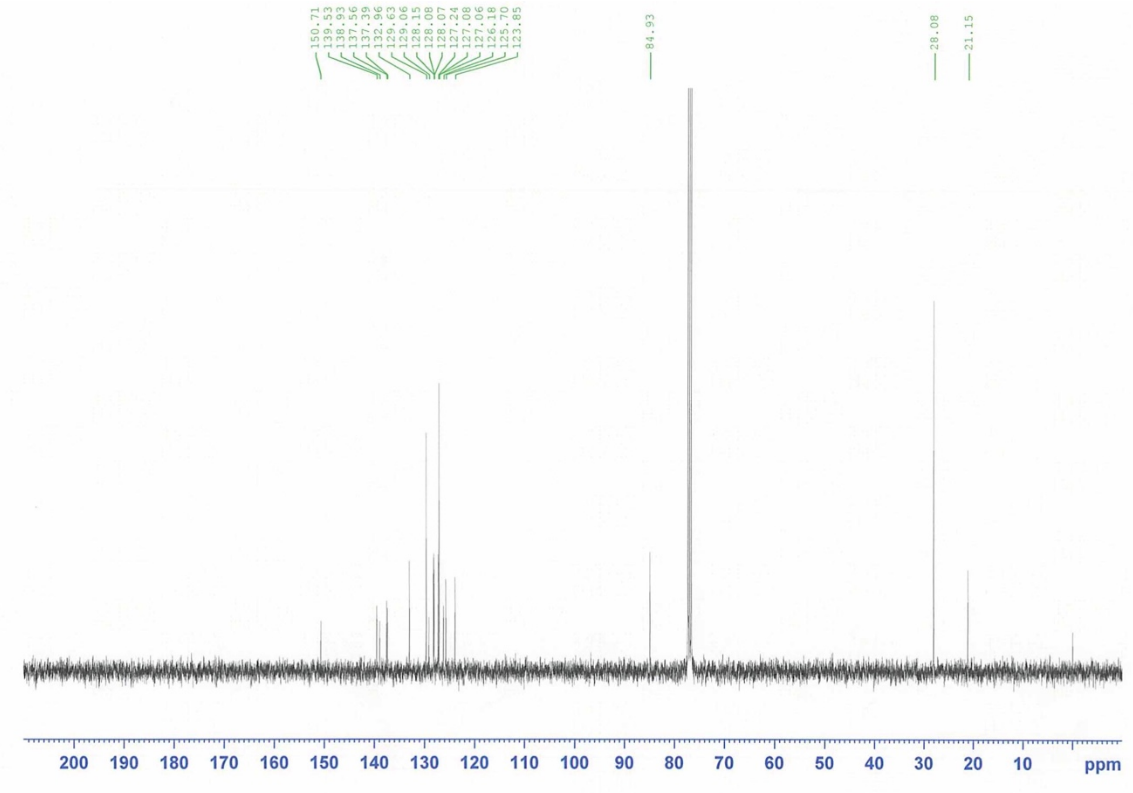

^{1}H NMR (400 MHz) and ^{13}C NMR (101 MHz) spectra of 5-(*tert*-butoxycarbonyl)-3-chloro-6-thiaphenanthridin-6(5*H*)-one (**3n**) ($CDCl_3$)

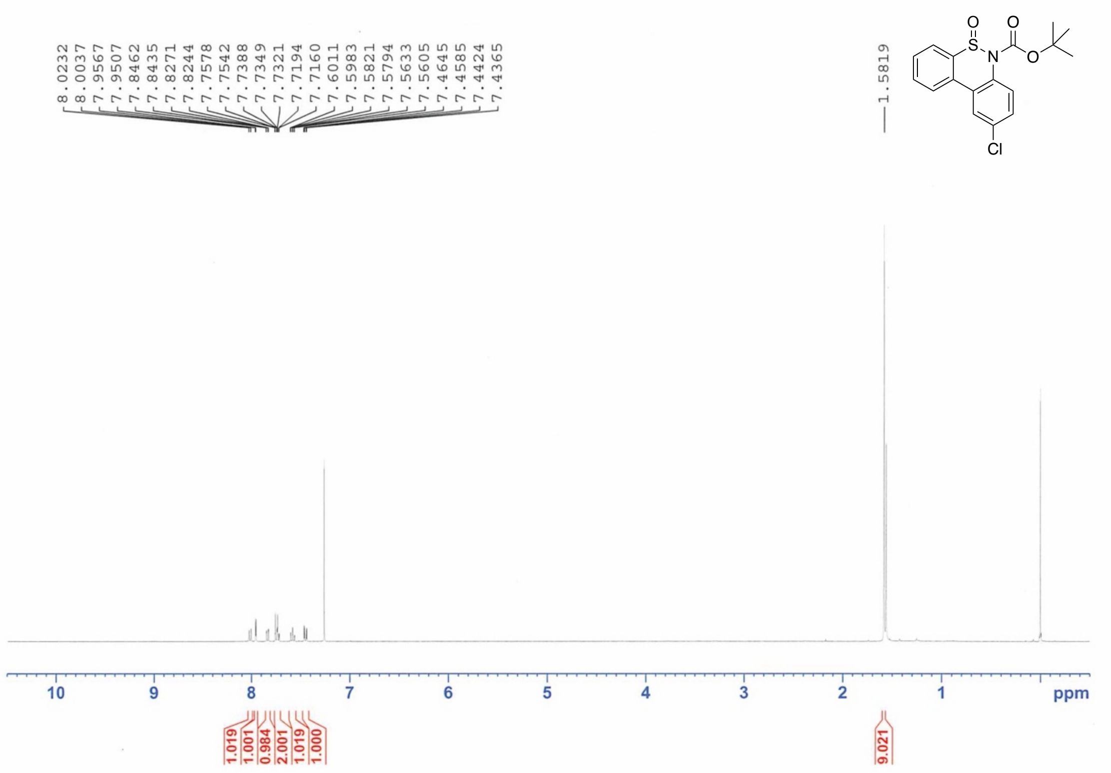

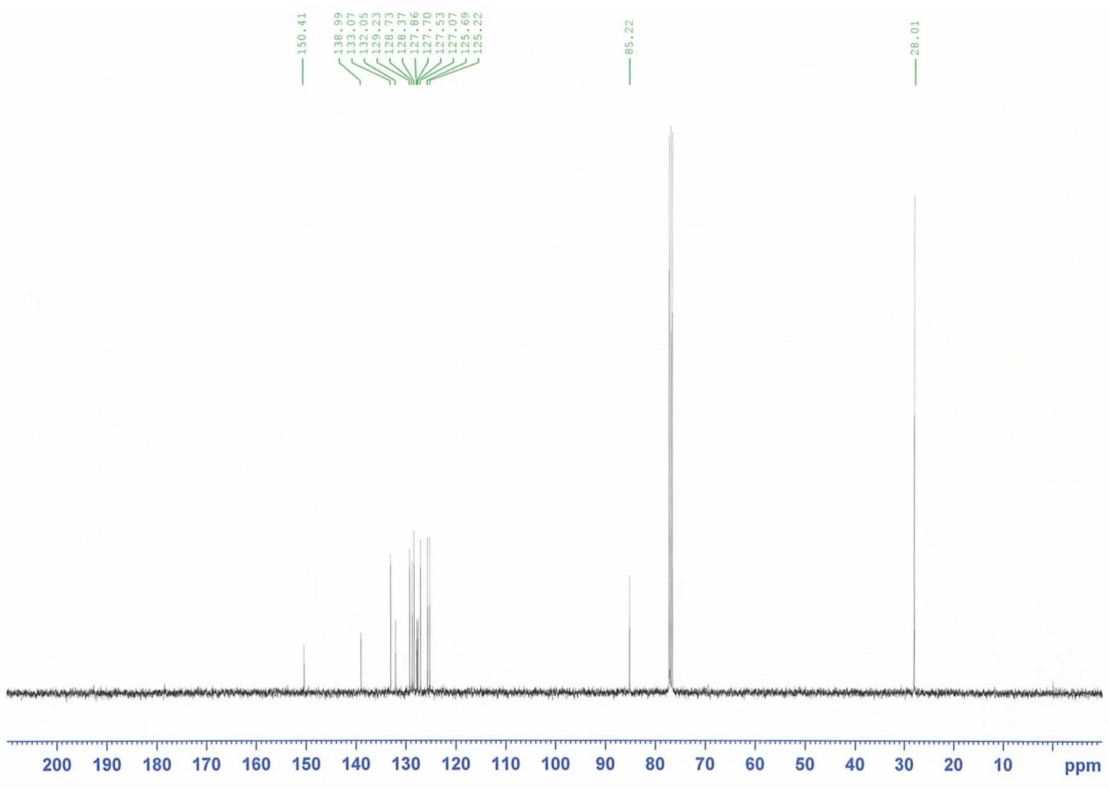

^{1}H NMR (400 MHz), ^{13}C NMR (101 MHz), and ^{19}F NMR (376 MHz) spectra of *tert*-butyl 9-fluoro-6*H*-dibenzo[*c*,*e*][1,2]thiazine-6-carboxylate 5-oxide (**3o**) ($CDCl_3$)

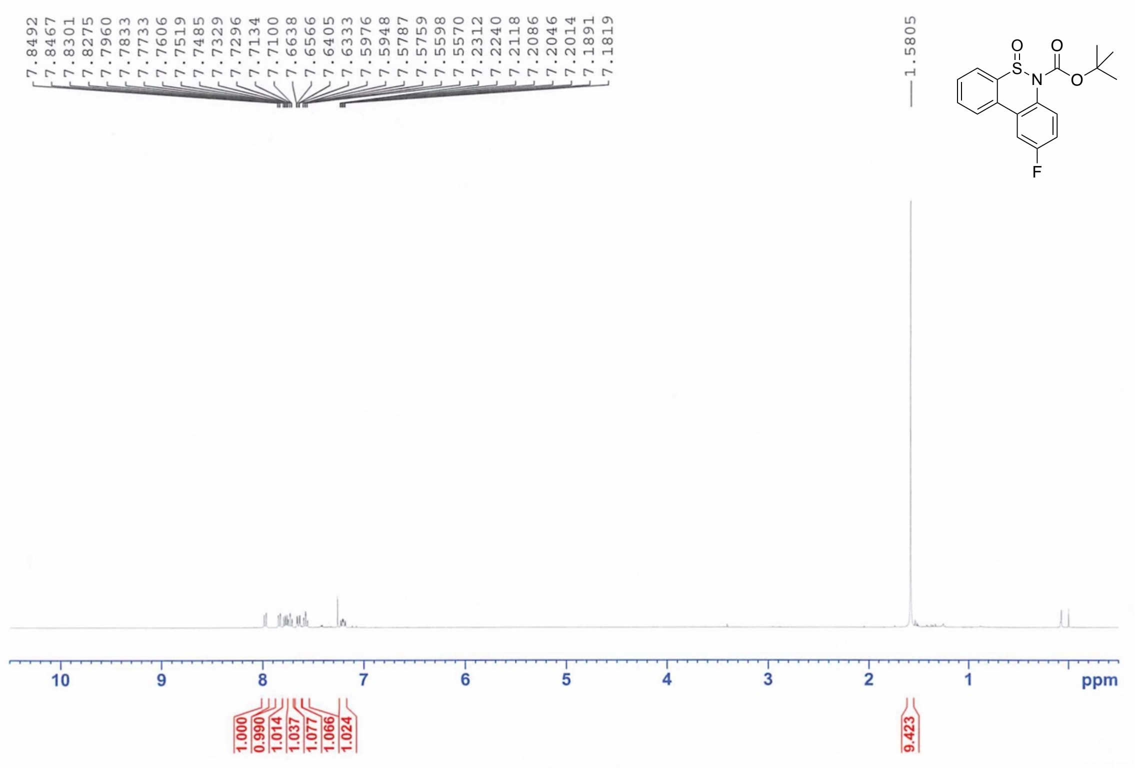

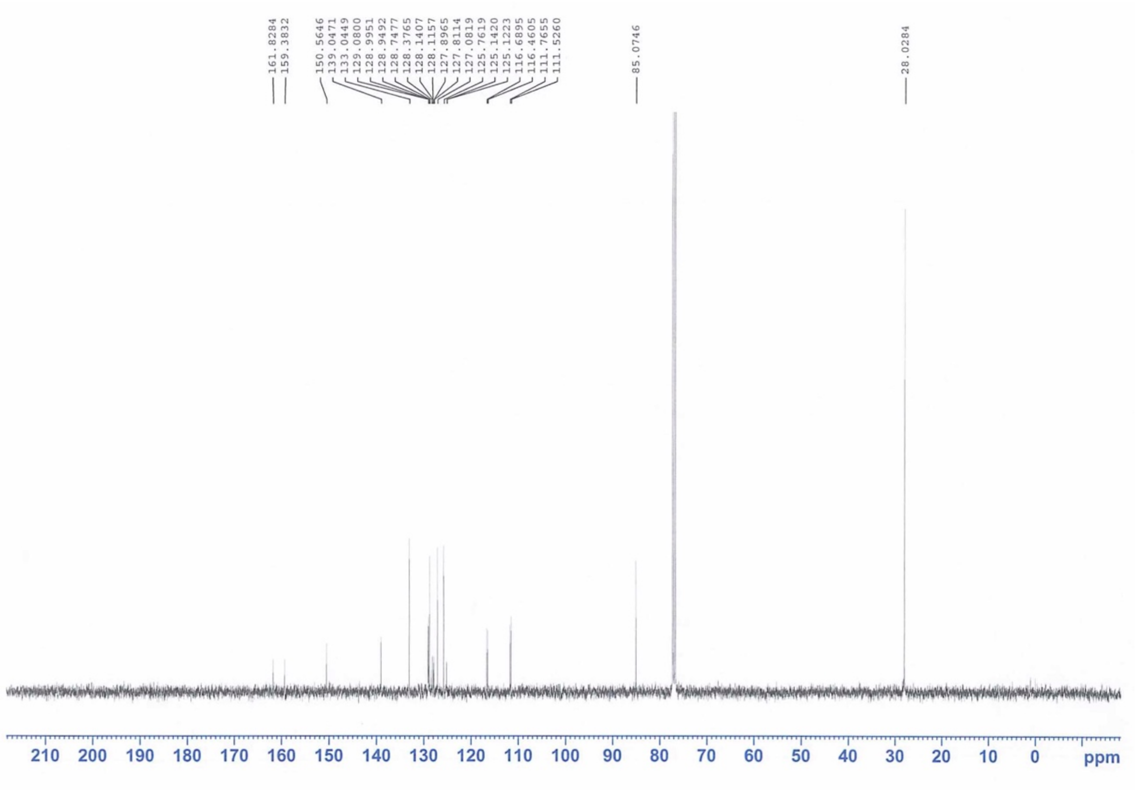

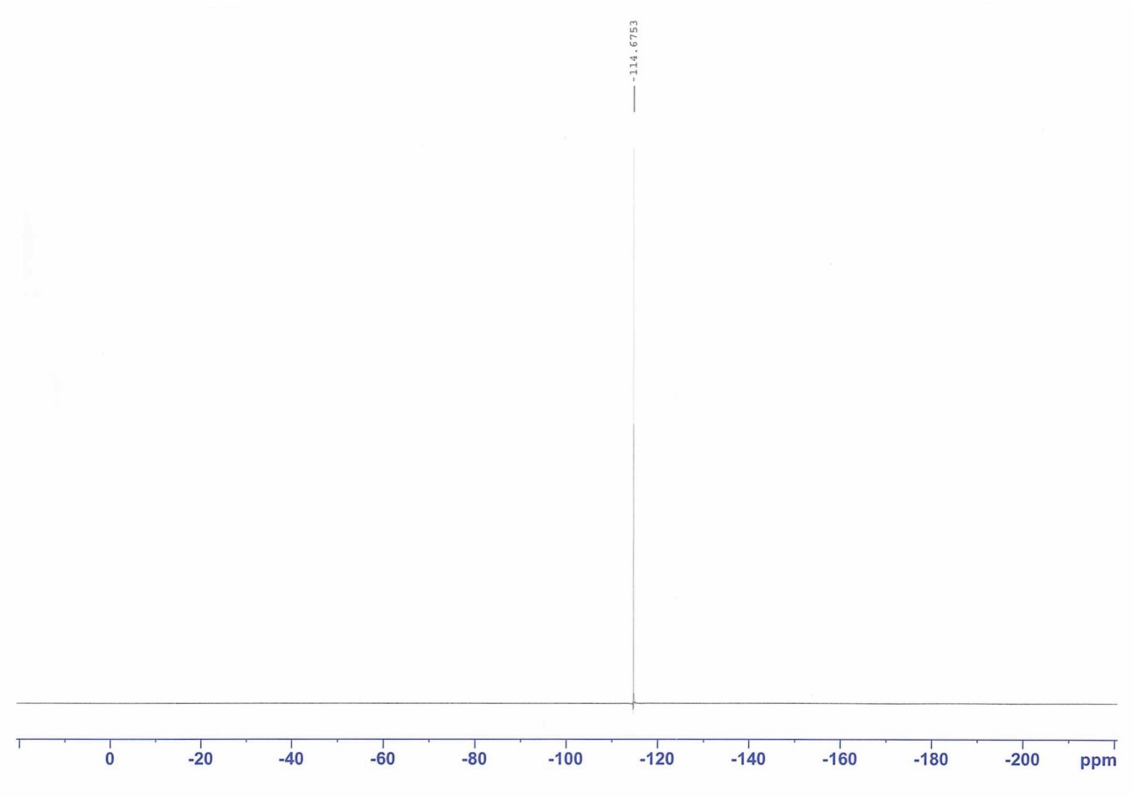

-114.6753
0
-20
-40
-60
-80
-100
-120
-140
-160
-180
-200
ppm

^{1}H NMR (400 MHz) and ^{13}C NMR (101 MHz) spectra of 5-(*tert*-butoxycarbonyl)-3-(ethoxycarbonyl)-6-thiaphenanthridin-6(5*H*)-one (**3p**) ($CDCl_3$)

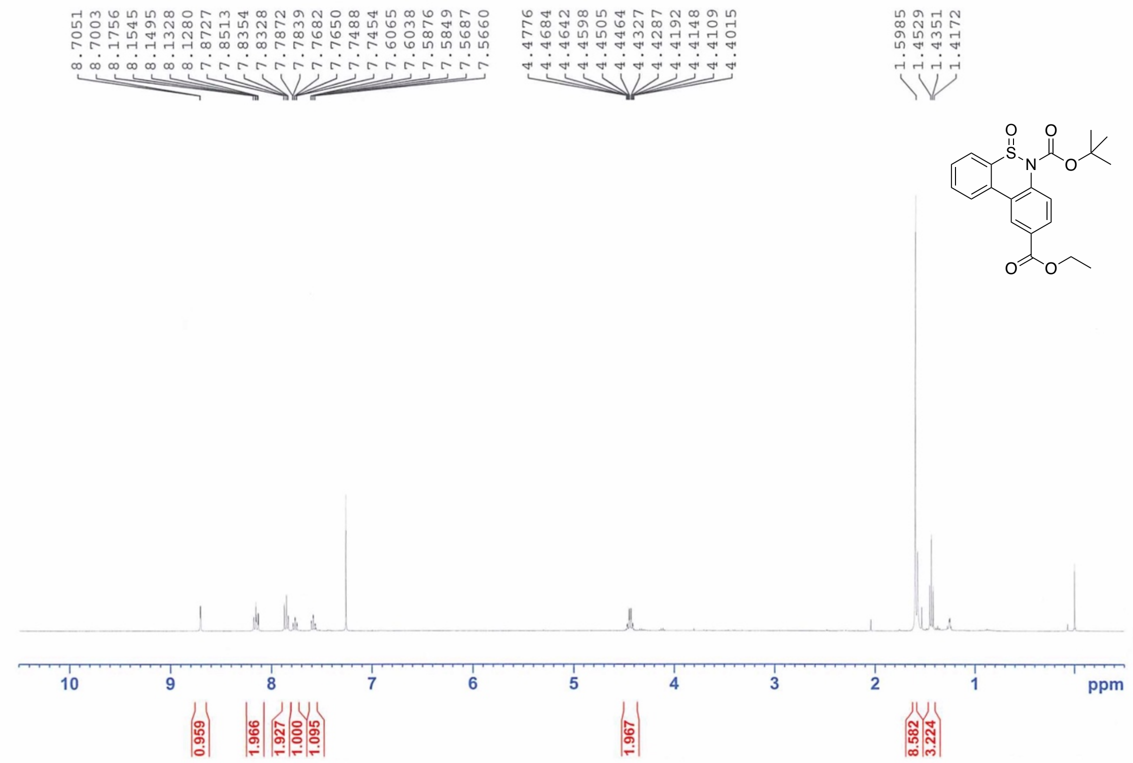

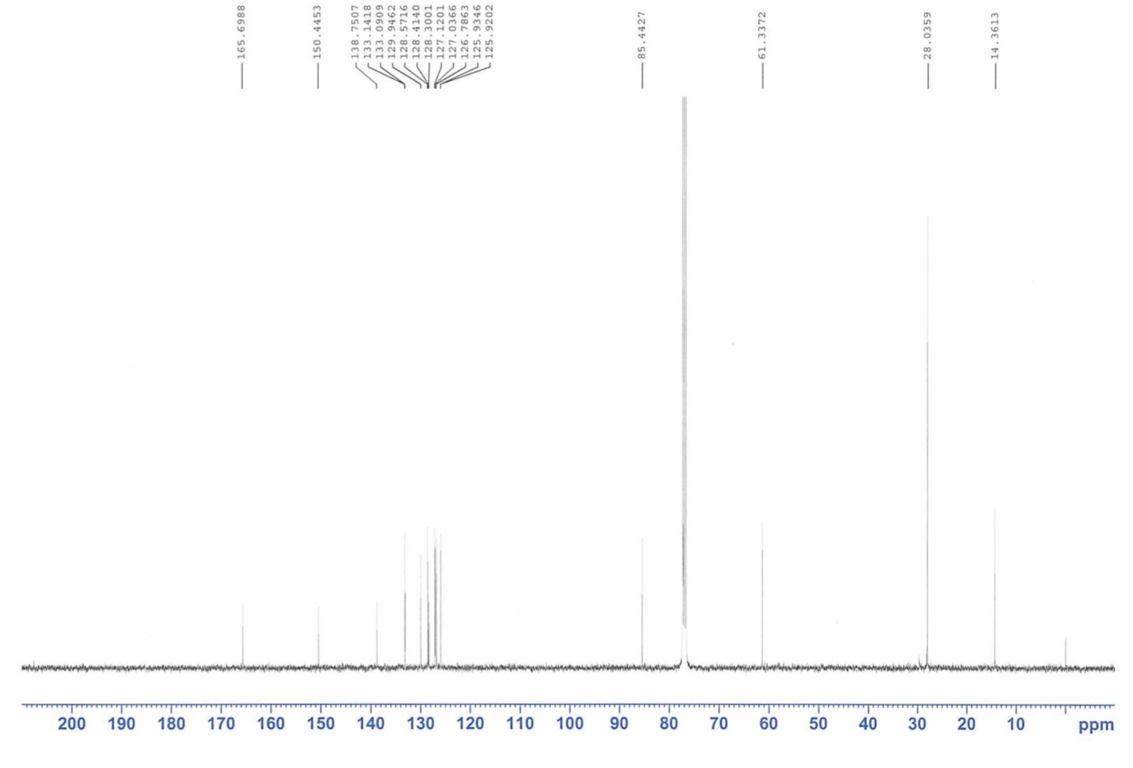

^{1}H NMR (400 MHz), ^{13}C NMR (101 MHz), and ^{19}F NMR (376 MHz) spectra of 5-(*tert*-butoxycarbonyl)-3-(trifluoromethyl)-6-thiaphenanthridin-6(5*H*)-one (**3q**) ($CDCl_3$)

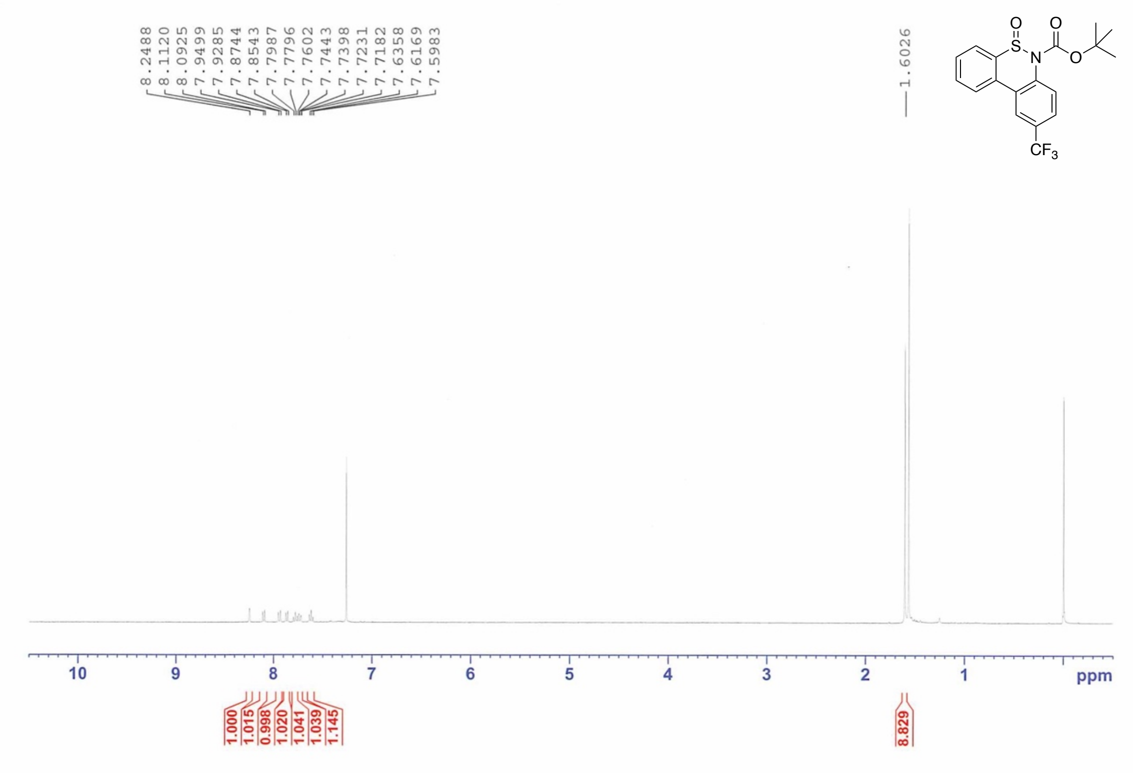

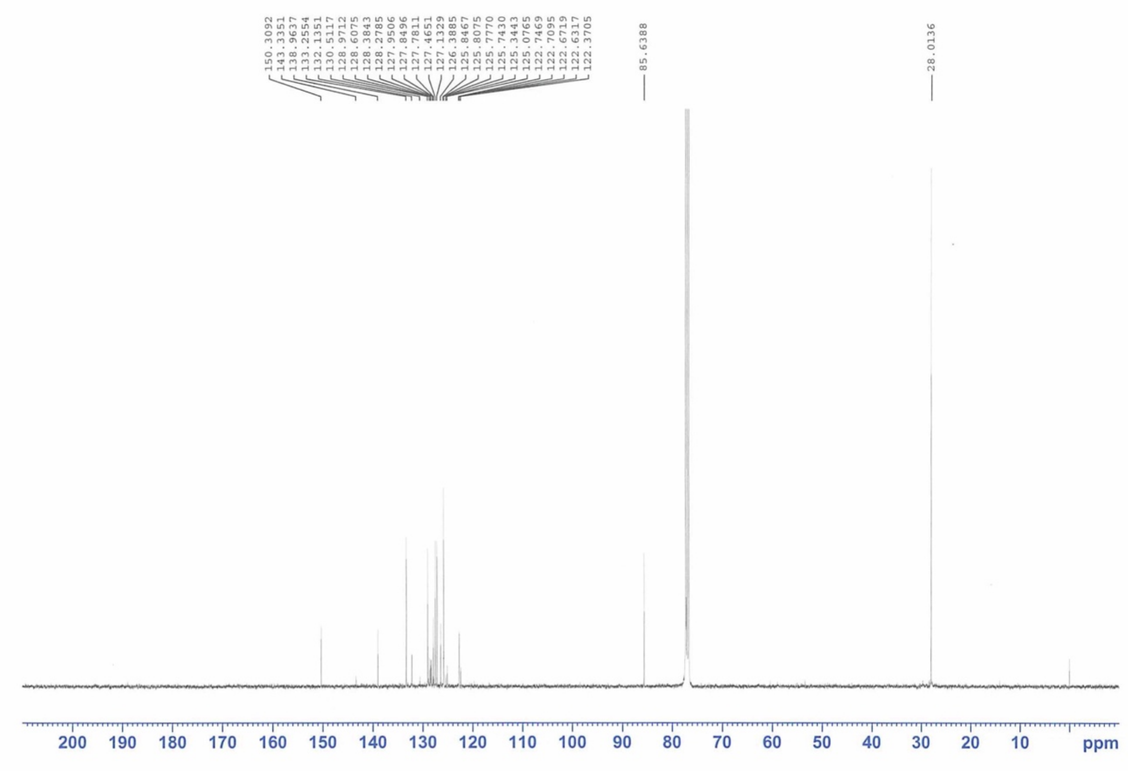

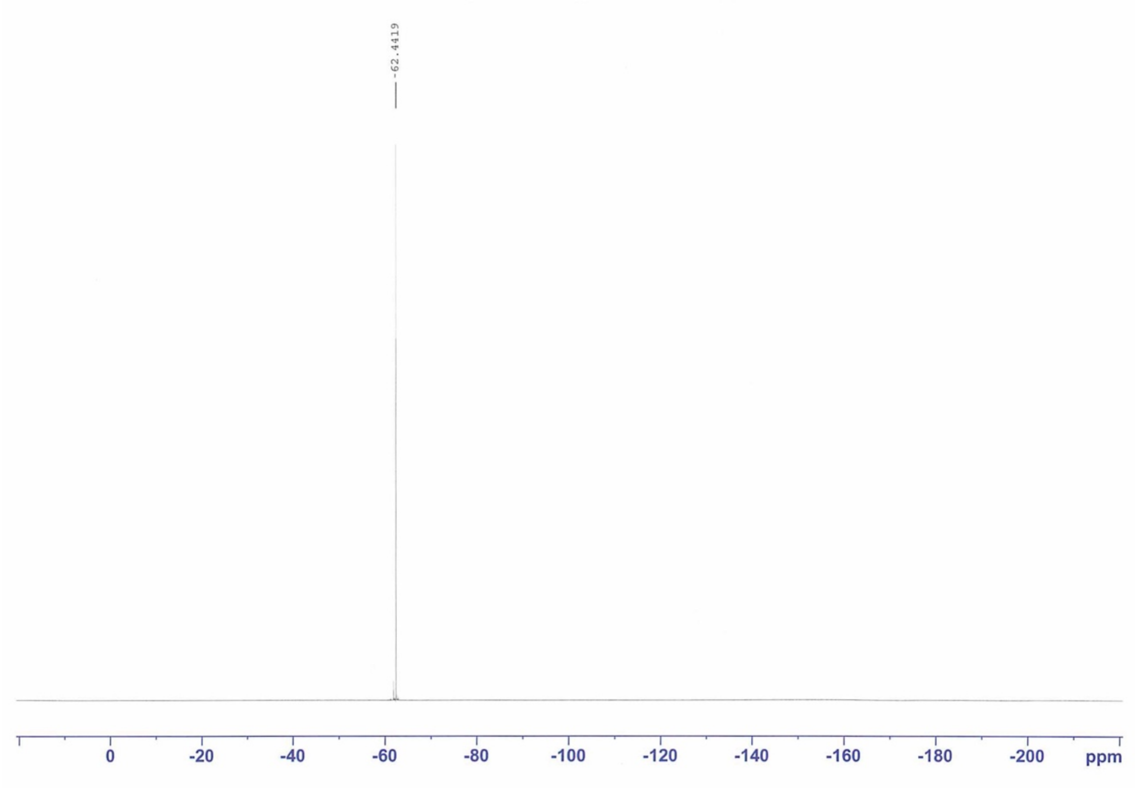

-62.4419
0
-20
-40
-60
-80
-100
-120
-140
-160
-180
-200
ppm

^{1}H NMR (400 MHz) and ^{13}C NMR (101 MHz) spectra of *tert*-butyl 4*H*-benzo[*e*]thieno[2,3-*c*][1,2]thiazine-4-carboxylate 5-oxide (**3r**) ($CDCl_3$)

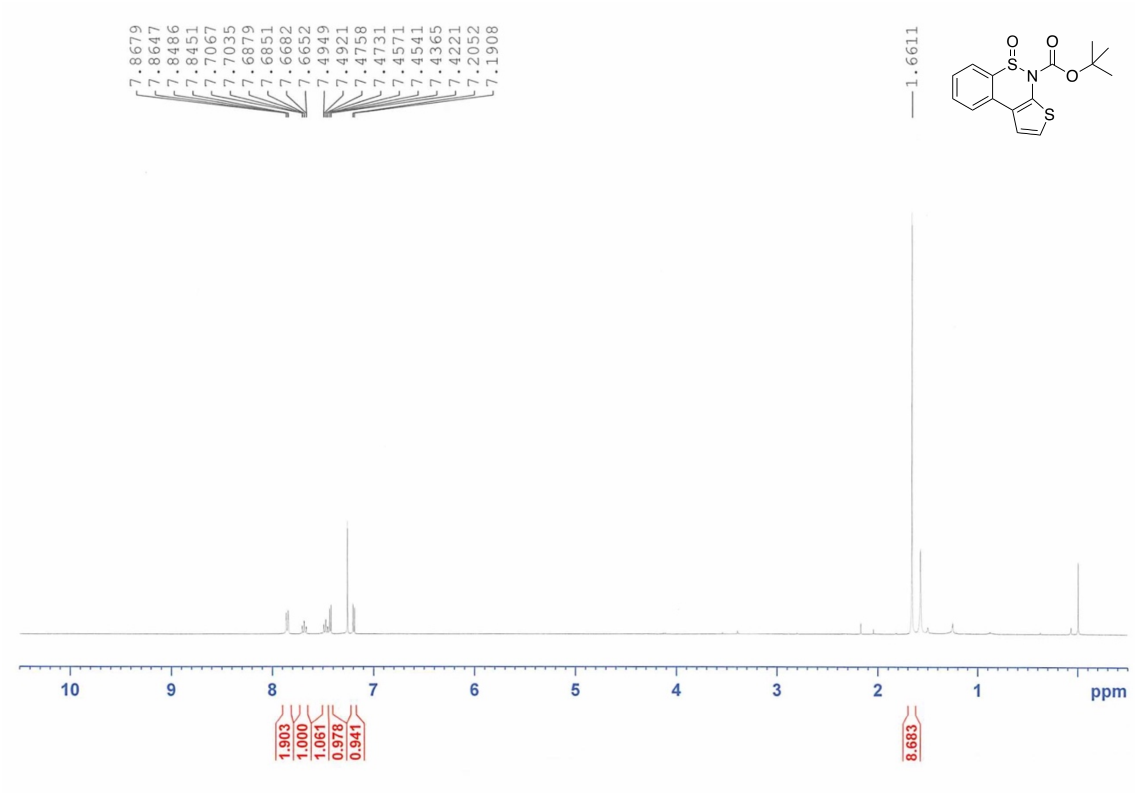

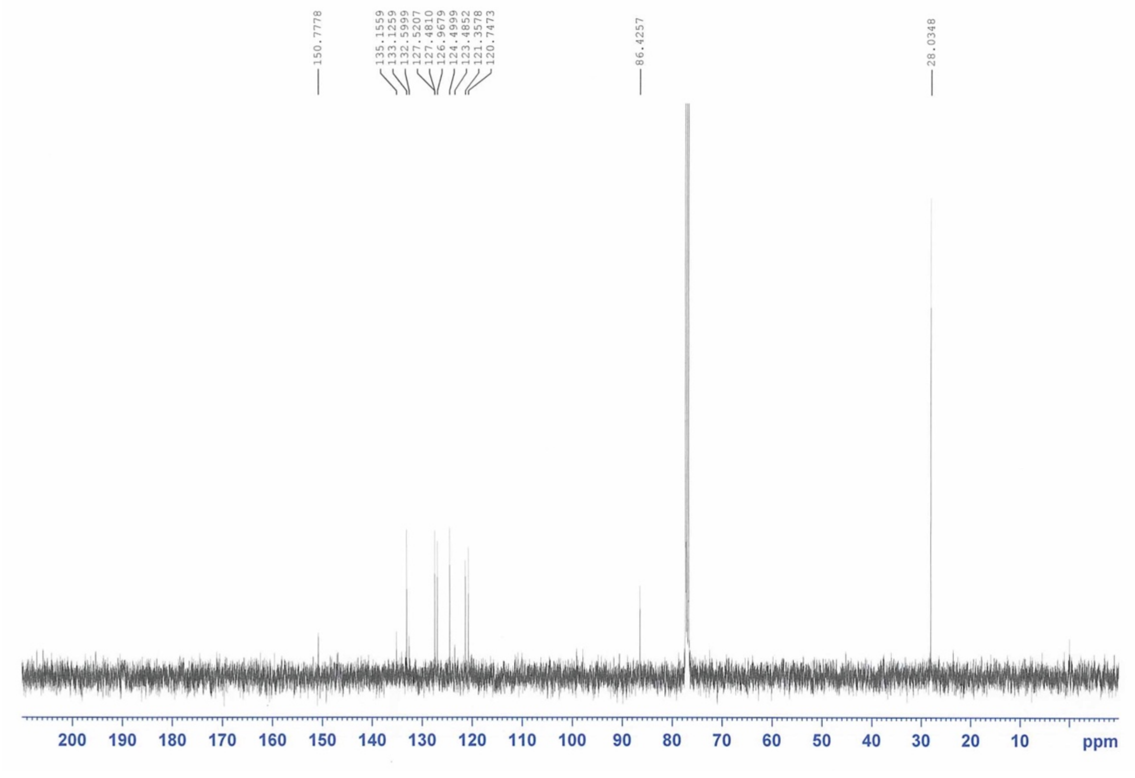

^{1}H NMR (400 MHz) and ^{13}C NMR (101 MHz) spectra of 5-(*tert*-butoxycarbonyl)-[1,3]dioxolo[4,5-*b*]-6-thiaphenanthridin-6(5*H*)-one (**3s**) ($CDCl_3$)

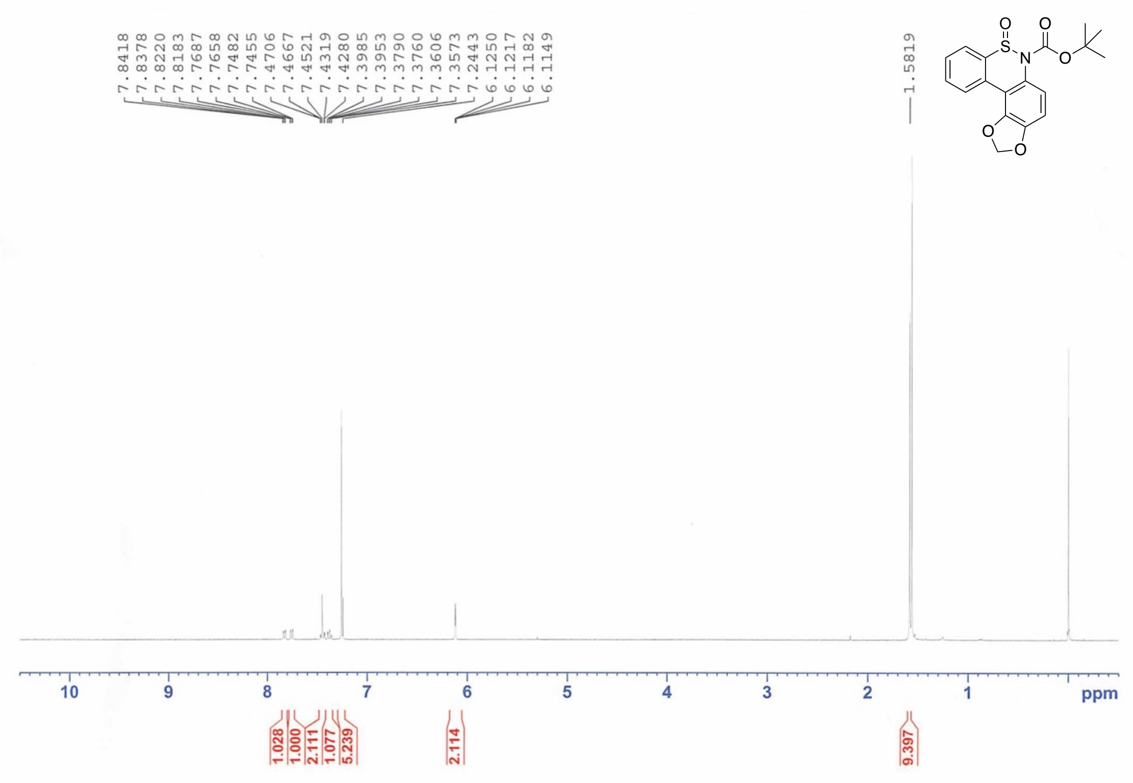

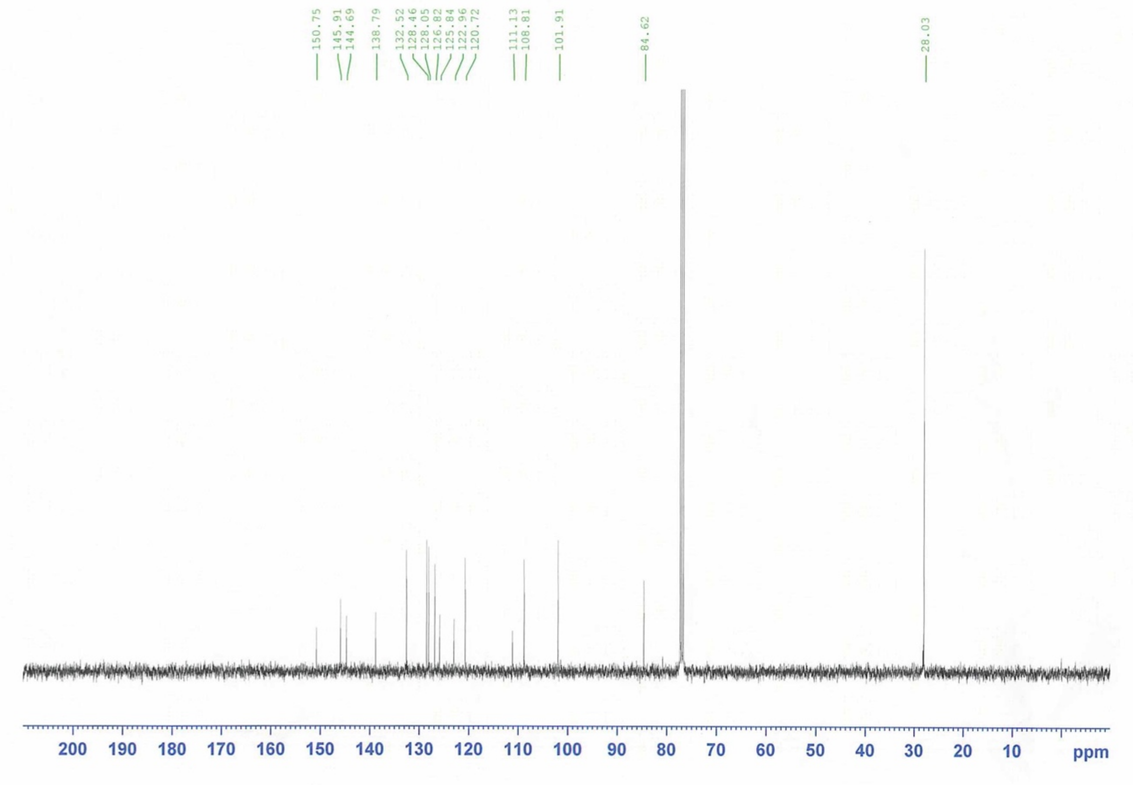

^{1}H NMR (400 MHz) and ^{13}C NMR (101 MHz) spectra of 6-benzyl-6*H*-dibenzo[*c*,*e*][1,2]thiazine 5-oxide (**3t**) ($CDCl_3$)

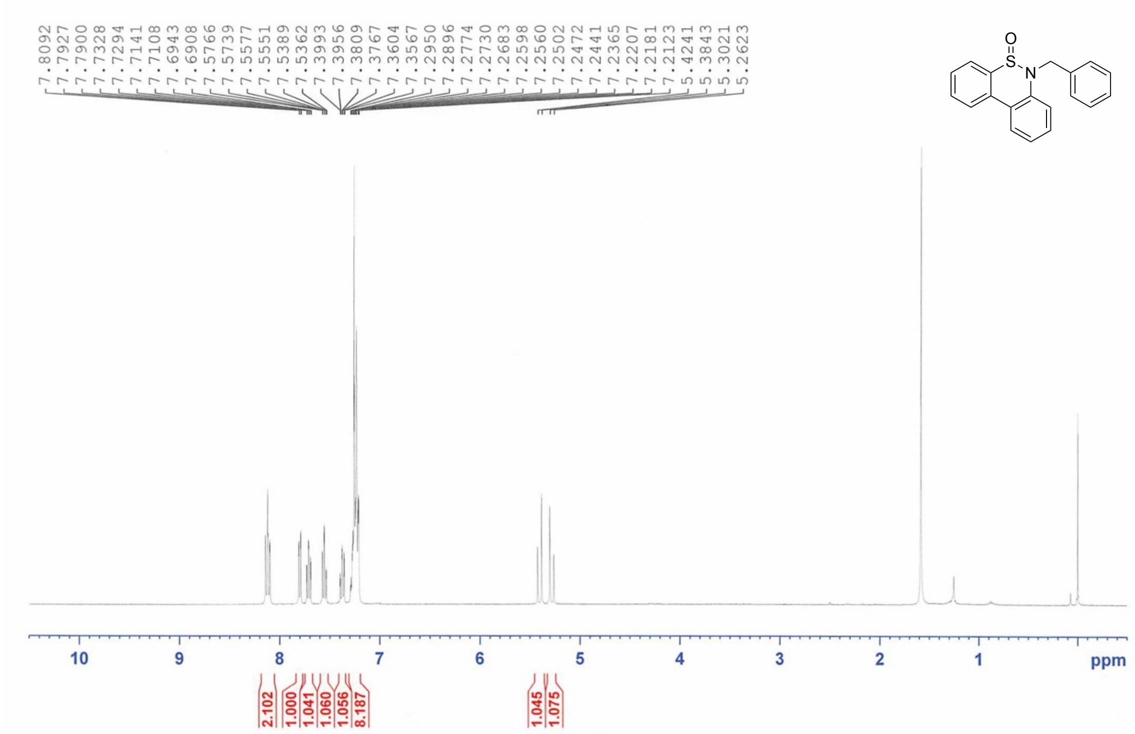

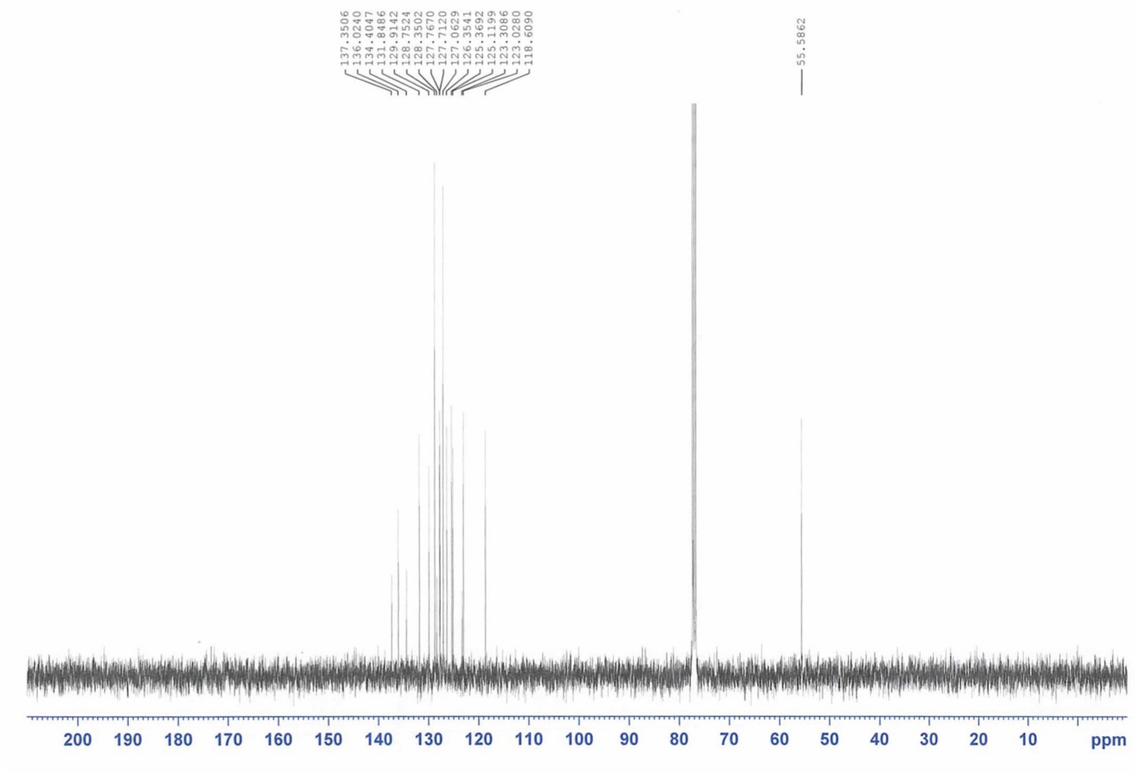

^{1}H NMR (400 MHz) and ^{13}C NMR (101 MHz) spectra of 5-methyl-6-thiaphenanthridin-6(5*H*)-one (**3u**) ($CDCl_3$)

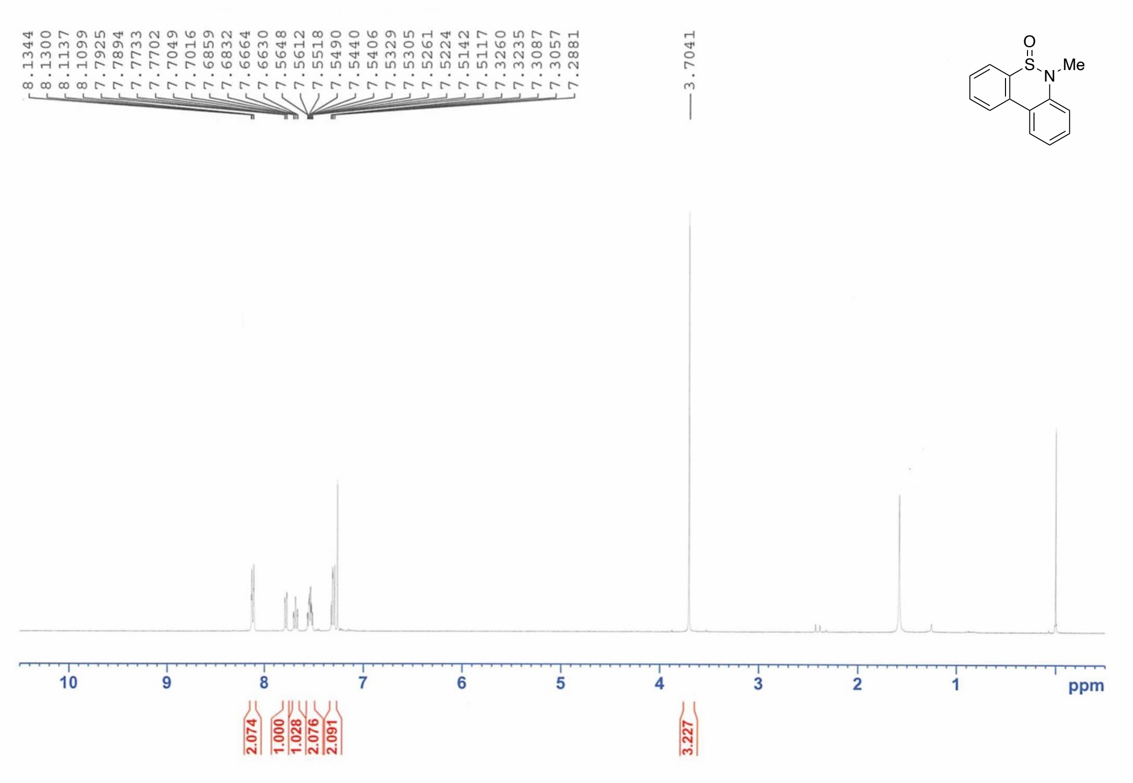

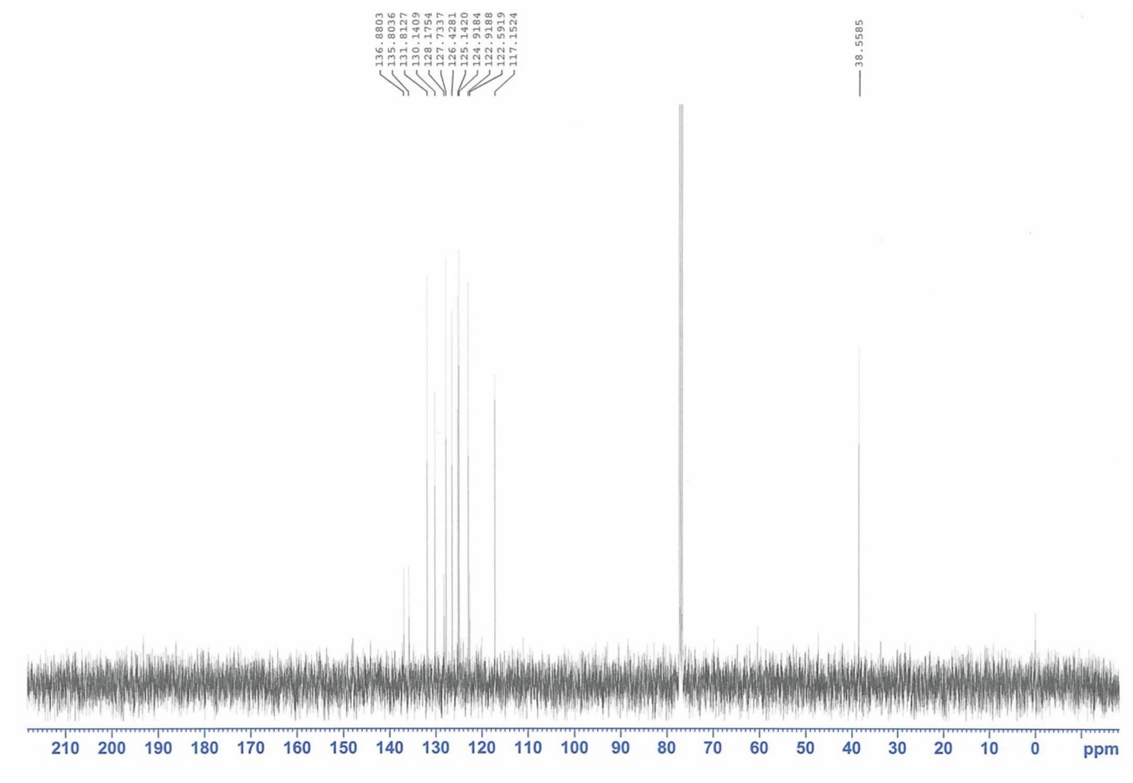

^{1}H NMR (400 MHz) and ^{13}C NMR (101 MHz) spectra of methyl (2-bromophenyl)methanesulfinate (**6**) ($CDCl_3$)

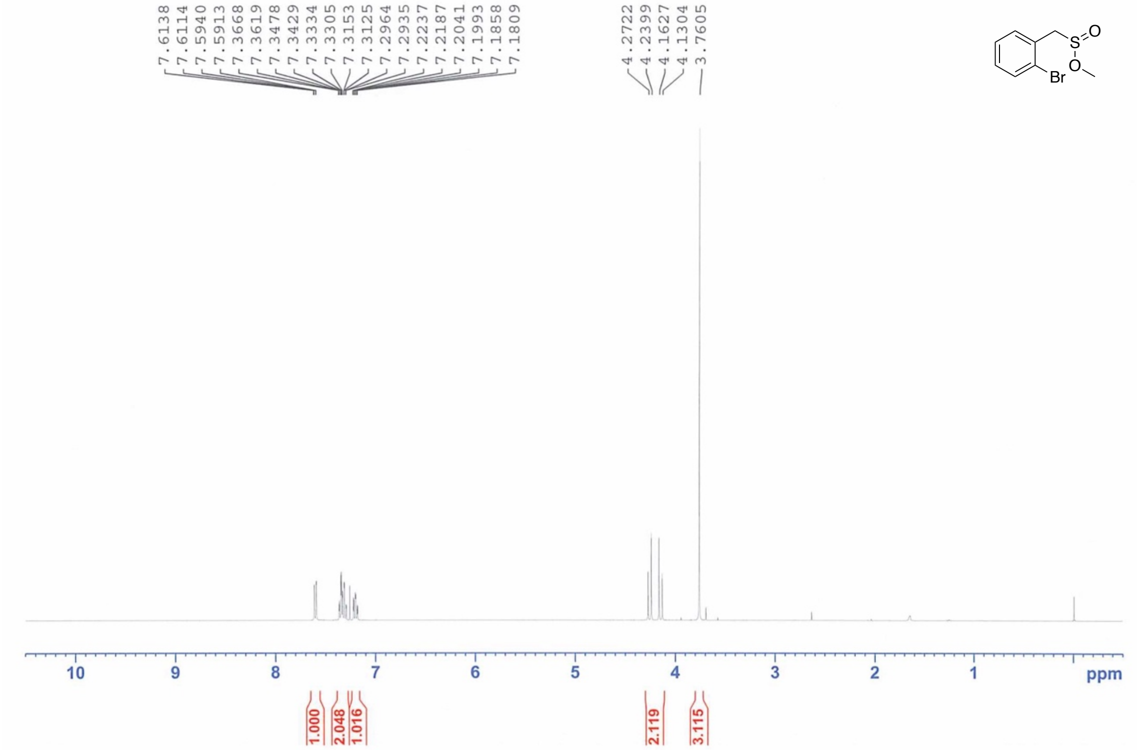

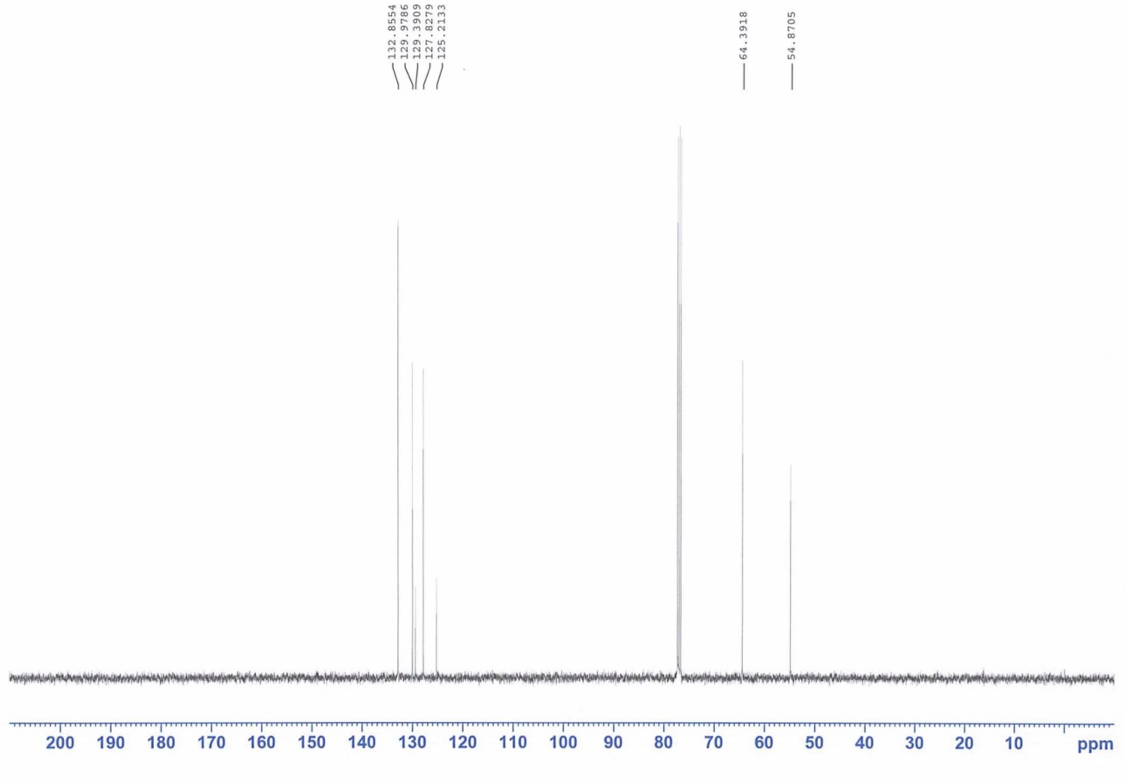

^{1}H NMR (400 MHz) and ^{13}C NMR (101 MHz) spectra of *tert*-butyl dibenzo[*c*,*e*][1,2]thiazepine-5(7*H*)-carboxylate 6-oxide (**7**) ($CDCl_3$)

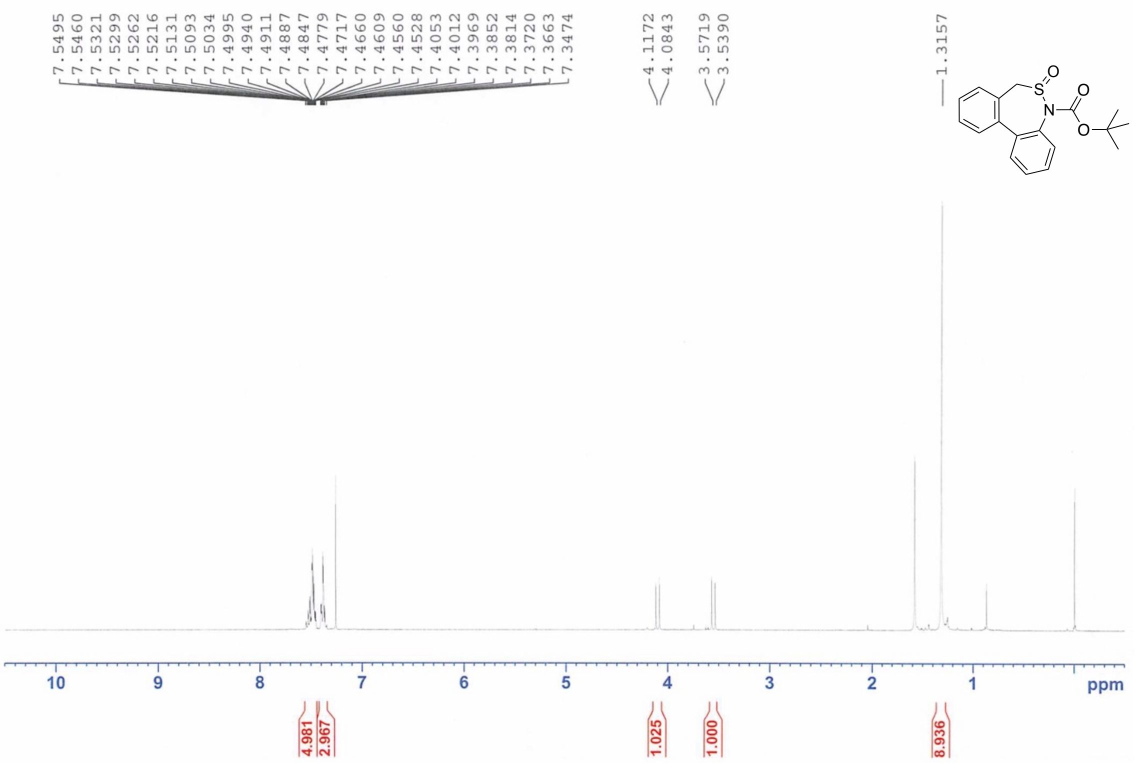

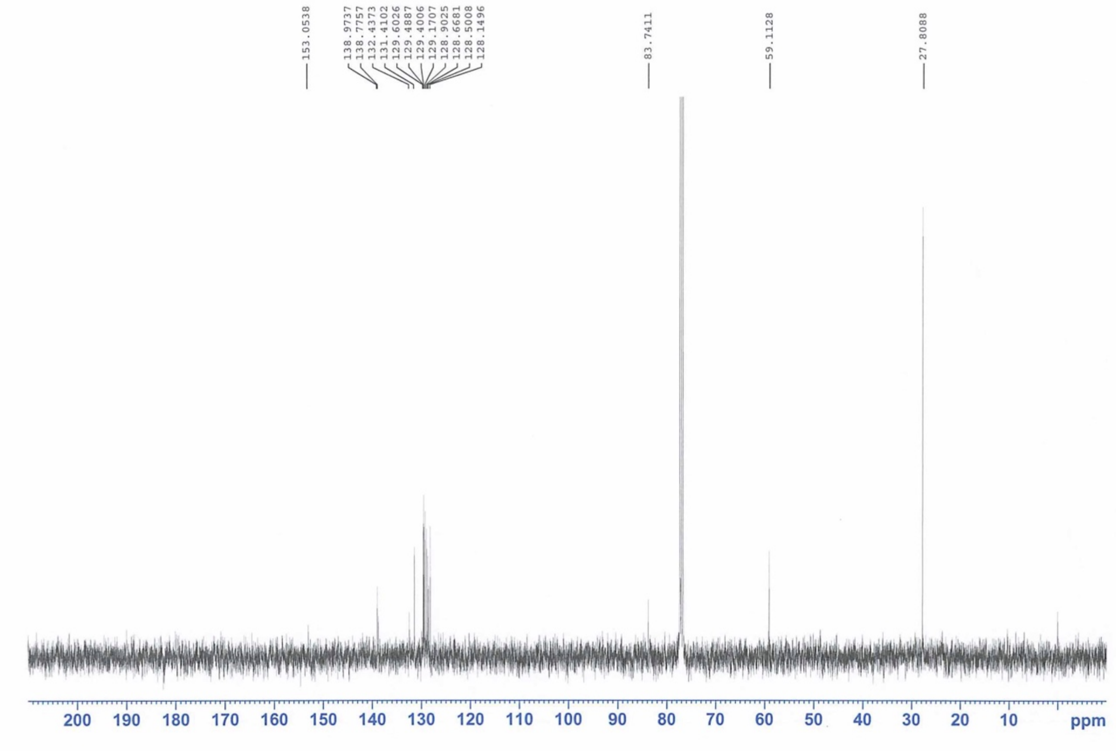

^{1}H NMR (400 MHz) and ^{13}C NMR (101 MHz) spectra of methyl 2′-((*tert*-butoxycarbonyl)amino)-[1,1′-biphenyl]-2-sulfinate (**4**) ($CDCl_3$)

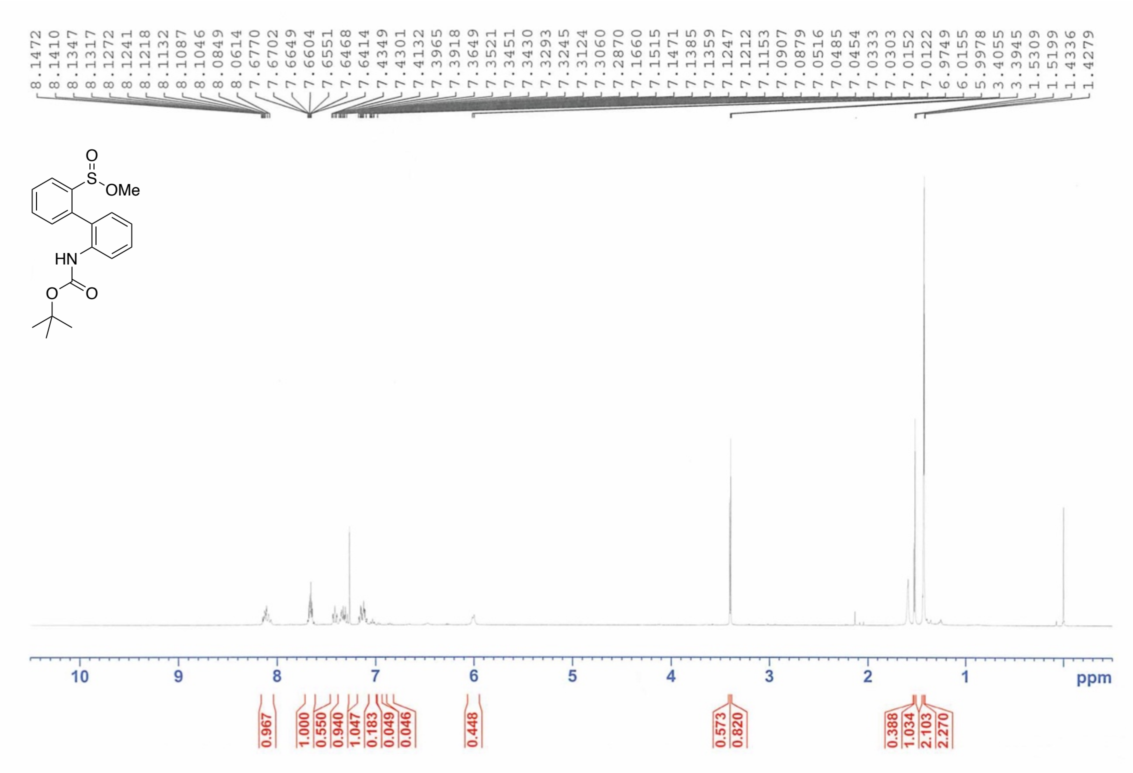

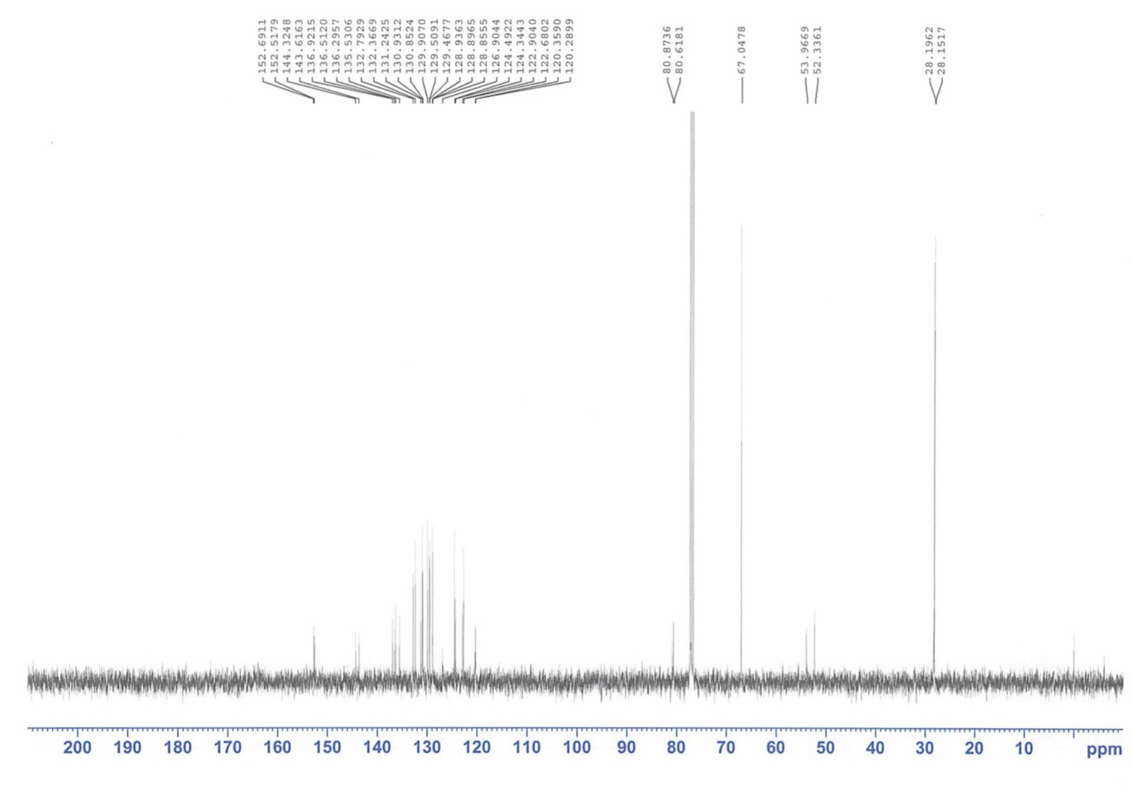

^{1}H NMR (400 MHz, $CDCl_3$) and ^{13}C NMR (101 MHz, CD_3OD) spectra of 6-thiaphenanthridin-6(5*H*)-one (**5a**)

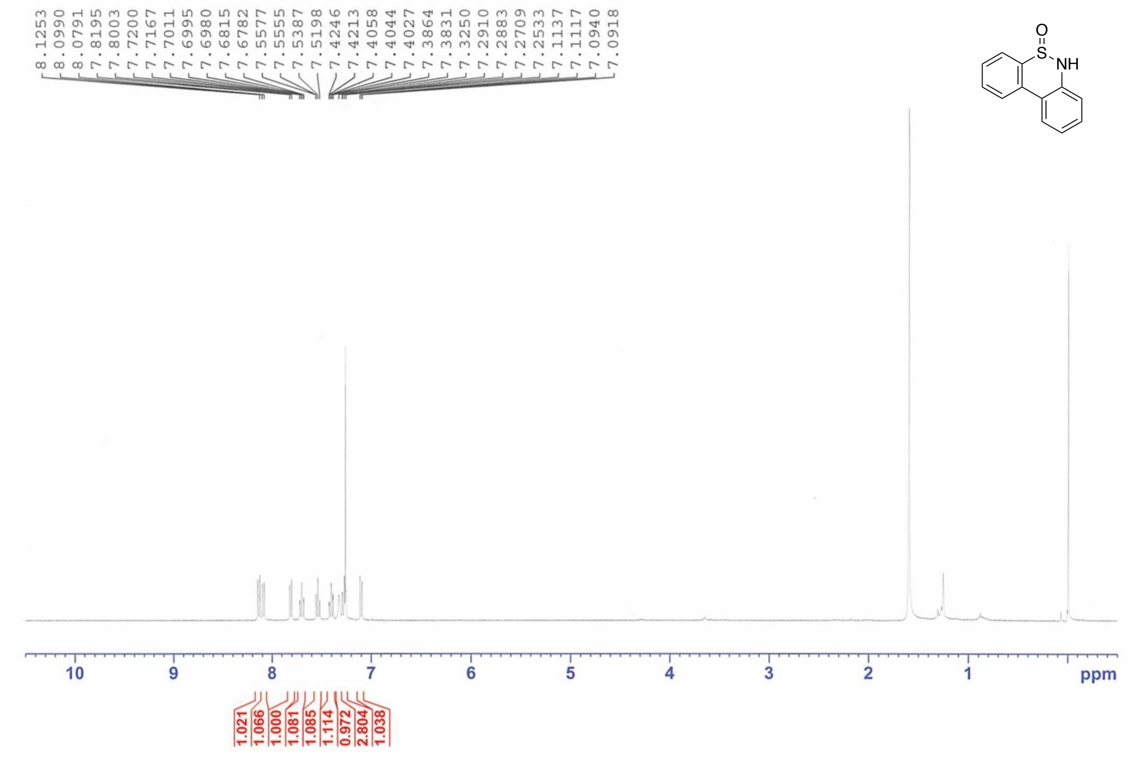

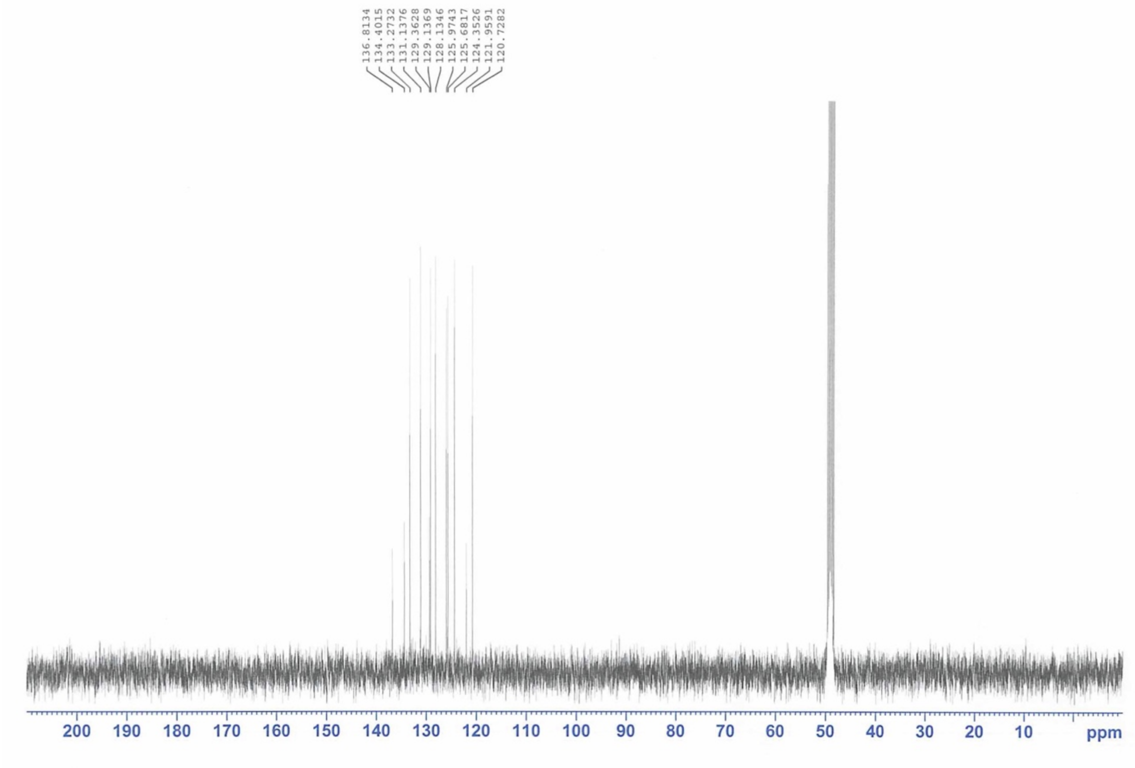

^{1}H NMR (400 MHz) and ^{13}C NMR (101 MHz) spectra of ethyl 6*H*-dibenzo[*c*,*e*][1,2]thiazine-2-carboxylate 5-oxide (**5b**) ($CDCl_3$)

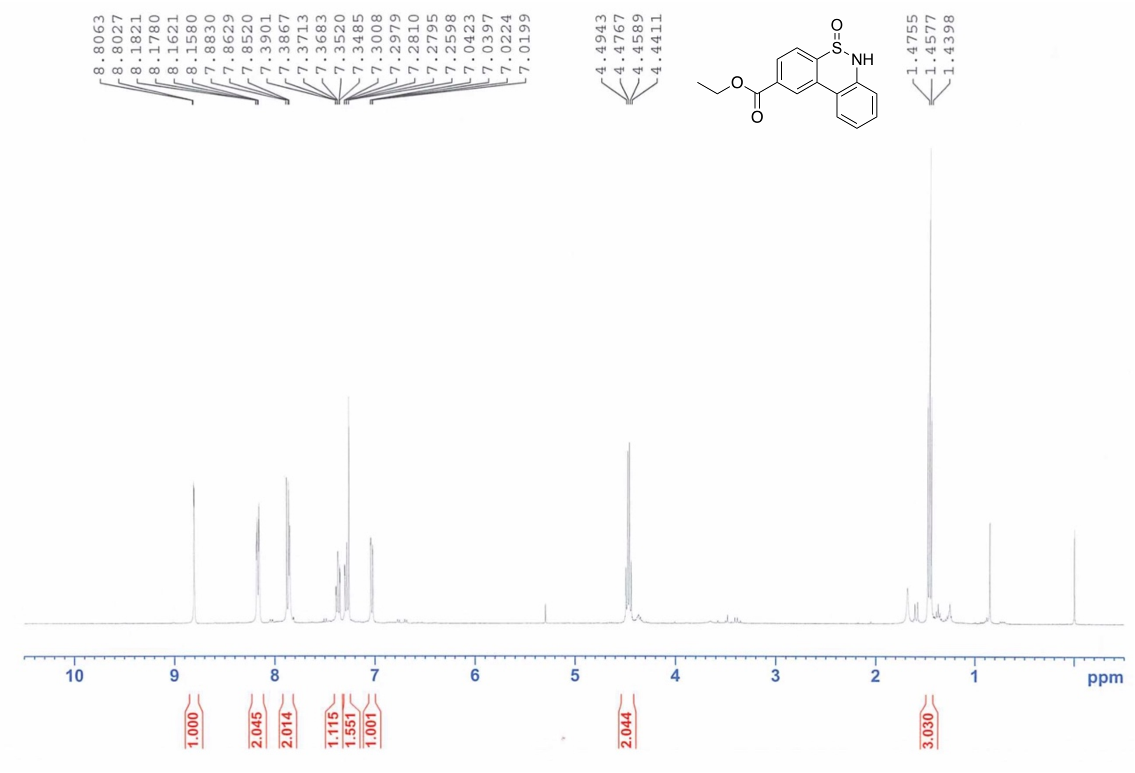

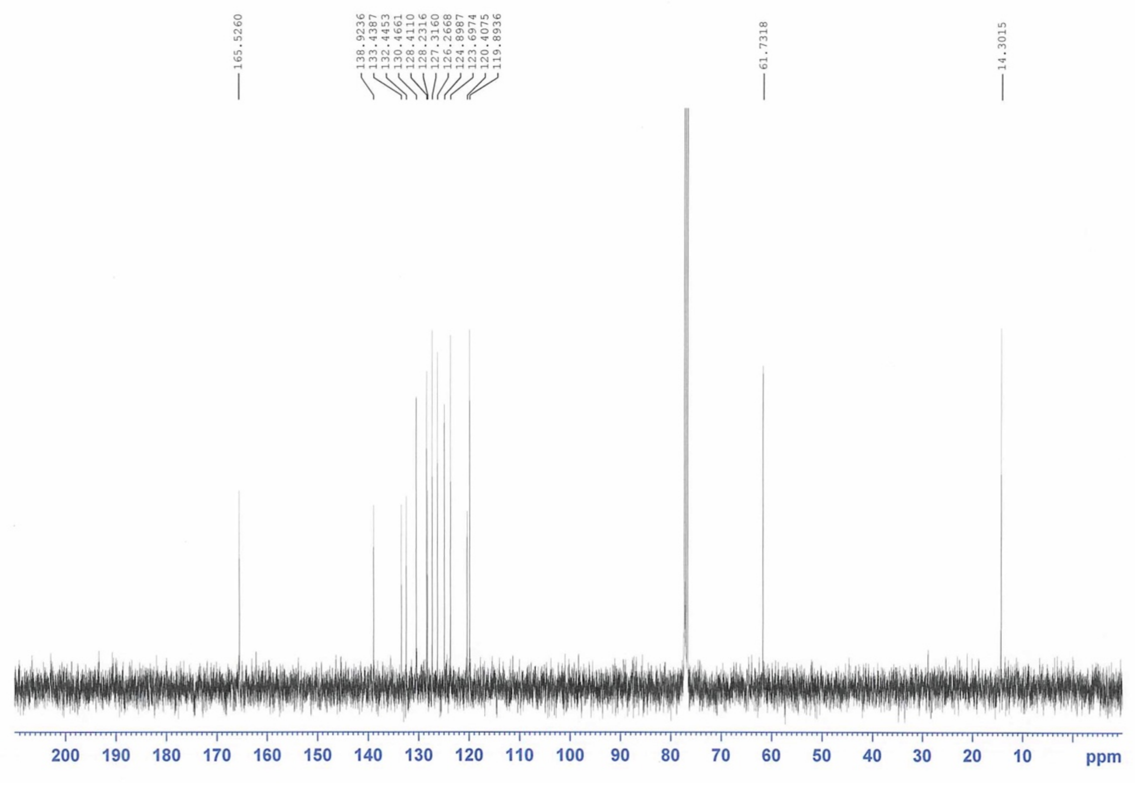

^{1}H NMR (400 MHz, $CDCl_3$) and ^{13}C NMR (101 MHz, CD_3OD) spectra of 9-chloro-6*H*-dibenzo[*c*,*e*][1,2]thiazine 5-oxide (**5c**)

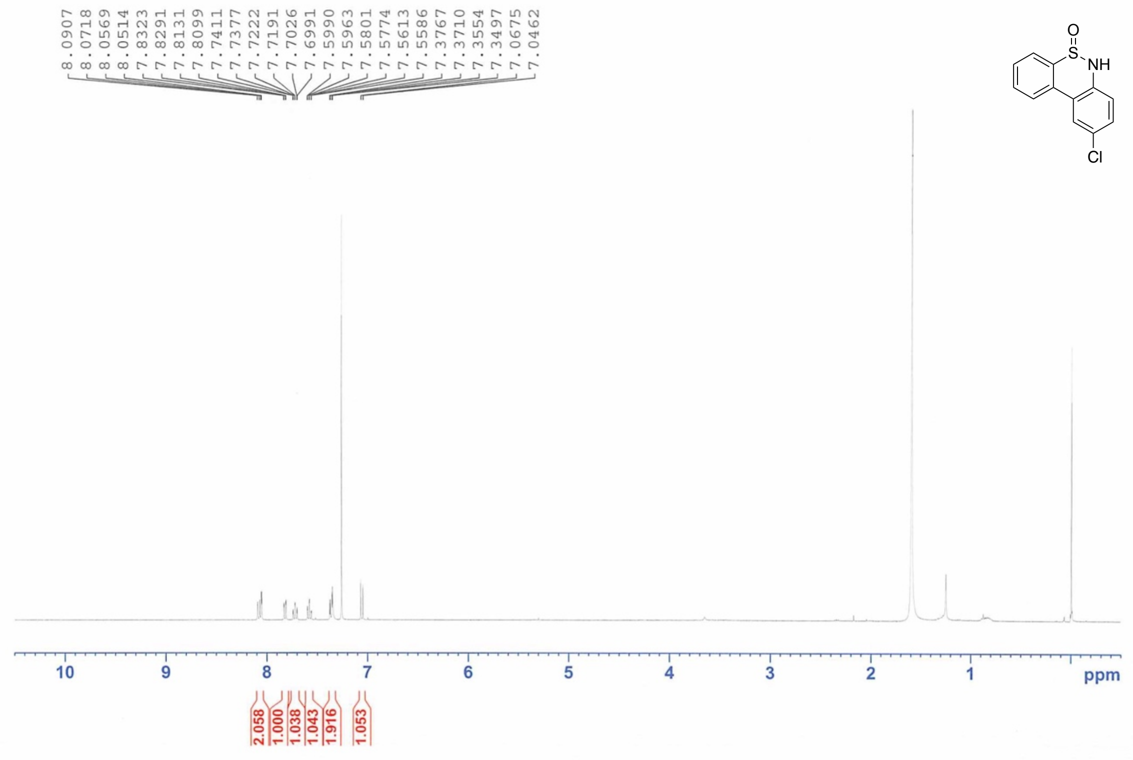

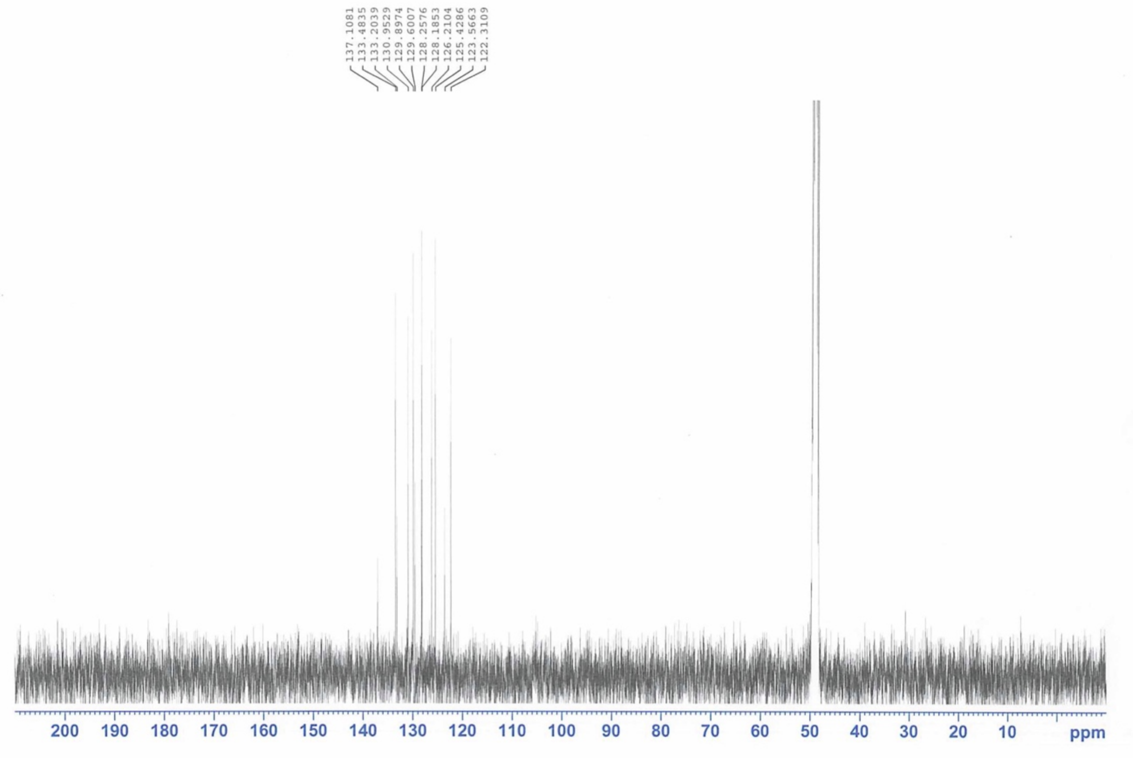

^{1}H NMR (400 MHz) and ^{13}C NMR (101 MHz) spectra of *tert*-butyl 6*H*-dibenzo[*c,e*][1,2]thiazine-6-carboxylate 5,5-dioxide (**8**) ($CDCl_3$)

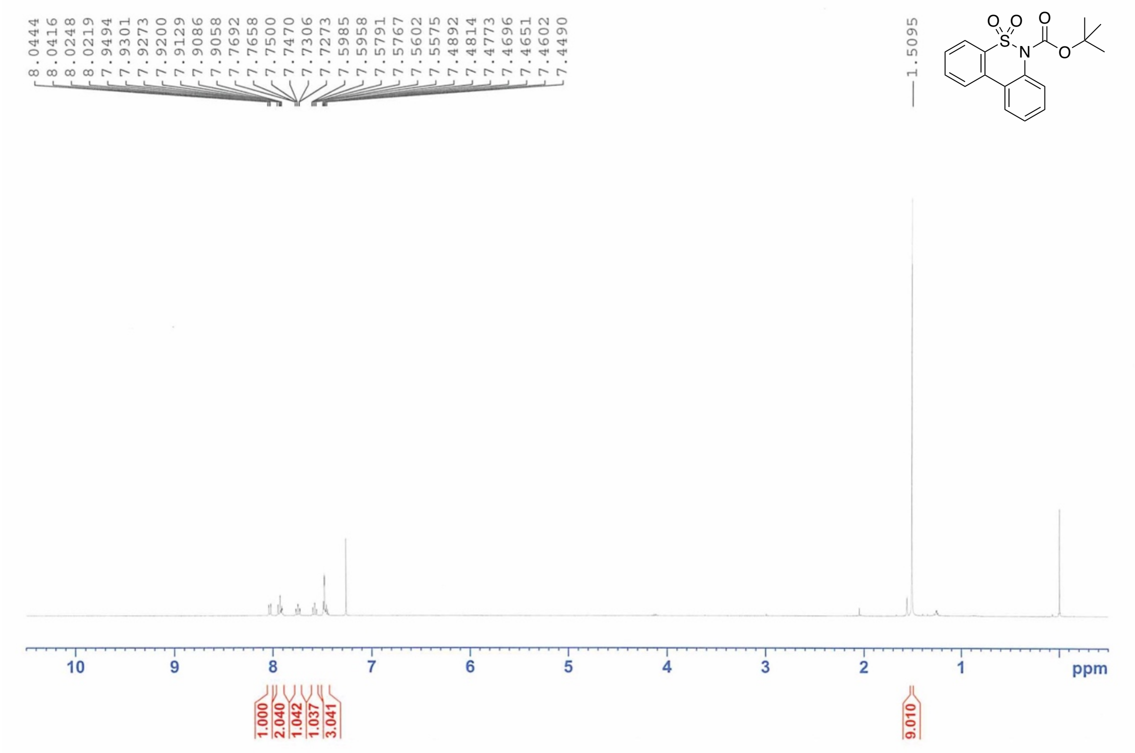

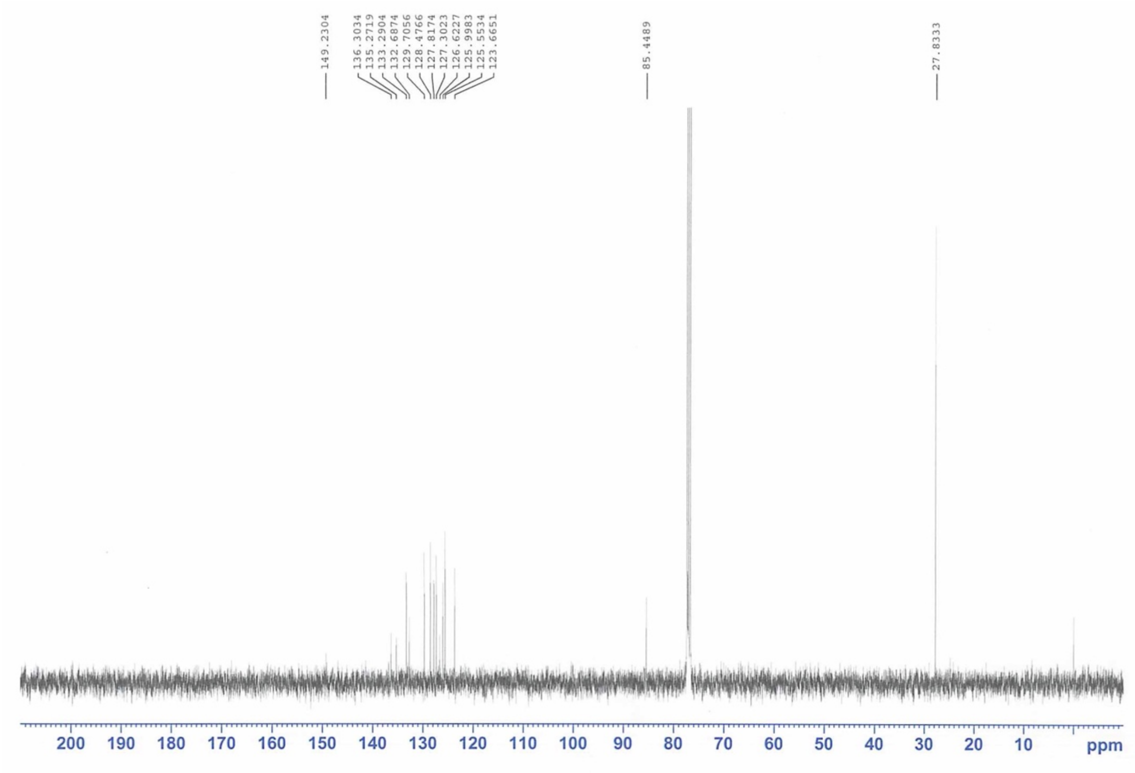

^{1}H NMR (400 MHz) and ^{13}C NMR (101 MHz) spectra of *tert*-butyl 6*H*-dibenzo[*c*,*e*][1,2]thiazine-6-carboxylate (**9**) ($CDCl_3$)

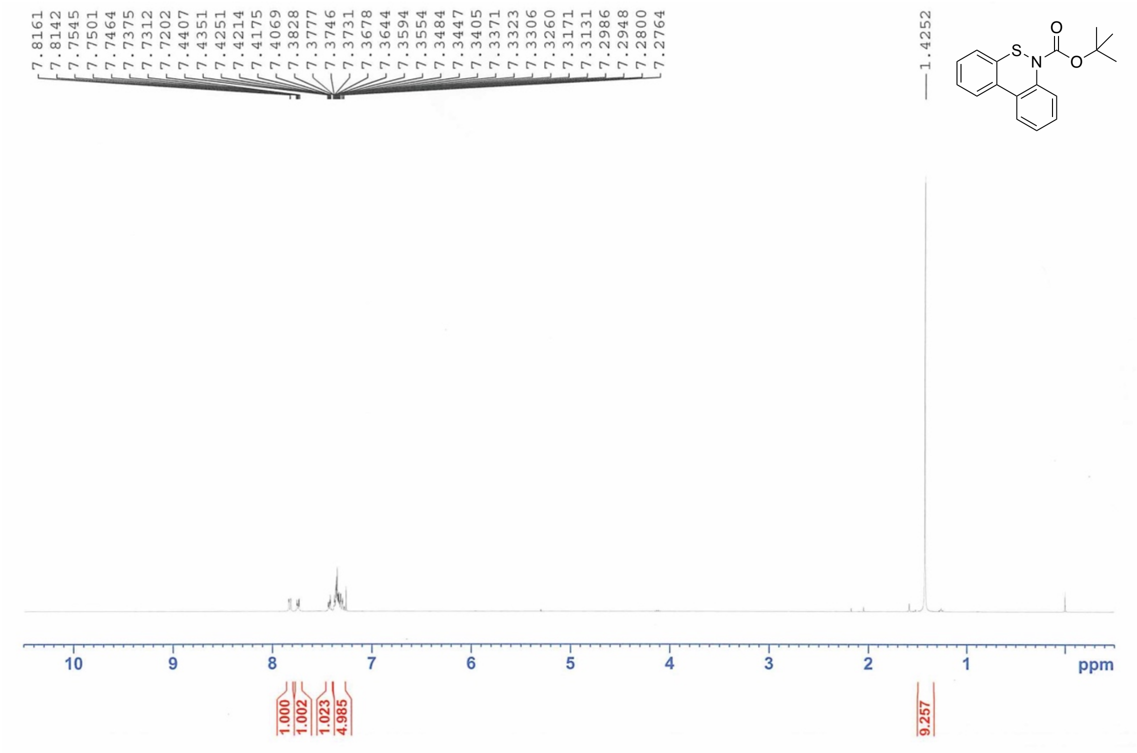

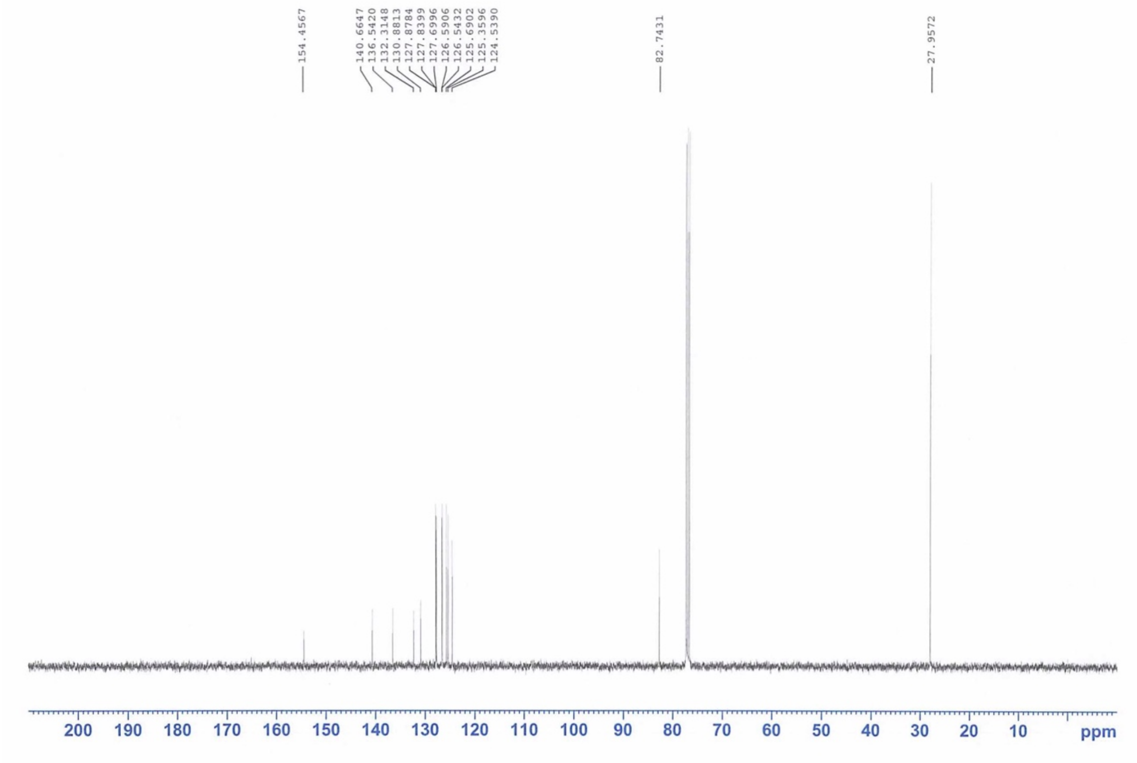

^{1}H NMR (400 MHz), ^{13}C NMR (101 MHz), and ^{19}F NMR (376 MHz) spectra of 5-fluoro-5λ[4]-dibenzo[*c*,*e*][1,2]thiazine 5-oxide (**11**) ($CDCl_3$)

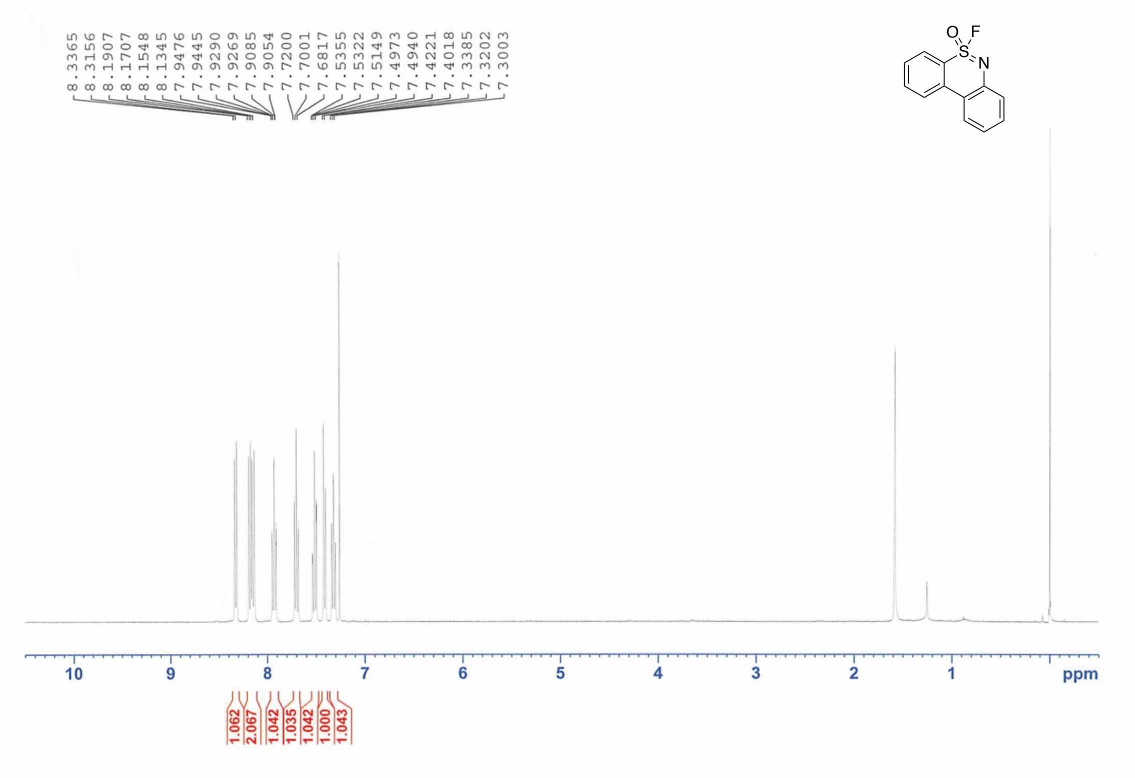

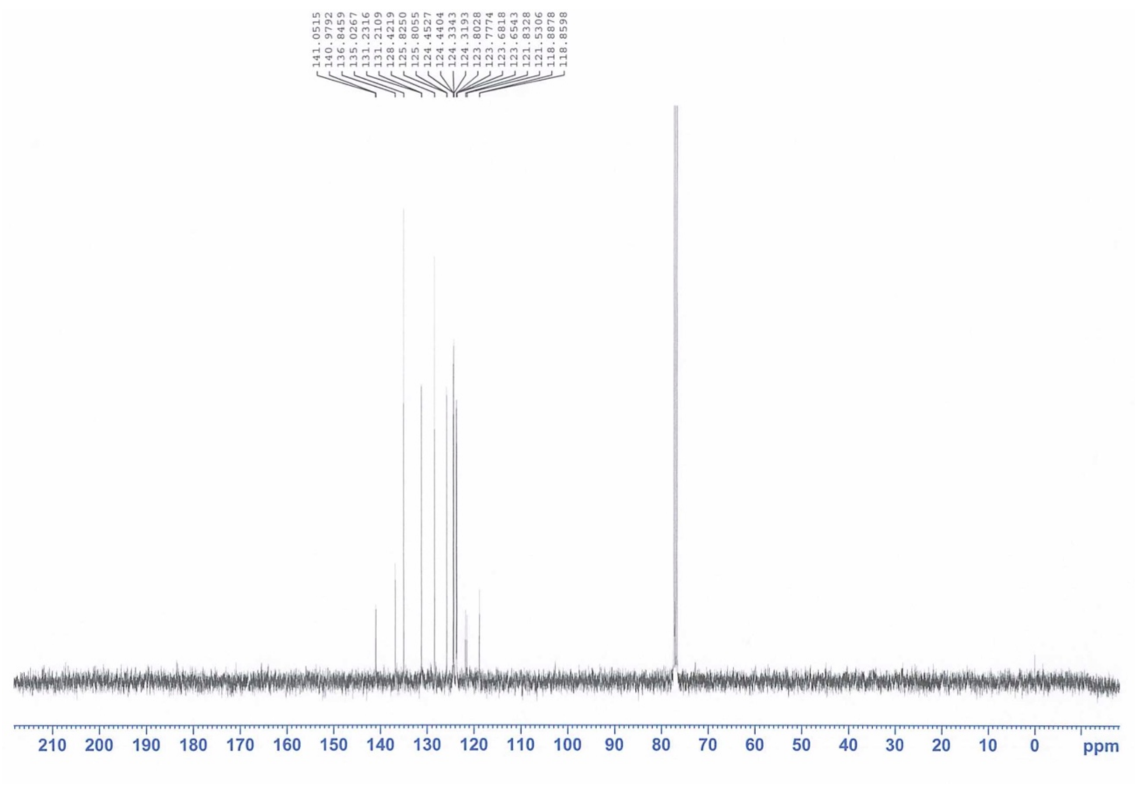

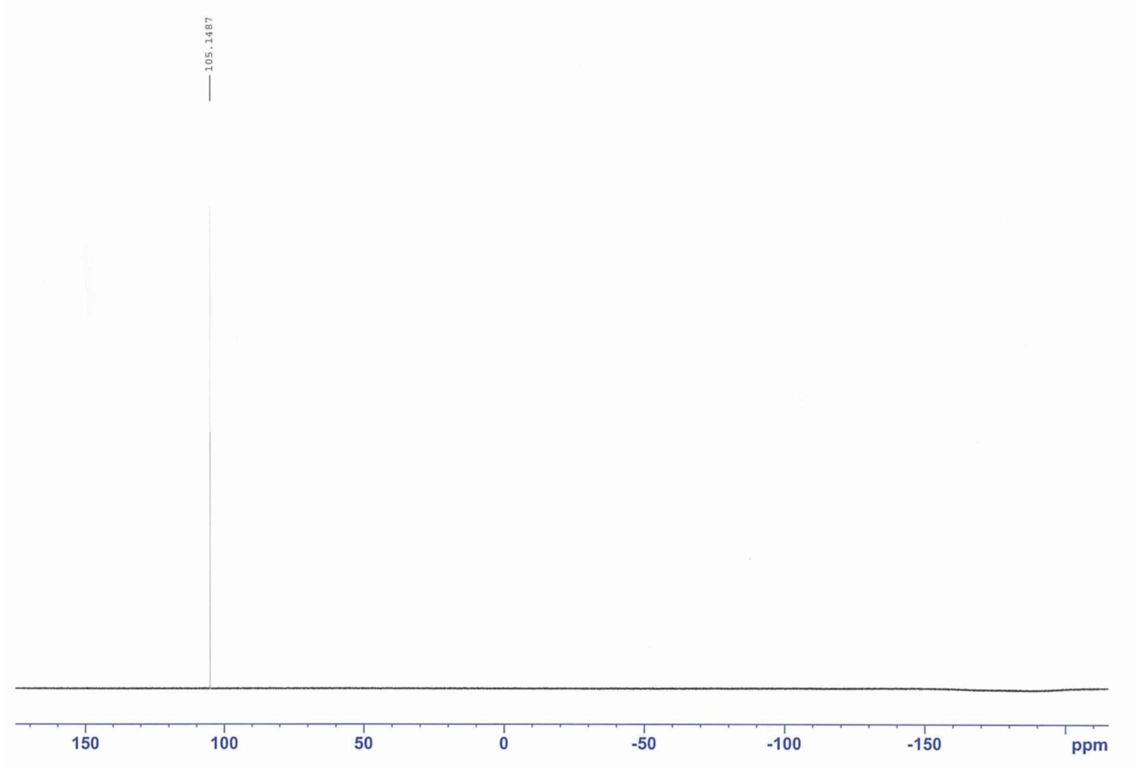
105.1487
150
100
50
0
-50
-100
-150
ppm

^{1}H NMR (400 MHz) and ^{13}C NMR (101 MHz) spectra of 5-butyl-5λ^4-dibenzo[*c*,*e*][1,2]thiazine 5-oxide (**12**) ($CDCl_3$)

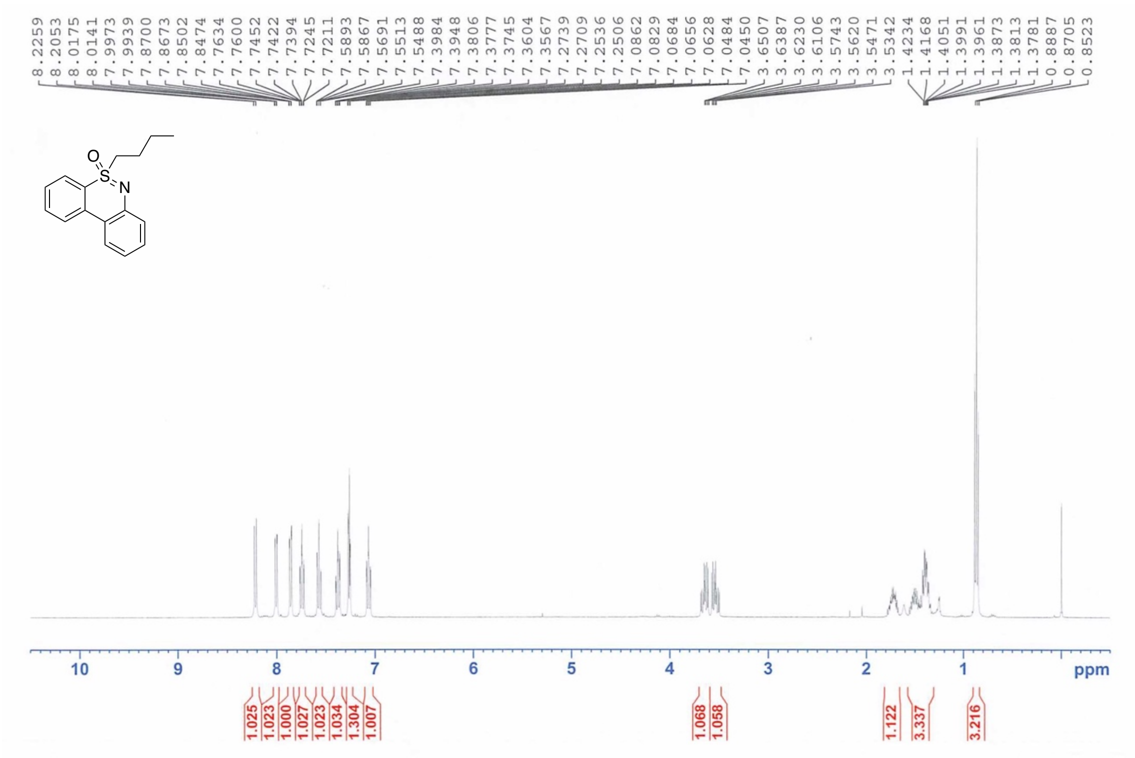

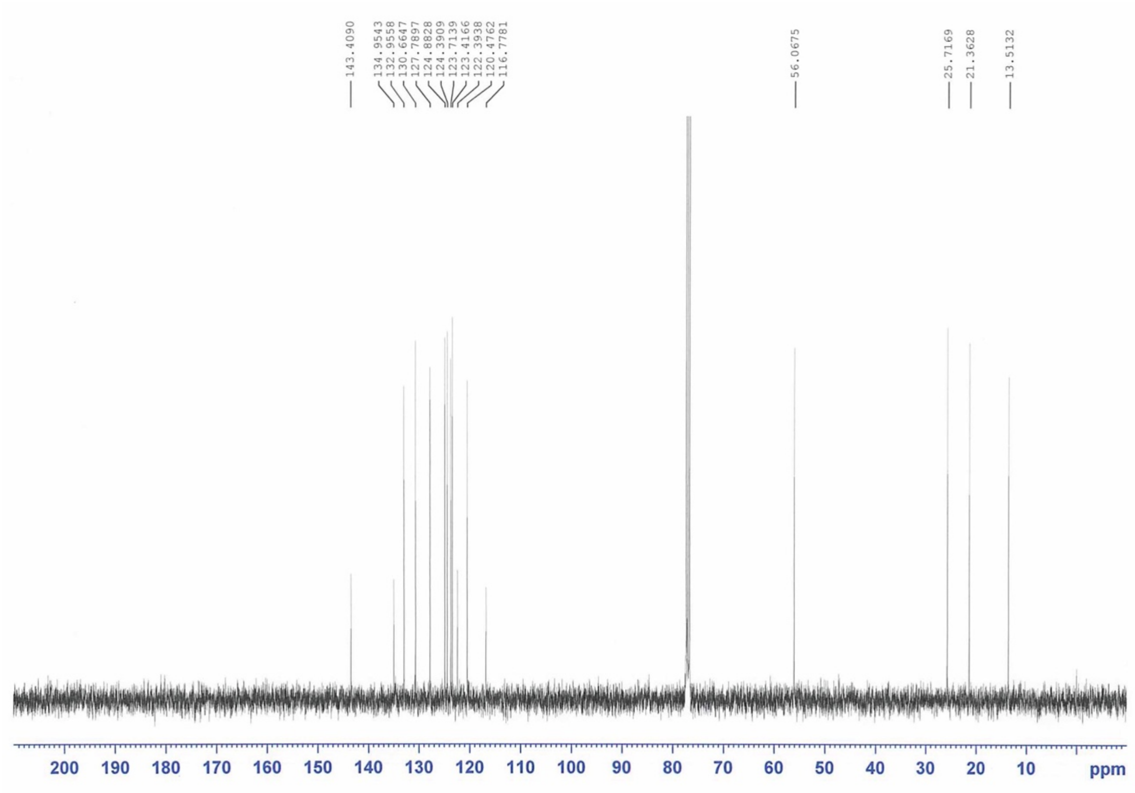

^{1}H NMR (400 MHz) and ^{13}C NMR (101 MHz) spectra of 5-(4-methoxyphenoxy)-5λ^{4}-dibenzo[*c*,*e*][1,2]thiazine 5-oxide (**13**) ($CDCl_3$)

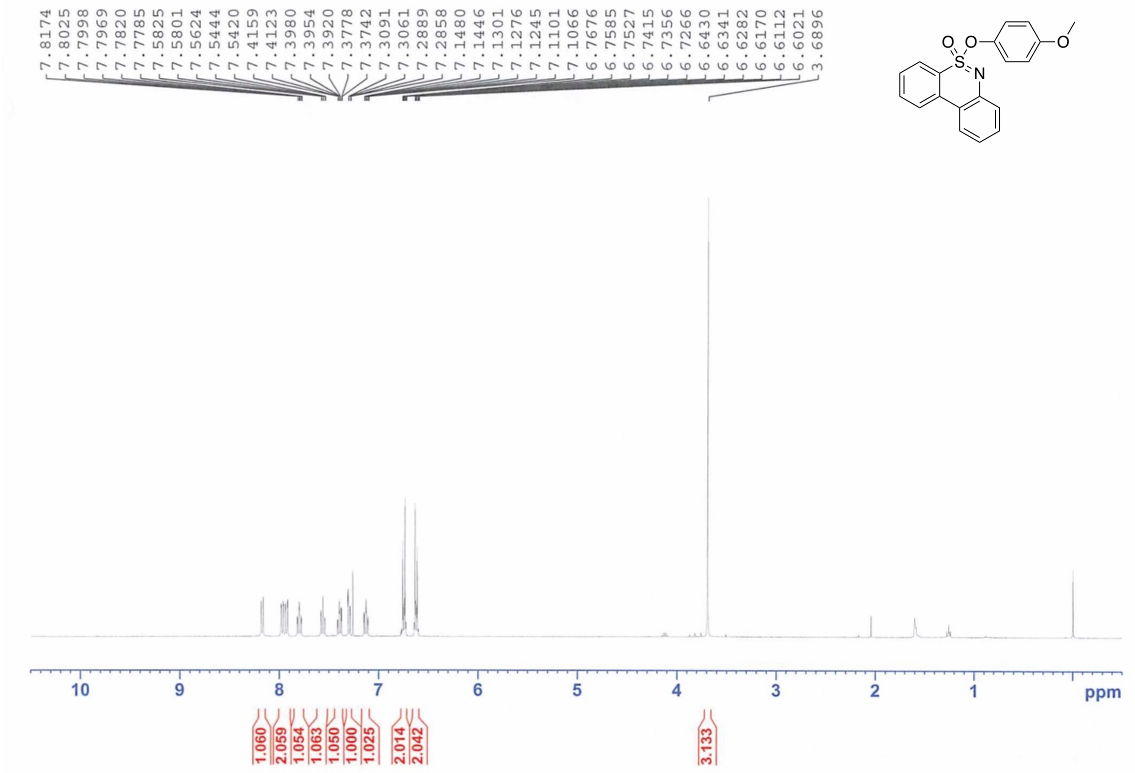

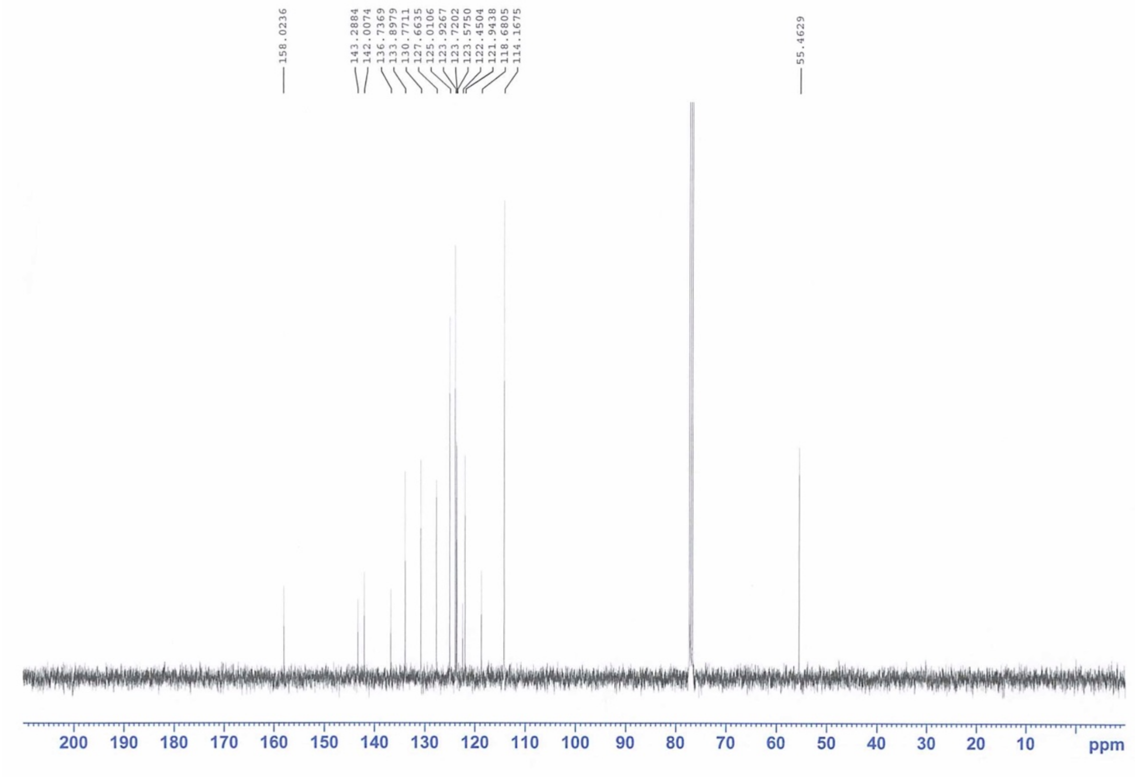

^{1}H NMR (400 MHz) and ^{13}C NMR (101 MHz) spectra of 5-morpholinodibenzo[*c*,*e*][1,2]thiazine 5-oxide (**14**) ($CDCl_3$)

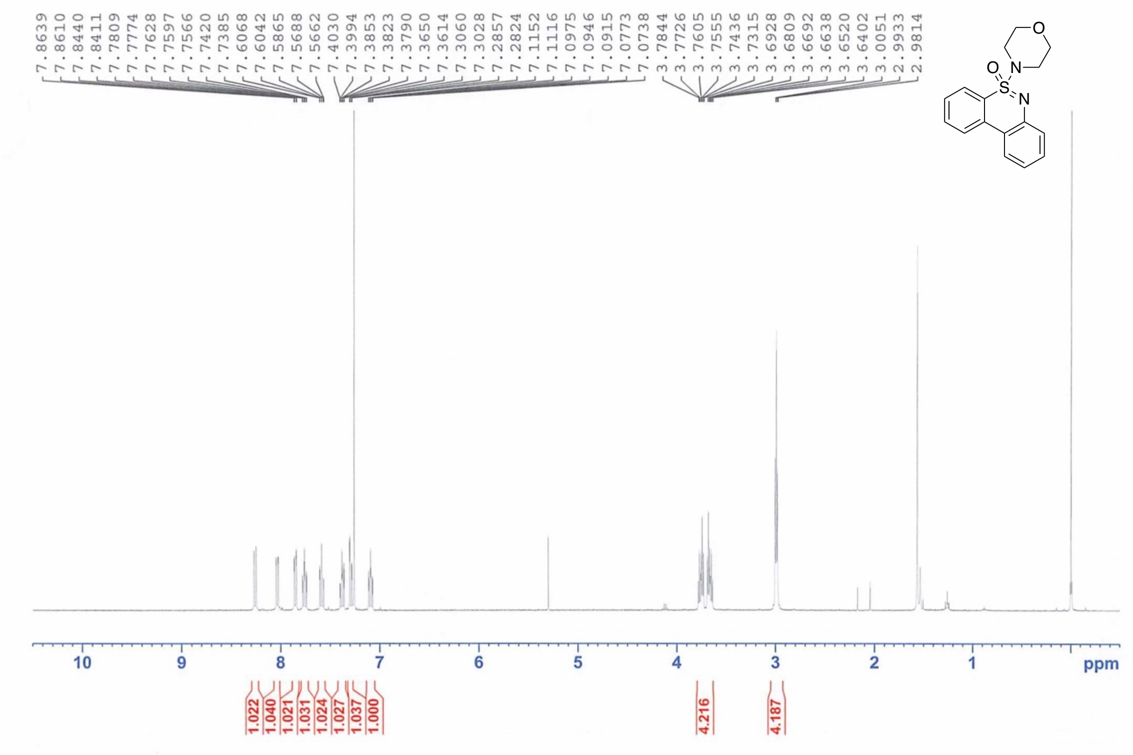

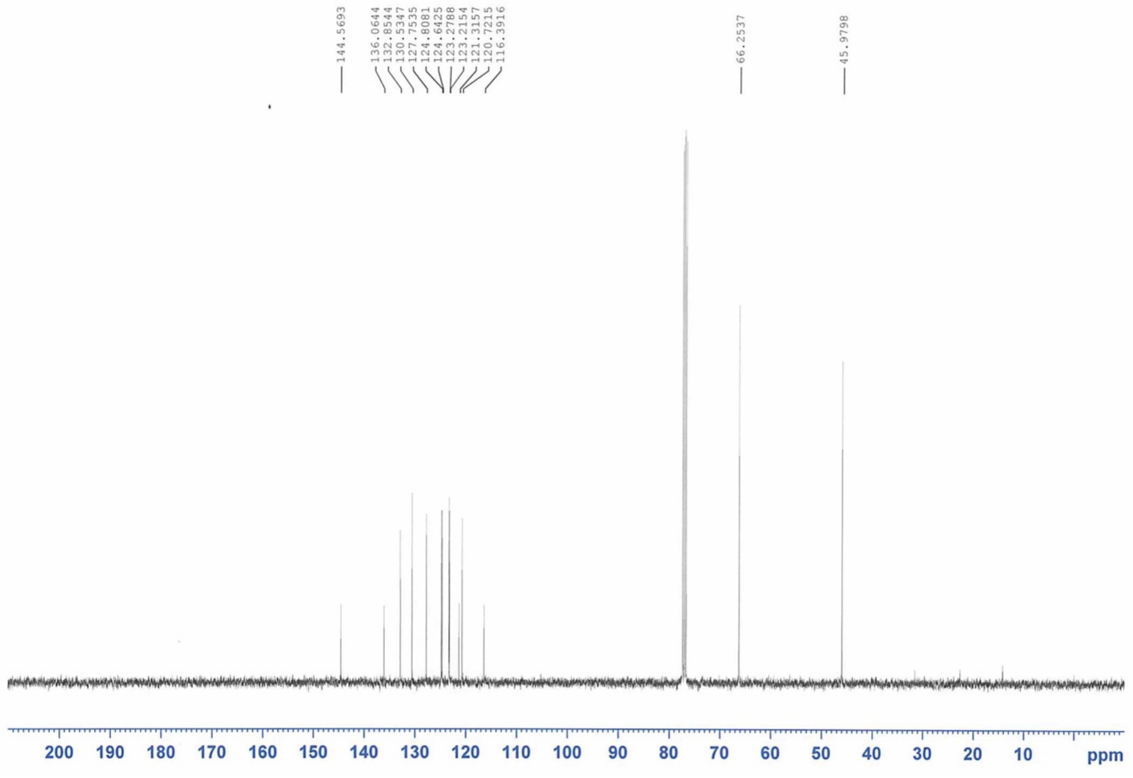

^{1}H NMR (400 MHz) and ^{13}C NMR (101 MHz) spectra of *tert*-butyl 5*H*-[1,3]dioxolo[4',5':4,5]benzo[1,2-*e*]benzo[*c*][1,2]thiazine-5-carboxylate 6-oxide (**3v**) ($CDCl_3$)

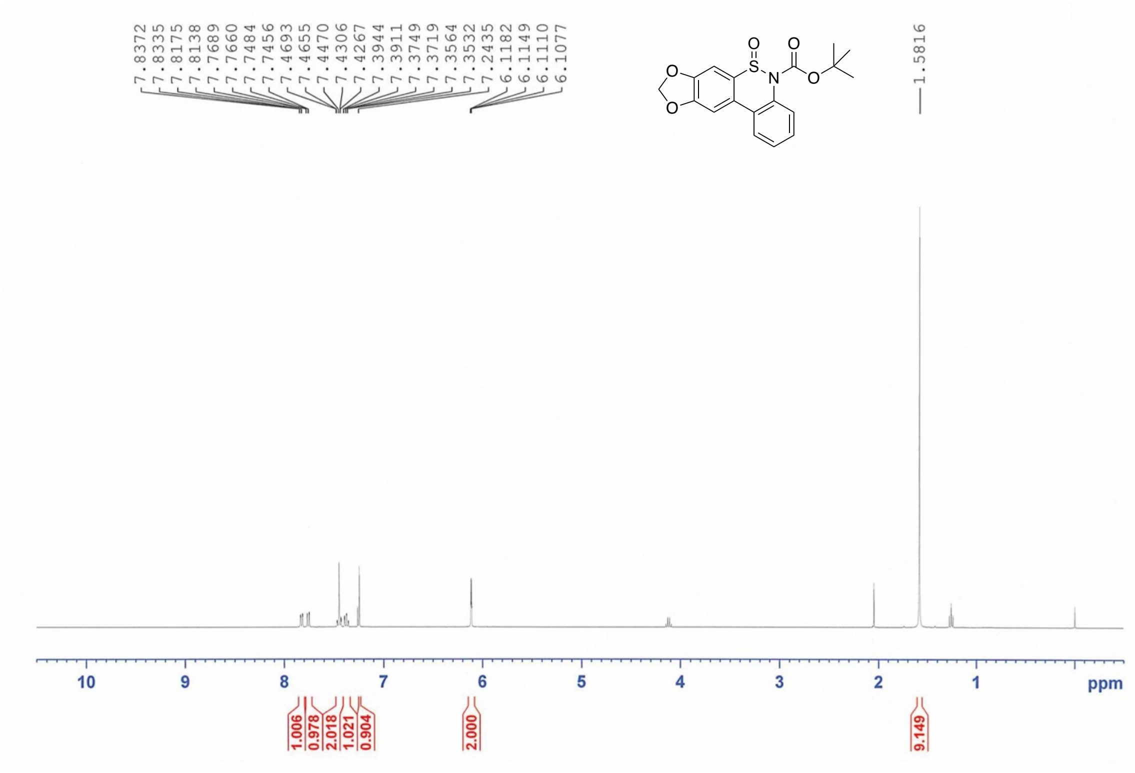

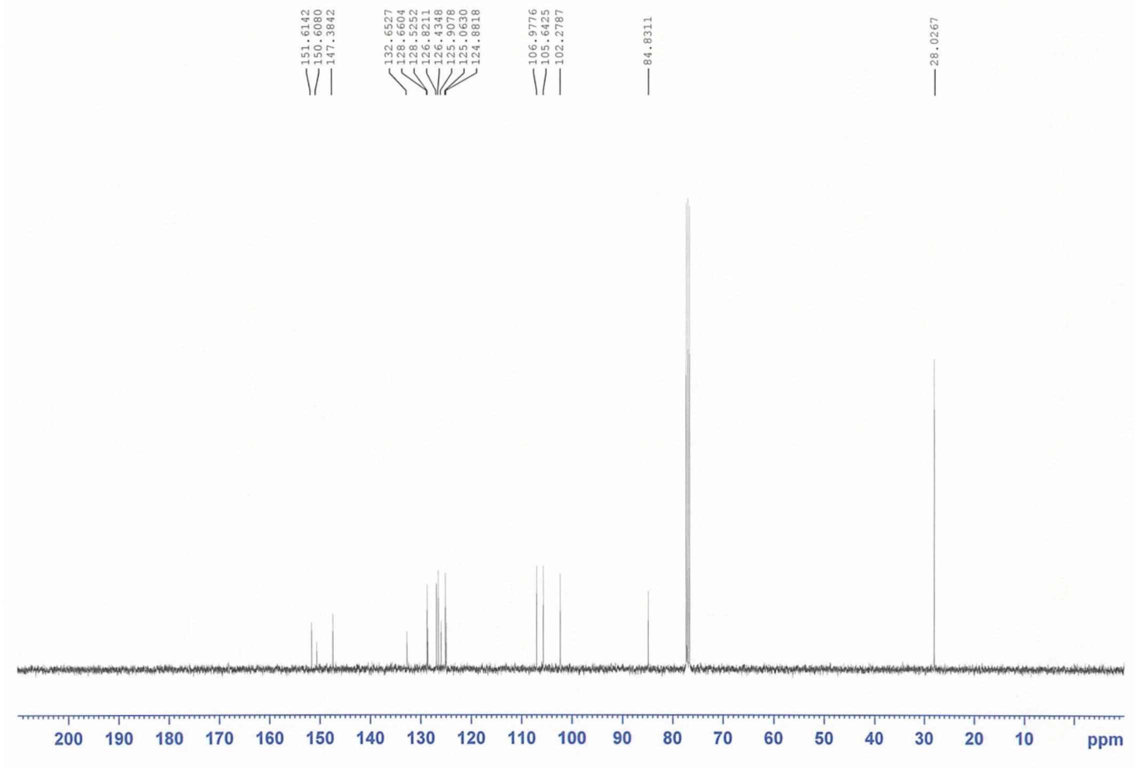

^{1}H NMR (400 MHz) and ^{13}C NMR (101 MHz) spectra of 5*H*-[1,3]dioxolo[4',5':4,5]benzo[1,2-*e*]benzo[*c*][1,2]thiazine 6-oxide (**5d**) ($CDCl_3$)

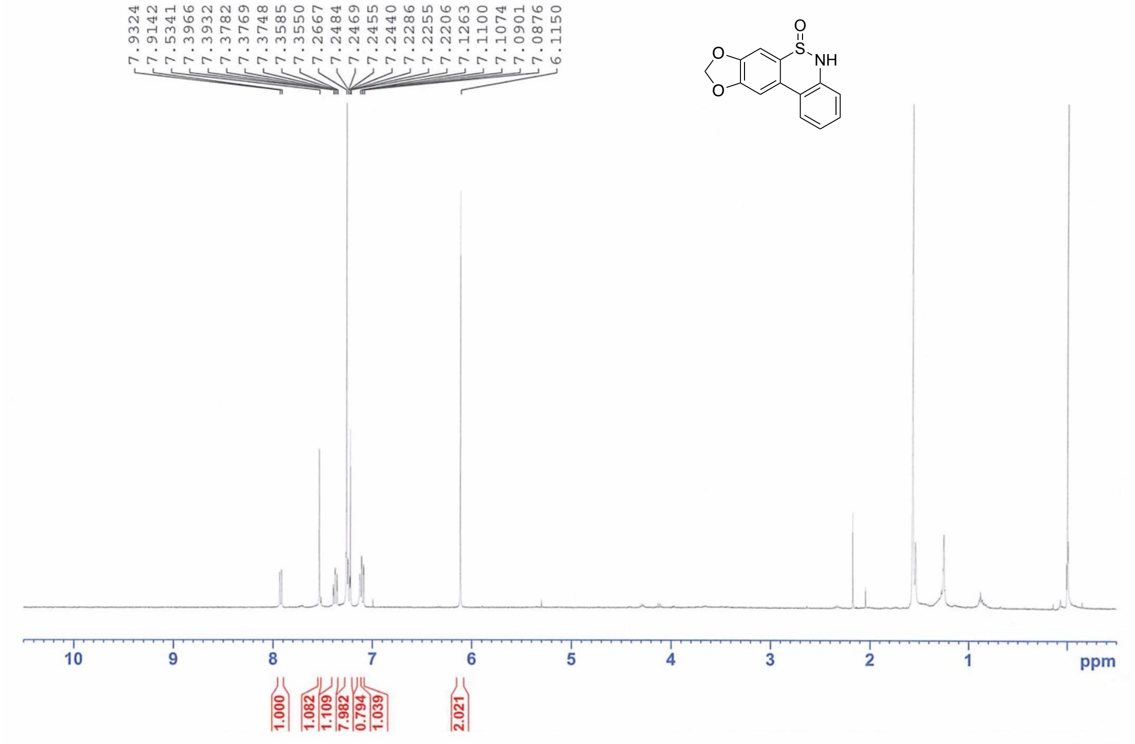

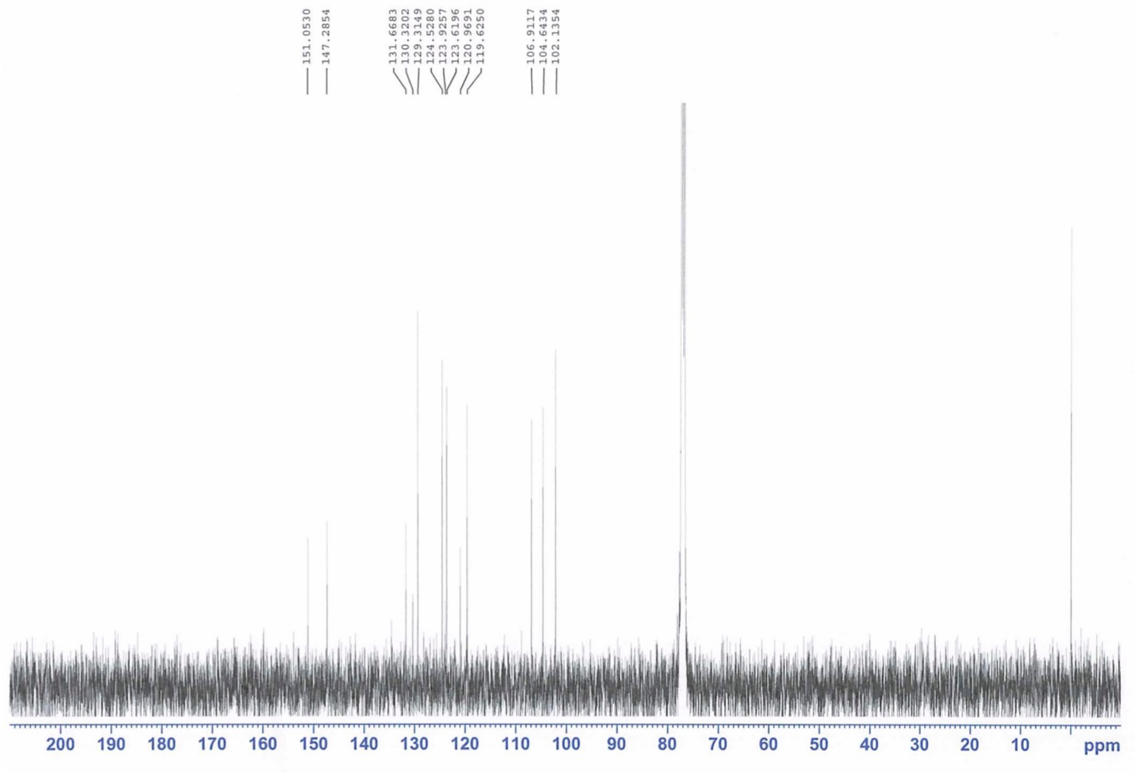

^{1}H NMR (400 MHz) and ^{13}C NMR (101 MHz) spectra of 5-(4-methoxyphenethyl)-5*H*-[1,3]dioxolo[4',5':4,5]benzo[1,2-*e*]benzo[*c*][1,2]thiazine 6-oxide (**17**) ($CDCl_3$)

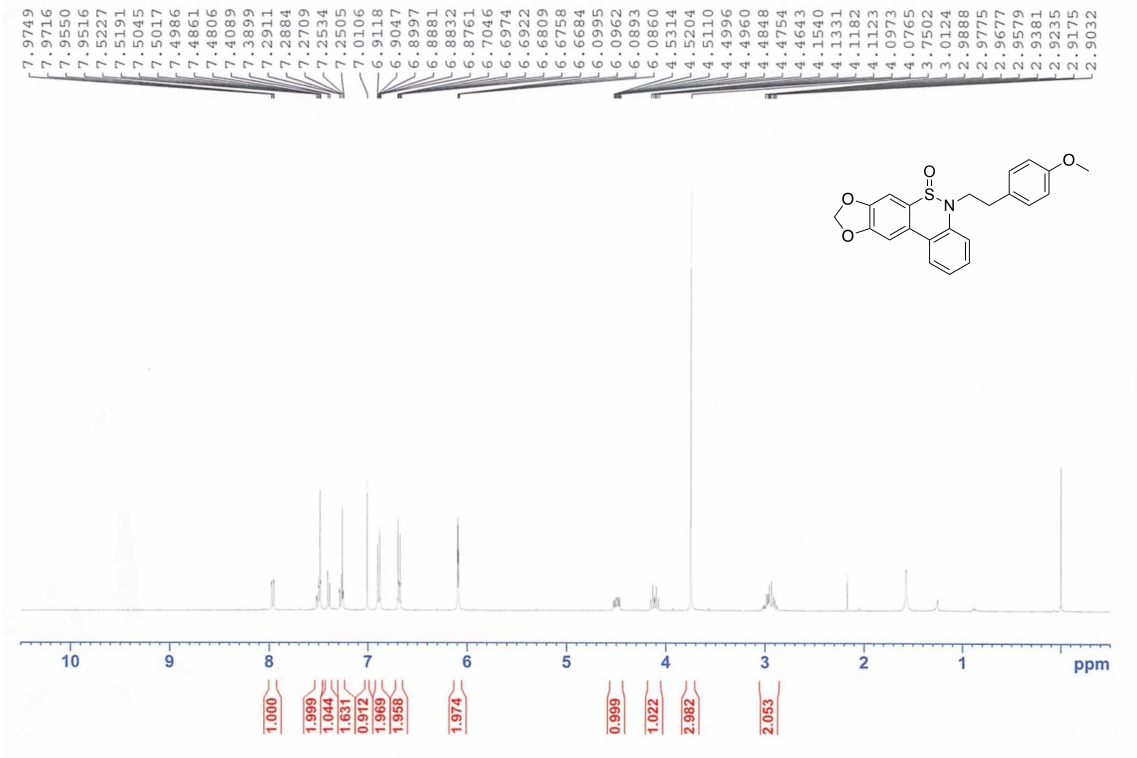

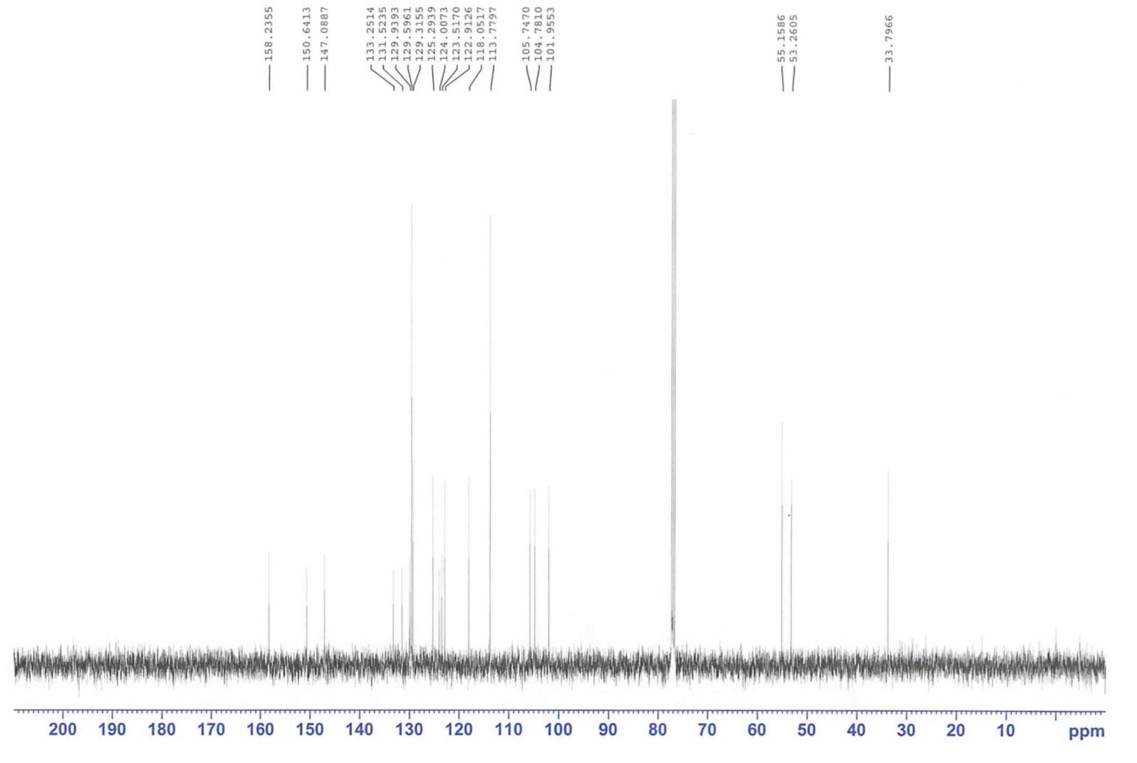

^{1}H NMR (400 MHz) and ^{13}C NMR (101 MHz) spectra of 3-bromo-4'-fluoro-4-iodo-1,1'-biphenyl ($CDCl_3$)

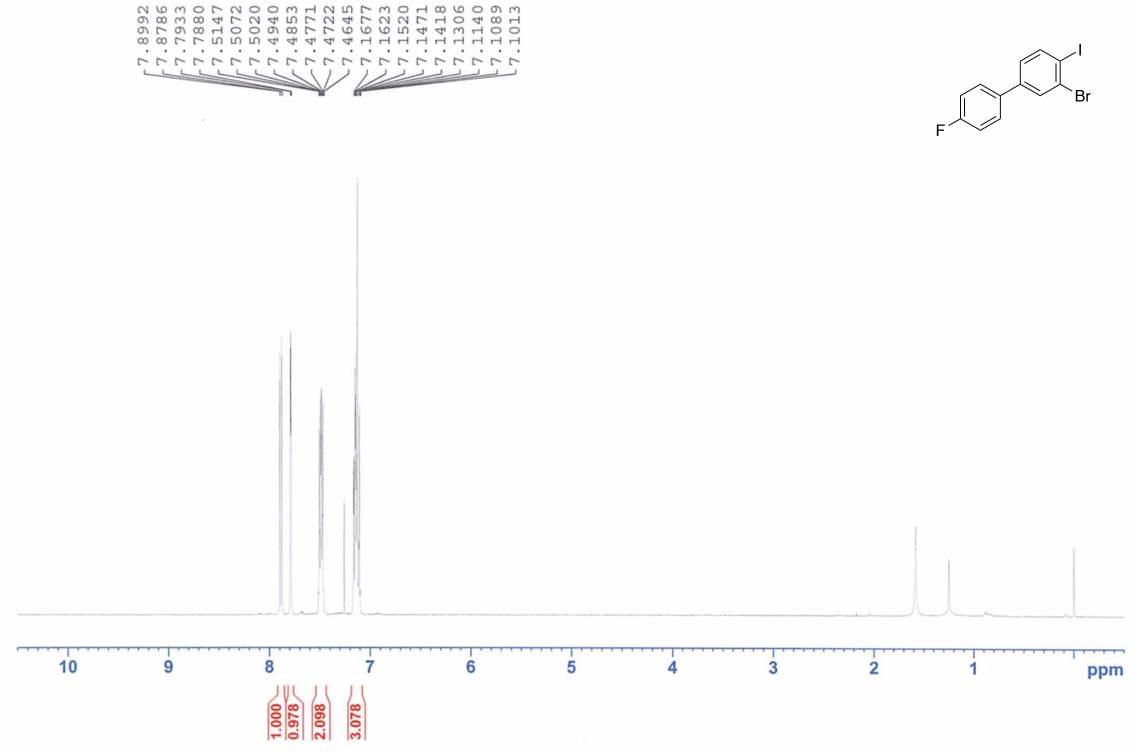

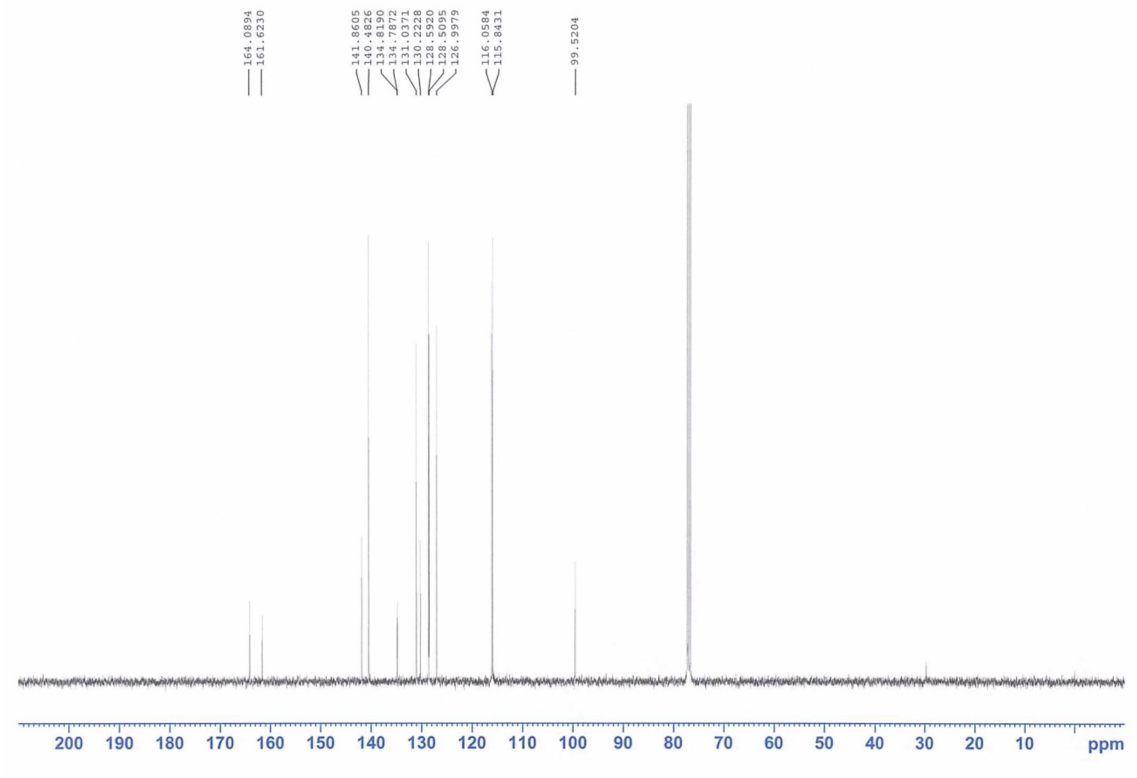

^{1}H NMR (400 MHz) and ^{13}C NMR (101 MHz) spectra of 3-bromo-4'-chloro-4-iodo-1,1'-biphenyl ($CDCl_3$)

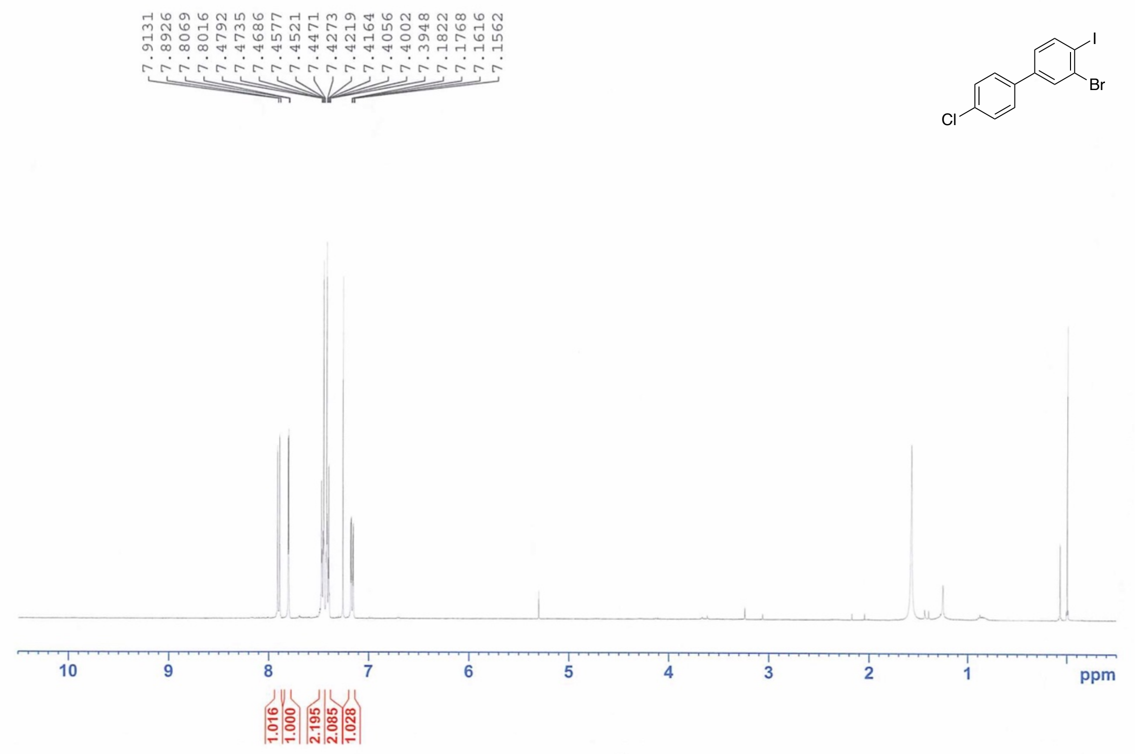

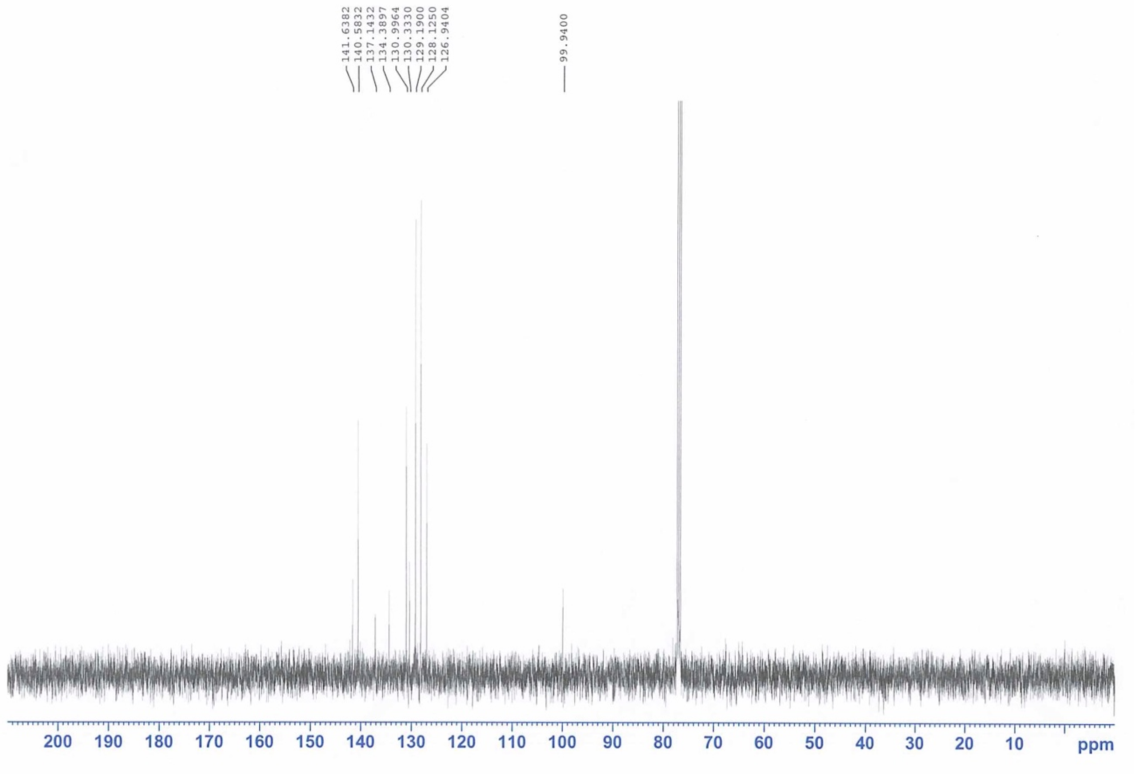

^{1}H NMR (400 MHz) and ^{13}C NMR (101 MHz) spectra of *tert*-butyl (4'-methyl-[1,1'-biphenyl]-4-yl)carbamate ($CDCl_3$)

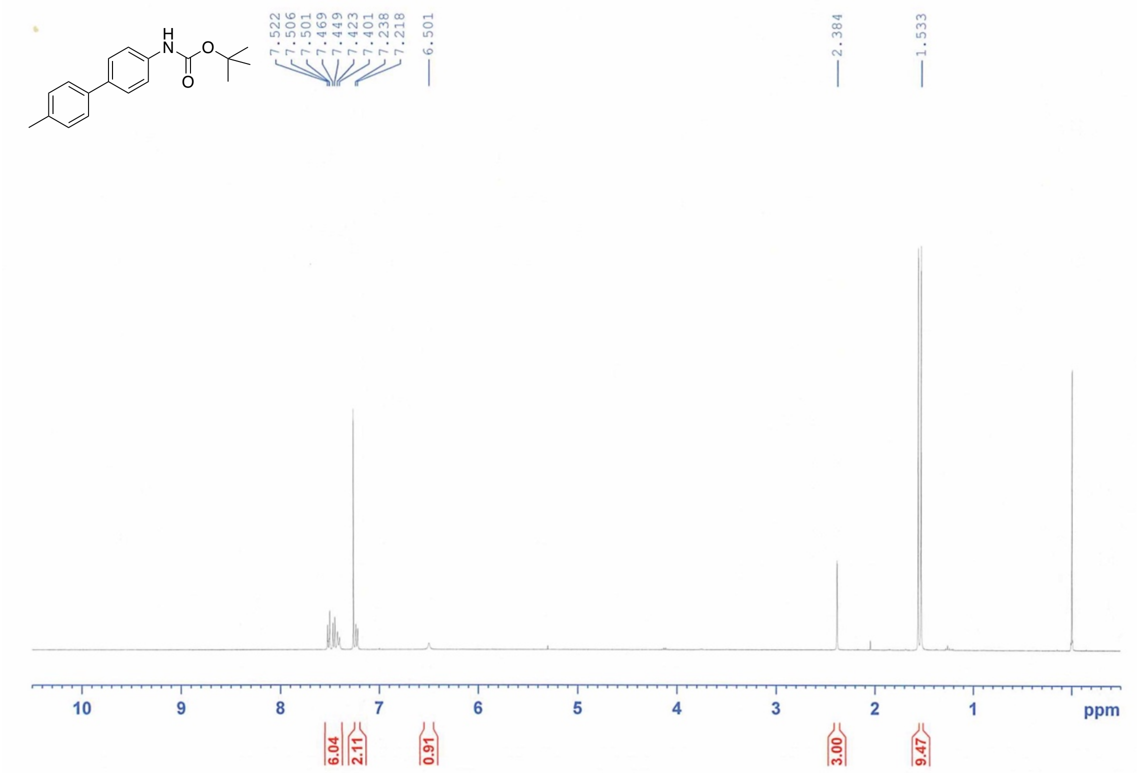

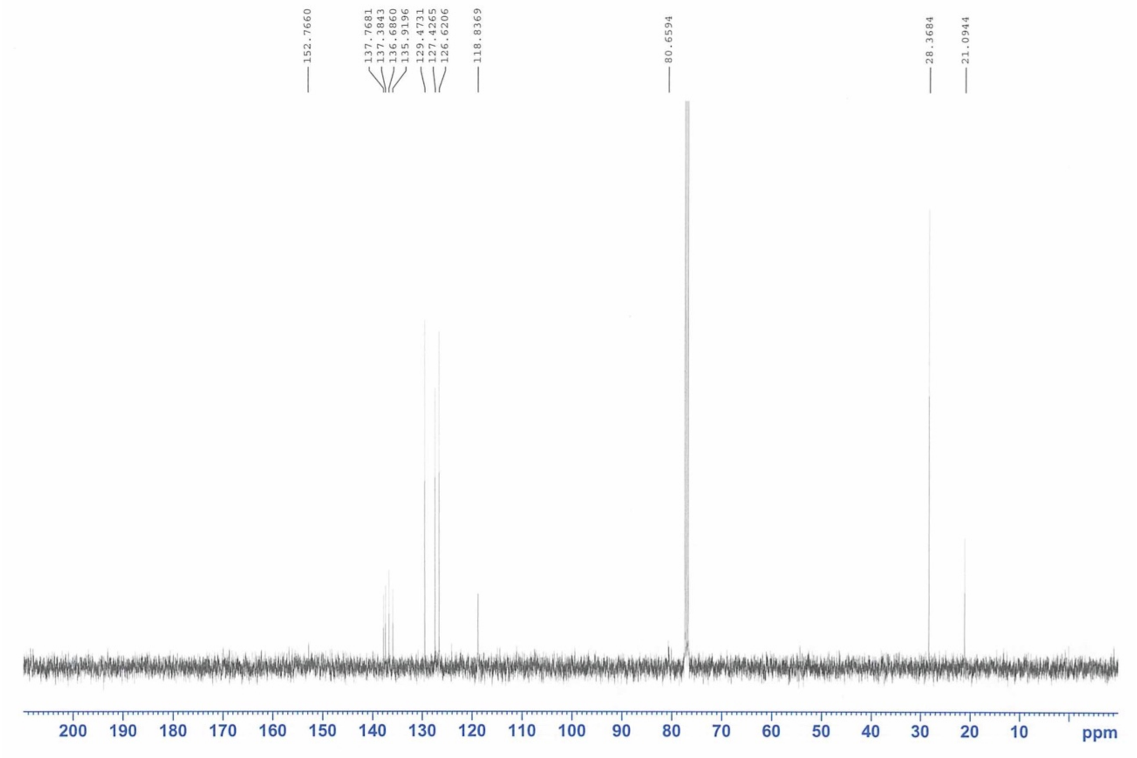

^{1}H NMR (400 MHz) and ^{13}C NMR (101 MHz) spectra of 3-bromo-4'-methyl-[1,1'-biphenyl]-4-amine ($CDCl_3$)

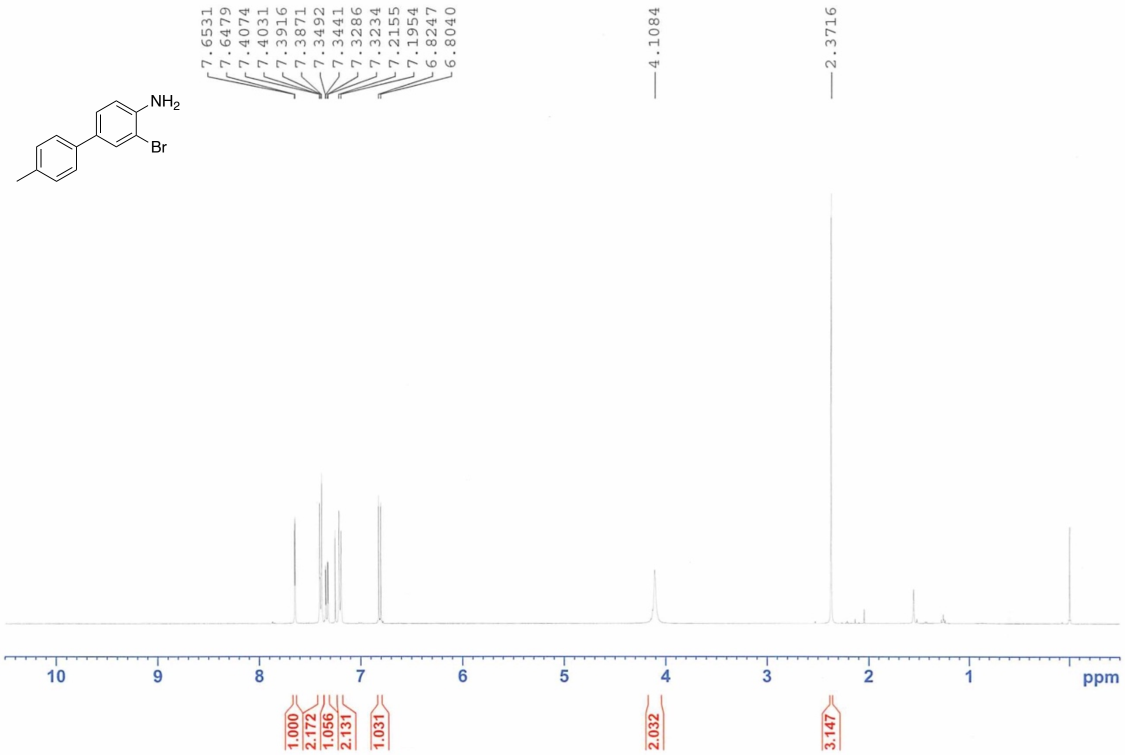

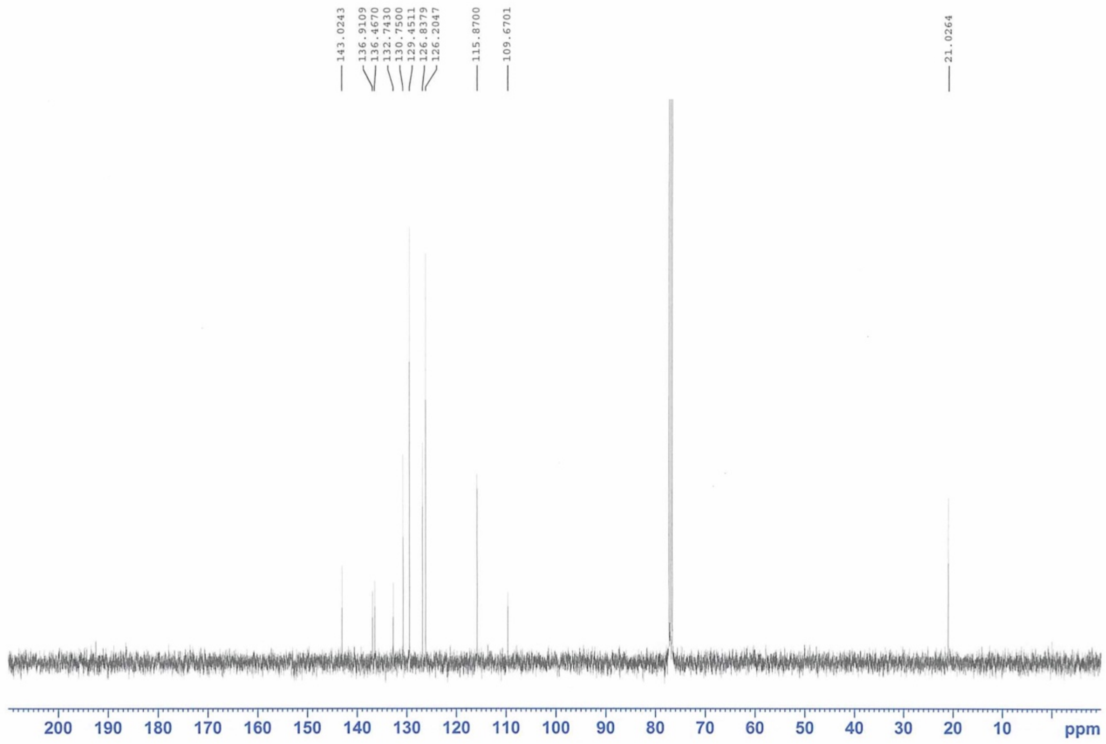

^{1}H NMR (400 MHz), ^{13}C NMR (101 MHz), and ^{11}B NMR (128 MHz) spectra of ethyl 4-amino-3-(4,4,5,5-tetramethyl-1,3,2-dioxaborolan-2-yl)benzoate ($CDCl_3$)

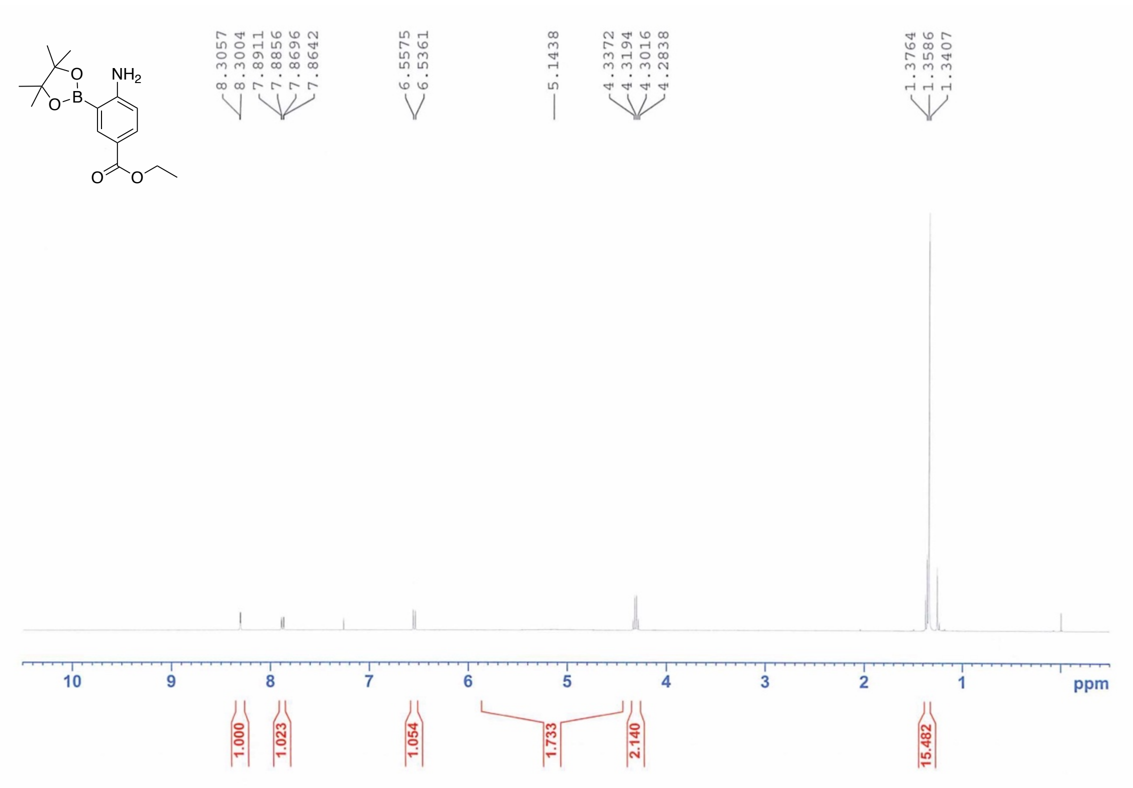

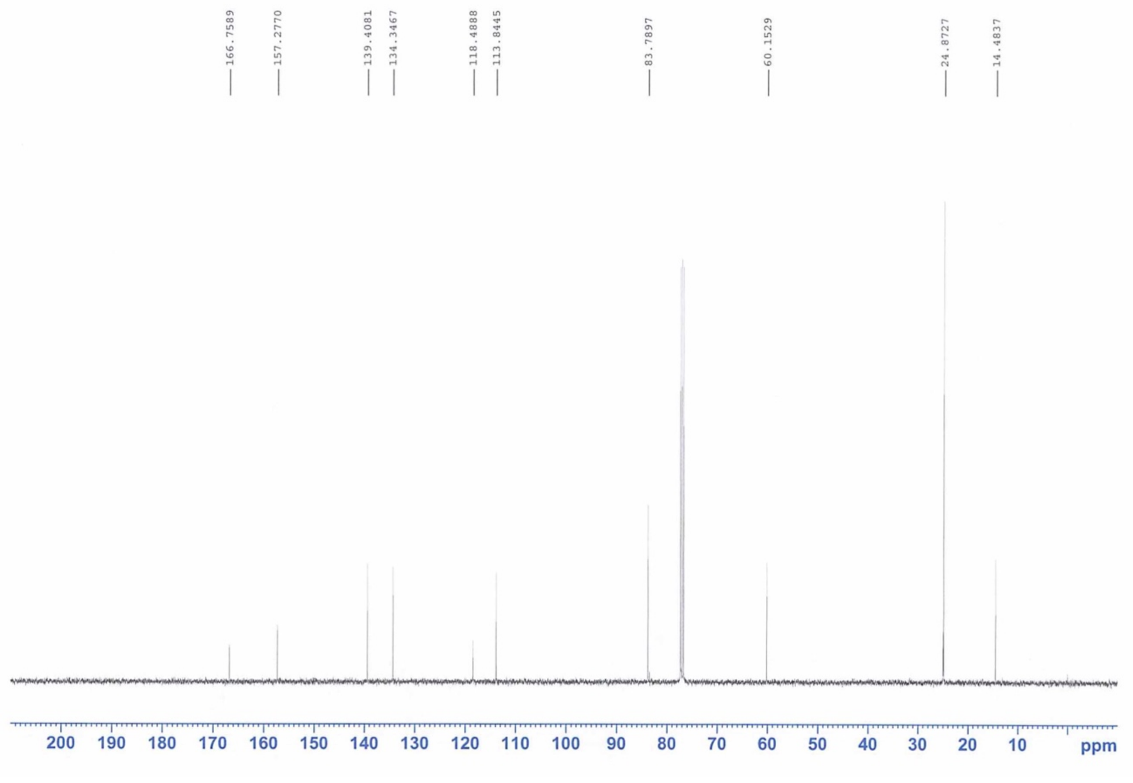

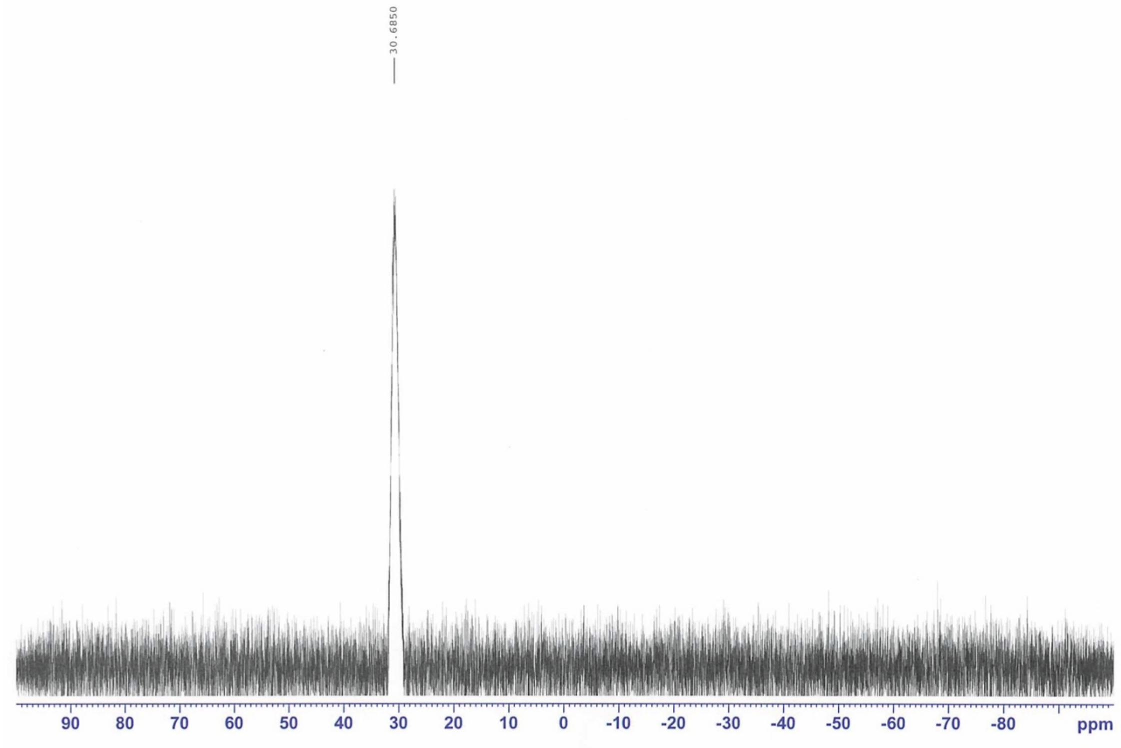
30.6850
90 80 70 60 50 40 30 20 10 0 -10 -20 -30 -40 -50 -60 -70 -80 ppm
